# Evaluating the Accuracy of Large Language Models in Risk-of-Bias Assessment Using Version 2 of the Cochrane Risk-of-Bias Tool for Randomized Trials: Exploratory Feasibility Study

**DOI:** 10.2196/84915

**Published:** 2026-09-25

**Authors:** Yu-Ju Lai, Shen-Hua Lin, Jen-Wei Liu

**Affiliations:** 1 Department of Pharmacy, Fu Jen Catholic University Hospital, Fu Jen Catholic University, No.69, Guizi Rd., Taishan Dist., New Taipei City, 24352, Taiwan; 2 Graduate Institute of Clinical Medicine, College of Medicine, Taipei Medical University, Taipei, Taiwan

**Keywords:** large language models, LLMs, risk of bias, version 2 of the Cochrane risk-of-bias tool for randomized trials, ROB 2, ChatGPT, randomized controlled trial, RCT, systematic reviews

## Abstract

**Background:**

Large language models (LLMs) have the potential to improve the efficiency of evidence synthesis, but their reliability in performing complex tasks such as risk-of-bias (ROB) assessment in randomized controlled trials (RCTs) remains unclear.

**Objective:**

This study aimed to evaluate whether LLMs can reliably assess ROB in RCTs using version 2 of the Cochrane ROB tool for randomized trials (ROB 2).

**Methods:**

This study was conducted between December 28, 2024, and February 28, 2025, in adherence to American Association for Public Opinion Research reporting guidelines. Twenty-nine RCTs were selected from published Cochrane systematic reviews across diverse medical fields. We developed a structured prompt engineering framework that transformed ROB 2 decision trees into logical rules for the LLM. Each RCT was independently evaluated twice by ChatGPT, with Cochrane review authors’ assessments serving as the reference standard for comparison. The main outcomes were the accuracy and consistency of ROB 2 assessments at both the domain and trial levels, evaluated using accuracy, sensitivity, specificity, and *F*_1_-score. Consistency between the repeated assessments was quantified using the Cohen κ and prevalence-adjusted, bias-adjusted κ.

**Results:**

The LLM demonstrated a moderate aggregate domain accuracy of 73.1% (95% CI 64.7%-81.5%) in the first assessment and 75.9% (95% CI 66.3%-85.4%) in the second assessment. Domain-averaged sensitivity decreased from 61.4% (95% CI 48.1%‐74.7%) to 53.4% (95% CI 41.7%‐65.0%), whereas domain-averaged specificity increased from 75.8% (95% CI 65.3%-86.3%) to 81.1% (95%CI 67.1%-95%), indicating a conservative tendency in identifying a high ROB. Domain-level accuracy ranged from 62.1% to 87.9%, with the lowest accuracy observed in domain 1 and the lowest *F*_1_-scores observed in domain 2. Consistency between repeated assessments was high, with a mean agreement of 89.0% (SD 7.5%), and Cohen κ values were 0.86, 0.39, 0.56, 0.84, and 0.85 in domains 1 to 5, respectively.

**Conclusions:**

In this exploratory study, ChatGPT demonstrated moderate accuracy and high consistency in assessing ROB in RCTs using the ROB 2. However, its reliability diminished in complex scenarios requiring interpretation of implicit narratives or behavioral nuance. These findings suggest that LLMs may support methodological evaluations in systematic reviews by acting as automated screeners to reduce reviewer burden, but current implementation still requires expert oversight, particularly for trials involving subjective outcomes or nonstandard reporting.

## Introduction

Systematic reviews play a foundational role in evidence-based medicine by synthesizing findings from primary studies to inform clinical decision-making, guideline development, and health policy [1]. As the volume of biomedical literature continues to grow rapidly, the demand for efficient, high-quality evidence synthesis has become more urgent. Among the key components of systematic reviews is the assessment of the risk of bias (ROB), particularly in randomized controlled trials (RCTs), which serve as the primary source of evidence for many clinical guidelines. The Grading of Recommendations Assessment, Development, and Evaluation (GRADE) framework emphasizes that the certainty of evidence is closely tied to the ROB in included studies within a systematic review [2].

To support rigorous and transparent ROB assessments, the Cochrane Collaboration developed version 2 of its ROB tool for randomized trials (ROB 2), which evaluates bias at the outcome level across 5 domains: randomization process, deviations from intended interventions, missing outcome data, measurement of the outcome, and selection of the reported results [3]. The tool relies on signaling questions and structured algorithms to facilitate reproducible judgments. Despite its methodological strengths, applying the ROB 2 remains time and resource intensive, requiring trained reviewers and substantial manual effort.

Large language models (LLMs), with their advanced capabilities in natural language understanding and reasoning, have been proposed as tools to automate complex tasks in medical text analysis [4]. Preliminary research suggests that LLMs may be able to interpret trial reports and mimic human judgment in various evaluative tasks. However, whether LLMs can perform structured ROB assessments in line with established tools such as the ROB 2 remains an open question [5]. To address this gap, we conducted a study to evaluate the feasibility, accuracy, and consistency of using LLMs to perform ROB assessments for RCTs guided by a structured prompt based on the ROB 2 framework.

## Methods

### Overview

This study was conducted between December 28, 2024, and February 28, 2025, in adherence to the American Association for Public Opinion Research reporting guidelines [6].

For this study, we used the GPT-o3-mini-high model via the web interface. OpenAI’s GPT-o3-mini has been optimized for science, technology, engineering, and mathematics reasoning, with medium reasoning effort matching the performance of GPT-o1 in math, coding, and science while delivering faster responses [7]. Due to the technical constraints of the ChatGPT web interface, all assessments were performed using the model’s default settings as manual adjustments to parameters such as temperature and top_p are not supported in this environment. This model was used to systematically assess the ROB based on the ROB 2 in RCTs using its reasoning capabilities to simulate the evaluation process conducted in systematic reviews.

### Ethical Considerations

We used only publicly available published literature and LLMs without involving any personal or identifiable private data. This study followed the “Scope of Human Research Projects Exempt from Institutional Review Board Review” (1010265075) [8] issued by the Ministry of Health and Welfare, Taiwan. As this study relied solely on legally and publicly disclosed information, it was exempt from ethical review or informed consent requirements.

### Model Training and Prompt Development

Prompt development followed the logic and structure outlined in the ROB 2 guidance document [9]. Systematic prompt engineering techniques were developed to create structured prompts, allowing ChatGPT to perform tasks.

**Figure 1.**
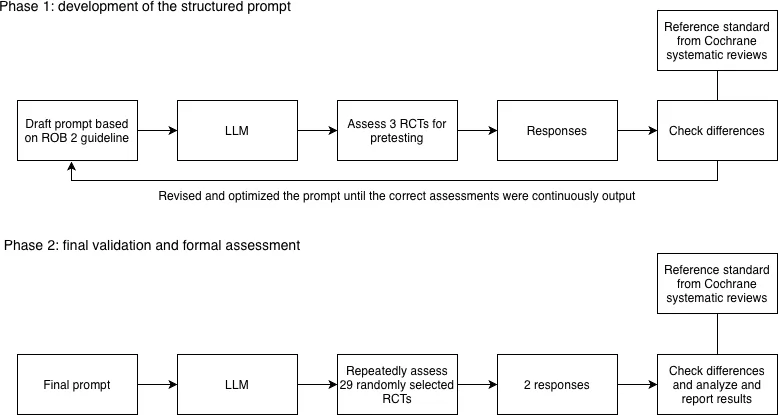
Flow diagram of the main study process. LLM: large language model; RCT: randomized controlled trial; ROB 2: version 2 of the Cochrane risk-of-bias tool for randomized trials; SR: systematic review.

We used 3 RCTs as pilot data, which were excluded from the final analytic sample. An iterative refinement process was used referencing published systematic reviews as a reference standard. On the basis of the framework by Lai et al [5], we defined the LLMs’ character, provided foundational definitions of the ROB 2 domains, and systematically transformed the official ROB 2 decision trees into logical rules within the prompt. When model-generated ROB judgments differed from the original assessments, prompts were revised and tested until alignment with expert assessments was consistently achieved. Figure 1 shows the main study process, and the final prompt can be found in Multimedia Appendix 1.

### Selection of Sample

We conducted a search of the PubMed database to identify Cochrane systematic reviews that used the ROB 2 for assessing ROB in RCTs. Two reviewers (YJL and JWL) independently screened the full texts of the retrieved systematic reviews to determine eligibility. Reviews were excluded if they did not provide detailed ROB assessments, such as an ROB table. From the eligible 178 Cochrane systematic reviews, we first randomized the identified reviews using numbers generated via Google Sheets and sequentially screened them until a pooled baseline of over 100 RCTs was established. Second, using the same random number generation method, we selected 30 RCTs from this pool for analysis to mitigate potential clustering effects. These 30 RCTs were distributed across 11 reviews. Of the 30 RCTs, 1 (3.3%) was excluded due to incomplete reporting of ROB assessment, resulting in a final analytic sample of 29 (96.7%) RCTs. Notably, one of the included trials, that by Maher et al [10], was retracted after our study period [11]. Because its Cochrane assessment contributed to our reference standard, we retained this trial in the primary analytic sample but conducted a sensitivity check to evaluate its impact.

### Application of ChatGPT for ROB Assessment

We used the GPT-o3-mini-high model for its strong reasoning capabilities, enabling consistent and accurate application of structured prompts in the ROB assessment process.

Each RCT was evaluated using a new ChatGPT conversation to ensure a clean contextual slate. The reviewers defined the primary outcome, provided domain-specific criteria, and uploaded the full trial text. ChatGPT then assessed ROB across the 5 domains in the ROB 2 framework, offering domain-level judgments with rationale (Multimedia Appendix 2). Each trial was assessed twice under identical conditions. In cases of system interruption, the session was discarded and repeated. A standardized interaction protocol was followed to maximize consistency and reproducibility.

### Establishment of the Reference Standard

The criterion standard for comparison was the ROB assessment reported by the Cochrane systematic review authors using the ROB 2 (Table S1 in Multimedia Appendix 3). These Cochrane assessments were selected for their rigorous methodology and multidisciplinary collaboration, which minimize bias and ensure clinical diversity [12,13]. When apparent errors were identified (eg, typographical mistakes), authors were contacted for clarification. If no response was received, the original Cochrane systematic review assessments were retained as the reference standard to ensure objectivity. A sensitivity analysis was then performed to evaluate the impact of potential errors using data that were manually corrected based on research team consensus.

### Discrepancy Categorization and Analysis

To evaluate the discrepancies between the LLM and the reference standard, we conducted an error analysis. Two researchers (YJL and JWL) independently categorized the discrepancies by comparing the LLM-generated rationales against the Cochrane reviewers’ evidence. Any disagreements were resolved through consensus.

Discrepancies were categorized as data extraction differences if there was a fundamental mismatch in the explicit information or evidence retrieved from the RCT between the LLM and the Cochrane reviewers. Conversely, they were classified as judgment differences if the LLM accurately extracted the same key points as the human reviewers but applied a different logical interpretation leading to a conflicting judgment.

### Statistical Analysis

The LLM was prompted to answer individual signaling questions using “yes,” “probably yes,” “no,” “probably no,” and “no information.” Signaling responses were then operationally mapped to the official “low risk” and “high risk” categories following the ROB 2 guideline. ROB domain judgments were categorized as follows: “low risk” was defined as a negative outcome, whereas “some concerns” and “high risk” were grouped as a positive outcome. On the basis of this classification, we calculated true positives (TPs), true negatives (TNs), false positives (FPs), and false negatives (FNs).

Model performance was evaluated using accuracy, sensitivity, specificity, precision, and *F*_1_-score. For domain-level analyses, sensitivity and specificity were macroaveraged across domains. Metrics were defined as follows:

Accuracy = (TPs + TNs)/total number of assessments

Sensitivity = TPs/(TPs + FNs)

Specificity = TNs/(TNs + FPs)

Precision = TPs/(TPs + FPs)

*F*_1_-score = 2 × (precision × sensitivity)/(precision + sensitivity)

To assess internal consistency, we computed the Cohen κ and the prevalence-adjusted, bias-adjusted (PABA) κ:

Cohen κ = (*P*_*o*_ − *P*_*e*_)/(1 − *P*_*e*_)

PABA κ = 2 × *P*_*o*_ – 1

In these equations, *P_o_* is calculated as (number of agreements on positive + number of agreements on negative)/total number of assessments.

*P*_*e*_ = [(*P*_1_ × *P*_2_) + (*N*_1_ × *N*_2_)]/(total number of assessments)^2^

For trials in which the model provided identical ratings across both assessments, the Cohen κ was mathematically undefined due to zero variance, which resulted in a zero denominator in the κ calculation and was therefore reported as “Not available.” The agreement thresholds in Table S2 in Multimedia Appendix 3 followed standard interpretations: κ values of 0.81 to 0.99 indicated near-perfect agreement. Additionally, exploratory subgroup analyses by clinical discipline were performed to investigate potential variability in model performance across different medical contexts. Trials were categorized based on the primary medical focus of the RCT and the scope of the parent Cochrane review from which the trial was extracted. In cases in which a trial potentially spanned multiple disciplines, the final categorization was determined through discussion and consensus between 2 authors (YJL and JWL). All statistical analyses were conducted using Google Sheets.

## Results

### Characteristics of the RCTs

The final dataset included 29 RCTs [10,14-41] extracted from 11 Cochrane systematic reviews [42-52]. These trials represented a spectrum of medical disciplines, including cardiology (n=5, 17.2%), psychology (n=6, 20.7%), infectious diseases (n=6, 20.7%), gastroenterology (n=5, 17.2%), pulmonology (n=4, 13.8%), bone health (n=1, 3.4%), and obstetrics and gynecology (n=2, 6.9%). Primary efficacy-related outcomes varied across studies, including binary outcomes (n=18, 62.1%), with most focusing on all-cause mortality and asthma exacerbation or treatment success. Continuous outcomes (n=11, 37.9%) mainly included pain, physical function, and quality of life. The trials were published between 2000 and 2024. All RCTs were published in English and assessed for ROB using the ROB 2, ensuring a standardized approach to bias evaluation.

### Accuracy

#### Aggregate Domain and Overall Accuracy

**Figure 2.**
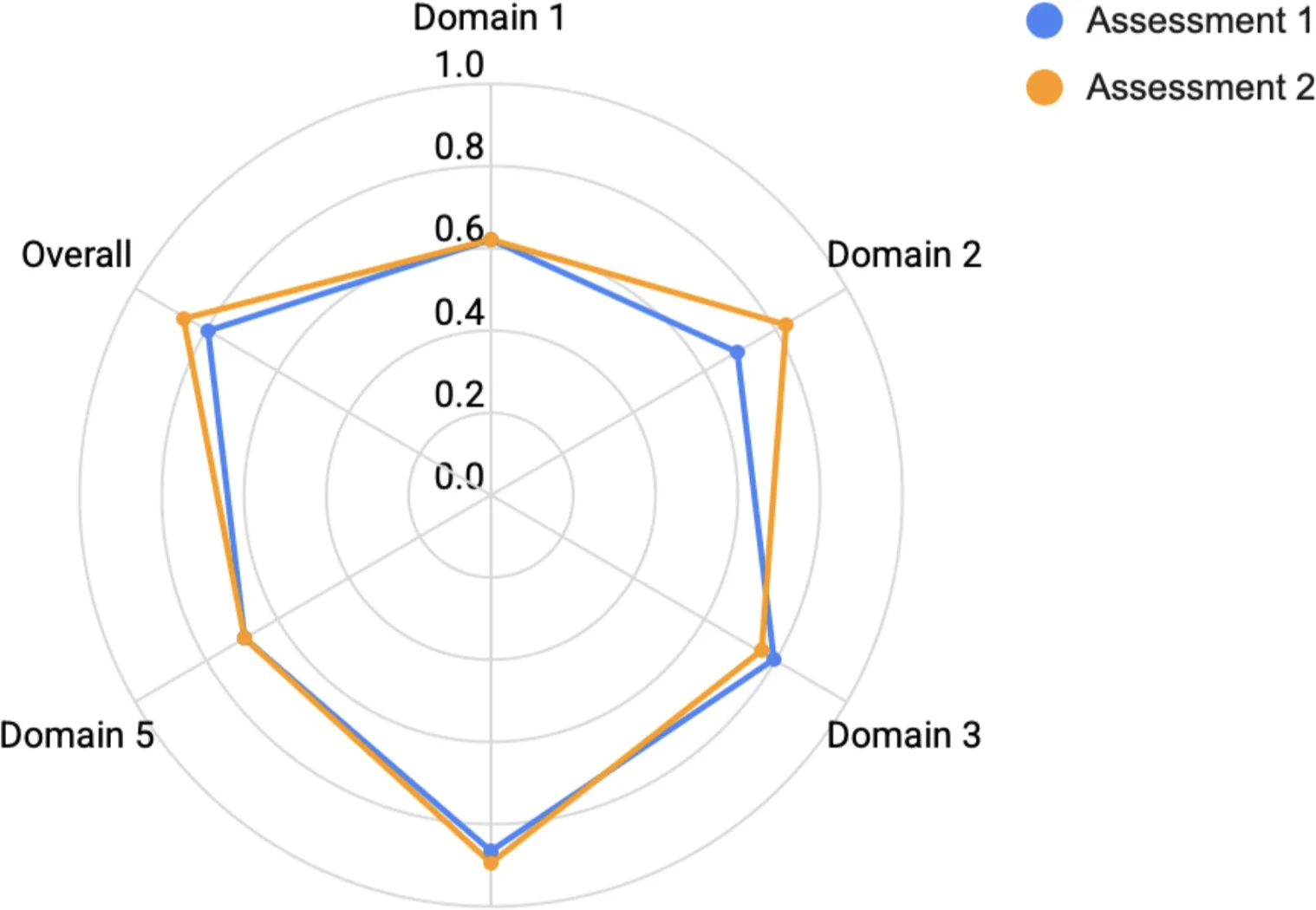
Radar chart of accuracy in domains.

The LLM underwent 2 independent assessments for ROB evaluation, as shown in Table S3 in Multimedia Appendix 3. The aggregate domain accuracy (Table 1 and Figure 2) was comparable between assessments at 73.1% (95% CI 64.7%-81.5%) for the first and 75.9% (95% CI 66.3%-85.4%) for the second, reflecting a marginal improvement in accuracy in the second assessment (relative difference [RD] 2.8%, 95% CI –3.1% to 8.6%).

**Table 1.** Domain-specific accuracy of assessments.

	TPs^a^, n	TNs^b^, n	FPs^c^, n	FNs^d^, n	Accuracy, %	Sensitivity, %	Specificity, %	Precision, %	*F*_1_-score, %
Domain 1									
Assessment 1	5	13	9	2	62.1	71.4	59.1	35.7	47.6
Assessment 2	5	13	9	2	62.1	71.4	59.1	35.7	47.6
Domain 2									
Assessment 1	3	17	5	4	69.0	42.9	77.3	37.5	40.0
Assessment 2	3	21	1	4	82.8	42.9	95.5	75.0	54.6
Domain 3									
Assessment 1	4	19	5	1	79.3	80.0	79.2	44.4	57.1
Assessment 2	2	20	4	3	75.9	40.0	83.3	33.3	36.4
Domain 4									
Assessment 1	2	23	2	2	86.2	50.0	92.0	50.0	50.0
Assessment 2	2	24	1	2	89.7	50.0	96.0	66.7	57.1
Domain 5									
Assessment 1	5	15	6	3	69.0	62.5	71.4	45.5	52.6
Assessment 2	5	15	6	3	69.0	62.5	71.4	45.5	52.6

a TP: true positive.

b TN: true negative.

c FP: false positive.

d FN: false negative.

In contrast, the overall ROB accuracy, derived from the final overall ROB judgment for each trial, was 79.3% (23/29) in the first assessment and 86.2% (25/29) in the second.

Sensitivity, reflecting the ability to detect high-risk judgments, was higher for the first assessment at 61.4% (95% CI 48.1%-74.7%) compared to 53.4% (95% CI 41.7%-65.0%) in the second, suggesting slightly reduced effectiveness in TP identification by the second assessment (RD 8.0%, 95% CI –7.7% to 23.7%). Specificity remained high in both assessments, increasing from 75.8% (95% CI 65.3%-86.3%) in the first to 81.1% (95% CI 67.1%-95.0%) in the second, demonstrating strong performance in identifying low-risk judgments. *F*_1_-scores were similar between assessments, with the first one slightly lower at 49.5% (95% CI 43.9%-55.1%) compared to 49.7% (95% CI 42.5%-56.9%) in the second.

#### Domain-Specific Accuracy

Across the 5 ROB 2 domains, the mean accuracy was 74.5% (95% CI 66.0%-83.0%) based on pooled results from 2 assessments. The lowest accuracy was observed in domain 1 (randomization process) at 62.1%, whereas the highest was observed in domain 4 (outcome measurement) at 87.9%. A total of 74 discrepancies were identified in ROB assessments, with 55 (74.3%) resulting from differences in judgments between the LLM and the reference standard and 19 (25.7%) arising from inconsistencies in data extraction. Domain 1 (randomization process) exhibited the highest number of total discrepancies (22/74, 29.7%), with 68.2% (15/22) related to judgment and 31.8% (7/22) related to data extraction. Domain 5 (selection of the reported results) accounted for 24.3% (18/74) of discrepancies, including 66.7% (12/18) judgment-related and 33.3% (6/18) extraction-related differences. Domains 2 and 3 showed intermediate discrepancy rates (14/74, 18.9% and 13/74, 17.6%, respectively), whereas domain 4 exhibited the fewest discrepancies (7/74, 9.5%), predominantly due to judgment differences.

Sensitivity ranged from 42.9% in domain 2 to 71.4% in domain 1, highlighting variability in identifying high-risk judgments. Specificity was consistently high (range 59.1%-94%), indicating reliable identification of low-risk judgments. The *F*_1_-score was highest in domain 4 (53.3%) and lowest in domain 2 (46.2%), highlighting the influence of both sensitivity and precision on performance in nuanced domains.

#### Trial-Specific Accuracy

**Figure 3.**
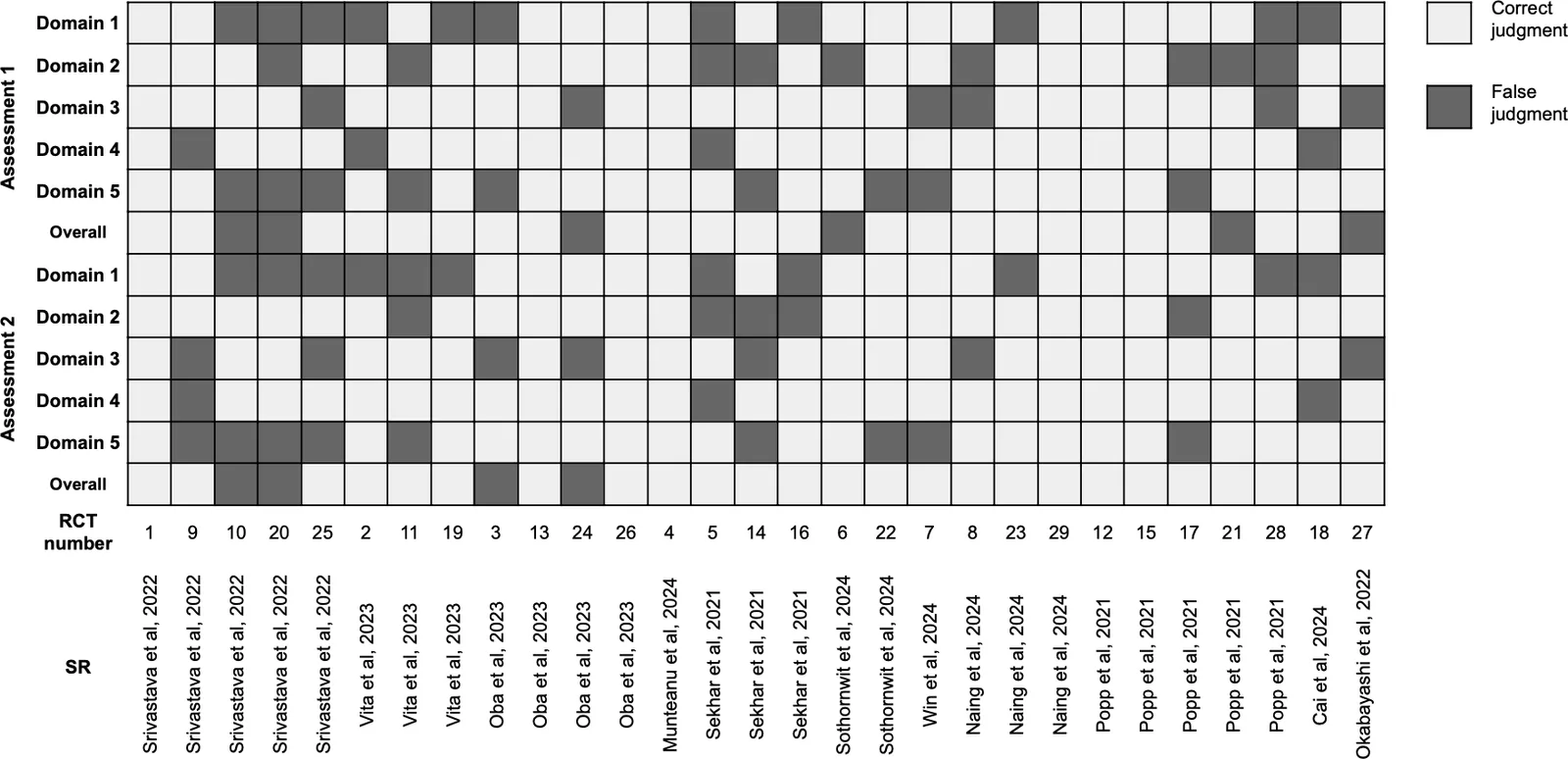
Heat map of accuracy across trial- and domain-specific contexts [42-52]. RCT: randomized controlled trial; SR: systematic review.

Across 58 assessments for the 29 trials (Table S4 in Multimedia Appendix 3), 7 (24.1%) trials achieved full accuracy (100% correct), whereas 7 (24.1%) trials attained an average accuracy between 80% and 90%. In total, 51.7% (15/29) of the trials had an accuracy of 70% or lower, with the lowest at 40% (Figure 3 [42-52]).

Performance varied across medical disciplines, although these findings were limited by small subgroup sample sizes. The reported counts represent aggregated domain-level assessments across 5 ROB 2 domains and 2 assessment cycles per trial. For bone health (1/29, 3.4%), the single trial was correctly judged in both assessments. For other disciplines, we observed the following aggregated domain-level accuracies: in gastroenterology, 39 out of 50 domains were correctly assessed (across 5/29, 17.2% of the trials); in infectious diseases, 48 out of 60 domains were correctly assessed (across 6/29, 20.7% of the trials); in pulmonology, 35 out of 40 domains were correctly assessed (across 4/29, 13.8% of the trials); and in obstetrics and gynecology, 17 out of 20 domains were correctly assessed (across 2/29, 6.9% of the trials). Cardiology and psychology exhibited the lowest accuracy, with 31 out of 50 domains (across 5/29, 17.2% of the trials) and 36 out of 60 domains (across 6/29, 20.7% of the trials) correctly assessed, respectively, with no domains in either discipline achieving full accuracy in the first or second assessment.

### Sensitivity Analysis

Notably, a typographical error in the initial assessments by Toouli et al [20] was identified in domains 2, 3, and 4. After attempting to contact the study authors without receiving a response, the research team conducted a sensitivity analysis to evaluate the impact of manual adjudications on the reference standard. Correction of the typographical error led to improvements in domain-specific performance. In the first assessment, *F*_1_-scores increased from 40.0% to 50.0% for domain 2 and from 57.1% to 61.5% for domain 3, whereas accuracy increased from 69.0% to 72.4% for domain 2 and from 79.3% to 82.8% for domain 3. In the second assessment, while the *F*_1_-score for domain 3 improved from 36.4% to 40.0% and its accuracy rose from 75.9% to 79.3%, the *F*_1_-score for domain 2 paradoxically decreased from 54.6% to 50.0%, and accuracy decreased from 82.8% to 79.3%. This decrease occurred because the LLM’s original judgment in assessment 2 happened to align with the uncorrected Cochrane reference. After manual correction of the reference standard to reflect the true methodological assessment, the LLM’s response was reclassified as discrepant. Additionally, we performed a sensitivity check by excluding the subsequently retracted trial by Maher et al [10]. Removing this single trial resulted in a slight decrease in the aggregate domain accuracy, shifting from 73.1% to 72.9% in the first assessment and from 75.9% to 75% in the second. These changes were of less than 1 percentage point and did not materially alter the overall trends or conclusions regarding the LLM’s performance. Following exclusion of this trial, obstetrics and gynecology was represented by 1 remaining trial in which 8 of 10 domains across the 2 assessments were correctly classified; this result was therefore reported descriptively.

### Consistency

The assessment rate (observed agreement; *P*_*o*_) demonstrated high consistency across domains, with a mean of 89.0% (SD 7.5%), ranging from 79.3% to 96.6%. Cohen κ values indicated near-perfect agreement in domains 1, 4, and 5 (κ=0.86, 0.84, and 0.85, respectively); moderate agreement in domain 3 (κ=0.56); and fair agreement in domain 2 (κ=0.39). PABA κ values followed similar trends (Table S5 in Multimedia Appendix 3).

At the trial level, of all 29 RCT assessments, 17 (58.6%) achieved full consistency (proportion of agreement; *P_o_*=1.00) across all domains, whereas 9 (31%) attained a proportion of agreement of exactly 0.80.

The Cohen κ was reported as “not available” due to zero variance in 24.1% (7/29) of the trials. The mean agreement across all studies was 0.89 (SD 0.16), reflecting high consistency (Table S6 in Multimedia Appendix 3).

## Discussion

### Principal Findings

In this study, we used GPT-o3-mini-high, a compute-intensive and reasoning-focused LLM to conduct structured ROB assessments for RCTs using the ROB 2. The LLM’s performance reflected a hybrid cognitive task: first performing semantic reasoning to extract and interpret complex clinical narratives for answering signaling questions and subsequently strictly following instructions by applying the structured logic of the ROB 2 decision tree. The judgments by review authors from published Cochrane systematic reviews served as a widely recognized reference standard for assessing the generalizability and representativeness of the LLM’s evaluations.

The LLM demonstrated a moderate mean accuracy of 74.5%. At the domain level, the averaged sensitivity (57.4%) and specificity (78.4%), calculated from 2 independent assessments, indicated a lower likelihood of identifying high-risk domain-level judgments while reliably detecting low-risk judgments. However, this pattern was not reflected in the overall trial-level assessment. When domain-level judgments were integrated into an overall ROB classification, the model showed an asymmetric pattern in the opposite direction, tending to classify trials as high risk rather than overlook truly high-risk trials. Specifically, across the 2 assessments, the overall judgments resulted in 9 FPs and only 1 FN. This tendency may be advantageous in screening applications by increasing the likelihood that studies with potential bias will be prioritized for subsequent expert review.

In evaluating performance across trial- and domain-specific contexts, we observed substantial variation in accuracy across medical disciplines, suggesting that the model’s alignment with expert judgments may depend on the characteristics of the underlying literature. This variability may partly reflect known challenges in ROB assessment, such as complex study structures, nonintuitive reporting terminology, and inconsistencies even among experienced reviewers, as noted in previous research [53,54]. Our findings provide a preliminary overview of model performance across medical disciplines. While high accuracy was observed in structured, objective end points such as bone health, these results should be interpreted with caution due to the limited number of trials in these subgroups. Accuracy diminished to approximately 60% in disciplines such as psychology [55] and cardiology [56], suggesting that while the model excels at processing structured data, its reliability diminishes when critical details such as cointerventions or adherence are implicit or conveyed through nuanced narratives. Future implementation should prioritize automated screening for trials with objective outcomes while maintaining intensive expert oversight for behavioral research.

At the domain-specific level, the model demonstrated the highest accuracy in domain 4 (measurement of the outcome), which presented the fewest discrepancies. Meanwhile, domains with well-defined criteria and clearly stated descriptions, such as domain 1 (randomization process), exhibited the highest κ value (0.86) despite a relatively higher frequency of judgment discrepancies compared with the reference standard. In contrast, domain 2 (deviations from intended interventions) exhibited the lowest consistency, with a κ value of 0.39 and the lowest observed agreement. This discrepancy likely reflects that the fixed structure of the prompt struggles with the inherent complexity of this domain. Following the ROB 2, our prompts required the LLM to initially categorize the trial as evaluating either an intention-to-treat or per-protocol effect. When trial reports use obscure or nonstandard terminology without further elaboration, the model’s initial classification is prone to error, resulting in cascading inconsistencies in the subsequent assessment. This aligns with empirical evidence [57] suggesting that human raters also demonstrate the lowest interrater reliability in domain 2, often due to the nuanced interpretive requirements of intention-to-treat and per-protocol effects. These issues present challenges not only for LLMs but also for human reviewers, contributing to discrepancies in judgment and highlighting the need for more precise information extraction, robust reasoning, and contextual understanding in automated ROB assessments.

A total of 74 discrepancies were observed between LLM assessments and the reference standard, with 55 (74.3%) related to differences in final risk judgments rather than data extraction errors. Most judgment-related discrepancies occurred in domains 1 and 5. Analysis showed that the explanations generated by LLMs often highlighted the presence or absence of specific keywords (eg, “concealment”) rather than synthesizing indirect cues or context. For instance, in domain 1, the absence of explicit terms such as “allocation concealment” in the model’s reasons was associated with a “some concerns” rating, whereas human reviewers inferred low risk based on indirect phrases such as “centralized randomization.” A similar discrepancy was observed in domain 5, where vague reporting often led to inconsistent bias judgments. This suggests that the textual outputs observed in the model’s generated justifications remain highly sensitive to surface-level terminology.

These discrepancies highlight a fundamental distinction between human and LLM-based ROB assessments: humans integrate contextual understanding and inferential logic, whereas LLMs’ output may appear influenced by surface features. Nevertheless, the model’s strong internal consistency and performance in well-structured domains suggest its potential as a tool in systematic review workflows.

In addition, our study used GPT-o3-mini-high, an LLM optimized for reasoning and instruction-following capabilities that was explicitly selected for its ability to perform inference tasks to conduct a rule-guided assessment task [7]. Compared to the models used in the previous study [5] (ChatGPT and Claude), which were based on earlier-generation architectures, the GPT-o3-mini model has been shown to generate more accurate and clearer answers by using deeper internal reasoning processes. The GPT-o3-mini model has demonstrated a 39% reduction in major errors on complex questions and was preferred over GPT-o1-mini in 56% of expert tester comparisons [7]. Furthermore, its performance on high-level scientific reasoning benchmarks such as GPQA Diamond (79.7%) significantly outperforms contemporary nonreasoning models such as Claude 3.5 Sonnet (65.0%) [58]. This demonstrates its superior capacity for tasks requiring nuanced judgment, such as ROB evaluation.

Finally, the methodological tools differed between studies. Lai et al [5] assessed bias using a modified version of the original Cochrane ROB tool developed by the CLARITY group [59]. In contrast, our study applied the updated version of the tool, which is currently recommended by the Cochrane Collaboration for evaluating randomized trials [3]. Despite its conceptual improvements, prior research has demonstrated that the ROB 2 exhibits relatively poor interrater reliability [54,60]. It involves more steps and requires more logical reasoning to apply consistently, thereby presenting inherent challenges for consistent evaluation. This complexity makes it particularly suitable for testing LLMs with higher reasoning capacity.

### Limitations

This study has several limitations that should be noted. First, while Cochrane systematic reviews represent a high standard of methodological rigor [12,13], their assessments are not definitive or immune to error. Our comparisons reflect alignment with expert assessments as a reference standard rather than assuming those assessments to be correct. Moreover, the ROB 2 is known to have relatively poor interrater reliability and produce inconsistent judgments even among human raters [53,54,60]. This inherent variability likely contributed to the discrepancies between the LLM-generated and expert assessments. Second, we evaluated a single LLM under fixed prompting conditions without exploring performance across different models, prompt styles, or fine-tuning approaches. Although we iteratively refined prompts using a small pilot set of RCTs based on a previous study [5] and achieved consistent alignment with experts, the approximately 75% accuracy observed in the validation dataset suggests potential limitations in generalizing prompts optimized on a small pilot test to a broader validation dataset. Third, our dataset, limited to 29 RCTs from 11 Cochrane systematic reviews, may not fully represent the diversity across all medical disciplines. Fourth, a limitation involves the potential for pretraining data contamination. Given the nature of LLMs, it is difficult to definitively distinguish between the retrieval of memorized patterns and de novo reasoning. Although mitigation efforts were made, the possibility of prior exposure to public medical datasets remains a fundamental constraint when evaluating LLM outputs. Finally, this study using the ChatGPT web interface introduced certain methodological fragility. As the precise mechanism of PDF parsing within the interface remains uncertain, the risk of data omission or formatting errors cannot be entirely ruled out, highlighting the need for future validation studies to use version-controlled APIs.

### Conclusions

In this exploratory feasibility study using one of the most advanced LLMs available, we found that the accuracy of ROB assessments for randomized trials was largely comparable to that of Cochrane review authors when using the ROB 2. These preliminary findings suggest the potential of LLMs in methodological evaluations, although domains with less structured reporting demand careful consideration. With further development, LLMs could significantly enhance the efficiency and capacity for large-scale application of systematic review processes in biomedical research.

## Supplementary material

10.2196/84915 Multimedia Appendix 1 Prompt for the large language model to assess risk of bias (ROB) in randomized controlled trials using version 2 of the Cochrane ROB tool for randomized trials.

10.2196/84915 Multimedia Appendix 2 Response from ChatGPT.

10.2196/84915 Multimedia Appendix 3 Supplementary tables for reference standard, interpretation criteria, and assessment results.

## Abbreviations

FN: false negative
FP: false positive
GRADE: Grading of Recommendations Assessment, Development, and Evaluation
LLM: large language model
PABA: prevalence-adjusted, bias-adjusted
RCT: randomized controlled trial
RD: relative difference
ROB: risk of bias
ROB 2: version 2 of the Cochrane risk-of-bias tool for randomized trials
TN: true negative
TP: true positive
