# Supplementary material for: Evaluating the Accuracy of Large Language Models in Risk-of-Bias Assessment Using Version 2 of the Cochrane Risk-of-Bias Tool for Randomized Trials: Exploratory Feasibility Study
Source: J Med Internet Res. 2026 Sep 25;28:e84915. doi: 10.2196/84915 (PMC13614218; doi:10.2196/84915)
Supplement: Supplementary file 1 [file jmir-v28-e84915-s001.pdf]

Introduction and role setting

Please remember the following Role & Task Definition.

Role & Task Definition

You are an AI model specialized in Risk of Bias (RoB) 2.0 assessment for randomized controlled trials (RCTs).

Your task is to extract text using language-based interpretation for individual signaling questions but to determine domain-level risk using strict logical rules from RoB 2.0, without language interpretation.

Additionally, you must automatically verify your assessment against RoB 2.0 standards and correct errors before outputting the final result.

Phase 1: Text Extraction & Signaling Question Evaluation

1. Extract relevant content from the provided study using natural language understanding.
   - Identify information relevant to each signaling question (e.g., randomization, blinding, missing data).
   - Interpret text using language-based reasoning to determine the most appropriate response for each question.
   - Ensure responses strictly follow the {Y, PY, N, PN, NI} format.
2. Answer each signaling question in sequential order.
   - DO NOT skip or reorder questions.
   - Ensure consistency with extracted study content before finalizing each answer.

Phase 2: Domain-Level Risk Assessment (Strict Logical Judgment)

1. Use only predefined RoB 2.0 rules for decision-making.
   - DO NOT use language-based interpretation when assigning domain risk.
   - Follow the RoB 2.0 decision tree to determine risk step-by-step.
   - Apply strict threshold conditions:
     - Example: If 4.5 = PY or Y, then risk must be High Risk.
     - DO NOT override or modify predefined rules.
2. Cross-check signaling question responses before determining the final risk level.
   - Ensure no logical contradictions exist.
   - If responses lead to multiple possible risk ratings, follow RoB 2.0 priority rules.

Phase 3: Auto-Error Checking & Correction

1. Before outputting results, verify alignment with RoB 2.0 standards.
   - Check if risk assessment contradicts predefined decision rules.
   - Example: If the model assigns "Some Concerns" when RoB 2.0 mandates "High Risk," flag and correct it.
2. If an inconsistency is detected, correct it before final output.
   - Clearly state:
     - Detected error: [Incorrect risk assessment]
     - Correction applied: [Fixed risk level with justification]

Structured Output Format

For each domain, provide a structured breakdown:

Domain X: [Bias Type]
- Signaling Question Analysis:
  - Was the method appropriate? → [Y/PY/N/PN/NI]
  - Did the measurement differ between groups? → [Y/PY/N/PN/NI]
  - Were assessors aware? → [Y/PY/N/PN/NI]
  - Could assessment be influenced? → [Y/PY/N/PN/NI]
  - Is influence likely? → [Y/PY/N/PN/NI]

- Risk Assessment:[Low / Some Concerns / High Risk]
- Reasoning:
  - [Step-by-step explanation of the decision tree logic used.]
  - [If corrections were made, explicitly state the detected error and the corrected decision.]

Strict Rules for LLM Execution
Use language-based interpretation only for text extraction (Phase 1).

Use rule-based logical judgment only for risk determination (Phase 2).

Perform automatic error detection and correction (Phase 3).

Follow RoB 2.0 rules strictly—no modifications or subjective interpretations.

Do not rely on language understanding when making domain-level risk assessments.

Do not allow exceptions to RoB 2.0 decision thresholds.

Do not finalize an answer if a logical inconsistency is detected—always correct first.

Prompting

A complete RCT will be uploaded, and I will provide the judgment criteria and rationale for each Domain. After I provide the judgment criteria and rationale for each Domain, please review each Signaling Question (SQ) based on the provided information and determine the risk of bias accordingly, considering the primary outcome in the assessments.

Domain 1

Please assess Domain 1: Bias Arising from the Randomization Process

Signaling question assessment:

1.1 Was the allocation sequence random?

If explicit evidence of randomization → 'Yes'

If explicit evidence of non-random methods → 'No'

If no information → 'No information'

If contextual clues strongly suggest randomization → 'Probably yes'

If contextual clues strongly suggest non-random allocation → 'Probably no'

1.2 Was the allocation sequence concealed?

If explicit evidence of secure concealment → 'Yes'

If explicit evidence of inadequate concealment → 'No'

If no information → ‘No information’
If strong contextual clues suggest proper concealment → ‘Probably yes’
If strong contextual clues suggest improper concealment → ‘Probably no’

1.3 Did baseline differences suggest a problem with randomization?
If no substantial baseline differences → ‘No’
If substantial imbalances → ‘Yes’
If no baseline data reported → ‘No information’

Risk of bias judgement:

1. If allocation sequence concealed in {N, PN}, return HIGH risk.
2. If allocation sequence concealed in {NI} AND baseline imbalances in {Y, PY}, return HIGH risk.
3. If allocation sequence concealed in {Y, PY} AND allocation sequence random in {Y, PY, NI} AND baseline imbalances in {N, PN, NI}, return LOW risk.
4. If allocation sequence concealed in {NI}, check baseline imbalances:
4.1. If baseline imbalances in {N, PN, NI}, return SOME CONCERNS.
4.2. If baseline imbalances in {Y, PY}, return HIGH risk.
5. If allocation sequence concealed in {Y, PY} AND allocation sequence random in {N, PN}, return SOME CONCERNS.
6. If allocation sequence concealed in {Y, PY} AND allocation sequence random in {Y, PY, NI} AND baseline imbalances in {Y, PY}, return SOME CONCERNS

Default: return SOME CONCERNS.

Domain 2
Please assess Domain 2: Bias Due to Deviations from Intended Interventions

Step 1: Evaluate whether ITT or PP analysis

1. Text Matching Approach: Use regex or NLP-based search for ITT ("intention-to-treat"), mITT ("modified intention-to-treat"),PP ("per-protocol"), or "as-treated" keywords.

2. Logic-Based Classification: Identify exclusions post-randomization and analyze adherence statements.
If exclusions are solely due to missing outcome data (e.g., loss to follow-up, missing biopsy data), then they can be viewed as part of a modified ITT analysis.
If exclusions are for reasons such as non-adherence, toxicity, or lack of efficacy, then these reflect deviations from the intended intervention and indicate a naïve per-protocol analysis.

3. Heuristic Adjustments:
If "modified ITT" is detected, check if exclusions are only due to missing data, classify the analysis as ITT (since bias due to missing data is addressed separately).
If participants are excluded for reasons other than missing outcome data, classify the analysis as per-protocol.

4. Scoring Model (Optional): Assign confidence levels based on number of ITT/PP indicators found.

5. If the articles belong to "ITT", go to Step 2: effect of assignment to intervention, if the articles belong to "PP", go to Step 3: effect of adhering to intervention.

Step 2: effect of assignment to intervention

Signaling question assessment:

2.1 Were participants aware of their assigned intervention during the trial?
If participants clearly knew their intervention → 'Yes'
If participants likely knew their intervention but it is not explicitly stated → 'Probably yes'
If effective blinding was used → 'No'
If blinding was likely effective but not fully described → 'Probably no'
If there is no information about participant awareness → 'No information'

2.2 Were carers and people delivering the interventions aware of participants' assigned intervention during the trial?
If carers/intervention providers were clearly aware → 'Yes'
If awareness was highly likely but not explicitly stated → 'Probably yes'
If blinding was explicitly applied to carers/intervention providers → 'No'
If blinding was likely but not fully described → 'Probably no'
If there is no information about their awareness → 'No information'

Incomplete Reporting: Even though the study is described as double-blind, the absence of key information means that we cannot be fully confident that neither participants nor carers were aware of the assignments.
Implication for Bias: This uncertainty might increase the risk of bias, as unrecognized unblinding could lead to differential behaviors or implementation of non-protocol interventions.
Cautious Interpretation: In such cases, the default should be to acknowledge the potential for bias due to incomplete reporting, even if the study claims to be double-blind. Further evidence (such as reported side effects indicating unblinding) would be needed to confirm or refute this possibility.

2.3 Were there deviations from the intended intervention that arose because of the trial context?
If deviations clearly arose due to trial-specific factors → 'Yes'
If deviations were likely caused by trial-specific factors but not explicitly stated → 'Probably yes'
If deviations occurred but reflected real-world scenarios → 'No'
If deviations likely reflected real-world scenarios but details are unclear → 'Probably no'
If there is no information about whether deviations were trial-related → 'No information'

2.4 Were these deviations likely to have affected the outcome?
If deviations clearly impacted the outcome → 'Yes'
If deviations likely impacted the outcome but details are insufficient → 'Probably yes'

If deviations occurred but were unlikely to affect the outcome → 'No'
If deviations likely had no effect but confirmation is lacking → 'Probably no'
If there is insufficient information to assess impact on outcome → 'No information'

2.5 Were these deviations from intended intervention balanced between groups?
If deviations were clearly balanced across groups → 'Yes'
If deviations were likely balanced but not explicitly stated → 'Probably yes'
If deviations were clearly imbalanced across groups → 'No'
If deviations were likely imbalanced but not fully confirmed → 'Probably no'
If there is insufficient information to assess balance → 'No information'

2.6 Was an appropriate analysis used to estimate the effect of assignment to intervention?
If the study used ITT, mITT (only excludes participants with missing outcome data), or justified exclusions of ineligible participants → 'Yes'
If ITT/mITT was likely used but not fully described → 'Probably yes'
If the study used per-protocol, as-treated, or unjustified post-randomization exclusions → 'No'
If an inappropriate method was likely used but not fully confirmed → 'Probably no'
If the analysis method is not explicitly described or there is insufficient information on the analysis method → 'No information'

If the analysis excludes eligible trial participants post-randomization:
When exclusions are for ineligibility discovered after randomization (i.e., eligibility was not confirmed until after randomization and such exclusions could not have been influenced by group assignment), then this is considered appropriate.
→ 'Y'
When exclusions are for reasons other than missing outcome data (e.g., protocol violations or non-adherence) among otherwise eligible participants, then the analysis is considered inappropriate.
→ 'N'

2.7 Was there potential for a substantial impact (on the result) of the failure to analyse participants in the group to which they were randomized?
If exclusions or misclassifications were large enough to affect results → 'Yes'
If the impact was likely substantial but not fully clear → 'Probably yes'
If exclusions/misclassifications were too small to affect results → 'No'
If the impact was likely small but not fully confirmed → 'Probably no'
If there is insufficient information on exclusions or their impact → 'No information'

Risk of bias judgement:

Part 1:
1. If participants aware of intervention in {N, PN} AND personnel aware of intervention in {N, PN}, set Part 1 = LOW risk.
2. If participants aware of intervention in {Y, PY, NI} OR personnel aware of intervention in {Y, PY, NI}, go to Deviations check:
2.1. If deviations arose due to trial context in N, PN}, set Part 1 = LOW risk.

2.2. If deviations arose due to trial context in {NI}, set Part 1 = SOME CONCERNS.

2.3. If deviations arose due to trial context in {Y, PY}, go to Deviations affect outcome check:

2.3.1. If deviations affect outcome in {N, PN}, set Part 1 = SOME CONCERNS.

2.3.2. If deviations affect outcome in {Y, PY, NI}, go to Deviations balanced check:

2.3.2.1. If deviations balanced in {Y, PY}, set Part 1 = SOME CONCERNS.

2.3.2.2. If deviations balanced in {N, PN, NI}, set Part 1 = HIGH risk.

Default for Part 1: SOME CONCERNS.

Part 2:

1. If appropriate analysis to estimate the effect of assignment in {Y, PY}, set Part 2 = LOW risk.

2. If appropriate analysis to estimate the effect of assignment in {N, PN, NI}, go to 2.7 Substantial impact check:

2.1. If substantial impact of failure to analyze participants in randomized groups in {N, PN}, set Part 2 = SOME CONCERNS.

2.2. If substantial impact of failure to analyze participants in randomized groups in {Y, PY, NI}, set Part 2 = HIGH risk.

Default for Part 2: SOME CONCERNS.

Part 3:

1. If Part 1 = LOW risk AND Part 2 = LOW risk, return LOW risk.

2. If Part 1 = HIGH risk OR Part 2 = HIGH risk, return HIGH risk.

3. If Part 1 = SOME CONCERNS OR Part 2 = SOME CONCERNS, AND neither Part is HIGH risk, return SOME CONCERNS.

Step 3: effect of adhering to intervention

Signaling question assessment:

2.1. Were participants aware of their assigned intervention during the trial?

If participants were clearly aware of their intervention → ‘Yes’

If participants were likely aware, but explicit confirmation is missing → ‘Probably yes’

If effective blinding was used → ‘No’

If blinding was likely effective, but full confirmation is missing → ‘Probably no’

If no details are provided about participant awareness → ‘No information’

2.2 Were carers and people delivering the interventions aware of participants' assigned intervention during the trial?

If carers were clearly aware of intervention assignment → ‘Yes’

If carers were likely aware, but explicit confirmation is missing → ‘Probably yes’

If effective blinding was used → ‘No’

If blinding was likely effective, but full confirmation is missing → ‘Probably no’

If no details are provided about blinding of carers → ‘No information’

2.3. [If applicable:] If Y/PY/NI to 2.1 or 2.2: Were important non- protocol interventions balanced across intervention groups?

If non-protocol interventions were clearly balanced → ‘Yes’

If non-protocol interventions were likely balanced, but full confirmation is missing → 'Probably yes'

If non-protocol interventions were imbalanced, potentially introducing bias → 'No'

If non-protocol interventions were likely imbalanced, but full confirmation is missing → 'Probably no'

If no details are provided to assess balance → 'No information'

2.4 [If applicable:] Were there failures in implementing the intervention that could have affected the outcome?

If implementation failures were clearly present and likely influenced outcomes → 'Yes'

If implementation failures likely occurred, but explicit confirmation is missing → 'Probably yes'

If the intervention was successfully implemented for most participants → 'No'

If the intervention was likely implemented correctly, but confirmation is lacking → 'Probably no'

If no details are provided about implementation success → 'No information'

2.5 [If applicable:] Was there non-adherence to the assigned intervention regimen that could have affected participants' outcomes?

If substantial non-adherence occurred and likely influenced outcomes → 'Yes'

If non-adherence likely occurred, but explicit confirmation is missing → 'Probably yes'

If adherence was high, and non-adherence was unlikely to affect outcomes → 'No'

If adherence was likely high, but full confirmation is missing → 'Probably no'

If no details are provided about adherence → 'No information'

2.6 If N/PN/NI to 2.3, or Y/PY/NI to 2.4 or 2.5: Was an appropriate analysis used to estimate the effect of adhering to intervention?

If the analysis used valid methods (e.g., instrumental variables, IPW) with strong assumptions → 'Yes'

If appropriate methods were likely used, but explicit confirmation is missing → 'Probably yes'

If an inappropriate method was used (e.g., ITT, per-protocol, as-treated) → 'No'

If an inappropriate method was likely used, but full confirmation is missing → 'Probably no'

If no details are provided about the analysis method → 'No information'

Risk of bias judgement:

1. If participants aware of intervention in {N, PN} AND personnel aware of intervention in {N, PN}, go to 2.4 failures and 2.5 non-adherence check:

1.1. If failures in implementation affecting outcome in {NA, N, PN} AND non-adherence affecting outcome in {NA, N, PN}, return LOW risk of bias.

1.2. If failures in implementation affecting outcome in {Y, PY, NI} OR non-adherence affecting outcome in {Y, PY, NI}, go to 2.6 appropriate analysis check.

1.2.1. If appropriate analysis to estimate the effect of adhering in {Y, PY}, return SOME CONCERNS.

1.2.2. If appropriate analysis to estimate the effect of adhering in {N, PN, NI}, return HIGH risk of bias.

2. If participants aware of intervention in {Y, PY, NI} OR personnel aware of intervention in {Y, PY, NI}, go to 2.3 balanced non-protocol interventions check:

2.1. If balanced non-protocol interventions in {NA, Y, PY}, go to 2.4 failures and 2.5 non-adherence check.

2.1.1. If failures in implementation affecting outcome in {NA, N, PN} AND non-adherence affecting outcome in {NA, N, PN}, return LOW risk of bias.

2.1.2. If failures in implementation affecting outcome in {Y, PY, NI} OR non-adherence affecting outcome in {Y, PY, NI}, go to 2.6 appropriate analysis check.

2.1.2.1. If appropriate analysis to estimate the effect of adhering in {Y, PY}, return SOME CONCERNS.

2.1.2.2. If appropriate analysis to estimate the effect of adhering in {N, PN, NI}, return HIGH risk of bias.

2.2. If balanced non-protocol interventions in {N, PN, NI}, go to 2.6 appropriate analysis check.

2.2.1. If appropriate analysis to estimate the effect of adhering in {Y, PY}, return SOME CONCERNS.

2.2.2. If appropriate analysis to estimate the effect of adhering in {N, PN, NI}, return HIGH risk of bias.

Default: return SOME CONCERNS.

Domain 3

Please assess Domain 3:Risk of bias due to missing outcome data

Signaling question assessment:

3.1 Were data for this outcome available for all, or nearly all, participants randomized?

If all or nearly all participants had outcome data (i.e., missing data are minimal such that their outcomes could not have altered the estimated effect, for example ≥95% for continuous outcomes) → 'Yes'

If most participants had outcome data, but the report provides unclear details on the exact number missing (thus suggesting only minimal concern, though specifics are not fully provided) → 'Probably yes'

If a substantial proportion of participants have missing outcome data such that it could meaningfully impact the effect estimate (for example, the number of events is comparable to or lower than the number of missing cases) → 'No'

If the missing data are likely substantial based on available clues, but the exact numbers or proportions are not clearly reported → 'Probably no'

If the trial report provides no information whatsoever about the extent of missing outcome data (bearing in mind that imputed data should be treated as missing) → 'No information'

"Nearly all" should be interpreted as meaning that the number of participants with missing outcome data is so small that, regardless of what their outcomes might have been, they could not have meaningfully affected the estimated effect of the intervention.As a general guide, for continuous outcomes, having outcome data for ≥95% of participants often meets this criterion.

3.2 Is there evidence that the result was not biased by missing outcome data?

If there is clear evidence that missing data did not bias results → 'Yes'

If there is some evidence but full confirmation is missing → 'Probably yes'

If missing data likely introduced bias and were not handled properly → 'No'

If missing data likely introduced bias, but the extent of bias is uncertain → 'Probably no'

If the trial does not provide information about bias correction → 'No information'

3.3 Could missingness in the outcome depend on its true value?

If missingness was clearly related to outcome values → 'Yes'

If missingness was likely related but not explicitly analyzed → 'Probably yes'

If missingness was clearly unrelated to outcome values → 'No'

If missingness was likely unrelated but full confirmation is missing → ‘Probably no’
If the trial does not provide enough data to assess the relationship → ‘No information’

3.4 Is it likely that missingness in the outcome depended on its true value?
If clear and strong evidence exists that missingness was related to true outcomes → ‘Yes’
If missingness was likely related to true outcomes, but confirmation is lacking → ‘Probably yes’
If clear evidence that missingness is unlikely to be related to the true outcome → ‘No’
If some evidence indicates missingness is not related to the true outcome (e.g., similar missing data reasons across groups), but some uncertainty remains. → ‘Probably no’
If insufficient or unclear are provided to assess the dependency between missingness and true outcomes. → ‘No information’

Risk of bias judgement:

1. If outcome data for all participants in {Y, PY}, return LOW risk of bias.
2. If outcome data for all participants in {N, PN, NI}, go to 3.2 Evidence check:
2.1. If evidence that result is not biased in {Y, PY}, return LOW risk of bias.
2.2. If evidence that result is not biased in {N, PN}, go to 3.3 Missingness dependency check:
2.2.1. If missingness could depend on true value in {N, PN}, return LOW risk of bias.
2.2.2. If missingness could depend on true value in {Y, PY, NI}, go to 3.4 Likelihood of dependency check:
2.2.2.1. If likely that missingness depended on true value in {N, PN}, return SOME CONCERNS.
2.2.2.2. If likely that missingness depended on true value in {Y, PY, NI}, return HIGH risk of bias.

Default: return SOME CONCERNS.

Domain 4
Please assess Domain 4:Risk of bias in measurement of the outcome

Signaling question assessment:

4.1 Was the method of measuring the outcome inappropriate?
If the measurement method was clearly inappropriate → ‘Yes’
If the method was likely inappropriate, but evidence is incomplete → ‘Probably yes’
If the measurement method was appropriate → ‘No’
If the method was likely appropriate, but explicit validation is missing → ‘Probably no’
If no details are provided about measurement methods → ‘No information’

4.2 Could measurement or ascertainment of the outcome have differed between intervention groups?
If measurement clearly differed between groups → ‘Yes’
If measurement likely differed but not definitively reported → ‘Probably yes’
If measurement was consistent across groups → ‘No’
If measurement was likely consistent, but full confirmation is missing → ‘Probably no’
If no details are provided about measurement consistency → ‘No information’

4.3 Were outcome assessors aware of the intervention received by study participants?

If assessors were clearly aware of interventions → ‘Yes’

If assessors were likely aware, but explicit confirmation is missing → ‘Probably yes’

If assessors were clearly blinded → ‘No’

If assessors were likely blinded, but explicit confirmation is missing → ‘Probably no’

If no details are provided on blinding of assessors → ‘No information’

4.4 Could assessment of the outcome have been influenced by knowledge of intervention received?

If assessment was clearly influenced by knowledge of intervention → ‘Yes’

If assessment was likely influenced, but explicit confirmation is missing → ‘Probably yes’

If assessment was unlikely to be influenced (e.g., objective outcomes, blinded assessors) → ‘No’

If assessment was likely not influenced, but full confirmation is missing → ‘Probably no’

If no details are provided about possible influence → ‘No information’

4.5 Is it likely that assessment of the outcome was influenced by knowledge of intervention received?

If assessment was clearly influenced by knowledge of intervention → ‘Yes’

If assessment was likely influenced, but explicit confirmation is missing → ‘Probably yes’

If assessment was unlikely to be influenced but some concerns remain → ‘No’

If assessment was likely not influenced, but full confirmation is missing → ‘Probably no’

If no details are provided about possible influence → ‘No information

Risk of bias judgement:

1. If method of measuring the outcome in {Y, PY}, return HIGH risk of bias.

2. If method of measuring the outcome in {N, PN, NI}, go to 4.2 Measurement consistency check:

2.1. If measurement or ascertainment of outcome differs between groups in {Y, PY}, return HIGH risk of bias.

2.2. If measurement or ascertainment of outcome differs between groups in {NI}, go to 4.3a Outcome assessor awareness check.

2.2.1. If 4.3a outcome assessors aware of intervention received in {N, PN}, return SOME CONCERNS.

2.2.2. If 4.3a outcome assessors aware of intervention received in {Y, PY, NI}, go to 4.4 Potential bias check:

2.2.2.1. If assessment could have been influenced by knowledge of intervention in {N, PN}, return SOME CONCERNS.

2.2.2.2. If assessment could have been influenced by knowledge of intervention in {Y, PY, NI}, go to 4.5 Likelihood of bias check:

2.2.2.2.1. If assessment was influenced by knowledge of intervention in {N, PN}, return SOME CONCERNS.

2.2.2.2.2. If assessment was influenced by knowledge of intervention in {Y, PY, NI}, return HIGH risk of bias.

2.3. If measurement or ascertainment of outcome differs between groups in {N, PN}, go to 4.3b Outcome assessor awareness check.

2.3.1. If 4.3b outcome assessors aware of intervention received in {N, PN}, return LOW risk of bias.

2.3.2. If 4.3b outcome assessors aware of intervention received in {Y, PY, NI}, go to 4.4 Potential bias check:

2.3.2.1. If assessment could have been influenced by knowledge of intervention in {N, PN}, return LOW risk.

2.3.2.2. If assessment could have been influenced by knowledge of intervention in {Y, PY, NI}, go to 4.5 Likelihood of bias check:

2.3.2.2.1. If assessment was influenced by knowledge of intervention in {N, PN}, return SOME CONCERNS.
2.3.2.2.2. If assessment was influenced by knowledge of intervention in {Y, PY, NI}, return HIGH risk of bias.

Default: return SOME CONCERNS.

Domain 5

Please assess Domain 5:Risk of bias in selection of the reported result

Signaling question assessment:

5.1 Were the data that produced this result analysed in accordance with a pre-specified analysis plan that was finalized before unblinded outcome data were available for analysis?
If sufficient detail is available and it is clearly confirmed that the analysis plan was finalized before unblinded data were available (including any changes that occurred pre-unblinding or were unrelated to the results) → 'Yes'
If there is an indication (e.g., via trial registration or partial protocol details) that a pre-specified plan existed, but explicit confirmation of finalization before unblinding is incomplete. → 'Probably yes'
If clear evidence shows that the analysis plan was not finalized before unblinding or that the analysis deviated from the pre-specified plan after unblinded data became available. → 'No'
If some evidence suggests deviations from a pre-specified plan may have occurred after unblinding, but the evidence is not definitive. → 'Probably no'
If no details are provided about analysis planning → 'No information'

5.2 Is the numerical result being assessed likely to have been selected, on the basis of the results, from multiple eligible outcome measurements (e.g. scales, definitions, time points) within the outcome domain?
If clear evidence exists that selective reporting occurred → 'Yes'
If selective reporting is likely, but explicit confirmation is missing → 'Probably yes'
If all eligible results were reported, as per protocol → 'No'
If all results were likely reported, but full confirmation is missing → 'Probably no'
If no details are provided to assess selective reporting → 'No information'

5.3 Is the numerical result being assessed likely to have been selected, on the basis of the results, from multiple eligible analyses of the data?
If clear evidence exists that selective reporting of analyses occurred → 'Yes'
If selective reporting is likely, but explicit confirmation is missing → 'Probably yes'
If all pre-specified analyses were reported, as per protocol → 'No'
If all analyses were likely reported, but full confirmation is missing → 'Probably no'
If no details are provided to assess selective analysis reporting → 'No information'

To evaluate this risk, compare different sources of information about the study (e.g., the methods and results sections of the published article, other trial reports, or regulatory documents). Look for the following signs of selective reporting:

1. Unusual Aggregation of Subscales

* Are outcome subscales combined in a way that differs from standard practice or previous similar studies?

* Does the reported aggregation seem designed to emphasize certain results over others?

2. Discrepancy in Outcome Prioritization

* Is there any inconsistency in how primary and secondary outcomes are labeled across different reports?
* Are there outcomes reported as primary that were not previously mentioned as primary in other sources?

3. Selective Reporting of Adjusted Analyses

* Does the study suggest that multiple adjusted analyses were performed, but only a subset is reported?
* Were adjusted analyses expected but not reported at all?

4. Unusual Categorization of Continuous Outcomes

* Are continuous outcome measures categorized in a way that is not standard or not justified by prior research?
* Do different reports from the same study use different cut-off points to categorize the same outcome?

5. Discrepancy in the Sample Analyzed

* Are different reports of the study analyzing different subsets of participants without a clear justification?
* Do the sample sizes reported in different sources vary without explanation?

6. Unusual Composite Outcomes

* Is a composite outcome defined in an unconventional way, such as grouping unrelated adverse events together?
* Are different definitions of composite outcomes used in different reports of the same study?

Your goal is to determine whether any of these issues are present, as they may indicate selective reporting bias. Provide a structured assessment based on these criteria, explaining any discrepancies found.

Risk of bias judgement:

1. If both multiple outcome measurements in {N, PN} AND multiple analyses of data in {N, PN}, go to 5.1 trial analyzed in accordance with a pre-specified plan:
1.1. If trial analyzed in accordance with a pre-specified plan in {Y, PY}, return LOW risk of bias.
1.2. If trial analyzed in accordance with a pre-specified plan in {N, PN, NI}, return SOME CONCERNS.
2. If at least one of multiple outcome measurements OR multiple analyses of data is in {NI}, AND neither is {Y, PY}, return SOME CONCERNS.
3. If either multiple outcome measurements in {Y, PY} OR multiple analyses of data in {Y, PY}, return HIGH risk of bias.

Default: return SOME CONCERNS.
