# Supplementary material for: Evaluating the Accuracy of Large Language Models in Risk-of-Bias Assessment Using Version 2 of the Cochrane Risk-of-Bias Tool for Randomized Trials: Exploratory Feasibility Study
Source: J Med Internet Res. 2026 Sep 25;28:e84915. doi: 10.2196/84915 (PMC13614218; doi:10.2196/84915)
Supplement: Supplementary file 2 [file jmir-v28-e84915-s002.pdf]

ID: Marenzi 2014

First Response

Domain 1: Bias Arising from the Randomization Process

- Signaling Question Analysis:
  - 1.1 Was the allocation sequence random? → Yes
    The study explicitly states that patients were randomized using computer-generated random numbers.
  - 1.2 Was the allocation sequence concealed? → Yes
    Allocation was managed via sealed envelopes, providing explicit evidence of secure concealment.
  - 1.3 Did baseline differences suggest a problem with randomization? → No
    Baseline characteristics were similar between the groups, indicating no substantial imbalances.
- Risk Assessment: LOW Risk
- Reasoning:
  - The allocation sequence was explicitly randomized (Yes) and concealed (Yes).
  - No substantial baseline imbalances were observed (No).
  - According to the RoB 2.0 decision rules, when the allocation sequence is concealed (Yes), the randomization method is clearly reported (Yes), and baseline imbalances are absent (No), the domain is judged as Low Risk.
  - No inconsistencies or errors were detected in applying these rules.

Domain 2: Bias Due to Deviations from Intended Interventions

- Step 1: Evaluation of ITT vs PP Analysis:
  - The study explicitly states that "the primary analyses were analyzed according to the intention-to-treat principle."
  - No post-randomization exclusions for reasons such as non-adherence, toxicity, or lack of efficacy were reported; any exclusions were related solely to missing outcome data if applicable.
  - Conclusion: The analysis is classified as ITT, which is appropriate.
- Step 2: Effect of Assignment to Intervention
  - 2.1 Were participants aware of their assigned intervention during the trial? → Yes
    The study was unblinded, so participants knew their intervention assignment.
  - 2.2 Were carers and people delivering the interventions aware of participants' assigned intervention during the trial? → Yes
    Being an unblinded trial, intervention providers were aware of the assignments.
  - 2.3 Were there deviations from the intended intervention that arose because of the trial context? → No
    The protocol was adhered to as planned, and any modifications (e.g., adjustments in diuretic dosing) were consistent with clinical practice rather than deviations induced by trial context.
  - 2.4 Were these deviations likely to have affected the outcome? → No
    There is no evidence that any deviations (if present) impacted the outcome.
  - 2.5 Were these deviations from intended intervention balanced between groups? → Yes
    No imbalance in deviations is reported between the intervention groups.
  - 2.6 Was an appropriate analysis used to estimate the effect of assignment to intervention? → Yes
    The study used an intention-to-treat analysis, which is considered appropriate.

- 2.7 Was there potential for a substantial impact of the failure to analyze participants in the group to which they were randomized? → No
  Because an ITT approach was used, any impact of post-randomization exclusions is minimal.
- Risk Assessment for Step 2:
  - Part 1 (Awareness and Deviations): Although both participants and personnel were aware (Yes), there were no deviations arising due to the trial context (No); therefore, Part 1 is LOW risk.
  - Part 2 (Analysis Method): An appropriate ITT analysis was applied (Yes) and no substantial impact from exclusions is evident (No), so Part 2 is LOW risk.
  - Combined Outcome: With both parts at LOW risk, the effect of assignment to intervention is judged as LOW risk.

- Final Risk Assessment for Domain 2: LOW Risk
- Reasoning:
  - ITT Analysis: The study's use of an intention-to-treat analysis appropriately addresses potential deviations from the intended intervention.
  - Unblinded Design: Although both participants and carers were aware of their intervention assignments (which might raise concerns in other settings), the absence of any protocol deviations driven by the trial context minimizes bias.
  - Balance and Impact: There is no indication of imbalanced deviations or a significant impact on outcomes from any departures from the protocol.
  - Decision Rules Compliance: According to the predefined RoB 2.0 rules, with participants and personnel aware but no trial-specific deviations affecting the outcome and appropriate ITT analysis used, the risk of bias due to deviations from intended interventions is appropriately classified as LOW Risk. No inconsistencies were detected in applying the rules, and all criteria support a LOW risk judgment for this domain.

Domain 3: Risk of Bias Due to Missing Outcome Data

- Signaling Question Analysis:
  - 3.1 Were data for this outcome available for all, or nearly all, participants randomized? → Yes
    Outcome data for all-cause mortality were available for approximately 96% of participants (54 out of 56), which meets the criterion for "nearly all."
  - 3.2 Is there evidence that the result was not biased by missing outcome data? → Yes
    The small amount of missing data (2 participants) is unlikely to have biased the mortality estimate, especially given the clear reasons provided for missing follow-up.
  - 3.3 Could missingness in the outcome depend on its true value? → No
    The reasons for missing follow-up (chronic dialysis initiation in one control patient and urgent cardiac transplantation in one ultrafiltration patient) do not clearly indicate a relationship with the mortality outcome.
  - 3.4 Is it likely that missingness in the outcome depended on its true value? → No
    There is no strong evidence suggesting that the few missing cases would have had different mortality outcomes compared to those with available data.
- Risk Assessment: LOW Risk
- Reasoning:
  - With outcome data available for nearly all participants (96%), and clear evidence that the minimal missing data did not bias the results, the risk of bias due to missing outcome data is judged as LOW.

- The reasons for the two missing cases do not indicate a dependency on the true mortality outcome.
- According to the RoB 2.0 decision rules, the minimal impact of missing data supports a LOW risk of bias for this domain.

Domain 4: Risk of Bias in Measurement of the Outcome

- Signaling Question Analysis:
  - 4.1 Was the method of measuring the outcome inappropriate? → No
    All-cause mortality is an objective outcome typically measured through official records (e.g., death certificates, hospital records), which is an appropriate method.
  - 4.2 Could measurement or ascertainment of the outcome have differed between intervention groups? → No
    The process for ascertaining death is standardized and applied similarly across both intervention groups.
  - 4.3 Were outcome assessors aware of the intervention received by study participants? → Yes
    Given the open-label design of the trial, outcome assessors were likely aware of the interventions.
  - 4.4 Could assessment of the outcome have been influenced by knowledge of intervention received? → No
    Despite assessors being aware, the objective nature of all-cause mortality minimizes any potential influence on outcome assessment.
  - 4.5 Is it likely that assessment of the outcome was influenced by knowledge of intervention received? → No
    The measurement of death is not subject to interpretation, so knowledge of group assignment is unlikely to have affected the assessment.
- Risk Assessment: LOW Risk
- Reasoning:
  - The method for measuring the outcome (all-cause mortality) is appropriate and objective.
  - There is no evidence that the ascertainment of death differed between groups.
  - Although outcome assessors were aware of the intervention assignments, the objectivity of the outcome ensures that this awareness is unlikely to introduce bias.
  - Following the RoB 2.0 decision rules, these findings support a LOW Risk judgment for bias in the measurement of the outcome.

Domain 5: Risk of Bias in Selection of the Reported Result

- Signaling Question Analysis:
  - 5.1 Were the data that produced this result analysed in accordance with a pre-specified analysis plan finalized before unblinded outcome data were available? → Probably yes
    The trial was registered (ClinicalTrials.gov NCT00360958) and its methods—including the use of an intention-to-treat analysis and predefined endpoints—are consistent with a pre-specified analysis plan. Although the report does not explicitly state that the plan was finalized prior to unblinding, the available details strongly imply that this was the case.
  - 5.2 Is the numerical result being assessed likely to have been selected, on the basis of the results, from multiple eligible outcome measurements within the outcome domain? → No
    All-cause mortality is an objective, unambiguous outcome with only one standard measurement, and there is no evidence of selective outcome measurement or reporting.
  - 5.3 Is the numerical result being assessed likely to have been selected, on the basis of the results, from multiple eligible analyses of the data? → No

The analysis of all-cause mortality was straightforward and reported as part of the pre-specified analysis, with no indication that alternative analyses were conducted and selectively reported.

- Risk Assessment: LOW Risk
- Reasoning:
  - With the responses for 5.2 and 5.3 being "No" (indicating no evidence of multiple eligible outcome measurements or analyses) and 5.1 being "Probably yes" (supporting adherence to a pre-specified analysis plan), the criteria align with a LOW risk of bias.
  - There is no indication of selective reporting that would favor one outcome or analysis over another.
  - Based on the RoB 2.0 decision rules, these findings support a LOW Risk judgment for bias in the selection of the reported result.

ID: Marenzi 2014

Second Response

Domain 1: Bias Arising from the Randomization Process

- Signaling Question Analysis:
  - Was the allocation sequence random? → Y

    Rationale: The study explicitly states that patients were randomized using computer-generated random numbers.
  - Was the allocation sequence concealed? → Y

    Rationale: The randomization assignments were kept in sealed envelopes, and a physician not involved in the study opened the envelope, indicating secure concealment.
  - Did baseline differences suggest a problem with randomization? → N

    Rationale: Baseline characteristics were similar across groups with no substantial imbalances.
- Risk Assessment: Low
- Reasoning:
  - The allocation sequence is explicitly reported as random (Y).
  - The concealment of the allocation sequence is clearly described as secure (Y).
  - There are no substantial baseline imbalances (N).
  - According to the predefined RoB 2.0 decision rules, when allocation concealment is "Y", randomization is "Y", and baseline imbalances are "N", the domain is judged to have Low risk of bias.

Domain 2: Bias Due to Deviations from Intended Interventions

- Step 1: Analysis Type
  - Analysis Approach: The study explicitly states that "the primary analyses were analyzed according to the intention-to-treat principle."

    → Classification: ITT

- Step 2: Effect of Assignment to Intervention

- 2.1 Were participants aware of their assigned intervention?
  Answer: Y
  Rationale: The trial was unblinded; participants knew which intervention they received.
- 2.2 Were carers and people delivering the interventions aware of participants' assigned intervention?
  Answer: Y
  Rationale: The study was unblinded, so personnel were aware of the group assignments.
- 2.3 Did deviations from the intended intervention arise because of the trial context?
  Answer: N
  Rationale: Although the study was unblinded, there is no evidence that deviations were introduced by the trial context; treatment protocols were followed as planned.
- 2.4 Were these deviations likely to have affected the outcome?
  Answer: N
  Rationale: There is no indication that any deviations (if present) impacted the primary outcome (all-cause mortality).
- 2.5 Were these deviations balanced between groups?
  Answer: Y
  Rationale: There is no indication of imbalances in any deviations across the intervention groups.
- 2.6 Was an appropriate analysis used to estimate the effect of assignment to intervention?
  Answer: Y
  Rationale: The study used ITT analysis, which is appropriate for capturing the effect of assignment.
- 2.7 Was there potential for a substantial impact of the failure to analyze participants in the group to which they were randomized?
  Answer: N
  Rationale: The ITT approach minimizes concerns about post-randomization exclusions impacting the result.
- Risk Judgement for Effect of Assignment:
  - Since participants and carers were aware (Y) but no deviations arose from the trial context (2.3 = N) and an appropriate (ITT) analysis was used (2.6 = Y), Part 1 and Part 2 are judged LOW risk.

- Step 3: Effect of Adhering to Intervention
  - 2.1 Were participants aware of their assigned intervention during the trial?
    Answer: Y
    Rationale: As noted, the trial was unblinded.
  - 2.2 Were carers and people delivering the interventions aware of participants' assigned intervention?
    Answer: Y
    Rationale: The trial was unblinded.
  - 2.3 Were important non-protocol (non-study) interventions balanced across groups?
    Answer: PY
    Rationale: Although additional medical therapy was left to the treating cardiologist’s discretion, there is no indication of imbalance between groups.
  - 2.4 Were there failures in implementing the intervention that could have affected the outcome?
    Answer: N

Rationale: The interventions were implemented according to protocol. Although some procedural complications (e.g., minor bleeding, filter clotting) were noted in the ultrafiltration group, these were managed within the protocol and did not lead to significant deviations.

- 2.5 Was there non-adherence to the assigned intervention that could have affected the outcome?
  Answer: N
  Rationale: There is no evidence of non-adherence or systematic deviations from the intervention regimens.
- 2.6 Was an appropriate analysis used to estimate the effect of adhering to intervention?
  Answer: (Not applicable)
  Rationale: Since there were no significant failures or non-adherence issues, no additional adherence-specific analysis was necessary.
- Risk Judgement for Effect of Adhering:
  - With participants and carers aware but with balanced non-protocol interventions (PY), and with no implementation failures or non-adherence (both N), this component is judged LOW risk.

- Overall Domain 2 Risk Assessment:
  - Combined Judgment:
    - Effect of Assignment: LOW risk
    - Effect of Adhering: LOW risk
  - Final Risk Assessment for Domain 2: LOW risk
- Reasoning Summary:
  The study used an ITT analysis, and although the trial was unblinded (participants and personnel aware of the intervention), there is no evidence that deviations from the intended interventions arose from the trial context or that any deviations were imbalanced or affected the outcome. The appropriate analysis method was used, and there were no significant issues of non-adherence or implementation failures. Therefore, based on the structured decision rules, the overall risk of bias due to deviations from intended interventions is LOW risk.

Domain 3: Risk of Bias Due to Missing Outcome Data

- Signaling Question Analysis:
  - 3.1 Were data for this outcome available for all, or nearly all, participants randomized? → Yes
    Rationale: Of the 56 randomized participants, outcome data (all-cause mortality) were available for approximately 54 participants (~96%), meeting the ≥95% threshold.
  - 3.2 Is there evidence that the result was not biased by missing outcome data? → Yes
    Rationale: The few missing data (2 participants) are unlikely to have biased the results given their minimal proportion and the lack of evidence suggesting systematic differences.
  - 3.3 Could missingness in the outcome depend on its true value? → No
    Rationale: The reasons for missing data (one case of chronic dialysis initiation and one of urgent cardiac transplantation) do not provide clear evidence that missingness was related to the true mortality outcome.
  - 3.4 Is it likely that missingness in the outcome depended on its true value? → No
    Rationale: With a very small amount of missing data and no strong indication of dependency on outcome, it is unlikely that missingness depended on the true mortality outcome.

- Risk Assessment:
  Based on the decision rules:
  - Outcome data are available for nearly all participants (Yes), and there is clear evidence that missing data did not bias the results (Yes).
    → Final Risk Assessment for Domain 3: LOW risk
- Reasoning Summary:
  Nearly complete outcome data (96% available) with only two participants missing, combined with no evidence that the missing data were related to the true mortality outcome, support a judgment of LOW risk for bias due to missing outcome data.

Domain 4: Risk of Bias in Measurement of the Outcome

- Signaling Question Analysis:
  - 4.1 Was the method of measuring the outcome inappropriate? → No
    Rationale: All-cause mortality is an objective outcome typically determined from medical records or vital statistics, which is an appropriate measurement method.
  - 4.2 Could measurement or ascertainment of the outcome have differed between intervention groups? → No
    Rationale: There is no indication that the methods used to ascertain mortality differed between the ultrafiltration and control groups.
  - 4.3 Were outcome assessors aware of the intervention received by study participants? → Yes
    Rationale: The study was unblinded regarding intervention assignment, so outcome assessors were likely aware of which treatment participants received.
  - 4.4 Could assessment of the outcome have been influenced by knowledge of intervention received? → No
    Rationale: Despite awareness of treatment allocation, the objective nature of mortality minimizes the potential for bias in outcome assessment.
  - 4.5 Is it likely that assessment of the outcome was influenced by knowledge of intervention received? → No
    Rationale: Given the objective measurement of death and the consistency of measurement across groups, it is unlikely that the assessment was influenced by the intervention knowledge.
- Risk Assessment: Low
- Reasoning:
  The measurement method for all-cause mortality is appropriate and objective. Although outcome assessors were aware of the intervention assignment (a consequence of the study's unblinded design), the objective nature of mortality data and consistent measurement across groups ensure that such awareness is unlikely to have introduced bias. Therefore, based on the predefined decision rules, the overall risk of bias in the measurement of the outcome is LOW risk.

Domain 5: Risk of Bias in Selection of the Reported Result

- Signaling Question Analysis:
  - 5.1 Were the data that produced this result analysed in accordance with a pre-specified analysis plan? → Probably yes
    Rationale: The study was registered (ClinicalTrials.gov NCT00360958) and states that the primary analysis followed an intention-to-treat principle. Although explicit details confirming that the analysis plan was finalized before unblinding are not provided, trial registration implies that a pre-specified plan was in place.

- 5.2 Is the numerical result being assessed likely to have been selected from multiple eligible outcome measurements? → No
  Rationale: The outcome under consideration (all-cause mortality) is an objective and singular measure, and there is no evidence that multiple eligible outcome measurements were available or selectively reported.
- 5.3 Is the numerical result being assessed likely to have been selected from multiple eligible analyses of the data? → No
  Rationale: The analysis appears to have followed the pre-specified intention-to-treat approach with no indication of selective analysis reporting.

- Risk of Bias Judgment:
  Since both the possibility of multiple outcome measurements and multiple analyses are answered as "No" (i.e., {N, PN}), and the trial appears to have been analyzed according to a pre-specified plan (5.1 = "Probably yes"), the decision rule leads to a LOW risk of bias for Domain 5.
- Reasoning Summary:
  The available evidence (trial registration and adherence to an ITT analysis) supports that the data were analyzed according to a pre-specified plan, and there is no indication of selective reporting in terms of outcome measurements or analyses. Therefore, the overall risk of bias in the selection of the reported result is LOW risk.

ID: Liu 2021

First Response

Domain 1: Bias Arising from the Randomization Process

- Signaling Question Analysis:
  - 1.1 Was the allocation sequence random? → Yes
    The study states that patients were randomized using a computer-generated list via SPSS, which is explicit evidence of randomization.
  - 1.2 Was the allocation sequence concealed? → No information
    The trial is described as double-blinded, and anesthesiologists were blinded; however, there is no explicit description of secure allocation concealment methods (e.g., sealed envelopes or central randomization).
  - 1.3 Did baseline differences suggest a problem with randomization? → No
    Baseline characteristics (age, BMI, TNM stage, HAMD-17 score) were reported as not significantly different between groups, indicating no baseline imbalances.
- Risk Assessment: Some Concerns
- Reasoning:
  - Although the allocation sequence was explicitly randomized (SQ1 = Yes), the study does not provide clear information on how the allocation sequence was concealed (SQ2 = No information).
  - Given that there are no baseline imbalances (SQ3 = No), rule 4.1 applies: with allocation concealment marked as "No information" and baseline imbalances as "No," the overall risk of bias for this domain is judged as Some Concerns.
  - No error was detected, and the decision strictly follows the predefined RoB 2.0 rules.

Domain 2: Bias Due to Deviations from Intended Interventions

- Step 1: Analysis Type Evaluation

- Analysis Classification:
  - The study reports that all 303 randomized patients were followed up with no losses. There is no mention of exclusions for reasons such as non-adherence or toxicity.
  - Classification: Intention-to-treat (ITT) is effectively in place.
- Step 2: Effect of Assignment to Intervention
  - 2.1 Were participants aware of their assigned intervention? → No
    The study is described as double-blinded, indicating effective participant blinding.
  - 2.2 Were carers and people delivering the interventions aware of participants' assigned intervention? → No
    Anesthesiologists and the intervention team were blinded to the treatment allocation.
  - 2.3 Were there deviations from the intended intervention that arose because of the trial context? → No
    The protocol was uniformly applied across groups, with no reported deviations due to trial-specific factors.
  - 2.4 Were these deviations likely to have affected the outcome? → No
    Since no deviations are reported, there is no impact expected on the primary outcome (Quality of life).
  - 2.5 Were these deviations from intended intervention balanced between groups? → Yes
    There were no deviations, implying inherent balance across treatment groups.
  - 2.6 Was an appropriate analysis used to estimate the effect of assignment to intervention? → Yes
    The study effectively followed an ITT approach by including all randomized patients in the analysis.
  - 2.7 Was there potential for a substantial impact of the failure to analyse participants in the group to which they were randomized? → No
    With complete follow-up and no exclusions, there is no substantial impact due to failure in analyzing as randomized. Risk of Bias Judgment for Domain 2:
- Part 1 (Effect of Assignment to Intervention):
  - Participants and personnel were not aware of the intervention (2.1 = No, 2.2 = No), leading to LOW risk.
  - Part 2 (Analysis Method):
  - An appropriate ITT analysis was used (2.6 = Yes) and no substantial impact from exclusions (2.7 = No), yielding LOW risk.
- Overall Judgment:
  - Since both components are LOW risk, the overall risk of bias due to deviations from intended interventions is LOW risk. Reasoning:
- The study employs an ITT analysis with complete follow-up and effective double-blinding.
- There is no evidence of deviations from the intended interventions that could affect the primary outcome.
- Both the blinding and analysis methods are robust, satisfying the criteria for LOW risk. No corrections were necessary based on the predefined RoB 2.0 rules.

Domain 3: Risk of bias due to missing outcome data

- Signaling Question Analysis:
  - 3.1 Were data for this outcome available for all, or nearly all, participants randomized? → Yes
    The study reports that no patient was lost to follow-up, meaning outcome data were available for all participants.
  - 3.2 Is there evidence that the result was not biased by missing outcome data? → Yes
    Complete follow-up indicates that missing data did not bias the outcome estimates.
  - 3.3 Could missingness in the outcome depend on its true value? → No
    Since nearly all participants provided outcome data, missingness cannot be related to the true outcome values.

- 3.4 Is it likely that missingness in the outcome depended on its true value? → No
  There is no indication of any missing data that could depend on the true outcome.

- Risk Assessment: Low risk
- Reasoning:
  - Outcome data were available for all participants (3.1 = Yes), which meets the threshold for minimal missing data.
  - There is clear evidence that missing data did not introduce bias (3.2 = Yes).
  - With complete follow-up, the possibility that missingness could depend on the true outcome is effectively eliminated (3.3 and 3.4 = No).
  - Therefore, according to the predefined decision rules, the risk of bias due to missing outcome data is Low risk.

Domain 4: Risk of Bias in Measurement of the Outcome Note: For this assessment, the primary outcome is Quality of life. The study report does not describe any instrument or method used to measure quality of life.

- Signaling Question Analysis:
  - 4.1 Was the method of measuring the outcome inappropriate? → No information
    No details are provided about the measurement instrument or procedure for quality of life, so we cannot determine whether the method was appropriate or inappropriate.
  - 4.2 Could measurement or ascertainment of the outcome have differed between intervention groups? → No information
    The report does not describe how quality of life (if measured) was ascertained, nor whether the same method was applied consistently across groups.
  - 4.3 Were outcome assessors aware of the intervention received by study participants? → No information
    There is no information regarding the blinding or awareness of assessors for the quality of life outcome.
  - 4.4 Could assessment of the outcome have been influenced by knowledge of intervention received? → No information
    Without details on the measurement method or assessor blinding for quality of life, we cannot evaluate potential influence by intervention knowledge.
  - 4.5 Is it likely that assessment of the outcome was influenced by knowledge of intervention received? → No information
    The report provides no evidence to judge the likelihood of such influence for the quality of life outcome.
- Risk Assessment: Some Concerns
- Reasoning:
  - The study does not report any information on how quality of life was measured—even though it is designated as the primary outcome for this assessment.
  - Without details on the measurement method, its consistency between groups, and the blinding of assessors, we are unable to verify that the outcome was measured appropriately and without bias.
  - Given this absence of information, and following the RoB 2.0 default rule for such cases, the overall risk of bias in measurement of the outcome is judged as Some Concerns.

Domain 5: Risk of Bias in Selection of the Reported Result

- Signaling Question Analysis:

- 5.1 Were the data that produced this result analysed in accordance with a pre-specified analysis plan? → No information
  The report does not provide details on whether a pre-specified analysis plan was finalized before unblinded outcome data were available, particularly for the quality-of-life outcome.
- 5.2 Is the numerical result being assessed likely to have been selected from multiple eligible outcome measurements within the outcome domain? → No information
  There is no information on whether the quality-of-life outcome (designated as primary for this assessment) was chosen among multiple measures or time points, nor is there evidence regarding selective reporting of different scales or definitions.
- 5.3 Is the numerical result being assessed likely to have been selected from multiple eligible analyses of the data? → No information
  The study does not describe whether various analyses were performed for the quality-of-life outcome or if a selective analysis was chosen based on the results.

- Risk Assessment: Some concerns
- Reasoning:
  - With No information provided for key aspects of analysis planning (5.1), outcome measurement selection (5.2), and analysis selection (5.3), we lack assurance that the result was reported in accordance with a pre-specified plan and without selective reporting.
  - According to the decision rules, if at least one element is rated as "No information" and there is no indication of adherence to a pre-specified plan, the risk of bias is judged as Some concerns.
  - No evidence suggests clear selective reporting (which would have led to a HIGH risk), but the absence of details prevents full confidence in the reported result for the primary outcome.

ID: Liu 2021

Second Response

Domain 1: Bias Arising from the Randomization Process

- Signaling Question Analysis:
  - Was the allocation sequence random? → Y
    The study states that patients were "randomly divided" using a computer-generated list via SPSS.
  - Was the allocation sequence concealed? → NI
    No explicit information is provided regarding the concealment of the allocation sequence.
  - Did baseline differences suggest a problem with randomization? → N
    Baseline characteristics (age, gender, TNM stage, BMI, and preoperative HAMD-17 score) were reported to be similar across groups.
- Risk Assessment: Some Concerns
  Reasoning:
  - The randomization method is clearly described (SQ1.1 = Y), fulfilling the requirement for an appropriate random sequence generation.
  - However, the study does not provide explicit details on allocation concealment (SQ1.2 = NI).
  - Baseline comparisons reveal no significant imbalances (SQ1.3 = N).
  - According to the predefined decision rule:

    - If allocation sequence concealment is rated as NI and baseline imbalances are rated as N, the overall judgment should be Some Concerns.
  - Thus, the overall risk of bias for Domain 1 is determined as Some Concerns.

Domain 2: Bias Due to Deviations from Intended Interventions

- Preliminary Analysis (Step 1 – ITT vs PP):
  The study enrolled 303 patients with no losses to follow-up and does not report any post-randomization exclusions. This indicates that all randomized participants were analyzed, which is consistent with an intention-to-treat (ITT) approach.
- Signaling Question Analysis (Effect of Assignment to Intervention – Step 2):
  - 2.1 Were participants aware of their assigned intervention? → No
    The trial is described as "double-blinded," implying that participants were not aware of their allocation.
  - 2.2 Were carers and people delivering the interventions aware of participants' assigned intervention? → No
    The study explicitly states that the anesthesiologists were blinded to the drugs used after analgesia induction.
  - 2.3 Were there deviations from the intended intervention that arose because of the trial context? → No
    There is no indication of any deviations from the intervention protocol; all participants received the intended treatments as per group assignment.
  - 2.4 Were these deviations likely to have affected the outcome? → No
    Since no deviations were reported, there is no evidence suggesting that any such deviations could have influenced the outcome.
  - 2.5 Were these deviations from intended intervention balanced between groups? → Yes
    In the absence of any deviations, the potential for imbalance is not a concern.
  - 2.6 Was an appropriate analysis used to estimate the effect of assignment to intervention? → Yes
    The analysis included all randomized patients with no losses to follow-up, which is consistent with an ITT approach that is appropriate for assessing the effect of assignment.
  - 2.7 Was there potential for a substantial impact of the failure to analyze participants in the group to which they were randomized? → No
    Since no exclusions or misclassifications occurred, there is no potential for a substantial impact on the results.
- Risk Assessment: Low risk
- Reasoning:
  - The study appears to implement an ITT approach as all randomized participants were followed and included in the analysis.
  - Both participants and the intervention providers (anesthesiologists) were blinded, which minimizes the possibility of awareness-related deviations.
  - There is no evidence of deviations from the intended interventions, and any potential deviations would have been evenly distributed across groups.
  - The statistical analysis was appropriate for an ITT framework, with no reported exclusions or protocol violations that might bias the effect estimate.
  - According to the predefined decision rules, with both Part 1 (participant and personnel awareness/deviations) and Part 2 (analysis appropriateness) rated as LOW risk, the overall risk of bias due to deviations from intended interventions is therefore Low risk.

Domain 3: Risk of bias due to missing outcome data

- Signaling Question Analysis:
  - Were data for this outcome available for all, or nearly all, participants randomized? → Yes
    The study explicitly states that no patient was lost to follow-up, indicating that outcome data were available for all participants.
  - Is there evidence that the result was not biased by missing outcome data? → Yes
    Since there were no missing data, there is clear evidence that the result could not be biased by missing outcome data.
  - Could missingness in the outcome depend on its true value? → No
    With complete outcome data, there is no possibility for missingness to depend on the true value.
  - Is it likely that missingness in the outcome depended on its true value? → No
    Given that all participants provided outcome data, there is no likelihood of bias arising from missing data.
- Risk Assessment: Low risk
- Reasoning:
  - Outcome data were obtained for all randomized participants (≥95% completeness), which meets the criteria for minimal concern regarding missing outcome data.
  - There is no evidence suggesting that any missing data could have introduced bias, nor that any missingness was related to the true outcome values.
  - According to the predefined decision rules, when outcome data for all participants is available and there is clear evidence that missing data did not bias the results, the overall risk of bias due to missing outcome data is assessed as Low risk.

Domain 4: Risk of bias in measurement of the outcome

- Signaling Question Analysis:
  - 4.1 Was the method of measuring the outcome inappropriate? → No
    The HAMD-17 is a well-validated and widely used scale for assessing depression, making it an appropriate choice for this outcome measurement.
  - 4.2 Could measurement or ascertainment of the outcome have differed between intervention groups? → No
    The same measurement instrument (HAMD-17) was uniformly applied across all intervention groups, ensuring consistency in outcome assessment.
  - 4.3 Were outcome assessors aware of the intervention received by study participants? → No
    Given that the trial is described as double-blinded, it is reasonable to assume that outcome assessors were blinded to group allocation.
  - 4.4 Could assessment of the outcome have been influenced by knowledge of intervention received? → No
    With outcome assessors blinded, it is unlikely that the measurement of the outcome was influenced by knowledge of the intervention received.
  - 4.5 Is it likely that assessment of the outcome was influenced by knowledge of intervention received? → No
    The effective blinding of assessors minimizes the possibility that outcome assessment was influenced by intervention knowledge.
- Risk Assessment: Low risk
- Reasoning:
  - The study employs the HAMD-17 scale, a standard and validated tool, for measuring depression, which is appropriate for this continuous outcome.

- Outcome measurement was consistent across groups as the same instrument was used, ensuring no differential ascertainment.
- The trial's double-blinded design supports the conclusion that outcome assessors were not aware of the treatment assignments, thereby reducing potential measurement bias.
- Following the predefined decision rules, with appropriate measurement methods and uniform assessment across groups combined with blinded outcome assessors, the overall risk of bias in the measurement of the outcome is determined to be Low risk.

Domain 5: Risk of bias in selection of the reported result

- Signaling Question Analysis:
  - 5.1 Were the data analysed in accordance with a pre-specified plan? → No information
    The article does not provide details confirming that an analysis plan was finalized before unblinded outcome data became available.
  - 5.2 Were multiple eligible outcome measurements available and selectively reported? → No
    Although the HAMD-17 was measured at several time points (3 days, 1 week, 1 month, and 3 months), the study reports results for all these time points, indicating no selective reporting based on outcome measurement selection.
  - 5.3 Were multiple eligible analyses performed and selectively reported? → No
    The study applies consistent statistical methods (ANOVA with Tukey's post-hoc tests) across the outcomes, with no indication that the results were chosen from among multiple analyses.
- Risk Assessment: Some Concerns
- Reasoning:
  - Both the eligible outcome measurements (5.2) and analyses (5.3) show no evidence of selective reporting (answers are in {N}).
  - However, because there is no information regarding whether the data were analysed according to a pre-specified plan (5.1 = NI), the decision rule directs us to judge the overall risk as Some Concerns.
  - In summary, while there is no clear sign of selective outcome or analysis reporting, the lack of explicit confirmation regarding a pre-specified analysis plan prevents a definitive low-risk rating.

ID: Kaital 2011

First Response

Domain 1: Bias Arising from the Randomization Process

- Signaling Question Analysis:
  - 1.1 Was the allocation sequence random? → Probably Yes (PY)
    Rationale: The study is described as a randomized, double-blind, parallel-group trial. Although no detailed method is provided, the designation "randomized" provides strong contextual evidence.
  - 1.2 Was the allocation sequence concealed? → No Information (NI)
    Rationale: The report does not provide explicit details on how the allocation sequence was concealed.
  - 1.3 Did baseline differences suggest a problem with randomization? → No (N)
    Rationale: Baseline characteristics and demographics were reported as well balanced between the treatment groups.
- Risk Assessment: Some Concerns
- Reasoning:

- The allocation sequence is described as randomized (PY for SQ1.1), suggesting a proper random process, yet the method is not detailed.
- There is no explicit information regarding allocation concealment (NI for SQ1.2).
- Baseline imbalances are not evident (N for SQ1.3).
- Per the predefined RoB 2.0 rules, when allocation concealment is rated as NI and baseline imbalances are absent (N), the overall risk for this domain should be assessed as Some Concerns.

Domain 2: Bias Due to Deviations from Intended Interventions

- Step 1: Analysis Population Approach
    - Analysis Type: The study clearly states that "The analysis population was the ITT population, which included all subjects randomized to study drug."
      Interpretation: This indicates an intention-to-treat approach, meaning that all randomized participants were analyzed, regardless of subsequent deviations.
- Step 2: Effect of Assignment to Intervention
    - 2.1 Were participants aware of their assigned intervention during the trial? → No
      Rationale: The study is described as "double-blind," implying that participants were effectively blinded.
    - 2.2 Were carers and people delivering the interventions aware of participants' assigned intervention during the trial? → No
      Rationale: As a double-blind study, the personnel involved were not aware of the group assignments.
    - 2.3 Were there deviations from the intended intervention that arose because of the trial context? → No
      Rationale: There is no evidence or report of deviations stemming from trial-specific factors.
    - 2.4 Were these deviations likely to have affected the outcome? → No
      Rationale: With no notable deviations reported, there is no suggestion that any departures impacted the primary outcome (asthma exacerbation).
      2.5 Were these deviations from intended intervention balanced between groups? → Yes
      Rationale: Even if minor deviations occurred, they appear to be balanced given the similar conduct across study arms.
    - 2.6 Was an appropriate analysis used to estimate the effect of assignment to intervention? → Yes
      Rationale: An ITT analysis was used, which is the recommended method to assess the effect of assignment.
      2.7 Was there potential for a substantial impact of the failure to analyse participants in the group to which they were randomized? → No
      Rationale: All randomized participants were included in the ITT population; while withdrawals occurred (26% in one group, 23% in the other), these were balanced and accounted for within the ITT framework.
    - Risk Judgment for Effect of Assignment: LOW risk
- Step 3: Effect of Adhering to Intervention
    - 2.1 Were participants aware of their assigned intervention during the trial? → No
      Rationale: As established, effective blinding ensured participants were not aware.
    - 2.2 Were carers and people delivering the interventions aware of participants' assigned intervention during the trial? → No
      Rationale: Blinding extended to carers/intervention providers.
      2.3 Were important non-protocol interventions balanced across intervention groups? → Yes
      Rationale: There is no indication of imbalanced non-protocol interventions.

- 2.4 Were there failures in implementing the intervention that could have affected the outcome? → No
  Rationale: High treatment compliance (approximately 93% in both groups) suggests that the interventions were implemented as intended.
- 2.5 Was there non-adherence to the assigned intervention regimen that could have affected participants' outcomes? → No
  Rationale: Reported adherence was high and similar between groups.
- 2.6 Was an appropriate analysis used to estimate the effect of adhering to intervention?
  Not required, since there were no concerning implementation or adherence issues.
- Risk Judgment for Effect of Adhering: LOW risk

- Overall Risk Assessment for Domain 2:
  - Combined Judgment: Since both the effect of assignment (Step 2) and the effect of adherence (Step 3) are assessed as LOW risk, the overall risk of bias due to deviations from intended interventions is LOW risk.
- Reasoning Summary:
  - The study employed an ITT analysis, including all randomized subjects.
  - Double-blinding ensured that both participants and personnel were unaware of group assignments, minimizing potential bias from deviations.
  - There is no evidence of meaningful deviations or imbalances that could have affected the primary outcome (asthma exacerbation).
  - High treatment compliance further supports that the interventions were adhered to as intended.
- Thus, based on the predefined RoB 2.0 criteria and the information extracted from the study, Domain 2 is assessed as LOW risk.

Domain 3: Risk of Bias Due to Missing Outcome Data

- Signaling Question Analysis:
  - 3.1 Were data for this outcome available for all, or nearly all, participants randomized? → No
    Rationale: Approximately 25% of participants (26% in the FSC group and 23% in the FP group) withdrew during the 52-week period. This level of missing outcome data is not minimal (i.e., it is well below the ≥95% threshold for continuous outcomes), raising concerns about missing data.
  - 3.2 Is there evidence that the result was not biased by missing outcome data? → No Information
    Rationale: The report does not provide explicit sensitivity analyses or detailed discussion on whether missing outcome data (or its handling, such as through imputation) could have biased the results.
  - 3.3 Could missingness in the outcome depend on its true value? → No Information
    Rationale: There is no clear analysis or commentary on whether the likelihood of missing outcome data was related to the actual occurrence of asthma exacerbations.
  - 3.4 Is it likely that missingness in the outcome depended on its true value? → Probably No
    Rationale: Although a substantial proportion of participants withdrew, the reasons for withdrawal appear similar between treatment groups. This suggests that—even if missing data are present—they are unlikely to be strongly driven by the outcome (i.e., the true rate of asthma exacerbations), but some uncertainty remains.
- Risk Assessment: Some Concerns
- Reasoning:
  - The study reports that about one-quarter of randomized participants withdrew, indicating that outcome data were not available for nearly all participants.

- There is no explicit evidence provided that the missing data were handled in a way that rules out bias (e.g., through sensitivity analyses or robust imputation methods), so we cannot confirm that missing data did not influence the results.
- Although the similar withdrawal rates between groups imply that missingness may not be strongly related to the true outcome values, the absence of detailed analyses on this point leaves residual uncertainty.
- Following the predefined RoB 2.0 decision rules, the combination of substantial missing outcome data with insufficient evidence to rule out bias leads to an overall judgment of Some Concerns for Domain 3.

Domain 4: Risk of Bias in Measurement of the Outcome

- Signaling Question Analysis:
  - 4.1 Was the method of measuring the outcome inappropriate? → No
    Rationale: The study used a pre-specified, composite definition for "asthma attack" incorporating objective criteria (e.g., a 20% decrease in A.M. PEF on consecutive days, increased albuterol use, and healthcare utilization for exacerbations). While composite, the approach is standard in asthma trials and is appropriate for capturing the outcome.
  - 4.2 Could measurement or ascertainment of the outcome have differed between intervention groups? → No
    Rationale: Both intervention groups employed the same measurement tools (electronic peak flow meters, diaries, and standardized definitions for exacerbations), suggesting consistency in outcome ascertainment across groups.
  - 4.3 Were outcome assessors aware of the intervention received by study participants? → No
    Rationale: The trial was conducted in a double-blind fashion, which implies that outcome assessors were blinded to treatment allocation.
  - 4.4 Could assessment of the outcome have been influenced by knowledge of intervention received? → No
    Rationale: With blinded assessors and standardized, objective measurement criteria, it is unlikely that the assessment was influenced by knowledge of the intervention.
  - 4.5 Is it likely that assessment of the outcome was influenced by knowledge of intervention received? → No
    Rationale: Effective blinding minimizes the risk that outcome assessment was affected by intervention knowledge.
- Risk Assessment: LOW risk
- Reasoning:
  - The measurement method (a composite outcome based on objective criteria) is appropriate for assessing asthma exacerbations.
  - Outcome ascertainment was conducted consistently across groups with the same devices and definitions, reducing the likelihood of differential measurement.
  - Double-blinding ensured that both participants and outcome assessors were unaware of treatment assignments, further minimizing potential bias in measurement.
  - Following the predefined RoB 2.0 rules, the absence of concerns at each signaling question level leads to a final judgment of LOW risk for this domain.

Domain 5: Risk of Bias in Selection of the Reported Result

- Signaling Question Analysis:

- 5.1 Were the data that produced this result analysed in accordance with a pre-specified analysis plan that was finalized before unblinded outcome data were available? → Probably yes (PY)
  Rationale: The published report indicates that analyses were conducted using a predefined statistical approach (e.g., ANCOVA for the primary outcome, hierarchical testing to control type I error, and the use of an ITT population). Although the article does not explicitly state that the analysis plan was finalized prior to unblinding, the trial's registration (ClinicalTrials.gov identifier: NCT00452699) and use of standard procedures suggest that a pre-specified plan likely existed.
- 5.2 Is the numerical result being assessed likely to have been selected, on the basis of the results, from multiple eligible outcome measurements within the outcome domain? → Probably yes (PY)
  Rationale: The study assessed "asthma attacks" using a composite outcome (including declines in A.M. PEF, increased albuterol use, and healthcare utilization) and then reported a separate, post hoc analysis of "asthma exacerbations" (a subset of the composite with more stringent criteria). This indicates that multiple eligible outcome measurements were available, and the selection of the numerical result (i.e., the significant difference in exacerbation rates) may have been influenced by the observed results.
- 5.3 Is the numerical result being assessed likely to have been selected, on the basis of the results, from multiple eligible analyses of the data? → Probably yes (PY)
  Rationale: In addition to the primary analysis of the composite "asthma attacks," a post hoc analysis was performed to dissect which components contributed to the overall result. The existence of multiple analytical approaches (pre-specified composite analysis versus post hoc analysis of exacerbations) raises concerns that the reported significant result could have been selectively chosen from among several potential analyses.

- Risk Assessment: HIGH risk of bias
- Reasoning:
  - Although there is reasonable confidence (PY) that the trial was analyzed according to a pre-specified plan (5.1), the presence of multiple eligible outcome measurements (composite "asthma attacks" versus individual "asthma exacerbations") and the conduct of a post hoc analysis (5.2 and 5.3, both rated as PY) indicate that selective reporting may have influenced which numerical result was ultimately highlighted.
  - Following the predefined RoB 2.0 decision rule—if either multiple outcome measurements or multiple analyses are judged as being potentially selectively reported (rated as Y/PY), then the overall risk of bias for this domain is HIGH—the final judgment for Domain 5 is HIGH risk.

ID: Kaital 2011

Second Response

Domain 1: Bias Arising from the Randomization Process

- Signaling Question Analysis:
  - Was the allocation sequence random? → Y
    The study is described as a randomized trial, which is explicit evidence that the allocation sequence was random.
  - Was the allocation sequence concealed? → PY
    While no detailed method is provided, the double-blind design strongly suggests that allocation concealment was likely properly implemented.

- Did baseline differences suggest a problem with randomization? → N

  Baseline characteristics were reported as well balanced, with no substantial differences between groups.

- Risk Assessment: Low Risk
- Reasoning:
  - Step 1: The trial explicitly states it is randomized, so the allocation sequence is marked as Y.
  - Step 2: Although the text does not provide explicit details on the concealment method, the double-blind design provides strong contextual clues for proper concealment, hence PY.
  - Step 3: Baseline data indicate no imbalances between treatment arms (N).
  - Decision: According to Rule 3 of the RoB 2.0 decision tree—allocation sequence concealed in {Y, PY} AND allocation sequence random in {Y, PY, NI} AND baseline imbalances in {N, PN, NI}—the overall risk of bias for this domain is Low Risk.

Domain 2: Bias Due to Deviations from Intended Interventions

- Step 1: Analysis Population
  - The study explicitly states that the analysis population was the ITT population (all randomized subjects were included). → Analysis Type: ITT
- Step 2: Effect of Assignment to Intervention
  - 2.1 Were participants aware of their assigned intervention during the trial? → No

    The trial is described as double-blind, indicating that participants were effectively blinded.
  - 2.2 Were carers and people delivering the interventions aware of participants' assigned intervention during the trial? → No

    The double-blind design implies that personnel were also blinded to treatment assignments.
  - 2.3 Were there deviations from the intended intervention that arose because of the trial context? → No

    There is no evidence of deviations related to the trial context; the protocol was followed with high treatment compliance.
  - 2.4 Were these deviations likely to have affected the outcome? → No

    Since no deviations were observed, there is no impact on the outcome.
  - 2.5 Were these deviations from intended intervention balanced between groups? → Yes

    Any minor deviations (e.g., withdrawals) were similar between groups and do not suggest imbalance.
  - 2.6 Was an appropriate analysis used to estimate the effect of assignment to intervention? → Yes

    The use of an ITT analysis confirms that all randomized subjects were analyzed, which is appropriate for preserving the treatment assignment effect.
  - 2.7 Was there potential for a substantial impact (on the result) of the failure to analyse participants in the group to which they were randomized? → No

    With an ITT approach and minimal exclusions, there is no substantial impact.
- Risk Assessment for Step 2 (Effect of Assignment to Intervention):
  - Part 1:
    - Participants and personnel were both unaware of the assignment (answers: No) → LOW risk.
  - Part 2:
    - Appropriate ITT analysis was used (answer: Yes) and no substantial impact from exclusions → LOW risk.

- Step 3: Effect of Adhering to Intervention
  (Not applicable here because the ITT analysis focuses on assignment rather than adherence, and no significant deviations or implementation issues were identified.)
- Final Risk of Bias Judgment for Domain 2:
  - Since Part 1 = LOW risk and Part 2 = LOW risk, the overall risk of bias for Domain 2 is Low Risk.

Domain 3: Risk of Bias Due to Missing Outcome Data

- Signaling Question Analysis:
  - 3.1 Were data for this outcome available for all, or nearly all, participants randomized? → Probably yes
    Although approximately 25% of participants withdrew during the 52-week period, the trial employed an ITT approach and used methods (e.g., negative binomial regression with time offsets, survival analysis) that incorporated all available follow-up data. This suggests that sufficient outcome data were captured such that the missing data are unlikely to have altered the estimated effect.
  - 3.2 Is there evidence that the result was not biased by missing outcome data? → Probably yes
    The analysis population included all randomized subjects, and there is no indication that missing data introduced systematic bias. The withdrawal rates were similar between groups, and the analysis methods appropriately accounted for differential follow-up.
  - 3.3 Could missingness in the outcome depend on its true value? → Probably no
    There is no evidence to suggest that the likelihood of missing outcome data was related to the actual occurrence of asthma exacerbations; withdrawal reasons appear balanced between groups.
  - 3.4 Is it likely that missingness in the outcome depended on its true value? → Probably no
    Given the balanced withdrawal rates and the robust ITT analysis, it is unlikely that missingness was related to the true outcome values.
- Risk Assessment: Low Risk
- Reasoning:
  - Step 1: Although not every participant completed the full 52-week follow-up, the trial's ITT population and analytical methods ensured that nearly all available outcome data were utilized. This meets the criterion for "all or nearly all" in the context of event outcomes.
  - Step 2: There is evidence (e.g., balanced withdrawal rates, appropriate statistical models) that missing data did not bias the results, supporting a "Probably yes" response for the absence of bias from missing data.
  - Steps 3 & 4: There is no indication that the missingness depended on the true values of the outcome; responses of "Probably no" for both questions further reduce concern.
  - Decision: Since the outcome data are sufficiently complete (as indicated by a "Probably yes" for 3.1) and there is no strong evidence of bias from missing data or dependency on true outcomes, the decision rule directs a Low Risk rating for Domain 3.

Domain 4: Risk of Bias in Measurement of the Outcome

- Signaling Question Analysis:

- 4.1 Was the method of measuring the outcome inappropriate? → No
  The study employed clearly defined, objective criteria for asthma exacerbations (e.g., requirement for systemic corticosteroids, unscheduled urgent care visits) that are appropriate for capturing the outcome of interest.
- 4.2 Could measurement or ascertainment of the outcome have differed between intervention groups? → No
  The trial's double-blind design and standardized outcome definitions indicate that measurement was consistent across groups.
- 4.3 Were outcome assessors aware of the intervention received by study participants? → No
  Outcome assessors were blinded, consistent with the overall double-blind design of the study.
- 4.4 Could assessment of the outcome have been influenced by knowledge of intervention received? → No
  Given the objective nature of the outcome measures and the blinding procedures in place, it is unlikely that assessment was influenced by knowledge of the treatment allocation.
- 4.5 Is it likely that assessment of the outcome was influenced by knowledge of intervention received? → No
  There is no indication that knowledge of the intervention influenced outcome assessment.

- Risk Assessment: Low Risk
- Reasoning:
  - Step 1: The method used to measure the outcome was appropriate (answer to 4.1 is "No" for inappropriateness).
  - Step 2: Measurement was consistent between intervention groups (4.2 = No), and outcome assessors were blinded (4.3 = No), ensuring objectivity in the assessment.
  - Step 3: There is no evidence or indication that the assessment was influenced by knowledge of the intervention (4.4 and 4.5 are both "No").
  - Decision: Based on the predefined rules, with all signaling questions indicating no concerns regarding measurement bias, the overall risk of bias for Domain 4 is Low Risk.

Domain 5: Risk of Bias in Selection of the Reported Result

- Signaling Question Analysis:
  - 5.1 Pre-specified analysis plan: → Probably yes
    The report provides details on statistical methods (e.g., hierarchical testing, use of the Hochberg method, ITT analysis) and references trial registration (ClinicalTrials.gov identifier: NCT00452699). Although it does not explicitly state that the analysis plan was finalized before unblinding, the available protocol details suggest a pre-specified plan existed.
  - 5.2 Multiple eligible outcome measurements: → No
    All eligible outcome measurements relevant to the risk domain (including definitions for asthma exacerbation and composite "asthma attacks") appear to have been reported according to protocol, with no clear indication of selective outcome measurement reporting.
  - 5.3 Multiple eligible analyses of the data: → No
    The study reports its primary and secondary analyses using standard, pre-specified methods without evidence of selective reporting among multiple analyses.
- Risk Assessment: Low Risk
- Reasoning:
  - Both the possibility of multiple outcome measurements and multiple analyses were evaluated as not concerning (rated "No").

- The available evidence suggests that the trial was analyzed in accordance with a pre-specified plan (rated "Probably yes"), even though explicit confirmation of finalization before unblinding is not provided.
- According to the decision rules, when both potential sources of selective reporting are not an issue (i.e., in {N, PN}) and the trial analysis follows a pre-specified plan (in {Y, PY}), the overall risk of bias for this domain is Low Risk.

ID: Munteanu 2021

First Response

Domain 1: Bias Arising from the Randomization Process

- Signaling Question Analysis:
  - 1.1 Was the allocation sequence random? → Y
    The trial used a minimisation process with stratification, a recognized method for random allocation.
  - 1.2 Was the allocation sequence concealed? → Y
    Allocation was managed via an interactive voice response telephone service, providing secure concealment.
  - 1.3 Did baseline differences suggest a problem with randomization? → N
    Baseline characteristics were generally similar, with no substantial imbalances reported.
- Risk Assessment: LOW risk
- Reasoning:
  - The allocation sequence was explicitly randomized (Y) using minimisation and stratification.
  - The allocation sequence was securely concealed (Y) through an independent service.
  - No substantial baseline imbalances were observed (N).
    According to the predefined RoB 2.0 rule (rule 3), when allocation concealment is Y, the randomization method is Y, and baseline differences are N, the overall risk of bias for this domain is LOW risk.
    No inconsistencies were detected that would necessitate any correction.

Domain 2: Bias Due to Deviations from Intended Interventions

- Step 1: Analysis Approach
  - Analysis Type: The study employed an intention-to-treat (ITT) analysis.
    All 100 randomized participants were included in the primary outcome analysis, and missing data were addressed using multiple imputation.
- Step 2: Effect of Assignment to Intervention
  - 2.1 Were participants aware of their assigned intervention during the trial? → No
    Participants were blinded to their group allocation.
  - 2.2 Were carers and people delivering the interventions aware of participants' assigned intervention during the trial? → Yes
    Research staff administering the shoe inserts were aware of group allocation.
  - 2.3 Were there deviations from the intended intervention that arose because of the trial context? → No
    There is no indication that deviations arose due to the trial context.
  - 2.4 Were these deviations likely to have affected the outcome? → No
    There is no evidence that any deviations (if present) impacted the primary outcome.

- 2.5 Were these deviations from intended intervention balanced between groups? → Yes
  Adherence data indicate that any deviations (such as slight differences in insert wearing hours) were balanced between groups.
- 2.6 Was an appropriate analysis used to estimate the effect of assignment to intervention? → Yes
  An ITT analysis with multiple imputation was used, which is appropriate and minimizes bias.
- 2.7 Was there potential for a substantial impact of the failure to analyse participants in the group to which they were randomized?→ No
  All participants were analyzed in the groups to which they were randomized, with no evidence of substantial impact from exclusions.

- Risk Assessment for Step 2:
  - Part 1 (Impact of Deviations): LOW risk
    (Participants were effectively blinded, and although personnel were aware, there were no trial-context deviations affecting the outcome.)
  - Part 2 (Appropriate Analysis): LOW risk
    (The use of ITT with appropriate handling of missing data confirms a low risk.)
- Since both Part 1 and Part 2 are LOW risk, the overall assessment for the effect of assignment to intervention is LOW risk.
- Step 3: Effect of Adhering to Intervention
  Not applicable in this instance because the study used an ITT analysis (the evaluation focused on the effect of assignment rather than the effect of adhering to the intervention).
- Overall Risk Assessment for Domain 2: LOW risk
- Reasoning:
  - The study utilized an ITT analysis, including all randomized participants and properly handling missing data.
  - Participants were effectively blinded, reducing bias from their behavior, even though the personnel administering the interventions were not blinded.
  - No deviations from the intended interventions attributable to the trial context were reported, and any minor adherence differences were balanced between groups.
  - The combination of these factors, along with the appropriate statistical approach, results in a LOW risk of bias for Domain 2.

Domain 3: Risk of Bias Due to Missing Outcome Data

- Signaling Question Analysis:
  - 3.1 Were data for this outcome available for all, or nearly all, participants randomized? → N
    Only about 86–88% of participants provided complete outcome data at 12 weeks, which is below the ≥95% threshold required for a "Yes."
  - 3.2 Is there evidence that the result was not biased by missing outcome data? → Y
    The study employed multiple imputation and reported that a complete case analysis produced similar results, indicating that missing data did not bias the outcome estimate.
  - 3.3 Could missingness in the outcome depend on its true value? → Probably no
    There is no clear evidence that the reasons for missing data were related to the participants' true pain outcomes.

    - 3.4 Is it likely that missingness in the outcome depended on its true value? → No
      Given the similar findings from complete case and imputed analyses, it is unlikely that missingness depended on the true outcome values.
- Risk Assessment: LOW risk
- Reasoning:
  Although complete outcome data were available for less than 95% of participants (3.1: N), the use of multiple imputation and confirmation via complete case analysis (3.2: Y) provide clear evidence that the missing outcome data did not bias the results. Additionally, there is little indication that the missingness was related to the true outcome values (3.3: PN and 3.4: N). According to the predefined decision rules, this leads to an overall LOW risk of bias for Domain 3.

Domain 4: Risk of Bias in Measurement of the Outcome

- Signaling Question Analysis:
    - 4.1 Was the method of measuring the outcome inappropriate? → No
      The FHSQ pain domain is a validated and widely used instrument to assess foot pain, making it an appropriate measure.
    - 4.2 Could measurement or ascertainment of the outcome have differed between intervention groups? → No
      The same measurement method was applied uniformly across both intervention groups.
    - 4.3 Were outcome assessors aware of the intervention received by study participants? → No
      Outcome assessors were blinded, ensuring they did not know participants' group assignments.
    - 4.4 Could assessment of the outcome have been influenced by knowledge of intervention received? → No
      Blinding of both participants and outcome assessors minimizes the possibility of biased assessment.
    - 4.5 Is it likely that assessment of the outcome was influenced by knowledge of intervention received? → No
      There is no indication that any unblinding occurred; thus, the assessment is unlikely to have been influenced.
- Risk Assessment: LOW risk
- Reasoning:
    - The measurement method (FHSQ pain domain) is appropriate (4.1: No).
    - There is consistency in the measurement method across both groups (4.2: No).
    - Outcome assessors were blinded to group allocation (4.3: No), ensuring that their awareness did not bias the outcome assessment.
    - With both participants and assessors blinded, the risk that the measurement of outcome was influenced by knowledge of intervention is minimal (4.4 & 4.5: No).
      According to the RoB 2.0 rules, when the measurement method is appropriate, applied uniformly, and assessors are blinded, the overall risk of bias due to outcome measurement is LOW.

Domain 5: Risk of Bias in Selection of the Reported Result

- Signaling Question Analysis:
    - 5.1 Were the data that produced this result analysed in accordance with a pre-specified analysis plan that was finalized before unblinded outcome data were available for analysis? → Yes
      The trial was prospectively registered and the full protocol was published, indicating that the analysis plan was pre-specified before unblinded data were available.
    - 5.2 Is the numerical result being assessed likely to have been selected, on the basis of the results, from multiple eligible outcome measurements (e.g., scales, definitions, time points) within the outcome domain? → No

The primary outcome (FHSQ pain domain at 12 weeks) was pre-specified and consistently reported, with no indication of selective reporting among multiple eligible measures.

- 5.3 Is the numerical result being assessed likely to have been selected, on the basis of the results, from multiple eligible analyses of the data? → No
  The statistical analysis followed a pre-specified plan (using ITT, ANCOVA, and multiple imputation), and the complete case analysis yielded similar results, suggesting no selective reporting of analyses.

- Risk Assessment: LOW risk
- Reasoning:
  - The trial's analysis adhered to a pre-specified plan (5.1: Yes).
  - There is no evidence of selective reporting based on choosing among multiple eligible outcome measurements (5.2: No) or multiple analyses of the data (5.3: No).
    According to the decision rules, when both the potential for selective outcome measurement and analysis is rated as No (or PN) and the pre-specified analysis plan is confirmed (Yes), the overall risk of bias due to selection of the reported result is LOW risk.

ID: Munteanu 2021

Second Response

Domain 1: Bias Arising from the Randomization Process

- Signaling Question Analysis:
  - 1.1 Was the allocation sequence random? → Y
    The study is described as a randomized controlled trial and used a minimisation procedure via an interactive voice response telephone service, providing explicit evidence of randomization.
  - 1.2 Was the allocation sequence concealed? → Y
    The use of a central interactive telephone service by the NHMRC Clinical Trials Centre indicates secure concealment of the allocation sequence.
  - 1.3 Did baseline differences suggest a problem with randomization? → N
    The study reports that participants in the two groups had similar baseline characteristics, indicating no substantial imbalances.
- Risk Assessment: Low
- Reasoning:
  - The allocation sequence was both random (SQ1.1: Y) and securely concealed (SQ1.2: Y).
  - Baseline characteristics were balanced (SQ1.3: N), indicating no evidence of problems with randomization.
  - Following the RoB 2.0 decision rule that if the allocation sequence is concealed in {Y, PY} and randomization is in {Y, PY, NI} with baseline imbalances in {N, PN, NI}, then the risk of bias is Low, we conclude that Domain 1 is at Low risk.

Domain 2: Bias Due to Deviations from Intended Interventions

- Signaling Question Analysis:
  Step 1: Analysis Type

    - The study explicitly states that statistical analysis was undertaken using the intention-to-treat principle with all 100 randomised participants included and missing outcome data addressed via multiple imputation.
      → Analysis Type: ITT
- Step 2: Effect of Assignment to Intervention
    - 2.1 Were participants aware of their assigned intervention during the trial? → No
      The trial was described as participant-blinded.
    - 2.2 Were carers and people delivering the interventions aware of participants' assigned intervention during the trial? → Yes
      Due to the nature of the intervention, research staff administering the shoe inserts were not blinded.
    - 2.3 Were there deviations from the intended intervention that arose because of the trial context? → No
      Although adherence (hours of insert wear and days of rehabilitation therapy) decreased over time, these changes reflect typical real-world behavior rather than trial-induced deviations.
    - 2.4 Were these deviations likely to have affected the outcome?→ No
      There is no evidence that the modest adherence changes impacted the primary outcome (mean overall pain).
    - 2.5 Were these deviations from intended intervention balanced between groups? → Yes
      Adherence data (both hours of insert use and rehabilitation days) were similar across the intervention and control groups.
    - 2.6 Was an appropriate analysis used to estimate the effect of assignment to intervention? → Yes
      The ITT approach with multiple imputation for missing data is an appropriate method.
    - 2.7 Was there potential for a substantial impact of the failure to analyse participants in the group to which they were randomized? → No
      All randomised participants were included in the primary outcome analysis, minimizing any potential impact.
- Risk Assessment: Low risk
- Reasoning:
    - The study used an ITT analysis, ensuring that participants were analyzed as randomized despite some dropouts, with missing data addressed via multiple imputation.
    - Participants were effectively blinded, and although personnel delivering the intervention were aware of group allocation, there is no evidence that this awareness led to trial-specific deviations.
    - The slight decline in adherence observed over time is consistent across both groups and does not appear to have materially influenced the primary outcome.
    - Given that deviations (if any) were balanced and the analysis appropriately maintained the randomized groups, following the RoB 2.0 decision rules, the overall risk of bias for Domain 2 is judged as Low risk.

Domain 3: Risk of Bias Due to Missing Outcome Data

- Signaling Question Analysis:
    - 3.1 Were data for this outcome available for all, or nearly all, participants randomized? → N
      At the 12-week primary endpoint, 13 out of 100 participants (≈13%) were dropouts, meaning that outcome data were available for roughly 87% of participants, which is below the ≥95% threshold.
    - 3.2 Is there evidence that the result was not biased by missing outcome data? → Y
      The study reports that complete case analysis produced similar results to the imputed dataset, indicating that missing data did not meaningfully bias the outcome estimates.

    - 3.3 Could missingness in the outcome depend on its true value? → No
      There is no clear evidence that the reasons for dropout were related to the participants' true pain outcomes, and similar findings between imputed and complete case analyses further support this view.
    - 3.4 Is it likely that missingness in the outcome depended on its true value? → No
      Given the evidence from sensitivity analyses, it is unlikely that the missing outcome data depended on the true values.
- Risk Assessment: Low risk of bias
- Reasoning:
    - Although outcome data were not available for all participants (only about 87% complete), robust methods were used to address missing data (multiple imputation) and sensitivity analyses (complete case analysis) showed similar results.
    - This evidence strongly suggests that the missing data did not introduce bias into the estimation of the primary outcome.
    - Following the RoB 2.0 decision rules—despite some missing data, the clear evidence (3.2 = Y) that the results were not biased leads us to conclude that the overall risk of bias due to missing outcome data is Low.

Domain 4: Risk of Bias in Measurement of the Outcome

- Signaling Question Analysis:
    - 4.1 Was the method of measuring the outcome inappropriate?→ No
      The primary outcome was measured using the Foot Health Status Questionnaire (FHSQ) pain domain, an established and validated instrument for assessing foot pain.
    - 4.2 Could measurement or ascertainment of the outcome have differed between intervention groups? → No
      The same measurement instrument was applied uniformly to both groups, ensuring consistency in outcome ascertainment.
    - 4.3 Were outcome assessors aware of the intervention received by study participants? → No
      Outcome assessors (those collecting and analyzing the FHSQ data) were blinded to group allocation.
    - 4.4 Could assessment of the outcome have been influenced by knowledge of intervention received? → No
      Blinding of participants and outcome assessors minimizes the potential for bias in self-reported outcomes such as the FHSQ pain domain.
    - 4.5 Is it likely that assessment of the outcome was influenced by knowledge of intervention received? → No
      There is no indication that knowledge of the intervention affected the outcome measurement.
- Risk Assessment: Low risk
- Reasoning:
    - The measurement method (FHSQ pain domain) is appropriate and validated.
    - Consistent application of the measurement across both groups (4.2 = No) ensures comparability.
    - Blinding of outcome assessors (4.3 = No) and lack of evidence for influence from intervention knowledge (4.4 and 4.5 = No) further support that the measurement process was not biased.
    - Following the RoB 2.0 decision rules, since none of the signaling questions indicate potential bias, the overall risk of bias in the measurement of the outcome is Low risk.

Domain 5: Risk of Bias in Selection of the Reported Result

- Signaling Question Analysis:

- 5.1 Were the data that produced this result analysed in accordance with a pre-specified analysis plan? → Probably yes
  The trial was prospectively registered and the full trial protocol was published, indicating that a pre-specified analysis plan was in place prior to unblinding, even though explicit confirmation of finalization before unblinding is not detailed.
- 5.2 Is the numerical result likely to have been selected from multiple eligible outcome measurements? → No
  The primary outcome (FHSQ pain domain at 12 weeks) was clearly pre-specified, and the analysis focused on this outcome, suggesting that selective reporting based on multiple measurements did not occur.
- 5.3 Is the numerical result likely to have been selected from multiple eligible analyses of the data? → No
  The analysis was conducted according to the pre-specified plan (using ITT with multiple imputation) and complete case analyses produced similar results, providing no evidence of selective analysis reporting.

- Risk Assessment: Low risk of bias
- Reasoning:
  - With 5.1 rated as "Probably yes" (indicating adherence to a pre-specified analysis plan), and both 5.2 and 5.3 rated as "No" (indicating no evidence of selective outcome measurement or analysis), the decision rules direct us to classify this domain as having Low risk of bias.
  - There is no indication of selective reporting or deviation from the pre-specified protocol that would compromise the reported numerical result.

ID: Lebares 2018

First Response

Domain 1: Bias Arising from the Randomization Process

- Signaling Question Analysis:
  - Was the allocation sequence random? → Y
    The study states that participants were randomized using third-party block randomization, providing explicit evidence of randomization.
  - Was the allocation sequence concealed? → PY
    The use of third-party block randomization strongly suggests proper concealment, though the report does not explicitly detail concealment methods.
  - Did baseline differences suggest a problem with randomization? → N
    Baseline characteristics (e.g., age and gender distributions) show no substantial imbalances between groups.
- Risk Assessment: LOW risk
- Reasoning:
  - The allocation sequence was explicitly randomized (Y).
  - The allocation concealment is supported by the use of a third-party block randomization procedure (PY).
  - Baseline differences do not suggest any imbalance (N).
  - According to the RoB 2.0 decision rule—if allocation concealment is in {Y, PY}, randomization is in {Y, PY, NI}, and baseline imbalances are in {N, PN, NI}—the overall risk of bias for this domain is LOW risk.
  - No inconsistencies or errors were detected in this logical assessment.

Domain 2: Bias Due to Deviations from Intended Interventions

- Signaling Question Analysis (Step 1: Analysis Type):

- Analysis Type Determination:
  The report does not state an "intention-to-treat" (ITT) analysis. One participant randomized to the control group mistakenly attended the intervention class and was subsequently re-assigned. Because this re-assignment (a protocol deviation not due to missing outcome data) was handled by analyzing according to the treatment received, the analysis is effectively "per-protocol."
- Signaling Question Analysis (Step 3: Effect of Adhering to Intervention – Applicable for PP Analysis):
  - 2.1 Were participants aware of their assigned intervention during the trial? → Yes
    In a behavioral intervention such as this, participants clearly know whether they are receiving formal mindfulness training or the active control (shared reading/discussion).
  - 2.2 Were carers and people delivering the interventions aware of participants' assigned intervention during the trial? → Yes
    Intervention providers (e.g., the MBSR instructor and control facilitators) were necessarily aware of which intervention was delivered.
  - 2.3 Were important non-protocol interventions balanced between intervention groups? → Yes
    There is no evidence of additional, non-protocol interventions; both groups received their respective active interventions as planned.
  - 2.4 Were there failures in implementing the intervention that could have affected the outcome? → No
    Apart from the one misallocation, the delivery of the intervention (class attendance, daily practice, etc.) was as intended.
  - 2.5 Was there non-adherence to the assigned intervention regimen that could have affected outcomes? → No
    Adherence was high, with similar attendance and practice patterns reported; deviations beyond the noted misallocation were minimal.
  - 2.6 Was an appropriate analysis used to estimate the effect of adhering to intervention?
    Because implementation and adherence were robust (i.e., 2.4 and 2.5 are "No"), the lack of a specialized adjustment method is less concerning. The single misallocation is unlikely to have a substantial impact on the outcome.
- Risk Assessment: LOW risk
- Reasoning:
  - Analysis Type: The study did not declare ITT and instead re-assigned one misallocated participant—this constitutes a per-protocol (PP) analysis.
  - Participant & Provider Awareness: In this behavioral trial, both participants and intervention providers were aware of the assignment. While awareness can open the door for deviations, it is expected in such trials.
  - Deviations & Their Impact:
    - The only notable deviation was the one participant misallocation—a protocol error that was managed by re-assignment.
    - There is no evidence that other deviations (e.g., failures in implementation or non-adherence) occurred to an extent that could impact the outcome (Depression immediately post-intervention).
    - Non-protocol interventions were either absent or balanced across groups.
  - Appropriate Analysis for Effect of Adhering: Although a PP (as-treated) analysis was used rather than a strict ITT approach, the minimal and isolated nature of the deviation means that any potential bias from non-adherence is negligible.

  - Conclusion: Given that all key indicators of deviations (implementation failures and non-adherence) are low and the misallocation was an isolated event, the effect of adhering to intervention is unlikely to bias the outcome. Thus, under the predefined criteria for PP analysis in Domain 2, the overall risk of bias is judged to be LOW risk.

Domain 3: Risk of Bias Due to Missing Outcome Data

- Signaling Question Analysis:
  - 3.1 Were data for this outcome available for all, or nearly all, participants randomized? → Y
    The report clearly states that there was no attrition from either the control or intervention group, meaning outcome data were obtained for all randomized participants.
  - 3.2 Is there evidence that the result was not biased by missing outcome data? → Y
    Because there were no missing data, there is clear evidence that the result was not biased by missing outcome data.
  - 3.3 Could missingness in the outcome depend on its true value? → No
    Since no outcome data are missing, missingness cannot be related to the true values.
  - 3.4 Is it likely that missingness in the outcome depended on its true value? → No
    With complete data available, there is no likelihood that missingness depended on the true outcome values.
- Risk Assessment: LOW risk
- Reasoning:
  - Completeness of Outcome Data: All randomized participants had outcome data available, satisfying the criterion for a “Yes” in 3.1.
  - Evidence Against Bias from Missing Data: With complete data, there is strong evidence that missing outcome data could not have biased the result (3.2 = Yes).
  - Dependency on True Outcome Values: Since there is no missing data, the possibility that missingness was related to the true outcome is ruled out (3.3 and 3.4 = No).
  - Conclusion: According to the predefined rules, when outcome data are available for all or nearly all participants (3.1 = Y), the risk of bias due to missing outcome data is judged to be LOW risk.

Domain 4: Risk of Bias in Measurement of the Outcome

- Signaling Question Analysis:
  - 4.1 Was the method of measuring the outcome inappropriate? → No information
    The study does not provide details on the instrument or method used to measure depression immediately post-intervention, so we cannot confirm whether a validated method was applied.
  - 4.2 Could measurement or ascertainment of the outcome have differed between intervention groups? → No
    There is no indication that different methods were used across groups; it is assumed that the same (albeit unspecified) instrument would be used for all participants.
  - 4.3 Were outcome assessors aware of the intervention received by study participants? → Yes
    Given that the depression outcome is most likely self-reported by the participants—and participants were aware of their intervention assignment—the outcome assessors (i.e., the participants themselves) were aware of the intervention received.

- 4.4 Could assessment of the outcome have been influenced by knowledge of intervention received? → Yes
  The self-reported nature of the depression measure means that participants' responses could be influenced by their knowledge of their group assignment.
- 4.5 Is it likely that assessment of the outcome was influenced by knowledge of intervention received? → Yes
  It is likely that participants' awareness of their treatment could bias their responses, for example, through expectancy effects.

- Risk Assessment: HIGH risk
- Reasoning:
  - Measurement Method (4.1): With no details provided (NI) about the instrument used to measure depression, we cannot confirm that a robust, validated method was employed.
  - Measurement Consistency (4.2): Although the same method is presumed to be used across both groups (answer: No difference), the lack of explicit information leaves uncertainty.
  - Outcome Assessor Awareness (4.3): Participants, as self-reporters of depression, are clearly aware of their intervention assignment.
  - Potential and Likelihood of Influence (4.4 & 4.5): Because the outcome is subjective and self-reported, there is a high likelihood that responses could be influenced by knowledge of the intervention received.
  - Decision Process: Following the RoB 2.0 decision rules, the awareness of the intervention by outcome assessors—combined with the likely influence on the assessment—leads to a classification of HIGH risk of bias in the measurement of the outcome.

Domain 5: Risk of Bias in Selection of the Reported Result

- Signaling Question Analysis:
  - 5.1 Were the data that produced this result analysed in accordance with a pre-specified analysis plan? → Probably yes
    The study was registered (ClinicalTrials.gov identifier: NCT03141190) and a protocol (Supplement 1) was available, suggesting that a pre-specified analysis plan likely existed, although explicit confirmation that it was finalized before unblinded data were available is not detailed.
  - 5.2 Is the numerical result being assessed likely to have been selected from multiple eligible outcome measurements? → Probably no
    There is no evidence that the depression outcome (assessed immediately post-intervention) was measured by multiple instruments or that selective reporting occurred among several eligible measures.
  - 5.3 Is the numerical result being assessed likely to have been selected from multiple eligible analyses of the data? → No
    The statistical analysis is reported using standard methods (e.g., independent t tests) and there is no indication that multiple analyses were performed with selective reporting of favorable results.
- Risk Assessment: LOW risk
- Reasoning:
  - The potential for selective reporting from multiple outcome measurements (5.2) and multiple analyses (5.3) is not supported by evidence—the responses of "Probably no" and "No" indicate that all eligible results appear to have been reported as per protocol.
  - The analysis appears to have been conducted in accordance with a pre-specified plan (5.1 = Probably yes), as implied by the trial registration and available protocol details.

- According to the decision rules: if both the risk of multiple outcome measurements and multiple analyses are in {N, PN} and the trial was analyzed per a pre-specified plan in {Y, PY}, then the overall risk of bias in the selection of the reported result is LOW risk.
- No discrepancies or selective reporting practices were identified based on the information provided.

ID: Lebares 2018

Second Response

Domain 1: Bias Arising from the Randomization Process

- Signaling Question Analysis:
  - Was the allocation sequence random? → Yes
    The study explicitly states that participants were randomized using third-party block randomization.
  - Was the allocation sequence concealed? → Yes
    Third-party block randomization implies that the allocation was conducted independently, ensuring proper concealment.
  - Did baseline differences suggest a problem with randomization? → No
    Baseline characteristics were balanced (e.g., similar age ranges and gender distributions), indicating no substantial imbalances.
- Risk Assessment: Low Risk
- Reasoning:
  - The allocation sequence was generated randomly (Yes) and concealed (Yes), as evidenced by the use of third-party block randomization.
  - There were no substantial baseline imbalances (No).
  - According to the predefined RoB 2.0 decision rule—if allocation is concealed in {Y, PY}, the sequence is random in {Y, PY, NI}, and baseline differences are in {N, PN, NI}—the overall risk is determined as Low Risk.

Domain 2: Bias Due to Deviations from Intended Interventions

- Step 1: Analysis Type Evaluation (ITT vs. PP)
  - Analysis Type:
    - The manuscript does not explicitly state an intention-to-treat (ITT) analysis. One participant randomized to the control group attended the active intervention and was subsequently re-assigned to that group.
    - Interpretation: This re-assignment indicates that the analysis was conducted on a per-protocol (or modified ITT) basis rather than strictly following the randomization.
- Step 3: Effect of Adhering to Intervention (for PP Analysis)
  - 2.1 Were participants aware of their assigned intervention during the trial? → Yes
    - Given the nature of the behavioral interventions (mindfulness training vs. an active control), participants clearly knew which intervention they received.
  - 2.2 Were carers and people delivering the interventions aware of participants' assigned intervention during the trial? → Yes
    - Instructors and intervention providers were necessarily aware of which program they were delivering.

- 2.3 Were important non-protocol interventions balanced across groups? → Yes
  - There is no indication that participants engaged in additional, non-protocol activities in an imbalanced way; both groups received their designated intervention with similar opportunities (e.g., similar class formats and scheduling).
- 2.4 Were there failures in implementing the intervention that could have affected the outcome? → No
  - All planned sessions were delivered as scheduled (no cancellations or truncations), and there was no attrition from either group.
- 2.5 Was there non-adherence to the assigned intervention regimen that could have affected participants' outcomes? → No
  - Apart from the single case of re-assignment (which was noted and managed), adherence was high and comparable between groups.
- 2.6 Was an appropriate analysis used to estimate the effect of adhering to the intervention?
  - Because there were no notable implementation failures or non-adherence issues that affected the outcome, and because the single re-assignment did not lead to systematic imbalance in adherence, further adjustment (e.g., via specialized statistical methods) was not necessitated.

- Risk Assessment:
  - Overall Judgment for Domain 2: Low Risk
- Reasoning:
  - Although the analysis deviated from a strict ITT approach—evidenced by the re-assignment of one participant (indicating a per-protocol approach)—this deviation was isolated and did not reflect systematic non-adherence or implementation failure.
  - Both participants and intervention providers were aware of the treatment assignment, which is typical in behavioral interventions; however, no trial-specific factors (such as differential implementation or imbalanced non-protocol care) emerged that could have influenced the outcome.
  - High adherence rates, full delivery of the planned sessions, and balanced exposure between groups support the conclusion that deviations from the intended intervention were minimal and unlikely to have biased the primary outcome (Depression immediately post-intervention).
- Thus, despite the departure from a strict ITT analysis, the deviations from intended interventions did not appear to influence the outcome, and the risk of bias for this domain is judged as Low Risk.

Domain 3: Risk of Bias Due to Missing Outcome Data

- Signaling Question Analysis:
  - 3.1 Were data for this outcome available for all, or nearly all, participants randomized? → Yes
    The study explicitly reports that there was no attrition from either the control or intervention group, ensuring that nearly all (in this case, all) participants provided outcome data.
  - 3.2 Is there evidence that the result was not biased by missing outcome data? → Yes
    Since outcome data were available for all participants, there is clear evidence that missing data did not bias the result.
  - 3.3 Could missingness in the outcome depend on its true value? → No
    With complete outcome data, there is no indication that any missingness could be related to the true outcome values.

- 3.4 Is it likely that missingness in the outcome depended on its true value? → No
  The absence of missing data precludes any concern regarding a dependency between missingness and the true outcome.

- Risk Assessment: Low Risk
- Reasoning:
  - Outcome data for the primary outcome (Depression immediately post-intervention) were available for all randomized participants (3.1 = Yes).
  - There is clear evidence that the results were not biased by missing data (3.2 = Yes).
  - There is no indication that missingness was related to the true outcome values (3.3 and 3.4 = No).
  - According to the predefined RoB 2.0 criteria, with complete or nearly complete outcome data and no bias from missing data, the overall risk of bias for this domain is Low Risk.

Domain 4: Risk of Bias in Measurement of the Outcome

- Signaling Question Analysis:
  - 4.1 Was the method of measuring the outcome inappropriate? → No
    Assuming that a validated self-report instrument (e.g., a standardized depression scale) was used, the measurement method is appropriate.
  - 4.2 Could measurement or ascertainment of the outcome have differed between intervention groups? → No
    Both intervention groups likely used the same method to assess depression, ensuring measurement consistency.
  - 4.3 Were outcome assessors aware of the intervention received by study participants? → Yes
    Since depression is self-reported, participants (who serve as their own outcome assessors) were aware of their assigned intervention.
  - 4.4 Could assessment of the outcome have been influenced by knowledge of intervention received? → Yes
    Participants' awareness of their intervention could bias their self-reporting of depression symptoms (e.g., due to expectations or placebo effects).
  - 4.5 Is it likely that assessment of the outcome was influenced by knowledge of intervention received? → Yes
    Given the unblinded nature of self-report in behavioral trials, it is likely that the assessment was influenced by this knowledge.
- Risk Assessment: High Risk
- Reasoning:
  - Although the method of measuring depression was appropriate (validated instrument assumed), the key concern arises from the fact that the outcome is self-reported by participants who were aware of their intervention assignment.
  - Measurement procedures were consistent across groups; however, because the participants (the outcome assessors) were not blinded, their responses could be biased by expectations or other psychological factors.
  - According to the predefined RoB 2.0 decision rules, when outcome assessors are aware of the intervention and this awareness could have influenced the assessment—as indicated by affirmative responses for both 4.4 and 4.5—the overall risk of bias in measurement is judged as High Risk.

Domain 5: Risk of Bias in Selection of the Reported Result

- Signaling Question Analysis:

- 5.1 Were the data analyzed in accordance with a pre-specified analysis plan finalized before unblinding? → Probably yes
  The trial was registered (ClinicalTrials.gov NCT03141190) and the protocol was made available (Supplement 1), indicating that a pre-specified analysis plan likely existed even though explicit confirmation of finalization prior to unblinding is not detailed.
- 5.2 Is the numerical result likely to have been selected from multiple eligible outcome measurements? → No
  There is no evidence suggesting that the depression outcome (the primary outcome for our assessment) was selectively chosen among various scales, definitions, or time points. The outcome appears to have been reported as specified in the protocol.
- 5.3 Is the numerical result likely to have been selected from multiple eligible analyses of the data? → No
  There is no indication that selective reporting of analyses occurred; the analyses appear to follow the pre-specified plan without deviations favoring particular results.

- Risk Assessment: Low Risk
- Reasoning:
  - The trial registration and availability of a protocol suggest that a pre-specified analysis plan existed (5.1 = Probably yes).
  - There is no evidence that the outcome data were selectively chosen among multiple eligible measurements (5.2 = No) or that multiple analyses were selectively reported (5.3 = No).
  - According to the predefined decision rules, when both multiple outcome measurements and analyses are not subject to selective reporting and the trial was analyzed in accordance with a pre-specified plan (in {Y, PY}), the overall risk of bias for this domain is judged as Low Risk.

ID: Maher 2017

First Response

Domain 1: Bias Arising from the Randomization Process

- Signaling Question Analysis:
  - Was the allocation sequence random? → Y
    The study reports that cases were "allocated randomly into three treatment groups according to a trial sequence determined via a computer generated number list," which is explicit evidence of randomization. citeturn0file0
  - Was the allocation sequence concealed? → Y
    The allocation was concealed using opaque sealed envelopes prepared by the research officer, ensuring secure concealment.citeturn0file0
  - Did baseline differences suggest a problem with randomization? → N
    Baseline characteristics were comparable across the groups, with no significant differences observed ($P > 0.05$). citeturn0file0
- Risk Assessment: LOW risk
- Reasoning:
  - The randomization process was explicitly performed using a computer-generated number list, fulfilling the requirement for a 'Yes' in SQ1.1.
  - Allocation concealment was ensured by using opaque sealed envelopes, meriting a 'Yes' in SQ1.2.
  - There were no significant baseline imbalances across the treatment groups, leading to a 'No' in SQ1.3.
  - According to the RoB 2.0 decision rules, when the allocation sequence is random (Y), properly concealed (Y), and there are no baseline imbalances (N), the overall risk of bias in this domain is classified as LOW risk.

Domain 2: Bias Due to Deviations from Intended Interventions

- Text Extraction & ITT/PP Evaluation:
  - The study explicitly states "Analysis was by intention to treat," indicating that all randomized participants were analyzed in the groups to which they were allocated.
- Step 2: Effect of Assignment to Intervention
  - SQ2.1: Were participants aware of their assigned intervention during the trial? → Yes
    Because the interventions were administered via clearly different routes (intraumbilical, intravenous, sublingual), participants were likely aware of their assignment.
  - SQ2.2: Were carers and people delivering the interventions aware of participants' assigned intervention during the trial? → Yes
    The investigator administering the drug was explicitly noted as being aware of the group allocation.
  - SQ2.3: Did deviations from the intended intervention arise because of the trial context? → Yes
    There were protocol deviations such as catheter insertion failure, cord avulsion, incomplete dosing, and vomiting that reflect technical or procedural issues within the trial context.
  - SQ2.4: Were these deviations likely to have affected the outcome? → Yes
    Such deviations could influence the effective delivery of the intervention and thus impact the primary outcome (need for manual removal of the placenta).
  - SQ2.5: Were these deviations from intended intervention balanced between groups? → Yes
    The frequency of such deviations appears similar across the treatment groups.
  - SQ2.6: Was an appropriate analysis used to estimate the effect of assignment to intervention? → Yes
    The use of an intention-to-treat analysis is appropriate as it includes all randomized participants regardless of deviations.
  - SQ2.7: Was there potential for a substantial impact (on the result) of the failure to analyse participants in the group to which they were randomized? → No
    The deviations, while present, affected only a small proportion of participants and are unlikely to have had a substantial impact on the results.
- Risk of Bias Judgement:
  - Part 1 (Effect of Assignment):
    - Because both participants and carers were aware (SQ2.1 & SQ2.2: Yes) and deviations arose due to trial context (SQ2.3: Yes) that likely affected the outcome (SQ2.4: Yes), yet these deviations were balanced across groups (SQ2.5: Yes), Part 1 is rated as SOME CONCERNS.
  - Part 2 (Appropriateness of Analysis):
    - The study used an intention-to-treat analysis (SQ2.6: Yes) and the impact of exclusions appears minimal (SQ2.7: No), leading to a LOW risk rating for Part 2.
  - Overall Domain 2 Risk:
    - With Part 1 rated as SOME CONCERNS and Part 2 as LOW risk, the overall risk for this domain is SOME CONCERNS.
- Reasoning:
  Although the study appropriately used an intention-to-treat analysis to mitigate bias from deviations, the lack of blinding (with both participants and carers aware of the intervention) and the occurrence of technical deviations (e.g., failed catheter insertion, cord avulsion) that likely impacted the effective administration of the intervention introduce some risk

of bias. These deviations were balanced across groups; however, their potential effect on the primary outcome leads to an overall judgment of SOME CONCERNS for Domain 2.

Domain 3: Risk of Bias Due to Missing Outcome Data

- Signaling Question Analysis:
  - 3.1 Were data for this outcome available for all, or nearly all, participants randomized? → Y
    Outcome data for the primary outcome (manual removal of placenta) were reported for all participants in the three groups (96, 94, and 91), meeting the “all or nearly all” criterion.citeturn0file0
  - 3.2 Is there evidence that the result was not biased by missing outcome data? → Y
    The complete reporting of outcomes and the intention-to-treat analysis indicate that missing data did not bias the results.citeturn0file0
  - 3.3 Could missingness in the outcome depend on its true value? → N
    With nearly complete data, missingness is minimal and is not likely related to the actual outcome values. citeturn0file0
  - 3.4 Is it likely that missingness in the outcome depended on its true value? → N
    Given the negligible amount of missing data, it is unlikely that any missingness depended on the true outcome value. citeturn0file0
- Risk Assessment: LOW risk of bias
- Reasoning:
  - Since outcome data were available for all participants (SQ3.1 = Y) and there is clear evidence that missing data did not introduce bias (SQ3.2 = Y), the potential for bias due to missing outcome data is minimal.
  - Additionally, there is no indication that missingness could depend on the true outcome (SQ3.3 and SQ3.4 = N).
  - According to the predefined RoB 2.0 decision rules, these responses collectively indicate a LOW risk of bias for Domain 3.

Domain 4: Risk of Bias in Measurement of the Outcome

- Signaling Question Analysis:
  - SQ4.1: Was the method of measuring the outcome inappropriate? → No
    The outcome—whether the placenta was delivered spontaneously within 30 minutes or if MROP was required—is measured using clear, objective criteria and a standardized protocol.
  - SQ4.2: Could measurement or ascertainment of the outcome have differed between intervention groups? → No
    The measurement method was consistent across all groups, with the same timing and clinical criteria applied to each.
  - SQ4.3: Were outcome assessors aware of the intervention received by study participants? → Yes
    Given the differing routes of administration (intraumbilical, intravenous, sublingual), it is evident that outcome assessors were aware of the intervention assignments.
  - SQ4.4: Could assessment of the outcome have been influenced by knowledge of intervention received? → No
    Despite the lack of blinding, the objective and protocol-driven nature of the outcome (timed placental delivery and subsequent decision for MROP) minimizes the potential for assessor influence.
  - SQ4.5: Is it likely that assessment of the outcome was influenced by knowledge of intervention received? → No
    The strict criteria for intervention (i.e., performing MROP after 30 minutes or severe bleeding) ensure that the outcome is determined objectively, making it unlikely that assessor knowledge introduced bias.

- Risk Assessment: LOW risk
- Reasoning:
  The measurement method for the primary outcome is appropriate and consistently applied across all groups. Although outcome assessors were aware of the intervention assignments, the outcome itself is objectively defined (placental expulsion within a strict 30-minute window or the need for MROP based on clear clinical criteria). This minimizes the risk that assessor awareness could influence the measurement. Therefore, following the RoB 2.0 decision rules, Domain 4 is judged as LOW risk.

Domain 5: Risk of Bias in Selection of the Reported Result

- Signaling Question Analysis:
  - SQ5.1: Were the data that produced this result analysed in accordance with a pre-specified analysis plan? → Probably yes
    The study was registered (ISRCTN10193593), and the methods indicate that an intention-to-treat analysis was employed. Although explicit confirmation that the analysis plan was finalized before unblinding is not detailed, the registration and adherence to CONSORT guidelines imply that a pre-specified plan likely existed.
  - SQ5.2: Is the numerical result being assessed likely to have been selected from multiple eligible outcome measurements?→ No
    The primary outcome (manual removal of the placenta) is clearly defined, and there is no evidence that multiple measurements or definitions were selectively reported.
  - SQ5.3: Is the numerical result being assessed likely to have been selected from multiple eligible analyses of the data? → No
    The analysis is consistently reported (intention-to-treat), with no indication of selective reporting among multiple analyses.
- Risk Assessment: LOW risk of bias
- Reasoning:
  - With both multiple outcome measurements (SQ5.2) and multiple analyses of data (SQ5.3) rated as No—indicating that all eligible results appear to have been reported—and with the trial being analyzed in accordance with a pre-specified plan (SQ5.1 = Probably yes), the predefined RoB 2.0 decision rules lead to a LOW risk of bias judgment for Domain 5.
  - There are no indications of selective outcome or analysis reporting based on the available study details.
- Thus, for Domain 5, the risk of bias in the selection of the reported result is LOW risk.

ID: Maher 2017

Second Response

Domain 1: Bias Arising from the Randomization Process

- Signaling Question Analysis:
  - Was the allocation sequence random? → Y
    The study explicitly states that a computer-generated number list was used to randomly allocate participants.
  - Was the allocation sequence concealed? → Y
    The allocation was concealed using opaque, sequenced envelopes prepared by a medical research officer, with all authors blinded to the envelope contents.

  - Did baseline differences suggest a problem with randomization? → N
    Baseline characteristics (Table 1) show no significant differences between groups.
- Risk Assessment: Low
- Reasoning:
  The study clearly reports that randomization was implemented using a computer-generated number list (Y) and that allocation concealment was secured with opaque envelopes (Y). Moreover, there were no substantial baseline imbalances (N). According to RoB 2.0 decision rule 3, when allocation sequence concealment is confirmed (Y), the allocation sequence is random (Y), and baseline differences do not indicate any problem (N), the risk of bias in this domain is judged as Low Risk.

Domain 2: Bias Due to Deviations from Intended Interventions

- Step 1: Analysis Type
  - The study explicitly states "analysis was by intention to treat."
    → Classification: ITT
- Step 2: Effect of Assignment to Intervention
  - 2.1 Were participants aware of their assigned intervention? → Yes
    Because the interventions differ in route (intraumbilical, intravenous, sublingual) and no explicit blinding of participants is reported, it is clear that participants were aware of their treatment assignment.
  - 2.2 Were carers and people delivering the interventions aware of participants' assigned intervention? → Yes
    The study explicitly notes that the investigator administering the drug was aware of the group allocation.
  - 2.3 Were there deviations from the intended intervention that arose because of the trial context? → No
    While technical issues (e.g., catheter failure, cord avulsion, vomiting) occurred, these reflect real-world difficulties inherent to the intervention routes rather than trial-specific deviations.
  - 2.4 Were these deviations likely to have affected the outcome? → No
    The minor technical issues did not substantially alter the primary outcome (need for manual removal of the placenta) and were relatively few compared to overall numbers.
  - 2.5 Were these deviations balanced between groups? → Yes
    The incidences of technical issues appear similar across all intervention arms.
  - 2.6 Was an appropriate analysis used to estimate the effect of assignment to intervention? → Yes
    An intention-to-treat analysis was performed, ensuring that all randomized participants were included regardless of deviations.
  - 2.7 Was there potential for a substantial impact of the failure to analyze participants in the groups to which they were randomized?→ No
    The small number of deviations did not materially affect the outcome, and all participants were analyzed as randomized.
- Risk Assessment Calculation:
  - Part 1 (Effect of Assignment):
    Since participants (2.1) and personnel (2.2) were aware (Y) but deviations from the intended intervention were absent (2.3 = No) and did not affect the outcome (2.4 = No), Part 1 is set to LOW risk.
  - Part 2 (Appropriateness of Analysis):
    An ITT approach was used (2.6 = Yes) and there is no substantial impact from exclusions (2.7 = No), so Part 2 is set to LOW risk.

- Final Judgment:
  Since both Part 1 and Part 2 are LOW risk, the overall risk of bias for this domain is Low Risk.
- Reasoning:
  Although both participants and carers were aware of the treatment assignments (due to the differing administration routes), there is no evidence that these awareness factors led to deviations from the intended interventions that arose from the trial context.

Domain 3: Bias Due to Missing Outcome Data

- Signaling Question Analysis:
  - Were data for this outcome available for all, or nearly all, participants randomized? → Y
    Outcome data were reported for all 281 randomized participants, with complete outcome information provided in Table 2.
  - Is there evidence that the result was not biased by missing outcome data? → Y
    The analysis was performed on an intention-to-treat basis, and no indication of missing outcome data was given.
  - Could missingness in the outcome depend on its true value? → N
    There is no indication of missing outcome data, making dependency on the true value highly unlikely.
  - Is it likely that missingness in the outcome depended on its true value? → N
    Given the near-complete outcome data, it is unlikely that missingness (if any) was related to the true outcome.
- Risk Assessment: Low Risk
- Reasoning:
  The study provided complete outcome data for the primary outcome (manual removal of the placenta) for nearly all (in fact, all) randomized participants. With an intention-to-treat analysis and no evidence of missing data that could bias the results, the conditions for a low risk of bias are fully met according to the RoB 2.0 criteria.

Domain 4: Bias in Measurement of the Outcome

- Signaling Question Analysis:
  - Was the method of measuring the outcome inappropriate? → No
    The primary outcome (manual removal of the placenta) was determined by clear, protocol-defined criteria (e.g., failure of placental delivery within 30 minutes or severe bleeding), representing an appropriate and objective method of measurement.
  - Could measurement or ascertainment of the outcome have differed between intervention groups? → No
    The method for assessing whether manual removal was needed was applied uniformly across all treatment groups.
  - Were outcome assessors aware of the intervention received by study participants? → Probably yes
    There is some indication that the clinicians administering the intervention were aware of group allocation; however, the outcome (need for manual removal) is defined by objective criteria, even if the assessors were likely aware.
  - Could assessment of the outcome have been influenced by knowledge of intervention received? → No
    The objective nature of the outcome and the clear threshold (30 minutes without placental delivery or the occurrence of severe bleeding) make it unlikely that awareness of intervention would meaningfully influence the decision to perform manual removal.

    - Is it likely that assessment of the outcome was influenced by knowledge of intervention received? → No
      Given the standardized protocol for determining the need for manual removal, any potential influence is minimal.
- Risk Assessment: Low Risk
- Reasoning:
  The outcome was measured using a clearly defined, objective, and standardized method that applied equally across all intervention groups. Although outcome assessors were probably aware of the treatment assignment, the strictly protocol-driven decision for manual removal of the placenta minimizes the potential for subjective influence. According to the RoB 2.0 decision rules—specifically following branch 2.3.2.1—the risk of bias in measurement of the outcome is judged as Low Risk.

Domain 5: Bias in Selection of the Reported Result

- Signaling Question Analysis:
    - 5.1: Were the data that produced this result analysed in accordance with a pre-specified analysis plan? → Probably yes
      The trial was registered (ISRCTN10193593) and the methods indicate an intention-to-treat analysis was planned, suggesting that a pre-specified analysis plan was in place prior to unblinded data access.
    - 5.2: Is the numerical result being assessed likely to have been selected, on the basis of the results, from multiple eligible outcome measurements within the outcome domain? → No
      The primary outcome—manual removal of the placenta following failure of placental delivery within 30 minutes—is clearly defined and consistently reported, with no evidence of multiple competing measurements.
    - 5.3: Is the numerical result being assessed likely to have been selected, on the basis of the results, from multiple eligible analyses of the data? → No
      The analysis appears to follow the pre-specified plan (as indicated by trial registration and the clear description of methods), with no indication that alternative analyses were selectively reported.
- Risk Assessment: Low Risk
- Reasoning:
  Since there is evidence (via trial registration and clear methodology) suggesting that the analysis was conducted according to a pre-specified plan (indicated by a "Probably yes" for 5.1) and there is no indication of selective measurement or selective analysis (both 5.2 and 5.3 are answered as "No"), the criteria for low risk of bias are met. According to the RoB 2.0 rules, when multiple outcome measurements and analyses raise no concerns and the trial appears to have adhered to a pre-specified analysis plan, the risk of bias in the selection of the reported result is judged as Low Risk.

ID: Daher 2000

First Response

Domain 1: Bias Arising from the Randomization Process

- Signaling Question Analysis:
    - Was the allocation sequence random? → No
      Rationale: The study clearly states that treatment assignment was based on the infectologist’s predetermined practice (one group always used penicillin while the other did not), which is explicit evidence of non-random allocation.

- Was the allocation sequence concealed? → No
  Rationale: There is explicit evidence that the allocation was known to the treating infectologists, with no indication of any secure concealment measures.
- Did baseline differences suggest a problem with randomization? → Yes
  Rationale: Although most baseline characteristics were similar, the treated group exhibited significantly higher proteinuria (840±610 vs. 454±246 mg/dL, p=0.03), indicating a substantial imbalance.

- Risk Assessment: High Risk
- Reasoning:
  - The allocation sequence was determined by the treating infectologist's practice rather than by a random process (SQ 1.1 = No).
  - There is explicit evidence that the allocation was not concealed (SQ 1.2 = No), which, per Rule 1 ("If allocation sequence concealed in {N, PN}, return HIGH risk"), mandates a High Risk judgment.
  - Additionally, the presence of a significant baseline imbalance (SQ 1.3 = Yes) further supports a problematic allocation process.
  - Following the decision tree rules, the combination of non-random allocation, lack of concealment, and substantial baseline differences results in a final judgment of High Risk.

Domain 2: Bias Due to Deviations from Intended Interventions

- Analysis Type Determination:
  - The study did not explicitly state "intention-to-treat" or similar terminology. Instead, eight patients were excluded at admission (for reasons such as age >60, non-confirmed diagnosis, or early death on penicillin therapy).
  - Because exclusions were made for reasons other than solely missing outcome data (e.g., protocol-defined ineligibility and early adverse outcomes), the analysis is classified as per-protocol.
  - Consequently, we proceed with the assessment for the effect of adhering to intervention (Step 3).
- Signaling Question Analysis (Effect of Adhering to Intervention):
  - 2.1 Were participants aware of their assigned intervention during the trial? → Yes
    Rationale: There is no indication of effective blinding; patients knew whether they were treated with penicillin or not.
  - 2.2 Were carers and people delivering the interventions aware of participants' assigned intervention during the trial? → Yes
    Rationale: The treating infectologists were fully aware of the intervention choice, as treatment was based on their established practice.
  - 2.3 Were important non-protocol interventions balanced across intervention groups? → Yes
    Rationale: Both groups received similar standard supportive care (e.g., hydration, early dialysis when indicated), and there is no evidence suggesting imbalance in additional care.
  - 2.4 Were there failures in implementing the intervention that could have affected the outcome? → No
    Rationale: The penicillin regimen (6 million IU/day for 8 days) was administered as planned in the treated group without reported implementation issues.
  - 2.5 Was there non-adherence to the assigned intervention regimen that could have affected participants' outcomes? → No

Rationale: There is no indication that participants deviated from the assigned intervention; treatment was delivered per protocol.

- Risk of Bias Judgement (Effect of Adhering to Intervention):
  - Because participants and intervention providers were aware of group assignments (2.1 and 2.2 = Yes), we refer to the branch that first checks whether non-protocol interventions were balanced.
  - With balanced non-protocol interventions (2.3 = Yes), we then check for implementation failures (2.4 = No) and non-adherence (2.5 = No).
  - Since there are no failures or non-adherence issues, the decision tree indicates a LOW risk of bias regarding deviations from the intended intervention.
- Final Risk Assessment for Domain 2:
  LOW risk
- Reasoning Summary:
  - The study's analysis is per-protocol because patients were excluded for reasons beyond missing outcome data.
  - Although the trial was open label (participants and carers were aware of the intervention), both groups received similar supportive care, and there is no evidence of deviations in delivering or adhering to the intended interventions.
  - The absence of implementation failures or non-adherence issues leads to a final judgment of LOW risk for bias due to deviations from intended interventions.

Domain 3: Risk of Bias Due to Missing Outcome Data

- Signaling Question Analysis:
  - 3.1 Were data for this outcome available for all, or nearly all, participants randomized? → No
    Rationale: Out of an initial 43 patients, only 35 were included in the analysis. This means approximately 81% of the participants provided outcome data, which does not meet the "nearly all" criterion (e.g., ≥95% for continuous outcomes).
  - 3.2 Is there evidence that the result was not biased by missing outcome data? → No
    Rationale: The study excluded 8 patients (including 4 early deaths) for reasons other than missing follow-up data, which raises concern that these exclusions might have biased the outcome estimate for all-cause mortality.
  - 3.3 Could missingness in the outcome depend on its true value? → Yes
    Rationale: The excluded participants, particularly the 4 early deaths, represent cases with the worst outcomes. Their exclusion suggests that missingness is clearly related to the true outcome values.
  - 3.4 Is it likely that missingness in the outcome depended on its true value? → Yes
    Rationale: Since the patients excluded early on were those who died, it is highly likely that the missingness is dependent on the true outcome (i.e., a worse outcome would have been recorded had they been included).
- Risk Assessment: High Risk
- Reasoning:
  - Outcome data were not available for nearly all participants (SQ 3.1 = No).
  - There is no evidence presented that the missing data did not bias the results (SQ 3.2 = No).
  - The missing outcome data are clearly related to worse outcomes (SQ 3.3 = Yes) and are very likely dependent on the true outcome values (SQ 3.4 = Yes).
  - According to the decision rules, because missingness is likely related to true outcomes (with answers in the {Y, PY, NI} category for SQ 3.3 and 3.4), the final judgment must be High Risk.

Domain 4: Risk of Bias in Measurement of the Outcome

- Signaling Question Analysis:
  - 4.1 Was the method of measuring the outcome inappropriate? → No
    Rationale: The primary outcome, all-cause mortality, is objectively recorded (e.g., via hospital records) and is not subject to interpretation, so the measurement method is appropriate.
  - 4.2 Could measurement or ascertainment of the outcome have differed between intervention groups? → No
    Rationale: Mortality was ascertained in the same way across both groups, with no indication that the method differed between intervention arms.
  - 4.3 Were outcome assessors aware of the intervention received by study participants? → Yes
    Rationale: The study was conducted in an open-label manner, so it is reasonable to conclude that outcome assessors were aware of which intervention participants received.
  - 4.4 Could assessment of the outcome have been influenced by knowledge of intervention received? → No
    Rationale: Despite assessors' awareness, the outcome (mortality) is objective and is unlikely to be influenced by such knowledge.
  - 4.5 Is it likely that assessment of the outcome was influenced by knowledge of intervention received? → No
    Rationale: Given the objective nature of mortality, even if assessors knew the intervention status, this would not likely bias the outcome measurement.
- Risk Assessment:
  LOW risk
- Reasoning:
  - The method of measuring the outcome is appropriate (4.1 = No), as all-cause mortality is an objective endpoint.
  - Measurement methods were consistent across both intervention groups (4.2 = No).
  - Although outcome assessors were aware of intervention assignments (4.3 = Yes), the objectivity of the outcome (mortality) makes it unlikely that this awareness would influence the assessment (4.4 and 4.5 = No).
  - Following the decision rules—since the measurement is appropriate and consistent, and any potential influence from assessor awareness does not translate into biased outcome assessment—the final judgment for Domain 4 is LOW risk.

Domain 5: Risk of Bias in Selection of the Reported Result

- Signaling Question Analysis:
  - 5.1 Were the data that produced this result analysed in accordance with a pre-specified analysis plan finalized before unblinded outcome data were available for analysis? → No Information
    Rationale: The article does not provide explicit details regarding a pre-specified analysis plan or trial registration. There is no confirmation that the statistical analysis plan was finalized before outcome data became unblinded.
  - 5.2 Is the numerical result being assessed likely to have been selected, on the basis of the results, from multiple eligible outcome measurements (e.g., scales, definitions, time points) within the outcome domain? → No
    Rationale: The primary outcome— all-cause mortality—was reported as a single, objective measure. There is no indication that the result was selectively chosen from among several eligible outcome measurements.
  - 5.3 Is the numerical result being assessed likely to have been selected, on the basis of the results, from multiple eligible analyses of the data? → No

Rationale: The analysis appears straightforward (using Student t-test and Fisher test for comparisons) with no evidence suggesting that alternative analytical approaches were performed and selectively reported.

- Risk Assessment:
  SOME CONCERNS
- Reasoning:
  - Both the possibility of multiple outcome measurements and multiple analyses of the data were evaluated and found not to be issues (answers for 5.2 and 5.3 are "No", which falls into the {N, PN} category).
  - However, there is no information regarding whether the analysis was performed according to a pre-specified plan (5.1 = No Information), placing it in the {N, PN, NI} category.
  - Following the decision rules: When both 5.2 and 5.3 are in {N, PN} but 5.1 is in {NI}, the judgment is to return SOME CONCERNS.
  - This indicates that while there is no evidence of selective reporting of outcomes or analyses, the absence of details on a pre-specified analysis plan leaves some room for potential selective reporting bias.

Thus, the final judgment for Domain 5 is SOME CONCERNS.

ID: Daher 2000

Second Response

Domain 1: Bias Arising from the Randomization Process

- Signaling Question Analysis:
  - Was the allocation sequence random? → N
    The study assigned treatment based on the attending infectologist's choice rather than a random procedure.
  - Was the allocation sequence concealed? → NI
    No information is provided regarding the concealment of the allocation sequence.
  - Did baseline differences suggest a problem with randomization? → N
    Baseline characteristics (age, gender, laboratory data) were similar between groups.
- Risk Assessment: Some Concerns
- Reasoning:
  - Non-random allocation: The allocation sequence was not random (scored N) because treatment was determined by the attending infectologist rather than by a randomized method.
  - Lack of information on concealment: There is no explicit information regarding allocation concealment (scored NI).
  - Balanced baseline characteristics: The groups had similar baseline characteristics (scored N), reducing concerns of systematic differences between groups.
  - Decision Process:
    - According to the provided decision rules, when the allocation concealment is NI and baseline imbalances are N (or PN/NI), rule 4.1 applies, resulting in a judgment of Some Concerns.
  - Conclusion: Despite balanced baseline data, the absence of randomization and unclear concealment leads to a potential risk of bias, warranting a "Some Concerns" rating.

Domain 2: Bias Due to Deviations from Intended Interventions

- Step 1: Analysis Approach

- ITT/PP Classification:
  - The study does not report any explicit mention of "intention-to-treat" (ITT), "modified ITT" (mITT), or "per-protocol" (PP). Given the absence of such keywords and that patients were analyzed according to the treatment they received (with exclusions occurring pre-assignment), the analysis is classified as per-protocol (PP).
- Step 3: Effect of Adhering to Intervention
  (Since the study is PP, we assess the effect of adherence to the intervention.)
  - 2.1 Were participants aware of their assigned intervention during the trial? → Yes
    There is no indication of any blinding; patients likely knew whether they received penicillin.
  - 2.2 Were carers and people delivering the interventions aware of participants' assigned intervention during the trial? → Yes
    The infectologists clearly decided on treatment, indicating that those delivering the intervention were aware of the assignment.
  - 2.3 Were important non-protocol interventions balanced across intervention groups? → Yes
    All patients received standard clinical support (including early dialysis when indicated), suggesting that any additional (non-protocol) interventions were applied similarly across groups.
  - 2.4 Were there failures in implementing the intervention that could have affected the outcome? → No
    There is no evidence of deviations or failures in administering the planned treatment.
  - 2.5 Was there non-adherence to the assigned intervention regimen that could have affected participants' outcomes? → No
    No indications of non-adherence are reported; patients received the interventions as assigned.
  - 2.6 Was an appropriate analysis used to estimate the effect of adhering to intervention?
    - Not applicable here because there were no failures in implementation or non-adherence that would require adjustment.
- Final Risk Assessment for Domain 2: LOW risk
- Reasoning:
  - Despite the open-label nature of the study (with both participants and personnel aware of the assigned interventions), there is no evidence that this awareness led to deviations from the intended interventions that could affect the primary outcome (all-cause mortality).
  - Non-protocol interventions (e.g., standard clinical support, early dialysis) were balanced between the treatment and non-treatment groups.
  - The absence of implementation failures or non-adherence indicates that the per-protocol analysis did not introduce bias in estimating the effect of adhering to the intervention.
  - Therefore, according to the decision rules provided, the overall risk of bias for deviations from intended interventions is assessed as LOW risk.

Domain 3: Risk of Bias Due to Missing Outcome Data

- Signaling Question Analysis:
  - Were data for this outcome available for all, or nearly all, participants randomized? → Yes
    In the analysis, all 35 participants had complete outcome data for all-cause mortality (despite 8 pre-enrollment exclusions based on protocol criteria, the analyzed cohort has full data).

- Is there evidence that the result was not biased by missing outcome data? → Yes
  There is clear evidence that the mortality outcome was recorded for every participant in the final cohort, indicating no bias from missing data.
- Could missingness in the outcome depend on its true value? → No
  With complete mortality data in the analyzed population, there is no indication that missingness could be related to the true outcome.
- Is it likely that missingness in the outcome depended on its true value? → No
  Given the complete outcome ascertainment for the included participants, it is unlikely that missingness was related to the true outcome.

- Risk Assessment: LOW risk of bias
- Reasoning:
  - The study reports complete outcome data for the primary outcome (all-cause mortality) in the final analysis cohort, with no loss to follow-up or unrecorded data among the 35 participants.
  - Although 8 patients were excluded at admission (including early deaths), these exclusions were predetermined by protocol and not due to post-randomization missing outcome data.
  - Since the outcome data are available for nearly all participants considered in the analysis and there is clear evidence that missing data could not have biased the result, the strict decision rules lead to a LOW risk of bias assessment.

Domain 4: Risk of Bias in Measurement of the Outcome

- Signaling Question Analysis:
  - 4.1 Was the method of measuring the outcome inappropriate?→ No
    All-cause mortality is measured using objective, standard clinical records, which is an appropriate method.
  - 4.2 Could measurement or ascertainment of the outcome have differed between intervention groups? → No
    The outcome (mortality) was ascertained consistently across both intervention groups.
  - 4.3 Were outcome assessors aware of the intervention received by study participants? → Yes
    Given the open-label nature of the study, outcome assessors were aware of the intervention assignments.
  - 4.4 Could assessment of the outcome have been influenced by knowledge of intervention received? → No
    Despite assessor awareness, the objective nature of mortality minimizes the potential for influence.
  - 4.5 Is it likely that assessment of the outcome was influenced by knowledge of intervention received? → No
    Mortality is an objective endpoint unlikely to be affected by assessor knowledge.
- Risk Assessment: LOW risk of bias
- Reasoning:
  - The measurement method for all-cause mortality is clearly appropriate and objective.
  - There is no indication that the method of outcome ascertainment differed between groups.
  - Although outcome assessors were aware of the intervention, the objective nature of the mortality endpoint ensures that this awareness did not influence the measurement.
  - Thus, according to the predefined decision rules, the risk of bias in the measurement of the outcome is judged as LOW risk.

Domain 5: Risk of Bias in Selection of the Reported Result

- Signaling Question Analysis:

- 5.1 Were the data that produced this result analysed in accordance with a pre-specified analysis plan? → No information
  The study methods describe the statistical tests used (Student t test and Fisher test) but do not provide any details regarding a pre-specified analysis plan finalized before unblinded outcome data were available.
- 5.2 Is the numerical result being assessed likely to have been selected, on the basis of the results, from multiple eligible outcome measurements? → No
  The primary outcome (all-cause mortality) appears to be the clearly defined and sole outcome reported, with no evidence of selective measurement among multiple eligible scales or time points.
- 5.3 Is the numerical result being assessed likely to have been selected, on the basis of the results, from multiple eligible analyses of the data? → No
  There is no indication that multiple analyses were performed with selective reporting; the reported analyses are consistent with the methods described.

- Risk Assessment: SOME CONCERNS
- Reasoning:
  - Although there is no evidence of selective reporting regarding outcome measurements (5.2) or multiple analyses (5.3), the absence of details about whether the analysis plan was finalized before unblinding (5.1: No information) prevents a definitive judgment of low risk.
  - According to the decision rules, when both multiple outcome measurements and multiple analyses are judged as "No" but the pre-specified analysis plan is unclear (scored as No information), the overall judgment defaults to Some Concerns.

ID: Toouli 2000

First Response

Domain 1: Bias Arising from the Randomization Process

- Signaling Question Analysis:
  - 1.1 Was the allocation sequence random? → Yes
    Rationale: The study explicitly states that patients were "randomised by the draw of a card," indicating a random method was used.
  - 1.2 Was the allocation sequence concealed? → No information
    Rationale: There is no explicit mention of secure concealment (e.g., sealed envelopes or independent allocation), so concealment cannot be confirmed.
  - 1.3 Did baseline differences suggest a problem with randomization? → No
    Rationale: There is no evidence of substantial baseline imbalances reported in the study.
- Risk Assessment: Some Concerns
- Reasoning:
  - The allocation sequence was generated using a random process (drawing a card), satisfying the criteria for randomization (SQ1.1 = Yes).
  - However, the study did not provide explicit evidence regarding the concealment of the allocation sequence (SQ1.2 = NI).
  - There were no substantial baseline imbalances noted (SQ1.3 = No).
  - Based on the predefined decision rules, when allocation sequence concealment is rated as "No information" and baseline imbalances are "No," the risk of bias is determined as Some Concerns (Rule 4.1).

  - No inconsistencies were detected during the auto-error check, so no correction was necessary.

Domain 2: Bias Due to Deviations from Intended Interventions

- Analysis of Analysis Type:
  - Analysis Approach Determination:
    A search of the text does not reveal any explicit mention of "intention-to-treat" (ITT) or "modified intention-to-treat" analysis. In the Results it is stated that "two patients were excluded from analysis: one was lost to follow-up after six months and one was withdrawn because of two episodes of pancreatitis."
    - The exclusion for loss to follow-up represents missing outcome data.
    - The exclusion for pancreatitis reflects a post-randomization removal for reasons related to toxicity/adverse events rather than merely missing data.
      Interpretation: These exclusions for reasons other than solely missing outcome data indicate that the study conducted a per-protocol (PP) analysis.
- Because the analysis is classified as PP, we proceed to Step 3: Effect of Adhering to Intervention.
- Step 3: Effect of Adhering to Intervention – Signaling Question Analysis
  - 2.1 Were participants aware of their assigned intervention during the trial?
    Answer: No
    Rationale: The trial is described as a "prospective double-blind study," indicating that participants were effectively blinded.
  - 2.2 Were carers and people delivering the interventions aware of participants' assigned intervention during the trial?
    Answer: Yes
    Rationale: Although the study is double-blind regarding patients and outcome assessment, the endoscopist performing the procedure must know whether to perform a sphincterotomy or a sham procedure.
  - 2.3 Were important non-protocol interventions balanced across intervention groups?
    Answer: Yes
    Rationale: The only "extra" procedure noted was the extension of the endoscopic incision at three months in patients undergoing sphincterotomy based on manometric evidence. This step was pre-specified for ensuring an adequate incision rather than representing an unplanned or non-protocol deviation; thus, no imbalance in non-protocol interventions is evident.
  - 2.4 Were there failures in implementing the intervention that could have affected the outcome?
    Answer: Yes
    Rationale: In 19 patients the endoscopic incision was extended at three months due to evidence of incomplete division of the sphincter. This indicates that the initial intervention was not uniformly successful, potentially impacting outcomes.
  - 2.5 Was there non-adherence to the assigned intervention regimen that could have affected participants' outcomes?
    Answer: No
    Rationale: Aside from the necessary extension of the incision (which was protocol-driven), there is no indication that participants failed to receive their assigned intervention.
  - 2.6 Was an appropriate analysis used to estimate the effect of adhering to intervention?
    Answer: No

Rationale: The analysis excluded two patients post-randomization (one for loss to follow-up and one due to pancreatitis), indicating a per-protocol approach. Exclusions for reasons such as toxicity (pancreatitis) mean that the analysis does not fully preserve the benefits of randomization when estimating the effect of adhering to the intervention.

- Risk Assessment for Domain 2:
  - Step 3 Judgment:
    - Because personnel were aware of the intervention (2.2 = Yes) and we then evaluated the balance of non-protocol interventions (2.3 = Yes), we proceeded to assess implementation failures (2.4) and non-adherence (2.5).
    - The presence of implementation failures (2.4 = Yes) combined with the use of a per-protocol analysis (2.6 = No for appropriate analysis) leads us, per the decision rule (branch 2.1.2.2), to assign a HIGH risk of bias for this domain.
- Final Risk Assessment: HIGH risk
- Reasoning Summary:
  - The study did not perform a full ITT analysis; exclusions (one for missing outcome data and one due to pancreatitis) indicate a per-protocol approach.
  - Although participants were effectively blinded (2.1 = No), the intervention providers were aware of assignments (2.2 = Yes).
  - No imbalances in non-protocol interventions were detected (2.3 = Yes), but the need to extend the sphincterotomy in several patients (2.4 = Yes) signifies implementation issues that could affect outcomes.
  - The per-protocol analysis (2.6 = No) is considered inappropriate for estimating the effect of adhering to intervention when such deviations occur.
  - Following the decision tree, these factors combine to yield a HIGH risk of bias for Domain 2.

No inconsistencies with the predefined decision rules were detected, so no corrections were necessary.

Domain 3: Risk of Bias Due to Missing Outcome Data

- Signaling Question Analysis:
  - 3.1 Were data for this outcome available for all, or nearly all, participants randomized? → Yes
    Rationale: Of the 81 participants, outcome data were available for 79 (approximately 97.5%), which meets the "nearly all" criterion (≥95%).
  - 3.2 Is there evidence that the result was not biased by missing outcome data? → Yes
    Rationale: With only 2 participants missing, the amount of missing data is minimal, making it unlikely to bias the results.
  - 3.3 Could missingness in the outcome depend on its true value? → No
    Rationale: There is no evidence suggesting that the few missing cases (one lost to follow-up and one withdrawn due to pancreatitis) were systematically related to the outcome values.
  - 3.4 Is it likely that missingness in the outcome depended on its true value? → No
    Rationale: Given the very low proportion of missing data, it is unlikely that the missingness depended on the true outcomes.
- Risk Assessment: LOW risk
- Reasoning:

- Nearly all participants (97.5%) provided outcome data (SQ 3.1 = Yes), fulfilling the criterion for minimal missing data.
- The minimal level of missing data provides clear evidence that the results are unlikely to be biased (SQ 3.2 = Yes).
- There is no indication that the small amount of missing data was related to the true outcome values (SQ 3.3 = No and SQ 3.4 = No).
- According to the RoB 2.0 decision rules, when outcome data are available for nearly all participants, the risk of bias is judged as LOW risk.

No inconsistencies were detected, and no corrections were necessary.

Domain 4: Risk of Bias in Measurement of the Outcome

- Signaling Question Analysis:
  - 4.1 Was the method of measuring the outcome inappropriate?→ No
    Rationale: Outcome measurement was based on pain diaries and assessment by a blinded, independent clinician using a structured questionnaire, which is an appropriate method for evaluating abdominal pain improvement.
  - 4.2 Could measurement or ascertainment of the outcome have differed between intervention groups? → No
    Rationale: The same outcome measurement procedures were applied across all intervention groups, with no indication of differential assessment.
  - 4.3 Were outcome assessors aware of the intervention received by study participants? → No
    Rationale: The study explicitly states that the clinician who assessed outcomes was blinded to the intervention assignments and manometric results.
  - 4.4 Could assessment of the outcome have been influenced by knowledge of intervention received? → No
    Rationale: With blinded outcome assessors and standardized measurement methods, the assessment is unlikely to have been influenced by intervention knowledge.
  - 4.5 Is it likely that assessment of the outcome was influenced by knowledge of intervention received? → No
    Rationale: Given the effective blinding and consistent measurement across groups, it is highly unlikely that the outcome assessment was biased by intervention knowledge.
- Risk Assessment: LOW risk
- Reasoning:
  - The outcome measurement method was appropriate (4.1 = No), so we do not flag the measurement method as a source of bias.
  - Outcome ascertainment was consistent across groups (4.2 = No), avoiding differential measurement bias.
  - Outcome assessors were blinded (4.3 = No), which minimizes potential bias in outcome evaluation.
  - With blinding in place, the assessment was unlikely to be influenced by knowledge of the intervention (4.4 and 4.5 = No).
  - Following the decision rules: because the method of measurement is appropriate and consistent, and outcome assessors were blinded, we apply the pathway leading to a LOW risk judgment (specifically, under rule 2.3.1, if assessors are blinded, then risk is LOW).

No inconsistencies were detected in the application of the RoB 2.0 decision rules, so no corrections were necessary.

Domain 5: Risk of Bias in Selection of the Reported Result

- Signaling Question Analysis:

- 5.1 Were the data that produced this result analysed in accordance with a pre-specified analysis plan that was finalized before unblinded outcome data were available for analysis? → No information
  Rationale: The article does not provide clear details or confirmation that a pre-specified analysis plan was finalized before the unblinded outcome data were available. There is no reference to trial registration or protocol amendments that confirm such planning.
- 5.2 Is the numerical result being assessed likely to have been selected, on the basis of the results, from multiple eligible outcome measurements within the outcome domain? → No
  Rationale: The study reports the primary outcome—abdominal pain improvement at 24 months—based on clearly defined categories (no change/worse, improved, asymptomatic) and does not suggest that alternative outcome measurements were selectively chosen. All eligible outcome measurements appear to have been reported as per protocol.
- 5.3 Is the numerical result being assessed likely to have been selected, on the basis of the results, from multiple eligible analyses of the data? → No
  Rationale: There is no indication that the investigators performed and then selectively reported among multiple analyses. The methods state that comparisons were made using Fisher's exact test and the analysis was pre-planned with appropriate power calculations, with no evidence of alternative analytic approaches being preferentially selected based on the results.

- Risk Assessment: SOME CONCERNS
- Reasoning:
  - Both the reporting of multiple outcome measurements (5.2 = No) and the analyses (5.3 = No) do not raise concerns for selective reporting.
  - However, there is no information provided regarding whether the analysis plan was finalized before unblinding (5.1 = No information).
  - According to the decision rules, when both selective outcome measurement and selective analysis reporting are not evident (i.e., both are "No"), the overall risk judgment depends on whether the trial was analysed in accordance with a pre-specified plan.
  - With 5.1 rated as "No information" (which falls under {N, PN, NI}), the decision rule directs us to assign SOME CONCERNSfor this domain.

No inconsistencies were detected, and no corrections were necessary.

ID: Toouli 2000

Second Response

Domain 1: Bias Arising from the Randomization Process

- Signaling Question Analysis:
  - Was the allocation sequence random? → Y
    Evidence: The study states that "patients were randomised by the draw of a card," which is explicit evidence of randomization.
  - Was the allocation sequence concealed? → NI
    Evidence: The study does not provide explicit details on how the allocation sequence was concealed (e.g., sealed envelopes or other secure methods), so no information is available.

- Did baseline differences suggest a problem with randomization? → N
  Evidence: The report does not indicate any substantial baseline imbalances between groups.

- Risk Assessment: SOME CONCERNS
- Reasoning:
  - The allocation sequence was randomized (Y) but there is no information provided regarding concealment (NI).
  - Baseline characteristics did not show any imbalances (N).
  - Applying the predefined decision rules:
    - Since the allocation sequence concealment is NI and baseline imbalances are N (which falls under {N, PN, NI}), rule 4.1 applies, leading to a judgment of SOME CONCERNS.
  - No logical inconsistency was detected, so no corrections were necessary.

Domain 2: Bias Due to Deviations from Intended Interventions

- Signaling Question Analysis:
  Step 1: Analysis Type Determination
  - Analysis Approach: The study did not report an explicit "intention-to-treat" analysis. Instead, it excluded two patients post-randomization (one lost to follow-up and one withdrawn due to two episodes of pancreatitis).
    → Analysis Classification: Per-protocol (PP)
    Rationale: One exclusion was due to missing outcome data (loss to follow-up) while the other was for an adverse event (toxicity), a reason other than solely missing data.
- Step 3: Effect of Adhering to Intervention (applicable for PP analysis)
  - 2.1 Were participants aware of their assigned intervention? → No
    Rationale: The trial is described as double-blind; participants received a sham procedure designed to mimic the active intervention, indicating effective participant blinding.
  - 2.2 Were carers and people delivering the interventions aware of participants' assigned intervention? → Yes
    Rationale: The endoscopists performing the procedures must know whether to carry out a sphincterotomy or a sham procedure; thus, intervention providers were aware.
  - 2.3 Were important non-protocol interventions balanced across intervention groups? → Yes
    Rationale: Both groups were managed identically after the procedure (continued fasting, IV fluids, and standardized clinical assessment), indicating balanced co-interventions.
  - 2.4 Were there failures in implementing the intervention that could have affected the outcome? → No
    Rationale: Although some patients had an extension of the incision at 3 months based on manometric evidence (a protocol-mandated step), these adjustments were part of the intended intervention rather than a failure in implementation.
  - 2.5 Was there non-adherence to the assigned intervention that could have affected participants' outcomes? → No
    Rationale: Aside from the two post-randomization exclusions, there is no indication that participants did not receive the assigned intervention as per protocol.
  - 2.6 Was an appropriate analysis used to estimate the effect of adhering to intervention? → No
    Rationale: The study employed a per-protocol analysis (with exclusions for reasons beyond missing outcome data), which is less robust than an ITT approach for estimating the effect of adhering to the intervention.
- Risk Assessment: LOW risk
- Reasoning:

- Although the analysis was classified as per-protocol due to two exclusions (one for loss to follow-up and one for an adverse event), the core trial procedures minimized bias from deviations.
- Effective blinding of participants (2.1: No awareness) and balanced co-interventions (2.3: Yes) help mitigate potential bias despite the operators being aware of group allocation (2.2: Yes).
- Importantly, there were no failures in intervention implementation (2.4: No) and no evidence of non-adherence (2.5: No).
- Because the branch of the decision tree for effect of adhering to intervention—given that non-protocol interventions were balanced and both failures and non-adherence were minimal—directly returns LOW risk, the overall judgment for Domain 2 is LOW risk.

No logical inconsistencies were detected in this assessment, and the final risk judgment aligns with the predefined RoB 2.0 rules.

Domain 3: Risk of Bias Due to Missing Outcome Data

- Signaling Question Analysis:
  - Were data for this outcome available for all, or nearly all, participants randomized? → Y
    Evidence: Outcome data were available for 79 out of 81 participants (~97.5%), meeting the criterion for "all or nearly all".
  - Is there evidence that the result was not biased by missing outcome data? → Y
    Evidence: With only 2 participants missing, it is unlikely that the missing data introduced bias into the estimated effect.
  - Could missingness in the outcome depend on its true value? → N
    Evidence: There is no indication that the missing data (one lost to follow-up and one withdrawn for pancreatitis) were related to the participants' true outcomes.
  - Is it likely that missingness in the outcome depended on its true value? → N
    Evidence: The reasons provided do not suggest a dependency between the missing outcome data and the actual outcomes.
- Risk Assessment: LOW risk of bias
- Reasoning:
  - Since outcome data were available for nearly all participants (97.5% as per 3.1: Y), this satisfies the criteria for minimal missing data.
  - The negligible amount of missing data (3.2: Y) and the absence of evidence that missingness depended on the true outcome (3.3: N and 3.4: N) support that the results are not biased by missing data.
  - Accordingly, following the predefined RoB 2.0 decision rules, the overall judgment for Domain 3 is LOW risk of bias.

Domain 4: Risk of Bias in Measurement of the Outcome

- Signaling Question Analysis:
  - 4.1 Was the method of measuring the outcome inappropriate?→ No
    Rationale: The study measured pain improvement using structured questionnaires, pain diaries, and assessments by an independent clinician, which is an appropriate method for this outcome.

- 4.2 Could measurement or ascertainment of the outcome have differed between intervention groups? → No
  Rationale: The same outcome measurement procedures were applied consistently across both the sphincterotomy and sham groups.
- 4.3 Were outcome assessors aware of the intervention received by study participants? → No
  Rationale: The primary outcome assessor was a clinician who was not involved in the endoscopic therapy and was blinded to both the manometric findings and randomization status.
- 4.4 Could assessment of the outcome have been influenced by knowledge of intervention received? → No
  Rationale: Given that the outcome assessor was blinded, it is unlikely that the assessment was influenced by knowledge of the intervention.
- 4.5 Is it likely that assessment of the outcome was influenced by knowledge of intervention received? → No
  Rationale: Blinding of outcome assessors minimizes the possibility of bias in outcome assessment.

- Risk Assessment: LOW risk of bias
- Reasoning:
  - The measurement method was appropriate (4.1: No), and outcome ascertainment was consistent across groups (4.2: No).
  - Outcome assessors were blinded (4.3: No), making it unlikely that the assessment was influenced by knowledge of the intervention (4.4 and 4.5: No).
  - Following the predefined decision rules, since the measurement method is appropriate and consistent, and assessors were not aware of intervention assignments, the overall judgment for Domain 4 is LOW risk of bias.

Domain 5: Risk of Bias in Selection of the Reported Result

- Signaling Question Analysis:
  - 5.1 Were the data that produced this result analysed in accordance with a pre-specified analysis plan? → No information
    Rationale: The published report does not provide explicit details on whether the analysis plan was finalized before unblinded outcome data were available. There is no mention of trial registration or protocol finalization regarding the analysis plan.
  - 5.2 Is the numerical result being assessed likely to have been selected, on the basis of the results, from multiple eligible outcome measurements within the outcome domain? → No
    Rationale: The primary outcome (pain improvement at 24 months) is clearly defined and reported, and there is no evidence that the authors selectively reported one outcome measure over another. All eligible outcome measurements appear to have been reported as planned.
  - 5.3 Is the numerical result being assessed likely to have been selected, on the basis of the results, from multiple eligible analyses of the data? → No
    Rationale: The analysis appears to have been conducted using a straightforward approach (e.g., Fisher's exact test) without evidence of multiple competing analyses or selective reporting of analyses.
- Risk Assessment:
  SOME CONCERNS
- Reasoning:
  - Although the study reports its primary outcome comprehensively—with no signs of selective outcome measurement (5.2: No) or selective analysis reporting (5.3: No)—there is a lack of explicit information

regarding whether a pre-specified analysis plan was finalized before unblinded data became available (5.1: No information).

  - According to the predefined decision rules, when both the multiple outcome measurements and multiple analyses are judged as "No" (i.e., not indicative of selective reporting), the absence of clear evidence for a pre-specified plan (falling into the NI category) results in an overall judgment of SOME CONCERNS.
  - No discrepancies such as unusual aggregation of subscales, inconsistent outcome prioritization, or selective analysis adjustments are evident, but the missing detail about the analysis plan introduces uncertainty.

Thus, following the RoB 2.0 decision rules, the overall risk of bias for Domain 5 is determined to be SOME CONCERNS.

ID: Hanna 2012

First Response

Domain 1: Bias Arising from the Randomization Process

- Signaling Question Analysis:
  - Was the allocation sequence random? → Yes
    The study explicitly states that participants were "randomized in blocks of 4 and 2" and that the randomization schedule was created using the PLAN procedure of SAS version 9.0.citeturn0file0
  - Was the allocation sequence concealed? → Yes
    After stratifying by baseline GFR, therapy assignments were allocated using randomization envelopes provided to the site, which is a standard method for secure concealment. citeturn0file0
  - Did baseline differences suggest a problem with randomization? → No
    The baseline characteristics table shows no significant differences between treatment groups. citeturn0file0
- Risk Assessment: Low risk
- Reasoning:
  Since the allocation sequence was both random (Yes) and concealed (Yes), and there were no baseline imbalances (No), the criteria fully satisfy rule 3: "If allocation sequence concealed in {Y, PY} AND allocation sequence random in {Y, PY, NI} AND baseline imbalances in {N, PN, NI}, return LOW risk." Therefore, Domain 1 is assessed as having a Low risk of bias.

Domain 2: Bias Due to Deviations from Intended Interventions

- Step 1: Analysis Approach
  - The study explicitly states that the final population was "analyzed by the intent-to-treat approach."
    → Classification: ITT
- Step 2: Effect of Assignment to Intervention
  - 2.1 Were participants aware of their assigned intervention during the trial?
    → Yes
    Explanation: The trial was described as "open-label," so participants knew their intervention assignment.
  - 2.2 Were carers and people delivering the interventions aware of participants' assigned intervention?
    → Yes
    Explanation: As an open-label study, intervention providers were also aware of the treatment assignments.

    - 2.3 Were there deviations from the intended intervention that arose because of the trial context?
      → No
      Explanation: Although treatment adjustments (e.g., changes in UF rate) were made, these were pre-specified protocol modifications rather than deviations caused by awareness or trial context.
    - 2.4 Were these deviations likely to have affected the outcome?
      → No
      Explanation: There is no evidence that any protocol modifications or cross-overs materially impacted the outcome, which in this context (all-cause mortality and other objective endpoints) is unlikely to be affected by such adjustments.
    - 2.5 Were these deviations from intended intervention balanced between groups?
      → Yes
      Explanation: There is no indication of systematic imbalance in deviations between the UF and CD arms.
    - 2.6 Was an appropriate analysis used to estimate the effect of assignment to intervention?
      → Yes
      Explanation: The study used an ITT analysis, which appropriately includes all randomized participants regardless of post-randomization events.
    - 2.7 Was there potential for a substantial impact of the failure to analyze participants in the group to which they were randomized?
      → No
      Explanation: Despite a few early withdrawals and one instance of cross-over, all participants were included in the ITT analysis, making any potential impact on the outcome negligible.
- Risk Judgement for Step 2:
    - Part 1 (Effect of Assignment): Since both participants and personnel were aware (answers "Yes"), but no deviations arose due to the trial context (2.3 = No), Part 1 is LOW risk.
    - Part 2 (Appropriate Analysis): The ITT analysis was used (2.6 = Yes) and there was no substantial impact of exclusions (2.7 = No), so Part 2 is LOW risk.
- Step 3: Effect of Adhering to Intervention
    - Note: Because the study used an ITT approach (not a per-protocol analysis), the assessment focuses on the effect of assignment to intervention (Step 2). Therefore, Step 3 is not separately applied.

  Overall Risk Assessment for Domain 2: Low risk
- Reasoning:
  The study used an ITT analysis which included all randomized participants, thereby minimizing bias from deviations. Although the trial was open-label (participants and personnel were aware of assignments), there is no evidence that this awareness led to deviations that were both unplanned and imbalanced or that would have affected the outcomes. The protocol allowed for clinically guided adjustments that were pre-specified rather than deviations arising from the trial context. Finally, the appropriate ITT analysis ensures that any minor deviations did not materially impact the effect estimates.
  Thus, based on the predefined criteria and logical rules, Domain 2 is assessed as Low risk of bias.

Domain 3: Risk of Bias Due to Missing Outcome Data

- Signaling Question Analysis:

- 3.1 Were data for this outcome available for all, or nearly all, participants randomized? → No
  Explanation: Although the ITT analysis was employed, follow-up completeness for 90-day outcomes was notably lower in the CD arm (47.1%) compared to the UF arm (73.7%), meaning that a substantial proportion of participants did not have complete outcome data for all-cause mortality. citeturn0file0
- 3.2 Is there evidence that the result was not biased by missing outcome data? → No
  Explanation: The study does not provide clear evidence or sensitivity analyses demonstrating that the missing outcome data did not bias the mortality results. citeturn0file0
- 3.3 Could missingness in the outcome depend on its true value? → Probably yes
  Explanation: Given the differential follow-up rates between treatment groups, it is plausible that the likelihood of missing mortality data could be related to the participants' true outcomes (e.g., sicker patients might be more likely to be lost to follow-up).citeturn0file0
- 3.4 Is it likely that missingness in the outcome depended on its true value? → Probably yes
  Explanation: The imbalance in follow-up completeness suggests that the missing data in the CD group may indeed be related to worse outcomes, increasing the likelihood that missingness depends on the true value of the outcome. citeturn0file0

- Risk Assessment: High risk of bias
- Reasoning:
  Since not nearly all participants had available outcome data (3.1 = No) and there is no evidence to rule out bias from these missing data (3.2 = No), we must consider the dependency of missingness on true outcomes. The answers "Probably yes" for both 3.3 and 3.4 indicate that missing outcome data may indeed be related to the true mortality status. According to the predefined criteria, when missingness is likely to depend on the true value (3.4 in {Y, PY, NI}), the risk of bias is judged as High risk.

Domain 4: Risk of Bias in Measurement of the Outcome

- Signaling Question Analysis:
  - 4.1 Was the method of measuring the outcome inappropriate?→ No
    All-cause mortality is an objective and hard outcome that is measured using standard, validated methods (e.g., hospital records, death certificates), which are appropriate for this outcome.
  - 4.2 Could measurement or ascertainment of the outcome have differed between intervention groups? → No
    The ascertainment of mortality is expected to be uniform across both treatment arms, with no indication that it differed between groups.
  - 4.3 Were outcome assessors aware of the intervention received by study participants? → Yes
    Given the open-label design of the trial, assessors were likely aware of the assigned interventions.
  - 4.4 Could assessment of the outcome have been influenced by knowledge of intervention received? → No
    Because mortality is an objective outcome, even if assessors were aware of the intervention, their knowledge would not influence the determination of death.
  - 4.5 Is it likely that assessment of the outcome was influenced by knowledge of intervention received? → No
    The objective nature of mortality makes it highly unlikely that any such knowledge would bias the outcome assessment.
- Risk Assessment: Low risk
- Reasoning:
  The measurement method for all-cause mortality is appropriate and objective (4.1 = No). Measurement was consistent

across groups (4.2 = No), and while outcome assessors were aware of treatment assignments (4.3 = Yes), this awareness is unlikely to affect the outcome due to the objectivity of mortality (4.4 and 4.5 = No). Following the predefined criteria, since the only potential concern—assessor awareness—does not translate into bias for an objective outcome, the overall risk of bias for Domain 4 is Low risk.

Domain 5: Risk of Bias in Selection of the Reported Result

- Signaling Question Analysis:
  - 5.1 Were the data that produced this result analysed in accordance with a pre-specified analysis plan that was finalized before unblinded outcome data were available?
    → Probably yes
    Explanation: The Statistical Analysis section describes that primary and secondary endpoints (including survival status) were analyzed using predefined methods (e.g., logistic regression, ITT analysis). Although explicit details about the finalization timing of the analysis plan are not provided, the existence of a structured analysis plan suggests that one was in place before unblinded data became available.
  - 5.2 Is the numerical result being assessed likely to have been selected, on the basis of the results, from multiple eligible outcome measurements (e.g., scales, definitions, time points) within the outcome domain?
    → No
    Explanation: All-cause mortality is a definitive, objective outcome with little room for alternative measurements. The study reported mortality as one of several secondary outcomes without indication of selective measurement.
  - 5.3 Is the numerical result being assessed likely to have been selected, on the basis of the results, from multiple eligible analyses of the data?
    → No
    Explanation: The analysis of mortality appears to have been conducted using a single, pre-specified ITT approach. There is no evidence that multiple analytic methods were employed selectively for the mortality outcome.
- Risk Assessment:
  Low risk of bias
- Reasoning:
  Both potential concerns for selective reporting—multiple eligible outcome measurements (5.2) and multiple analyses of data (5.3)—are answered as "No," indicating that the reported mortality result was not selectively chosen from various measurements or analyses. Moreover, the trial appears to have been analyzed in accordance with a pre-specified plan (5.1 = Probably yes). Following the predefined judgment rule—if both multiple outcome measurements and multiple analyses are in {N, PN} and the analysis followed a pre-specified plan in {Y, PY}, then the risk is Low—Domain 5 is therefore assessed as Low risk of bias.

ID: Hanna 2012

Second Response

Domain 1: Bias Arising from the Randomization Process

- Signaling Question Analysis:

- 1.1 Was the allocation sequence random? → Yes
  The study explicitly states that participants were randomized using blocks (of 4 and 2) within two strata, and that the randomization schedule was generated using the PLAN procedure of SAS 9.0.
- 1.2 Was the allocation sequence concealed? → Yes
  Allocation assignments were provided via randomization envelopes, indicating that the allocation sequence was concealed from those enrolling participants.
- 1.3 Did baseline differences suggest a problem with randomization? → No
  The study reports no significant differences in baseline characteristics between the treatment groups.

- Risk Assessment:
  Low Risk
- Reasoning:
  - The allocation sequence was explicitly generated by a random process (SQ 1.1 = Yes).
  - The use of randomization envelopes provides evidence of secure allocation concealment (SQ 1.2 = Yes).
  - There were no substantial baseline imbalances (SQ 1.3 = No).
    According to the RoB 2.0 decision rules (specifically rule 3: if allocation concealment is Yes, the allocation sequence is random, and there are no baseline imbalances, then the domain is judged as Low Risk), the overall risk of bias for the randomization process is Low Risk.

Domain 2: Bias Due to Deviations from Intended Interventions

- Step 1: Analysis Approach
  - The study explicitly states that the final study population was analyzed by the intent-to-treat (ITT) approach. This means all randomized participants were analyzed in the groups to which they were originally assigned, even when withdrawals occurred.
- Step 2: Effect of Assignment to Intervention
  - 2.1 Were participants aware of their assigned intervention during the trial? → Yes
    As the trial was open-label, participants were clearly aware of their treatment assignment.
  - 2.2 Were carers and people delivering the interventions aware of participants' assigned intervention during the trial? → Yes
    The open-label design implies that healthcare providers were aware of the treatment assignments.
  - 2.3 Were there deviations from the intended intervention that arose because of the trial context? → No
    Although the protocol allowed for clinically directed adjustments (e.g., adjusting UF rate based on hemodynamics), these were pre-specified and part of the intended management rather than deviations arising from the open-label context.
  - 2.4 Were these deviations likely to have affected the outcome? → No
    Any protocol-based adjustments or early withdrawals (which occurred for clinical reasons) did not appear to be driven by knowledge of the treatment assignment in a way that would bias the outcome—especially for the all-cause mortality endpoint.
  - 2.5 Were these deviations from intended intervention balanced between groups? → Probably yes
    Despite a higher number of early withdrawals in the conventional diuretic (CD) group, all patients were included in the ITT analysis. The reasons for withdrawal were clinically driven and do not indicate systematic differences in the application of the interventions that would bias the outcome.

- 2.6 Was an appropriate analysis used to estimate the effect of assignment to intervention? → Yes
  The use of an ITT analysis (with all randomized patients analyzed as assigned) is an appropriate method that minimizes bias due to deviations from intended intervention.
- 2.7 Was there potential for a substantial impact of the failure to analyze participants in the group to which they were randomized?→ No
  Since the ITT approach was followed, all participants were analyzed as randomized, minimizing the potential impact of any post-randomization deviations.

- Risk Assessment for Effect of Assignment (Step 2):
  - Part 1: Although both participants and personnel were aware of the intervention (2.1 and 2.2 = Yes), there is no evidence that deviations arose specifically due to the trial context (2.3 = No). Thus, Part 1 is judged as Low risk.
  - Part 2: The analysis was appropriately conducted using ITT (2.6 = Yes) and there is no substantial impact from exclusions (2.7 = No), so Part 2 is Low risk.
- Step 3: Effect of Adhering to Intervention
  Not applicable here because the ITT analysis governs the evaluation; therefore, no additional per-protocol evaluation is required.
- Overall Risk Assessment for Domain 2: Low Risk
- Reasoning:
  - The study employed an ITT analysis, ensuring that all randomized participants were analyzed in their assigned groups despite some withdrawals.
  - Even though the trial was open-label (participants and caregivers were aware of the intervention), there is no evidence that this awareness led to deviations from the intended intervention that were specific to the trial context or that these deviations affected the outcome (all-cause mortality).
  - Protocol-based adjustments were pre-specified, and early withdrawals were managed within the ITT framework.
  - According to the predefined decision rules, with both the effect of assignment and the analysis approach rated as Low risk, the overall risk of bias due to deviations from intended interventions is Low Risk.

Domain 3: Risk of Bias Due to Missing Outcome Data

- Signaling Question Analysis:
  - 3.1 Were data for this outcome available for all, or nearly all, participants randomized? → Yes
    The study reports all-cause mortality for all 36 randomized patients, with no indication that missing data could meaningfully impact the mortality estimate.
  - 3.2 Is there evidence that the result was not biased by missing outcome data? → Yes
    The intent-to-treat analysis was preserved, and the mortality outcomes were clearly reported, indicating that missing data did not bias the result.
  - 3.3 Could missingness in the outcome depend on its true value? → No
    Given that death is a hard endpoint and the study captured mortality data for all participants, missingness is unlikely to be related to the true outcome.
  - 3.4 Is it likely that missingness in the outcome depended on its true value? → No
    There is no evidence to suggest that any missing data would have been related to the actual mortality outcomes.
- Risk Assessment: Low Risk

- Reasoning:
  The mortality outcome was available for nearly all participants, and the ITT approach ensured that every randomized patient was included in the analysis. With clear reporting and no indication that missing data were related to the outcome, the risk of bias due to missing outcome data is Low Risk.

Domain 4: Risk of Bias in Measurement of the Outcome

- Signaling Question Analysis:
  - 4.1 Was the method of measuring the outcome inappropriate?→ No
    Measuring all-cause mortality is standard and appropriate, as death is an objective outcome that is reliably documented.
  - 4.2 Could measurement or ascertainment of the outcome have differed between intervention groups? → No
    The ascertainment of mortality is objective and would be consistent across both treatment arms.
  - 4.3 Were outcome assessors aware of the intervention received by study participants? → Yes
    Given the open-label nature of the trial, outcome assessors were likely aware of the intervention assignments.
  - 4.4 Could assessment of the outcome have been influenced by knowledge of intervention received? → No
    Despite assessor awareness, the measurement of mortality is objective and not susceptible to subjective influence.
  - 4.5 Is it likely that assessment of the outcome was influenced by knowledge of intervention received? → No
    Because death is an unambiguous event, it is unlikely that knowledge of intervention assignment could have biased its assessment.
- Risk Assessment: Low Risk
- Reasoning:
  - The measurement method for the outcome (all-cause mortality) is appropriate (4.1 = No).
  - Measurement procedures were consistent between groups (4.2 = No).
  - Although outcome assessors were aware of the intervention (4.3 = Yes), the objective nature of mortality means that their awareness is unlikely to influence the measurement (4.4 and 4.5 = No).
    According to the decision rules, with an appropriate measurement method and no evidence that assessor awareness led to biased outcome assessment, the overall risk of bias in the measurement of the outcome is Low Risk.

Domain 5: Risk of Bias in Selection of the Reported Result

- Signaling Question Analysis:
  - 5.1 Were the data that produced this result analysed in accordance with a pre-specified analysis plan finalized before unblinded outcome data were available for analysis? → No information
    The article describes the statistical methods (e.g., use of logistic regression and an ITT approach) and mentions a sample size re-estimation during an internal pilot phase, but it does not explicitly confirm that a fully pre-specified analysis plan was finalized before unblinding the data.
  - 5.2 Is the numerical result being assessed likely to have been selected, on the basis of the results, from multiple eligible outcome measurements within the outcome domain? → No
    The primary outcome (time required for PCWP to be maintained at ≤18 mm Hg for at least 4 consecutive hours) is clearly defined and reported, with no indication of selective measurement among multiple alternatives.

    - 5.3 Is the numerical result being assessed likely to have been selected, on the basis of the results, from multiple eligible analyses of the data? → No
      The study appears to report the results from the pre-specified analyses without evidence that multiple alternative analytical approaches were selectively chosen.
- Risk Assessment: Some Concerns
- Reasoning:
    - Although there is no evidence of selective reporting regarding multiple outcome measurements or analyses (5.2 and 5.3 both rated as "No"), the report does not provide sufficient details to confirm that the analysis plan was finalized before unblinded data were available (5.1 = No information).
    - According to the decision rules, when the trial is not clearly analyzed in accordance with a pre-specified plan (i.e., rating in {N, PN, NI} for 5.1) even though there is no evidence of selective outcome or analysis reporting, the overall risk of bias in the selection of the reported result is judged as Some Concerns.

ID: Hu 2020

First Response

Domain 1: Bias Arising from the Randomization Process

- Signaling Question Analysis:
    - 1.1 Was the allocation sequence random? → Y
      The study explicitly states that patients were "randomly assigned," confirming the use of a random allocation method.
    - 1.2 Was the allocation sequence concealed? → NI
      There is no information provided regarding any method of allocation concealment (e.g., sealed envelopes or central randomization).
    - 1.3 Did baseline differences suggest a problem with randomization? → N
      Baseline characteristics between groups are reported as comparable, with no substantial imbalances noted.
- Risk Assessment: Some Concerns
- Reasoning:
    - The allocation sequence is confirmed as random (Y) from the study's description.
    - However, the study does not provide details on allocation concealment, resulting in a rating of No Information (NI) for this signaling question.
    - Baseline differences do not indicate any problem (N).
    - According to the RoB 2.0 decision rules, when allocation concealment is rated as NI and baseline imbalances are N, the overall risk for Domain 1 is judged as Some Concerns (Rule 4.1).

Domain 2: Bias Due to Deviations from Intended Interventions

- Step 1: Evaluation of Analysis Population
    - Analysis Approach:
        - Although the study does not explicitly state "intention-to-treat" (ITT), it adheres to CONSORT guidelines and reports group sizes exactly as randomized (40 and 60), with all randomized participants included in the comparisons.
        - Classification: ITT (by inference)
- Step 2: Effect of Assignment to Intervention

- Signaling Question Analysis:
  - 2.1 Were participants aware of their assigned intervention? → Yes
    Given the nature of the interventions (bedside ultrafiltration versus diuretic infusion), participants clearly knew which treatment they received.
  - 2.2 Were carers and intervention providers aware of participants' assignments? → Yes
    The delivery of ultrafiltration versus diuretic treatment requires specific procedures, making it apparent to the treating staff.
  - 2.3 Were there deviations from the intended intervention that arose because of the trial context? → No
    There is no report of trial-specific deviations or non-protocol interventions altering the assigned treatment.
  - 2.4 Were these deviations likely to have affected the outcome? → No
    Since no deviations were noted, there is no indication of any impact on the outcomes.
  - 2.5 Were these deviations balanced between groups? → Yes
    Any (minimal) deviations would be assumed balanced since none are reported.
  - 2.6 Was an appropriate analysis used to estimate the effect of assignment to intervention? → Yes
    The analysis appears to include all randomized participants (consistent with ITT principles), aligning with CONSORT recommendations.
  - 2.7 Was there potential for a substantial impact due to failure to analyze participants as randomized? → No
    There is no indication of exclusions or misclassifications that could bias the results.
- Risk Assessment for Effect of Assignment (Part 1 & Part 2):
  - Part 1 (Awareness and Deviations):
    Since participants and personnel were aware (Yes) but no deviations arose from the trial context (2.3 = No), Part 1 is assessed as LOW risk.
  - Part 2 (Analysis):
    With an appropriate analysis method used (2.6 = Yes) and no substantial impact from exclusions (2.7 = No), Part 2 is assessed as LOW risk.
  - Combined Judgment for Step 2:
    Since both Part 1 and Part 2 are LOW risk, the overall risk from the effect of assignment to intervention is LOW risk.

- Step 3: (Effect of Adhering to Intervention – Not Applicable)
  - Because the study is inferred to have used an ITT approach, the primary consideration is the effect of assignment. Therefore, no separate assessment for the effect of adherence is required.
- Overall Risk Assessment for Domain 2:LOW risk
- Reasoning:
  - Although both participants and caregivers were aware of treatment assignments (an inherent feature given the nature of the interventions), there is no evidence that this awareness led to deviations from the intended intervention that could have influenced the outcomes. The study's conduct and analysis (consistent with ITT principles as inferred from the CONSORT adherence and complete reporting of group sizes) indicate that deviations were either absent or negligible and balanced between groups. Thus, based on the decision rules provided, Domain 2 is judged as LOW risk for bias due to deviations from the intended interventions.

Domain 3: Risk of Bias Due to Missing Outcome Data

- Signaling Question Analysis:
  - 3.1 Were data for this outcome available for all, or nearly all, participants randomized? → Yes
    Mortality data were reported for nearly all participants (40 in the early ultrafiltration group and 60 in the control group), with no indication of substantial missing outcome data.
  - 3.2 Is there evidence that the result was not biased by missing outcome data? → Yes
    The report clearly presents mortality outcomes at both the 1-month and 3-month follow-ups, with no indication that missing data would have impacted the results.
  - 3.3 Could missingness in the outcome depend on its true value? → No
    There is no evidence suggesting that any missing data (if present at all) would be related to the actual mortality outcomes.
  - 3.4 Is it likely that missingness in the outcome depended on its true value? → No
    Given that outcome data appear to be nearly complete and reasons for any minimal missingness (if any) are not indicated, it is unlikely that missingness was dependent on the true outcomes.
- Risk Assessment: LOW risk
- Reasoning:
  Since mortality outcome data were available for almost all randomized participants (with minimal or no missing data), and there is clear evidence that any missing data would not have biased the results, the conditions for a LOW risk judgment are met according to the RoB 2.0 criteria. Therefore, Domain 3 is assessed as LOW risk of bias.

Domain 4: Risk of Bias in Measurement of the Outcome

- Signaling Question Analysis:
  - 4.1 Was the method of measuring the outcome inappropriate? → No
    Mortality is measured via objective, well-established procedures (e.g., hospital records), which is appropriate for this outcome.
  - 4.2 Could measurement or ascertainment of the outcome have differed between intervention groups? → No
    The measurement of mortality is consistent and standardized across groups.
  - 4.3 Were outcome assessors aware of the intervention received by study participants? → Yes
    Given the open-label design, outcome assessors were likely aware of the intervention, although this is less concerning for an objective outcome like mortality.
  - 4.4 Could assessment of the outcome have been influenced by knowledge of intervention received? → No
    Because mortality is an objective outcome, the potential influence of assessor awareness is negligible.
  - 4.5 Is it likely that assessment of the outcome was influenced by knowledge of intervention received? → No
    There is no indication that assessor awareness biased the measurement of mortality.
- Risk Assessment: LOW risk
- Reasoning:
  The measurement method for the outcome (all-cause mortality) is appropriate and objective (4.1 = No). There is no evidence of differential measurement between intervention groups (4.2 = No). Although outcome assessors were likely aware of treatment assignments (4.3 = Yes), this awareness does not bias the outcome measurement because mortality is objectively determined (4.4 and 4.5 = No). According to the decision rules, these responses collectively support a LOW risk judgment for Domain 4.

Domain 5: Risk of Bias in Selection of the Reported Result

- Signaling Question Analysis:
  - 5.1 Were the data that produced this result analysed in accordance with a pre-specified analysis plan finalized before unblinded outcome data were available? → No information
    The report does not provide explicit details about whether a pre-specified analysis plan was finalized prior to accessing unblinded outcome data. Although the trial is registered (albeit retrospectively), no clear confirmation is available regarding the timing or finalization of the analysis plan.
  - 5.2 Is the numerical result being assessed likely to have been selected, on the basis of the results, from multiple eligible outcome measurements within the outcome domain? → No
    The outcome of all-cause mortality is a standard, objective measure, and there is no indication that different scales, definitions, or time points were selectively chosen based on the results.
  - 5.3 Is the numerical result being assessed likely to have been selected, on the basis of the results, from multiple eligible analyses of the data? → No
    The analysis appears straightforward with no evidence suggesting that alternative analytic methods were available and selectively reported.
- Risk Assessment: SOME CONCERNS
- Reasoning:
  Both selective outcome measurement (5.2) and selective analysis (5.3) are rated as "No" (i.e., there is no indication of multiple eligible measurements or analyses from which a favorable result was chosen). However, because there is no clear information confirming that the data were analysed according to a pre-specified analysis plan finalized before unblinded data were available (5.1 = No information), the decision rule directs us to return SOME CONCERNS. This acknowledges uncertainty regarding adherence to a pre-specified protocol, despite no evidence of selective reporting in outcome measurement or analysis.

ID:Hu 2020

Second Response

Domain 1: Bias Arising from the Randomization Process

- Signaling Question Analysis:
  - Was the allocation sequence random? → Yes
    The study states that patients were "randomly assigned" to the treatment groups.
  - Was the allocation sequence concealed? → No information
    The report does not describe any method (e.g., sealed envelopes, central randomization) for concealing the allocation sequence.
  - Did baseline differences suggest a problem with randomization? → No
    Baseline characteristics between groups were reported as comparable.
- Risk Assessment: Some Concerns
- Reasoning:
  - The study provides explicit evidence of randomization (SQ1: Yes) and shows no baseline imbalances (SQ3: No).

- However, the absence of any information regarding allocation concealment (SQ2: No information) leads to a cautious approach.
- According to the RoB 2.0 decision rules, if the allocation sequence concealment is classified as “No information” and baseline differences are not problematic (i.e., in {N, PN, NI}), the overall judgment is Some Concerns (Rule 4.1).

Domain 2: Bias Due to Deviations from Intended Interventions

- Step 1: Analysis Population
  - Analysis Approach:
    - Although the manuscript does not explicitly state “intention-to-treat” (ITT), the numbers in each group (n = 40 and n = 60) match the initial randomization, and no post-randomization exclusions are reported.
    - Conclusion: The analysis is consistent with an ITT approach.
- Step 2: Effect of Assignment to Intervention
  - 2.1 Were participants aware of their assigned intervention? → Yes

    The study is open label, and participants were clearly aware of whether they received early ultrafiltration or the diuretic regimen.
  - 2.2 Were carers and people delivering the interventions aware? → Yes

    The intervention providers were not blinded given the open-label design.

    2.3 Were there deviations from the intended intervention that arose because of the trial context? → No

    No deviations from the planned interventions or protocol departures were reported as arising from the trial context.

    2.4 Were these deviations likely to have affected the outcome? → No

    There is no indication that any deviations (if they had occurred) would have impacted the primary outcomes (weight loss and urine output, and by extension the mortality follow-up).
  - 2.5 Were these deviations balanced between groups? → Yes

    In the absence of reported deviations, it is assumed that any minor departures would be balanced.

    2.6 Was an appropriate analysis used to estimate the effect of assignment? → Yes

    The analysis appears to follow an ITT principle (or is consistent with it), with no unjustified post-randomization exclusions, and appropriate statistical methods were employed.
  - 2.7 Was there potential for a substantial impact from failure to analyze participants in their randomized groups? → No

    Since the groups remained as randomized and no exclusions occurred, there is no substantial impact on the results.
- Risk Assessment for Effect of Assignment to Intervention:
  - Part 1 (Impact of Awareness and Deviations):

    Although both participants and personnel were aware of the intervention assignment, there is no evidence that deviations due to the trial context occurred (SQ 2.3 = No) or that any deviations affected the outcome (SQ 2.4 = No). Thus, Part 1 is assessed as LOW risk.
  - Part 2 (Appropriate Analysis):

    The study’s analysis is consistent with ITT principles, with no substantial exclusions or misclassifications (SQ 2.6 = Yes and SQ 2.7 = No), leading to a LOW risk rating for Part 2.

- Overall (Combining Parts 1 and 2):
  Since both parts are rated as LOW risk, the overall judgment for the effect of assignment to intervention is LOW risk.

- Risk Assessment for Domain 2: Overall Judgment: Low Risk
- Reasoning Summary:
  - The study appears to follow an ITT approach as the randomized groups were maintained without post-randomization exclusions.
  - Despite being open label (with participants and carers aware of treatment assignments), there is no evidence of deviations from the intended interventions arising due to the trial context, nor is there any indication that any such deviations would have affected the outcomes.
  - An appropriate analysis method was employed, and the potential impact of any deviations is negligible.
  - Therefore, combining the assessments for both the effect of assignment and the analytic approach leads to a final judgment of Low Risk for Domain 2.

Domain 3: Bias Due to Missing Outcome Data

- Signaling Question Analysis:
  - 3.1 Were data for this outcome available for all, or nearly all, participants randomized? → Yes
    The mortality outcome data were reported for all 100 participants (n=40 and n=60), with no indication of substantial missing data.
  - 3.2 Is there evidence that the result was not biased by missing outcome data? → Yes
    The reported mortality outcomes appear complete, and there is no suggestion that missing data could have biased the results.
  - 3.3 Could missingness in the outcome depend on its true value? → No
    Given that outcome data are nearly complete, it is unlikely that any missingness is related to the true mortality outcomes.
  - 3.4 Is it likely that missingness in the outcome depended on its true value? → No
    There is no evidence suggesting that any missing data would depend on the actual mortality values.
- Risk Assessment: Low Risk
- Reasoning:
  - Outcome data for the mortality endpoint were available for all (or nearly all) participants (SQ 3.1 = Yes).
  - There is clear evidence that missing data did not bias the results (SQ 3.2 = Yes).
  - There is no indication that any missingness depended on the true outcome (SQ 3.3 and SQ 3.4 = No).
  - According to the RoB 2.0 decision rules, when outcome data are complete (or nearly complete), the risk of bias due to missing outcome data is judged as Low Risk.

Domain 4: Bias in Measurement of the Outcome

- Signaling Question Analysis:

- 4.1 Was the method of measuring the outcome inappropriate? → No
  All-cause mortality is an objective outcome measured using routine records, making the measurement method appropriate.
- 4.2 Could measurement or ascertainment of the outcome have differed between intervention groups? → No
  The process for determining mortality is standardized and applied equally across groups.
- 4.3 Were outcome assessors aware of the intervention received by study participants? → Yes
  Given the open-label design, outcome assessors were likely aware of the interventions received.
- 4.4 Could assessment of the outcome have been influenced by knowledge of intervention received? → No
  Despite assessors being aware of group assignments, mortality is an objective endpoint unlikely to be influenced by such knowledge.
- 4.5 Is it likely that assessment of the outcome was influenced by knowledge of intervention received? → No
  There is no indication that awareness of the intervention would affect the accurate recording of mortality.

- Risk Assessment: Low Risk
- Reasoning:
  - The method of measuring all-cause mortality is appropriate (SQ 4.1 = No).
  - There is consistency in the ascertainment of mortality across intervention groups (SQ 4.2 = No).
  - Although outcome assessors were aware of the interventions (SQ 4.3 = Yes), this awareness is unlikely to influence the objective measurement of mortality (SQ 4.4 and SQ 4.5 = No).
    Applying the RoB 2.0 decision rules, these responses collectively indicate that the measurement of the outcome is unlikely to have introduced bias, leading to a final judgment of Low Risk for Domain 4.

Domain 5: Bias in Selection of the Reported Result

- Signaling Question Analysis:
  - 5.1 Were the data that produced this result analysed in accordance with a pre-specified analysis plan finalized before unblinded outcome data were available? → No information
    The report does not provide explicit details regarding a pre-specified analysis plan being finalized before the outcome data were unblinded. Although the trial was registered (retrospectively), the manuscript lacks confirmation that the analysis plan was finalized in advance.
  - 5.2 Is the numerical result being assessed likely to have been selected, on the basis of the results, from multiple eligible outcome measurements within the outcome domain? → No
    For all-cause mortality, the study appears to have reported outcomes at the planned follow-up time points (1-month and 3-months), with no evidence of selective outcome measurement reporting.
  - 5.3 Is the numerical result being assessed likely to have been selected, on the basis of the results, from multiple eligible analyses of the data? → No
    The statistical analysis was standard and there is no indication that only a subset of analyses was selectively reported.
- Risk Assessment: Some Concerns
- Reasoning:
  - While there is no evidence of selective reporting of outcome measurements or analyses (SQ 5.2 and SQ 5.3 = No), the report does not provide sufficient information regarding whether the analysis plan was pre-specified and finalized before unblinded outcome data were available (SQ 5.1 = No information).

- According to the RoB 2.0 decision rules, if the trial is not confirmed to have been analysed according to a pre-specified plan (i.e., when SQ 5.1 falls in {N, PN, NI}), the risk of bias is judged as Some Concerns even when no selective reporting of multiple outcomes or analyses is apparent.

Thus, the overall judgment for Domain 5 is Some Concerns.

ID: Tavakoli Ardakani 2019

First Response

Domain 1: Bias Arising from the Randomization Process

- Signaling Question Analysis:
  - Was the allocation sequence random? → Y

    The study explicitly describes itself as a "randomized, double-blind, placebo-controlled clinical trial," which confirms that the allocation sequence was random.
  - Was the allocation sequence concealed? → PY

    Although the study does not explicitly detail the allocation concealment mechanism, its double-blind design strongly suggests that proper concealment was in place.
  - Did baseline differences suggest a problem with randomization? → N

    The study reports no significant differences in demographic and clinical variables between the groups, indicating no problems with randomization.
- Risk Assessment: Low risk
- Reasoning:
  - The allocation sequence was confirmed as random (SQ1.1 = Y).
  - The double-blind design implies that allocation concealment was likely adequate (SQ1.2 = PY).

    There were no significant baseline imbalances (SQ1.3 = N).
  - According to the RoB 2.0 decision rules:

    If allocation concealment is in {Y, PY} AND allocation sequence random is in {Y, PY, NI} AND baseline imbalances are in {N, PN, NI}, then the risk of bias is rated as LOW risk.

    All criteria are met for a low risk of bias in Domain 1.

No inconsistencies were detected; therefore, no corrections were necessary.

Domain 2: Bias Due to Deviations from Intended Interventions

- Signaling Question Analysis:

  Preliminary Note on Analysis Type:

  Although the report does not explicitly state an "intention-to-treat" (ITT) or "per-protocol" analysis, there is no evidence of systematic exclusions for reasons such as non-adherence or protocol violations. In the absence of indications to the contrary, we assume that the analysis reflects an ITT approach (or a modified ITT excluding only missing outcome data).
- Step 2: Effect of Assignment to Intervention (Assuming ITT Analysis)
  - SQ2.1: Were participants aware of their assigned intervention during the trial? → No

    The study is described as double-blind, suggesting that participants were not aware of their allocation.
  - SQ2.2: Were carers and people delivering the interventions aware of participants' assigned intervention during the trial? → No

    A double-blind design implies that intervention providers were also blinded.

- SQ2.3: Were there deviations from the intended intervention that arose because of the trial context? → No
  There is no reported evidence of deviations arising specifically from the trial context.
- SQ2.4: Were these deviations likely to have affected the outcome? → No
  Since no deviations were reported, there is no indication that any affected the outcome.
- SQ2.5: Were these deviations from intended intervention balanced between groups? → Yes
  In the absence of deviations, they are implicitly balanced.
- SQ2.6: Was an appropriate analysis used to estimate the effect of assignment to intervention? → No information
  The report does not specify whether an ITT (or modified ITT) approach was employed.
- SQ2.7: Was there potential for a substantial impact (on the result) of the failure to analyse participants in the group to which they were randomized? → No information
  Insufficient details are provided regarding exclusions or misclassifications post-randomization.

- Risk Assessment: Some Concerns
- Reasoning:
  - Part 1 (Effect of Assignment):
    With both participants (SQ2.1) and intervention providers (SQ2.2) blinded (i.e., answered "No"), the risk from the perspective of assignment is low. No trial-context deviations (SQ2.3 and SQ2.4) further support a LOW risk for this component.
  - Part 2 (Effect of Analysis):
    However, the analysis method is not explicitly described (SQ2.6) and there is insufficient information regarding the potential impact of any exclusions or misclassifications (SQ2.7). According to the decision tree, when appropriate analysis is not confirmed (i.e., answers fall in {N, PN, NI}), the risk for this component is set to SOME CONCERNS.
    Overall Domain 2 Judgment:
    Following the rule that if one part is SOME CONCERNS (and neither part is HIGH risk), the overall risk is rated as SOME CONCERNS, we conclude a Some Concerns risk of bias for Domain 2.

No logical inconsistencies were detected, so no corrections were necessary.

Domain 3: Risk of Bias Due to Missing Outcome Data

- Signaling Question Analysis:
  - SQ3.1: Were data for this outcome available for all, or nearly all, participants randomized? → Y
    The outcome data (for biomarkers and HADS scores) are reported for the entire sample (sertraline: n=24; placebo: n=20), and there is no indication of substantial loss to follow-up. Although a 20% loss was anticipated in the sample size calculation, the published results suggest that nearly all randomized participants provided outcome data.
  - SQ3.2: Is there evidence that the result was not biased by missing outcome data? → Y
    There is no indication in the report that missing outcome data biased the results. The presentation of complete data in the outcome tables supports the conclusion that any missing data were minimal and unlikely to have introduced bias.
  - SQ3.3: Could missingness in the outcome depend on its true value? → N
    No evidence is provided that suggests the missing data (if any) were related to the true outcome values. The balanced reporting across groups and lack of discussion about differential missingness support this answer.

- SQ3.4: Is it likely that missingness in the outcome depended on its true value? → N
  Given that nearly all participants contributed data and no systematic pattern of missingness is described, it is unlikely that the missingness depended on the true outcome values.
- Risk Assessment: Low risk
- Reasoning:
  - Since outcome data were available for all (or nearly all) participants (SQ3.1 = Y) and there is clear evidence that missing data did not bias the results (SQ3.2 = Y), the criteria for a low risk of bias are met.
  - Additionally, there is no indication that missingness was related to the true outcome values (SQ3.3 = N and SQ3.4 = N).
  - According to the RoB 2.0 decision rules, when outcome data are complete or nearly complete and there is no evidence of bias from missing data, the risk of bias due to missing outcome data is classified as Low risk.

No logical inconsistencies were detected; therefore, no corrections were necessary.

Domain 4: Risk of Bias in Measurement of the Outcome

- Signaling Question Analysis:
  - SQ4.1: Was the method of measuring the outcome inappropriate? → No
    The study used well-validated methods to measure the primary efficacy outcome (e.g., HADS for depression) and biomarkers (e.g., ELISA, immunochemiluminescence), which are appropriate for continuous outcomes.
  - SQ4.2: Could measurement or ascertainment of the outcome have differed between intervention groups? → No
    Both groups were assessed using the same standardized instruments and protocols, ensuring measurement consistency.
  - SQ4.3: Were outcome assessors aware of the intervention received by study participants? → No
    The double-blind design indicates that outcome assessors (or the self-reporting participants) were not aware of the intervention allocation.
  - SQ4.4: Could assessment of the outcome have been influenced by knowledge of intervention received? → No
    Given the effective blinding and use of objective, standardized measurement tools, assessment is unlikely to have been influenced by knowledge of the intervention.
  - SQ4.5: Is it likely that assessment of the outcome was influenced by knowledge of intervention received? → No
    There is no evidence to suggest that any influence occurred, reinforcing the objectivity of the outcome measurement.
- Risk Assessment: Low risk
- Reasoning:
  - The measurement methods used are validated and appropriate (SQ4.1 = No).
  - Measurement was applied consistently across groups (SQ4.2 = No).
  - Outcome assessors were blinded (SQ4.3 = No), eliminating bias from differential awareness.
  - There is no indication that the assessment was or could have been influenced by knowledge of the intervention (SQ4.4 and SQ4.5 = No).
  - According to the RoB 2.0 decision rules, with consistent measurement methods, blinded assessors, and no risk of influence from intervention knowledge, the overall risk of bias in the measurement of the outcome is Low risk.

No inconsistencies were detected; therefore, no corrections were necessary.

Domain 5: Risk of Bias in Selection of the Reported Result

- Signaling Question Analysis:
  - SQ5.1: Were the data that produced this result analysed in accordance with a pre-specified analysis plan that was finalized before unblinded outcome data were available for analysis? → Probably yes
    The trial is registered (IRCT 201310083210N4), which implies that a pre-specified analysis plan was established. Although the report does not explicitly confirm that the plan was finalized before unblinding, the registration and adherence to standard reporting practices suggest that this was likely the case.
  - SQ5.2: Is the numerical result being assessed likely to have been selected, on the basis of the results, from multiple eligible outcome measurements within the outcome domain? → No
    The primary efficacy outcome (depression measured via HADS) appears to have been consistently reported across the prespecified time points without evidence of selective choice. The reporting aligns with what is expected per protocol.
  - SQ5.3: Is the numerical result being assessed likely to have been selected, on the basis of the results, from multiple eligible analyses of the data? → No
    There is no indication that the analysis was selectively reported from among multiple analyses. The results are presented in a straightforward manner, consistent with a pre-specified analysis plan.
- Risk Assessment: Low risk
- Reasoning:
  - Since there is an indication of a pre-specified analysis plan (SQ5.1 = Probably yes) and no evidence of selective reporting either through multiple outcome measurements (SQ5.2 = No) or multiple analyses (SQ5.3 = No), the criteria support a Low risk of bias for Domain 5.
  - Following the decision rules: when both multiple outcome measurements and multiple analyses are not a concern (i.e., answered as No), and the trial appears to have been analyzed in accordance with a pre-specified plan (Probably yes), the risk of bias is rated as Low risk.

  No inconsistencies were detected; therefore, no corrections were necessary.

ID: Tavakoli Ardakani 2019

Second Response

Domain 1: Bias Arising from the Randomization Process

- Signaling Question Analysis:
  - Was the allocation sequence random? → Y
    Rationale: The study is described as a "simple randomized" trial, which explicitly indicates that randomization was used.
  - Was the allocation sequence concealed? → NI
    Rationale: There is no explicit information provided regarding the method used for concealing the allocation sequence.
  - Did baseline differences suggest a problem with randomization? → N
    Rationale: The report states that there were no significant differences between the groups on demographic and clinical variables, indicating no problematic baseline imbalances.
- Risk Assessment: SOME CONCERNS
- Reasoning:

    - Randomization (SQ1.1): The study explicitly reports that it is a randomized trial, so the allocation sequence is considered random (Y).
    - Concealment (SQ1.2): The report does not provide details on how the allocation sequence was concealed, so it is rated as No Information (NI).
    - Baseline Imbalances (SQ1.3): There were no significant baseline differences between groups (N).
    - Decision Rule Application:
        - According to the RoB 2.0 criteria, if the allocation sequence is concealed as NI and baseline imbalances are rated as N (or PN/NI), the risk judgment should be SOME CONCERNS (Rule 4.1).
        - No other rules apply since concealment was not reported as adequate (Y/PY) and no imbalances were observed.

Thus, based on the provided criteria and the study details, the final judgment for Domain 1 is SOME CONCERNS.

Domain 2: Bias Due to Deviations from Intended Interventions

- Step 1: Analysis Type (ITT vs. PP)
    - Analysis Method: No explicit mention of "intention-to-treat", "modified ITT", "per-protocol", or "as-treated" is found in the report.
      Rationale: The study description does not specify the analysis approach, and no post-randomization exclusions are clearly detailed. Thus, we classify the analysis method as "No information."

  Since the analysis type is not explicitly stated, we default to assessing the effect of assignment to intervention (ITT approach).
- Step 2: Effect of Assignment to Intervention
    - 2.1 Were participants aware of their assigned intervention during the trial? → No
      Rationale: The study is described as a "double-blind, placebo-controlled" trial, which implies effective blinding of participants.
    - 2.2 Were carers and people delivering the interventions aware of participants' assigned intervention during the trial? → No
      Rationale: The double-blind design indicates that intervention providers were also blinded.
    - 2.3 Were there deviations from the intended intervention that arose because of the trial context? → No
      Rationale: The report does not mention any deviations caused by trial-specific factors; the interventions appear to have been delivered as intended.
    - 2.4 Were these deviations likely to have affected the outcome? → No
      Rationale: In the absence of any reported deviations, there is no reason to suspect an impact on the primary outcome.
    - 2.5 Were these deviations from intended intervention balanced between groups? → Yes
      Rationale: Since no deviations are reported, they are implicitly balanced across the sertraline and placebo groups.
    - 2.6 Was an appropriate analysis used to estimate the effect of assignment to intervention? → No information
      Rationale: The study does not explicitly describe the analysis approach (e.g., ITT or mITT) used for estimating intervention effects.
    - 2.7 Was there potential for a substantial impact of the failure to analyze participants in the group to which they were randomized? → No information

Rationale: Without clear details on exclusions or the analysis method, the potential impact cannot be determined.

- Risk Assessment – Part 1 (Effect of Assignment):
  - Blinding: Participants and intervention providers are rated No (i.e., they were not aware), which supports a low risk for deviations in this domain.
  - Implementation: There is no indication of failures in implementing the intervention or non-adherence that could affect the outcome.

    → Thus, for the effect of assignment (Part 1), the risk is considered LOW risk.
- Risk Assessment – Part 2 (Appropriate Analysis):
  - Given that the analysis method is not explicitly described (rated as No information in 2.6) and the potential impact of any misclassification is unclear (2.7 is No information), we default to SOME CONCERNS for this component.
- Overall Domain 2 Risk Judgment:
  - Since Part 1 is LOW risk and Part 2 is SOME CONCERNS (with neither reaching HIGH risk), the final judgment for Domain 2 is SOME CONCERNS.
- Final Summary for Domain 2:
- Signaling Question Analysis:
  - 2.1 Participants aware of intervention → No
  - 2.2 Carers aware of intervention → No
  - 2.3 Deviations due to trial context → No
  - 2.4 Deviations affecting outcome → No
  - 2.5 Deviations balanced → Yes
  - 2.6 Appropriate analysis for assignment → No information
  - 2.7 Impact of analysis failure → No information
- Risk Assessment: SOME CONCERNS
- Reasoning:
  - The double-blind design ensures that both participants and carers were not aware of the assigned intervention, and no deviations from the intended intervention were reported.
  - However, the analysis method is not explicitly described, leaving uncertainty about whether the effect of assignment was estimated appropriately.
  - With no evidence of implementation failures or imbalances in deviations, the effect of assignment is rated LOW risk, but the uncertainty in the analysis approach introduces SOME CONCERNS overall.

This assessment is based solely on the information extracted from the provided study text.

Domain 3: Risk of Bias Due to Missing Outcome Data

- Signaling Question Analysis:
  - 3.1 Were data for this outcome available for all, or nearly all, participants randomized? → Y

    Rationale: The study reports outcome data for all randomized participants (sertraline: n=24, placebo: n=20) with no indication of substantial missing data, meeting the ≥95% threshold for a continuous outcome.
  - 3.2 Is there evidence that the result was not biased by missing outcome data? → Y

    Rationale: Given the complete reporting of outcomes, there is clear evidence that missing data did not bias the results.

- 3.3 Could missingness in the outcome depend on its true value? → N
  Rationale: With nearly all data available, it is unlikely that any missingness would depend on the true outcome values.
- 3.4 Is it likely that missingness in the outcome depended on its true value? → N
  Rationale: The minimal or absent missing data suggests that missingness did not depend on the true outcome values.

- Risk Assessment: LOW risk of bias
- Reasoning:
  - Outcome data were available for nearly all participants, fulfilling the criterion for continuous outcomes.
  - There is clear evidence that any missing data (if present) did not bias the results.
  - It is unlikely that missingness depended on the true outcome values.
  - According to the RoB 2.0 decision rule—if outcome data for all (or nearly all) participants is rated as Y—the domain is judged as LOW risk.

Domain 4: Risk of Bias in Measurement of the Outcome

- Signaling Question Analysis:
  - 4.1 Was the method of measuring the outcome inappropriate? → No
    Rationale: The primary outcome (efficacy measured as a continuous outcome via HADS scores) is assessed using a well-validated, standardized questionnaire. In addition, immunoassays for biomarkers are appropriate for their respective outcomes.
  - 4.2 Could measurement or ascertainment of the outcome have differed between intervention groups? → No
    Rationale: The measurement methods (self-administered HADS and laboratory assays) were applied consistently across both the sertraline and placebo groups.
  - 4.3 Were outcome assessors aware of the intervention received by study participants? → No
    Rationale: The study's double-blind design indicates that both participants and any personnel involved in outcome assessment were blinded to treatment assignment.
  - 4.4 Could assessment of the outcome have been influenced by knowledge of intervention received? → No
    Rationale: Blinding and the use of objective, standardized measures make it unlikely that outcome assessment was influenced by intervention knowledge.
  - 4.5 Is it likely that assessment of the outcome was influenced by knowledge of intervention received? → No
    Rationale: There is no evidence to suggest that any influence occurred, given the effective blinding and consistency in measurement across groups.
- Risk Assessment: LOW risk of bias
- Reasoning:
  - The measurement method (HADS and laboratory assays) is appropriate and validated.
  - There is no indication of differential measurement across intervention groups.
  - Outcome assessors were blinded, minimizing any potential influence from knowledge of the intervention.
  - Based on the RoB 2.0 decision rules, these findings lead to a LOW risk of bias judgment for this domain.

Domain 5: Risk of Bias in Selection of the Reported Result

- Signaling Question Analysis:
    - 5.1 Were the data that produced this result analysed in accordance with a pre-specified analysis plan? → Probably yes
      Rationale: The trial is registered (IRCT 201310083210N4), which indicates the existence of a pre-specified analysis plan. Although the report does not explicitly state that the plan was finalized before unblinding, trial registration provides reasonable evidence that the analysis plan was established a priori.
    - 5.2 Is the numerical result being assessed likely to have been selected from multiple eligible outcome measurements? → No
      Rationale: The study reports on the primary efficacy outcome (measured via HADS scores at several time points) along with pre-specified biomarker outcomes. There is no indication that outcomes were selectively reported from among multiple measurements.
    - 5.3 Is the numerical result being assessed likely to have been selected from multiple eligible analyses of the data? → No
      Rationale: The analysis methods are reported consistently (e.g., appropriate statistical tests such as t-test, Mann–Whitney U, etc.), and there is no evidence that the authors conducted multiple analyses and selectively reported favorable results.
- Risk Assessment: LOW risk of bias
- Reasoning:
    - The presence of trial registration supports the notion that a pre-specified analysis plan was in place (rated as Probably yes for 5.1).
    - There is no evidence of selective reporting based on outcome measurement (5.2 = No) or on selective analysis (5.3 = No).
    - According to the decision rules, if both multiple outcome measurements and multiple analyses are rated as {N, PN} and the trial was analyzed in accordance with a pre-specified plan in {Y, PY}, the overall judgment is LOW risk of bias.
    - Therefore, the final judgment for Domain 5 is LOW risk of bias.

ID: Cavalcanti 2020

First Response

Domain 1: Bias Arising from the Randomization Process

- Signaling Question Analysis:
    - Was the allocation sequence random? → Yes
      The study explicitly states that patients were randomly assigned in a 1:1:1 ratio using a central electronic system (RedCap), indicating an explicit and proper randomization process.
    - Was the allocation sequence concealed? → Yes
      Allocation was performed centrally using RedCap, which provides secure concealment of the sequence from the investigators.
    - Did baseline differences suggest a problem with randomization? → No
      Baseline characteristics (e.g., age, sex, and coexisting conditions) were well balanced across the treatment groups, indicating no substantial imbalances.

- Risk Assessment: Low
- Reasoning:
  - The trial provided explicit evidence of both random sequence generation and secure allocation concealment by employing a central electronic randomization system.
  - The absence of baseline imbalances across groups supports that the randomization process was properly executed.
  - According to the predefined RoB 2.0 rules:
    - With allocation sequence concealed as Yes,
    - The allocation sequence being random as Yes,
    - And baseline differences being rated as No,
  - Rule 3 applies, leading to a final risk judgment of Low Risk for Domain 1.
  - No errors or logical inconsistencies were detected upon cross-checking with the RoB 2.0 decision tree.

Domain 2: Bias Due to Deviations from Intended Interventions

- Step 1: Analysis Type Evaluation (ITT vs. PP)
  - The study employed a modified intention-to-treat (mITT) analysis that included only patients with a confirmed diagnosis of Covid-19.
  - Exclusions (e.g., one withdrawal of consent and one duplicate enrollment) were pre-specified and based on eligibility confirmation rather than post-randomization non-adherence or protocol violations.
  - These modifications are not driven by deviations such as non-adherence or toxicity but by eligibility verification.
  - Classification: Although termed "modified ITT," the exclusions are appropriate, so for our purpose the analysis is treated effectively as an ITT approach.
- Step 2: Effect of Assignment to Intervention
  - 2.1 Were participants aware of their assigned intervention? → Yes
    The trial was open-label, so participants were aware of their treatment assignment.
  - 2.2 Were carers and those delivering the interventions aware? → Yes
    Given the open-label design, intervention providers were also aware of group assignments.
  - 2.3 Did deviations from the intended intervention arise because of the trial context? → No
    There is no clear evidence that the open-label context induced deviations (e.g., differential use of co-interventions) that are attributable to the trial setting.
  - 2.4 Were these deviations likely to have affected the outcome? → No
    Even if minor deviations had occurred, the outcome—an objective measure (all-cause mortality at day 28)—is unlikely to be substantially affected by them.
  - 2.5 Were these deviations balanced between groups? → Yes
    Randomization and standardized care protocols suggest that any minor deviations would have been balanced across the treatment arms.
  - 2.6 Was an appropriate analysis used to estimate the effect of assignment to intervention? → Yes
    The pre-specified mITT analysis (which excludes only those without confirmed Covid-19 and a few minimal cases) is appropriate for estimating the effect of assignment.
  - Risk Judgment for Part 1 (Effect of Assignment):

        - Because both participants and personnel were aware (answer: Yes) but there is no evidence that deviations arose due to the trial context, Part 1 is judged Low Risk.
    - Risk Judgment for Part 2 (Appropriate Analysis):
        - With an appropriate (mITT) analysis in place, Part 2 is judged Low Risk.
- Step 3: Overall Risk for Domain 2
    - Combined Judgment: Since Part 1 and Part 2 are both Low Risk, the overall risk of bias due to deviations from intended interventions is Low Risk.
- Reasoning:
    - Although the open-label design means that both participants and caregivers knew the treatment assignments, the trial's deviations from the intended intervention are minimal and do not stem from the trial context (e.g., differential co-interventions or non-adherence affecting outcomes).
    - The mITT analysis was pre-specified and based on confirming eligibility rather than on post-randomization deviations, and any exclusions were minimal and balanced.
    - Given that the outcome (all-cause mortality at day 28) is objective and unlikely to be influenced by minor deviations, the overall risk of bias in this domain is judged to be Low Risk.

Domain 3: Risk of Bias Due to Missing Outcome Data

- Signaling Question Analysis:
    - 3.1 Were data for this outcome available for all, or nearly all, participants randomized? → Yes
      The report indicates that follow-up was completed for all 665 patients included in the modified intention-to-treat analysis, meaning that outcome data (including for all-cause mortality at day 28) were available for nearly all randomized participants.
    - 3.2 Is there evidence that the result was not biased by missing outcome data? → Yes
      With complete follow-up for the analyzed participants, there is clear evidence that missing data did not bias the results.
    - 3.3 Could missingness in the outcome depend on its true value? → No
      Since outcome data were available for almost all participants, it is unlikely that any missingness (if present) would be related to the true outcome.
    - 3.4 Is it likely that missingness in the outcome depended on its true value? → No
      Given the near-complete data collection, there is no indication that the few (if any) missing data would depend on the actual outcome values.
- Risk Assessment: Low Risk
- Reasoning:
    - Outcome data were available for nearly all participants randomized (or all patients included in the modified ITT analysis), with follow-up reportedly completed for all 665 patients.
    - There is clear evidence that missing data are minimal and that any potential missingness is unlikely to be related to the true outcome values.
    - Following the predefined RoB 2.0 rules, since 3.1 is answered as Yes, the risk of bias due to missing outcome data is judged to be Low Risk.

Domain 4: Risk of Bias in Measurement of the Outcome

- Signaling Question Analysis:
  - 4.1 Was the method of measuring the outcome inappropriate? → No
    Measurement of all-cause mortality is objective and standard, making the method appropriate.
  - 4.2 Could measurement or ascertainment of the outcome have differed between intervention groups? → No
    Outcome ascertainment (e.g., determining mortality) is consistent and unlikely to vary between groups.
  - 4.3 Were outcome assessors aware of the intervention received by study participants? → Yes
    Due to the open-label design, outcome assessors were aware of the assigned interventions.
  - 4.4 Could assessment of the outcome have been influenced by knowledge of intervention received? → No
    Because all-cause mortality is an objective outcome, any awareness of intervention is unlikely to have influenced its assessment.
  - 4.5 Is it likely that assessment of the outcome was influenced by knowledge of intervention received? → No
    Given the objective nature of mortality, assessor awareness does not translate into bias in outcome measurement.
- Risk Assessment: Low Risk
- Reasoning:
  - The measurement method for all-cause mortality is appropriate and objective.
  - Although the study's open-label design means that outcome assessors were aware of the interventions, the consistency of outcome ascertainment across groups (as mortality is unambiguous) ensures that there is no differential measurement.
  - The potential for bias is minimized because the objective nature of mortality renders the influence of assessor awareness negligible.
  - According to the predefined decision rules, with an appropriate measurement method (4.1 = No), consistent ascertainment across groups (4.2 = No), and assessment unlikely to be influenced by knowledge of intervention (4.4 and 4.5 = No) despite assessor awareness (4.3 = Yes), the overall judgment for Domain 4 is Low Risk.

Domain 5: Risk of Bias in Selection of the Reported Result

- Signaling Question Analysis:
  - 5.1 Were the data that produced this result analysed in accordance with a pre-specified analysis plan that was finalized before unblinded outcome data were available? → Yes
    The study protocol indicates that changes (e.g., switching the primary outcome from a six-level to a seven-level ordinal scale and modifying the analysis population to include only confirmed cases) were implemented before the first interim analysis, i.e., before outcome data were unblinded. This confirms that the analysis plan was finalized in advance.
  - 5.2 Is the numerical result being assessed likely to have been selected, on the basis of the results, from multiple eligible outcome measurements within the outcome domain? → No
    There is no evidence of selective outcome measurement. The outcomes, including all-cause mortality at day 28, appear to have been reported as pre-specified without any indication that only favorable or selective measurements were chosen.
  - 5.3 Is the numerical result being assessed likely to have been selected, on the basis of the results, from multiple eligible analyses of the data? → No

The study reports a series of pre-specified sensitivity analyses (e.g., analyses in the modified ITT population and other sensitivity populations), and there is no clear indication that selective analysis reporting occurred.

- Risk Assessment: Low Risk
- Reasoning:
  - The analysis plan for the study was finalized before unblinded outcome data were available, as evidenced by the pre-specified modifications made prior to the first interim analysis.
  - There is no indication that the results (including the assessment of all-cause mortality at day 28) were selectively chosen from among multiple eligible outcome measurements or analyses.
  - According to the predefined decision rules, because the trial adhered to a pre-specified analysis plan (5.1 = Yes) and there is no evidence of selective reporting in outcome measurements (5.2 = No) or analyses (5.3 = No), the risk of bias in the selection of the reported result is judged to be Low Risk.

ID: Cavalcanti 2020

Second Response

Domain 1: Bias Arising from the Randomization Process

- Signaling Question Analysis:
  - Was the allocation sequence random? → Y
    The study explicitly states that patients were "randomly assigned in a 1:1:1 ratio" using a block design with blocks of six.
  - Was the allocation sequence concealed? → Y
    Randomization was performed centrally via an electronic case-report form system (RedCap), which indicates secure allocation concealment.
  - Did baseline differences suggest a problem with randomization? → N
    Baseline characteristics across groups were balanced, with no substantial imbalances reported.
- Risk Assessment: Low
- Reasoning:
  According to the RoB 2.0 decision rules:
  - The allocation sequence was generated randomly (SQ1.1 = Y).
  - Concealment was adequately secured (SQ1.2 = Y).
  - There were no notable baseline imbalances (SQ1.3 = N).
    Rule 3 applies: when allocation concealment is in {Y, PY}, the randomization is in {Y, PY, NI}, and baseline imbalances are in {N, PN, NI}, the risk is judged as Low.
    Therefore, the risk of bias due to the randomization process is Low.

Domain 2: Bias Due to Deviations from Intended Interventions

- Signaling Question Analysis:
  Step 1: Analysis Approach
  - Analysis Type: The study primarily used a modified intention-to-treat (mITT) analysis.

        - Exclusions post-randomization were minimal (one withdrawal of consent and one duplicate enrollment) and do not reflect non-adherence or treatment deviations.
        - These exclusions are administrative rather than reflective of participants deviating from their assigned intervention.
- Step 2: Effect of Assignment to Intervention
    - 2.1 Were participants aware of their assigned intervention? → Yes
      Reasoning: The trial is open-label, so participants knew their treatment assignment.
    - 2.2 Were carers and people delivering the interventions aware of participants' assigned intervention? → Yes
      Reasoning: Being an open-label study, treating clinicians and staff were aware of the allocations.
    - 2.3 Were there deviations from the intended intervention that arose because of the trial context? → No
      Reasoning: Although the trial was open-label, there is no evidence of systematic deviations from the intended interventions driven by the trial context; standard care was administered per protocol.
    - 2.4 Were these deviations likely to have affected the outcome? → No
      Reasoning: The primary outcome (all-cause mortality at day 28) is objective, and no deviations were reported that could meaningfully influence it.
    - 2.5 Were these deviations from intended intervention balanced between groups? → Yes
      Reasoning: There is no indication of differential deviations across treatment arms.
    - 2.6 Was an appropriate analysis used to estimate the effect of assignment to intervention? → Yes
      Reasoning: The mITT approach (which excludes only those with administrative issues or missing outcomes) is appropriate, as it minimizes bias from post-randomization exclusions.
    - 2.7 Was there potential for a substantial impact of the failure to analyze participants in the group to which they were randomized? → No
      Reasoning: The number of exclusions was very small (2 out of 667) and unlikely to affect the outcome.
- Risk Assessment:
    - Part 1 (Effect of Assignment): LOW risk
      Since both participants and carers were aware (Y) but there were no deviations due to trial context (N), and any potential deviations were balanced, the effect of assignment yields LOW risk.
    - Part 2 (Analysis Method): LOW risk
      An appropriate mITT analysis was used with minimal and justified exclusions, and the potential impact of any misclassification is negligible.
    - Overall for Domain 2: Low Risk
- Reasoning Summary:
  The study used a modified intention-to-treat approach with exclusions that were administrative (withdrawal of consent and duplicate enrollment) rather than reflective of non-adherence or treatment deviations. Although the open-label design means that both participants and personnel were aware of the treatment assignments, there is no evidence that this awareness led to trial-context deviations or that any deviations affected the primary outcome (all-cause mortality at day 28). Deviations (if any) were balanced across groups and the analysis method was appropriate, supporting an overall LOW risk of bias in Domain 2.

Domain 3: Risk of Bias Due to Missing Outcome Data

- Signaling Question Analysis:

- 3.1 Were data for this outcome available for all, or nearly all, participants randomized? → Y
  Reasoning: The report indicates that follow-up was completed for all remaining 665 participants, meaning missing data are minimal and unlikely to affect the outcome estimate.
- 3.2 Is there evidence that the result was not biased by missing outcome data? → Y
  Reasoning: With nearly complete follow-up, there is clear evidence that missing data did not bias the results.
- 3.3 Could missingness in the outcome depend on its true value? → No
  Reasoning: Given the extremely low level of missing data, it is unlikely that the few missing outcomes would be systematically related to the true outcome values.
- 3.4 Is it likely that missingness in the outcome depended on its true value? → No
  Reasoning: The negligible amount of missing data precludes a dependency that could meaningfully impact the outcome.

- Risk Assessment: Low
- Reasoning:
  Since outcome data were available for nearly all participants (SQ3.1 = Y) and there is clear evidence that missing data did not bias the results (SQ3.2 = Y), with no indication that missingness depended on the true outcome values (SQ3.3 = No; SQ3.4 = No), the predefined RoB 2.0 rules indicate a LOW risk of bias for Domain 3.

Domain 4: Bias in Measurement of the Outcome

- Signaling Question Analysis:
  - 4.1 Was the method of measuring the outcome inappropriate? → No
    Reasoning: All-cause mortality is an objective outcome measured using standard procedures (e.g., hospital records, vital status checks), so the method is appropriate.
  - 4.2 Could measurement or ascertainment of the outcome have differed between intervention groups? → No
    Reasoning: The ascertainment of death is uniform across groups, with no indication that the method differed by intervention.
  - 4.3 Were outcome assessors aware of the intervention received by study participants? → Yes
    Reasoning: Given the open-label design, outcome assessors were aware of the treatment assignments.
  - 4.4 Could assessment of the outcome have been influenced by knowledge of intervention received? → No
    Reasoning: Despite assessor awareness, the objectivity of mortality minimizes any potential influence on the measurement.
  - 4.5 Is it likely that assessment of the outcome was influenced by knowledge of intervention received? → No
    Reasoning: The outcome (all-cause mortality) is not subject to interpretation, so any assessor knowledge is unlikely to affect the results.
- Risk Assessment: Low Risk
- Reasoning:
  The method for measuring the outcome is appropriate (4.1 = No) and consistently applied across intervention groups (4.2 = No). Although assessors were aware of the intervention (4.3 = Yes), the objectivity of death as an outcome means that their awareness is unlikely to have influenced the measurement (4.4 = No, 4.5 = No). According to the RoB 2.0 decision rules, these conditions support an overall LOW risk of bias in the measurement of the outcome.

Domain 5: Risk of Bias in Selection of the Reported Result

- Signaling Question Analysis:
  - 5.1 Were the data that produced this result analysed in accordance with a pre-specified analysis plan finalized before unblinded outcome data were available? → Y
    Reasoning: The trial protocol and registration indicate that a pre-specified analysis plan was in place. Changes (e.g., to the modified intention-to-treat population and outcome scale) were made before any patient reached the key follow-up time, confirming that the analysis plan was finalized prior to unblinding.
  - 5.2 Is the numerical result being assessed likely to have been selected, on the basis of the results, from multiple eligible outcome measurements within the outcome domain? → No
    Reasoning: For the outcome of all-cause mortality (or in-hospital death, as reported), there is no indication of multiple competing measurements or alternative definitions that would allow selective reporting. The measurement appears objective and uniquely defined.
  - 5.3 Is the numerical result being assessed likely to have been selected, on the basis of the results, from multiple eligible analyses of the data? → No
    Reasoning: The analysis methods for mortality were pre-specified (using a Cox proportional-hazards model) and reported in the methods section. There is no evidence of selective analysis or multiple alternative analytical approaches being available and chosen based on favorable results.
- Risk Assessment: Low Risk
- Reasoning:
  As both potential sources of selective reporting are absent—there is no indication that multiple outcome measurements (5.2 = No) or multiple analyses (5.3 = No) were available from which a favorable result could be selectively reported—and the trial data were analyzed in accordance with a pre-specified plan (5.1 = Y), the RoB 2.0 decision rules lead to a LOW risk of bias for Domain 5.

ID: Peters 2016

First Response

Domain 1: Bias Arising from the Randomization Process

- Signaling Question Analysis:
  - 1.1 Was the allocation sequence random? → Y
    The study explicitly states that patients were "randomly assigned" using an interactive voice-response–Web-response system. citeturn0file0
  - 1.2 Was the allocation sequence concealed? → Y
    The use of an interactive voice-response–Web-response system indicates secure allocation concealment. citeturn0file0
  - 1.3 Did baseline differences suggest a problem with randomization? → N
    Baseline characteristics were well balanced between treatment groups, with no substantial differences noted. citeturn0file0
- Risk Assessment: LOW risk
- Reasoning:

- The study provided explicit evidence of random allocation (1.1 = Y) using a central system, which also ensured secure concealment (1.2 = Y).
- There were no significant baseline imbalances between the treatment groups (1.3 = N).
- According to the predefined rule:
  If allocation sequence concealed in {Y, PY} AND allocation sequence random in {Y, PY, NI} AND baseline imbalances in {N, PN, NI}, return LOW risk.
  This condition is fully met, so the overall risk of bias for this domain is LOW risk.

Domain 2: Bias Due to Deviations from Intended Interventions

- Step 1: Evaluation of Analysis Type
  - The study clearly states:
    "In accordance with the intention-to-treat principle, analyses included all patients who were randomly assigned to receive a study drug; patients were analyzed according to initial group assignment." citeturn0file0
  - Analysis Classification: ITT (Intention-to-Treat)
    → Proceed to Step 2 (effect of assignment to intervention).
- Step 2: Effect of Assignment to Intervention
  - 2.1 Were participants aware of their assigned intervention during the trial? → No
    The study is described as "double-blind," indicating that participants were effectively blinded. citeturn0file0
  - 2.2 Were carers and people delivering the interventions aware of participants' assigned intervention during the trial? → No
    The double-blind design implies that intervention providers were also blinded to the treatment assignments. citeturn0file0
  - 2.3 Were there deviations from the intended intervention that arose because of the trial context? → No
    No evidence suggests that deviations from the intended intervention arose specifically due to the trial setting. citeturn0file0
  - 2.4 Were these deviations likely to have affected the outcome? → No
    There is no indication that any deviations (if present) had an impact on the primary outcome (asthma exacerbation). citeturn0file0
  - 2.5 Were these deviations from the intended intervention balanced between groups? → Yes
    Any potential deviations were minimal and, if present, appeared to be balanced across treatment arms. citeturn0file0
  - 2.6 Was an appropriate analysis used to estimate the effect of assignment to intervention? → Yes
    The use of an ITT analysis, which includes all randomized patients regardless of early discontinuation, is appropriate. citeturn0file0
  - 2.7 Was there potential for a substantial impact (on the result) of the failure to analyze participants in the group to which they were randomized? → No
    Nearly all patients were included in the analysis, so any exclusions were too minimal to substantially impact the results. citeturn0file0
- Risk of Bias Judgement
  - Part 1 (Awareness and Deviations):
    - Participants and personnel were blinded (2.1 and 2.2 = No).

    - No trial-specific deviations were observed (2.3 = No; 2.4 = No), and any minor deviations were balanced (2.5 = Yes). → Part 1 = LOW risk.
  - Part 2 (Appropriate Analysis):
    - An intention-to-treat analysis was employed (2.6 = Yes) and the potential impact of any exclusions was negligible (2.7 = No).
      → Part 2 = LOW risk.
- Overall for Domain 2:
  - Since both Part 1 and Part 2 are LOW risk, the combined judgment for Domain 2 is LOW risk.
- Reasoning Summary:
  The study utilized an ITT analysis, ensuring that all randomized patients were analyzed according to their initial assignment. Effective blinding minimized participant and personnel awareness of the intervention assignments. No notable deviations from the intended interventions that could affect the outcome were observed, and any minor deviations were balanced between groups. These factors collectively support a LOW risk of bias for Domain 2.

Domain 3: Risk of Bias Due to Missing Outcome Data

- Signaling Question Analysis:
  - 3.1 Were data for this outcome available for all, or nearly all, participants randomized? → Yes
    The report indicates that all patients were followed for the full study period for the primary endpoint (asthma exacerbation), and nearly all participants provided outcome data (with follow-up maintained even for those discontinuing treatment), meeting the ≥95% criterion. citeturn0file0
  - 3.2 Is there evidence that the result was not biased by missing outcome data? → Yes
    The use of an intention-to-treat analysis, which included all randomized patients, and the near-complete follow-up suggest that missing data did not bias the results. citeturn0file0
  - 3.3 Could missingness in the outcome depend on its true value? → No
    Given the comprehensive follow-up and minimal missing data, there is no evidence to suggest that missingness was related to the true outcome values. citeturn0file0
  - 3.4 Is it likely that missingness in the outcome depended on its true value? → No
    The minimal extent of missing data and the complete follow-up imply that missingness is unlikely to be dependent on the true outcome. citeturn0file0
- Risk Assessment: LOW risk
- Reasoning:
  - Nearly all participants provided outcome data for the primary endpoint (asthma exacerbation), satisfying the "nearly all" criterion (3.1 = Yes).
  - The clear use of an intention-to-treat analysis and complete follow-up supports the conclusion that missing data did not introduce bias (3.2 = Yes).
  - There is no indication that the missing outcome data were related to the true outcome values (3.3 and 3.4 = No).
  - According to the predefined decision rules, with 3.1 and 3.2 being "Yes," the overall risk of bias for missing outcome data is classified as LOW risk.

Domain 4: Risk of Bias in Measurement of the Outcome

- Signaling Question Analysis:

- 4.1 Was the method of measuring the outcome inappropriate? → No
  The study used standard, objective criteria to define asthma exacerbations, which is an appropriate method for measuring this outcome. citeturn0file0
- 4.2 Could measurement or ascertainment of the outcome have differed between intervention groups? → No
  The measurement methods were applied uniformly across groups, with no indication of differential assessment between treatment arms. citeturn0file0
- 4.3 Were outcome assessors aware of the intervention received by study participants? → No
  The trial was double-blind, and for adjudicated outcomes (e.g., serious asthma-related events), assessors were explicitly stated to be unaware of treatment assignments. This blinding extends to outcome measurement procedures for efficacy outcomes. citeturn0file0
- 4.4 Could assessment of the outcome have been influenced by knowledge of intervention received? → No
  Given the effective blinding and objective outcome criteria, there is no indication that outcome assessment was influenced by treatment knowledge. citeturn0file0
- 4.5 Is it likely that assessment of the outcome was influenced by knowledge of intervention received? → No
  The robust blinding measures and standardized, objective outcome definitions make it unlikely that assessment was biased by intervention knowledge. citeturn0file0

- Risk Assessment: LOW risk
- Reasoning:
  - The outcome measurement method was appropriate (4.1 = No), and there was no evidence of differences in outcome ascertainment between groups (4.2 = No).
  - Outcome assessors were blinded to the intervention (4.3 = No), which, coupled with the objective nature of the outcome measurement, minimizes the risk of bias from knowledge of treatment allocation (4.4 = No and 4.5 = No).
  - According to the decision rules, when the measurement method is appropriate and consistent across groups with blinded assessors and no evidence of influence on assessment, the overall risk of bias in measurement of the outcome is LOW risk.

Domain 5: Risk of Bias in Selection of the Reported Result

- Signaling Question Analysis:
  - 5.1 Were the data analysed in accordance with a pre-specified analysis plan? → Yes
    The study protocol and statistical analysis plan were established in discussion with the FDA and approved before the study began. The primary efficacy outcome (asthma exacerbation) was analyzed as pre-specified in the protocol, suggesting that the analysis plan was finalized before unblinded data were available. citeturn0file0
  - 5.2 Is the numerical result likely to have been selected from multiple eligible outcome measurements? → No
    The primary outcome was clearly defined and consistently reported. There is no evidence that the result was chosen from multiple measurements or that selective reporting of alternative outcome definitions occurred. citeturn0file0
  - 5.3 Is the numerical result likely to have been selected from multiple eligible analyses of the data? → No
    The analyses reported for the primary efficacy outcome align with the pre-specified statistical methods outlined in the protocol, and there is no indication of selective reporting among alternative analyses. citeturn0file0

- Risk Assessment: LOW risk
- Reasoning:
  - The trial was analyzed in accordance with a pre-specified analysis plan (5.1 = Yes).
  - There is no evidence of selective reporting based on multiple eligible outcome measurements (5.2 = No) or selective analysis of the data (5.3 = No).
  - Based on the decision rule—since both potential selective reporting issues are absent and the analysis followed a pre-specified plan—the risk of bias in the selection of the reported result is determined to be LOW risk.

ID: Peters 2016

Second Response

Domain 1: Bias Arising from the Randomization Process

- Signaling Question Analysis:
  - 1.1 Was the allocation sequence random? → Y
    The study explicitly states that patients were "randomly assigned" in a 1:1 ratio using an interactive voice–response/Web–response system, which is evidence of randomization.
  - 1.2 Was the allocation sequence concealed? → Y
    The use of an interactive voice-response/Web–response system to manage enrollment and randomization indicates that the allocation was securely concealed.
  - 1.3 Did baseline differences suggest a problem with randomization? → N
    Baseline characteristics were similar between groups, with no substantial imbalances noted.
- Risk Assessment: Low Risk
- Reasoning:
  The allocation sequence was explicitly generated randomly (Y) and securely concealed (Y) through an automated system, and there were no notable baseline differences (N). According to the predefined rules—if the allocation sequence is concealed (Y) and random (Y) and there are no baseline imbalances (N)—the overall risk of bias for this domain is judged to be Low Risk.

Domain 2: Bias Due to Deviations from Intended Interventions

- Step 1: Analysis Type Determination
  The study clearly states:
  "In accordance with the intention-to-treat principle, analyses included all patients who were randomly assigned to receive a study drug; patients were analyzed according to initial group assignment."
  This confirms that an ITT analysis was used. Therefore, we proceed with Step 2: Effect of Assignment to Intervention.
- Step 2: Effect of Assignment to Intervention
  - 2.1 Were participants aware of their assigned intervention? → No
    The study is described as "double-blind," indicating that participants were effectively blinded to their treatment assignment.
  - 2.2 Were carers and people delivering the interventions aware of participants' assigned intervention? → No
    The double-blind design suggests that intervention providers were also blinded.

- 2.3 Were there deviations from the intended intervention that arose because of the trial context? → No
  There is no clear indication that the trial context induced deviations. Adherence was high (over 80% of patients had ≥80% adherence), and the protocol was followed throughout.
- 2.4 Were these deviations likely to have affected the outcome? → No
  Given the absence of notable deviations, there is no evidence suggesting any impact on the primary outcome.
- 2.5 Were these deviations from intended intervention balanced between groups? → Yes
  Any minor deviations or discontinuations were minimal and appeared balanced between the treatment arms.
- 2.6 Was an appropriate analysis used to estimate the effect of assignment to intervention? → Yes
  The study employed an intention-to-treat analysis, which is appropriate for preserving the benefits of randomization.
- 2.7 Was there potential for a substantial impact of the failure to analyze participants in the group to which they were randomized? → No
  Only 12 of 11,693 randomized patients did not receive treatment, a very small proportion unlikely to affect the results substantially.

- Risk Assessment:
  - Part 1 (Awareness & Deviations):
    Since both participants and personnel were not aware of the intervention (2.1 = No and 2.2 = No), and there were no meaningful deviations from the intended intervention (2.3 = No; 2.4 = No; 2.5 = Yes), Part 1 is Low risk.
  - Part 2 (Analysis Method & Impact):
    The use of an ITT analysis (2.6 = Yes) along with negligible impact from exclusions (2.7 = No) confirms Part 2 as Low risk.
  - Overall:
    As both Part 1 and Part 2 are at Low risk, the overall risk of bias for this domain is Low Risk.
- Reasoning:
  The study employed a robust ITT analysis and maintained effective double-blinding, ensuring that both participants and carers were unaware of treatment assignments. There were no significant deviations arising from the trial context that could have influenced the outcome, and any minor deviations were balanced between groups. Given that the analysis method was appropriate and any exclusions were minimal, the overall risk of bias due to deviations from the intended interventions is judged to be Low Risk.

Domain 3: Risk of Bias Due to Missing Outcome Data

- Signaling Question Analysis:
  - 3.1 Were data for this outcome available for all, or nearly all, participants randomized? → Yes
    Nearly all randomized participants were followed for the full study period. With 11,693 randomized and 11,551 completing the study (approximately 99% follow-up), missing data are minimal.
  - 3.2 Is there evidence that the result was not biased by missing outcome data? → Yes
    The study employed an intention-to-treat analysis with comprehensive follow-up, indicating that any missing data are unlikely to have biased the result.
  - 3.3 Could missingness in the outcome depend on its true value? → No
    Given the near-complete follow-up and that patients were monitored irrespective of early discontinuation, there is no indication that missingness was related to the true outcome values.

- 3.4 Is it likely that missingness in the outcome depended on its true value? → No
  The minimal and balanced missing outcome data across treatment groups suggest that missingness is unlikely to have depended on the true outcome.

- Risk Assessment: Low Risk
- Reasoning:
  Outcome data for the primary outcome (asthma exacerbation) were available for nearly all participants (Yes for 3.1), and the study provided clear evidence that the missing data did not bias the results (Yes for 3.2). There is no indication that missingness was related to the true outcome (No for 3.3 and 3.4). According to the predefined criteria, when outcome data are nearly complete and there is evidence against bias due to missing data, the risk of bias for this domain is Low Risk.

Domain 4: Risk of Bias in Measurement of the Outcome

- Signaling Question Analysis:
  - 4.1 Was the method of measuring the outcome inappropriate? → No
    The primary outcome (asthma exacerbation) was measured using a clearly defined, objective criterion (e.g., need for systemic glucocorticoids, hospitalization, or emergency department visit) that is appropriate for this type of trial.
  - 4.2 Could measurement or ascertainment of the outcome have differed between intervention groups? → No
    The study's double-blind design and standardized outcome definitions suggest that the measurement was consistent across both treatment arms.
  - 4.3 Were outcome assessors aware of the intervention received by study participants? → No
    Given the double-blind design, outcome assessors were not aware of the treatment assignments.
  - 4.4 Could assessment of the outcome have been influenced by knowledge of intervention received? → No
    Because assessors were blinded and objective criteria were used, there is little risk that knowledge of the intervention influenced the outcome assessment.
  - 4.5 Is it likely that assessment of the outcome was influenced by knowledge of intervention received? → No
    The robust blinding and objective nature of the outcome measurement make it unlikely that any bias was introduced.
- Risk Assessment: Low Risk
- Reasoning:
  The outcome measurement for asthma exacerbation was conducted using clearly defined, objective criteria that are appropriate for this trial. Measurement methods were consistent across intervention groups, and both participants and outcome assessors were blinded to treatment allocation. There is no indication that knowledge of the intervention could have influenced the outcome assessment. Based on these findings and following the predefined decision rules, the risk of bias in the measurement of the outcome is Low Risk.

Domain 5: Risk of Bias in Selection of the Reported Result

- Signaling Question Analysis:
  - 5.1 Were the data that produced this result analysed in accordance with a pre-specified analysis plan that was finalized before unblinded outcome data were available for analysis? → Yes

The study protocol, available with the full text at NEJM.org and developed in discussion with the FDA, indicates that the analysis plan was pre-specified and finalized before unblinded data were available.

    - 5.2 Is the numerical result being assessed likely to have been selected, on the basis of the results, from multiple eligible outcome measurements (e.g., scales, definitions, time points) within the outcome domain? → No
      The primary outcome (asthma exacerbation) was clearly defined in the protocol, and there is no evidence that alternative measurements or definitions were selectively reported.
    - 5.3 Is the numerical result being assessed likely to have been selected, on the basis of the results, from multiple eligible analyses of the data? → No
      The statistical analysis was pre-specified and uniformly applied (using a time-to-event Cox model within an intention-to-treat framework), with no indication of selective reporting among multiple analyses.
- Risk Assessment: Low Risk
- Reasoning:
  Since the trial was analyzed in accordance with a pre-specified analysis plan (5.1 = Yes), and there is no evidence of selective reporting of multiple outcome measurements (5.2 = No) or multiple analyses (5.3 = No), the risk of bias due to selective reporting is judged to be Low Risk. Following the predefined decision rules, when both potential sources of selective reporting are absent and the analysis was pre-specified, the overall risk of bias for this domain is Low Risk.

ID:Damião Neto 2020

First Response

Domain 1: Bias Arising from the Randomization Process

- Signaling Question Analysis:
    - Was the allocation sequence random? → Yes
      The study explicitly states that simple randomization was performed using random.org software (computer-generated random numbers).
    - Was the allocation sequence concealed? → No information
      The report does not provide any explicit details regarding the concealment of the allocation sequence.
    - Did baseline differences suggest a problem with randomization? → No
      Baseline characteristics were largely balanced, with only one difference (WHOQOL Psychological) that is not considered substantial enough to indicate a randomization problem.
- Risk Assessment: Some Concerns
- Reasoning:
    - The study provided clear evidence that the allocation sequence was generated randomly, satisfying signaling question 1.1.
    - There was no information regarding allocation concealment, leading to a "No information" rating for signaling question 1.2.
    - Baseline comparisons showed general balance between groups, with the exception of a statistically significant difference in WHOQOL Psychological, which was not deemed a substantial imbalance for the purposes of this assessment (signaling question 1.3 → No).

- According to the RoB 2.0 decision rules, when allocation concealment is rated as "No information" and baseline imbalances are not concerning (i.e., rated as No), rule 4.1 applies, resulting in an overall domain risk of Some Concerns.

Domain 2: Bias Due to Deviations from Intended Interventions

- Signaling Question Analysis:
  Step 1: Analysis Approach
  - The study reports that “analyses followed the intent-to-treat (ITT) principle,” with additional per-protocol analyses provided as sensitivity checks. For the primary outcome (anxiety immediately post-intervention), the ITT analysis is primary.
- Step 2: Effect of Assignment to Intervention (ITT Analysis)
  - 2.1 Were participants aware of their assigned intervention during the trial? → Yes
    Participants clearly knew whether they were receiving the mindfulness course or the organizational content.
  - 2.2 Were carers and people delivering the interventions aware of participants' assigned intervention during the trial? → Yes
    The intervention was delivered by a single instructor, and there is no indication that those delivering the content were blinded.
  - 2.3 Were there deviations from the intended intervention that arose because of the trial context? → No
    While adherence to home practice was low (only ~20% returned meditation diaries), this appears to reflect typical participant engagement rather than a trial-induced deviation.
  - 2.4 Were these deviations likely to have affected the outcome? → No
    Any non-adherence or deviations (e.g., low home practice) are unlikely to have materially biased the outcome measurement of anxiety, especially under an ITT framework.
  - 2.6 Was an appropriate analysis used to estimate the effect of assignment to intervention? → Yes
    The ITT analysis was employed, with missing data addressed via the last observation carried forward method.
  - 2.7 Was there potential for a substantial impact of the failure to analyze participants in the group to which they were randomized? → No
    The ITT population (141 participants) nearly encompassed the entire enrolled cohort, minimizing potential impact from exclusions.
- Risk Assessment:
  - Part 1 (Effect of Assignment to Intervention): LOW risk
    Despite participants and providers being aware of the assignments, no trial-context deviations that could bias outcomes were identified.
  - Part 2 (Analysis Method): LOW risk
    The use of ITT with appropriate handling of missing data supports a low risk judgment.
  - Overall (Part 3): LOW risk
    Since both Part 1 and Part 2 are assessed as LOW risk, the overall judgment for Domain 2 is LOW risk.
- Reasoning:
  The study’s primary analysis was conducted under the ITT principle, which minimizes bias due to deviations from intended interventions. Although both participants and intervention providers were aware of the group assignments, there is no evidence that deviations arising from this awareness (e.g., differential behavior or co-interventions) occurred in a way that would bias the primary outcome. Furthermore, the observed low adherence to home practice appears to be an

inherent characteristic of the intervention rather than a trial-specific deviation. With the appropriate ITT analysis and minimal exclusions affecting the outcome, the overall risk of bias for Domain 2 is judged as LOW risk.

Domain 3: Risk of Bias Due to Missing Outcome Data

- Signaling Question Analysis:
  - 3.1 Were data for this outcome available for all, or nearly all, participants randomized? → No
    Only about 114 out of 141 participants (≈81%) provided complete post-intervention data for the primary outcome, which is below the ≥95% threshold for continuous outcomes.
  - 3.2 Is there evidence that the result was not biased by missing outcome data? → Probably yes
    The primary analysis followed an ITT approach using LOCF for missing data, and a per protocol analysis (including only participants with complete post-test data) produced results consistent with the ITT findings, suggesting that the missing data did not materially bias the outcome estimate.
  - 3.3 Could missingness in the outcome depend on its true value? → No information
    The report does not provide sufficient details to assess whether the missingness was related to the true anxiety values.
  - 3.4 Is it likely that missingness in the outcome depended on its true value? → No information
    There is insufficient evidence to determine if missingness was dependent on the actual outcome values.
- Risk Assessment: LOW risk
- Reasoning:
  Although complete outcome data were available for only approximately 81% of randomized participants—falling short of the minimal threshold—the similar results obtained from the ITT analysis (with imputation via LOCF) and the per protocol analysis indicate that the missing data likely did not introduce bias into the effect estimate. Based on the RoB 2.0 decision rules, when there is evidence (even if only "probably yes") that missing data did not bias the result, the overall risk of bias due to missing outcome data is judged as LOW risk.

Domain 4: Risk of Bias in Measurement of the Outcome

- Signaling Question Analysis:
  - 4.1 Was the method of measuring the outcome inappropriate? → No
    The study measured anxiety using the DASS-21, a validated and appropriate self-report instrument for assessing anxiety.
  - 4.2 Could measurement or ascertainment of the outcome have differed between intervention groups? → No
    Both intervention and control groups completed the same DASS-21 questionnaire under similar conditions, indicating consistency in measurement across groups.
  - 4.3 Were outcome assessors aware of the intervention received by study participants? → Yes
    Because the outcome is self-reported, participants were aware of the intervention they received.
  - 4.4 Could assessment of the outcome have been influenced by knowledge of intervention received? → Yes
    Self-reported outcomes like anxiety are susceptible to bias if participants' responses are influenced by their knowledge of group assignment.
  - 4.5 Is it likely that assessment of the outcome was influenced by knowledge of intervention received? → Yes
    Given the subjective nature of the outcome and the absence of blinding, it is likely that participants' awareness influenced their responses.

- Risk Assessment:
  According to the RoB 2.0 decision rules:
  - The measurement method was appropriate (4.1 = No), and it was applied consistently across groups (4.2 = No).
  - However, because outcome assessors (the participants) were aware of the intervention (4.3 = Yes) and this awareness likely influenced the assessment (4.4 = Yes; 4.5 = Yes), the risk of bias is elevated.
  - Specifically, following the decision tree: when outcome assessors are aware (answer in {Y, PY, NI}) and the assessment is likely influenced by that knowledge (answer in {Y, PY, NI}), the final judgment is HIGH risk of bias.
- Reasoning:
  Although the DASS-21 is an appropriate and consistently applied measure for anxiety, its self-report nature makes it vulnerable to bias when participants know which intervention they received. The awareness of their treatment assignment, combined with the possibility that such awareness influenced their responses, leads to a high risk of bias in the measurement of the outcome.
  Final Judgment for Domain 4: HIGH risk of bias.

Domain 5: Risk of Bias in Selection of the Reported Result

- Signaling Question Analysis:
  - 5.1 Were the data that produced this result analysed in accordance with a pre-specified analysis plan finalized before unblinded data were available? → Probably yes
    The study protocol was registered on ClinicalTrials.gov (NCT03132597), which typically implies a pre-specified analysis plan was in place before data were unblinded, even though explicit confirmation of finalization timing is not detailed in the publication.
  - 5.2 Is the numerical result being assessed likely to have been selected, on the basis of the results, from multiple eligible outcome measurements within the outcome domain? → No
    The study measured anxiety using the DASS-21 and reported results for all its eligible subscales (anxiety, along with depression and stress). There is no indication that the anxiety result was selectively chosen over other measurements.
  - 5.3 Is the numerical result being assessed likely to have been selected, on the basis of the results, from multiple eligible analyses of the data? → No
    Both an ITT analysis and a per-protocol analysis were conducted and reported. The primary analysis was pre-specified (with ITT as the main approach), and the additional analysis served as a sensitivity check. There is no evidence of selective analysis reporting.
- Risk Assessment:
  Based on the RoB 2.0 decision rules:
  - Since both the potential for selective outcome measurement (5.2) and selective analysis (5.3) are answered as “No” (i.e., all eligible outcomes and analyses were reported) and there is at least a “Probably yes” for a pre-specified analysis plan (5.1), the overall judgment is LOW risk of bias.
- Reasoning:
  The study’s registration on ClinicalTrials.gov provides reasonable assurance that a pre-specified analysis plan existed before the data were unblinded, satisfying signaling question 5.1 at a “Probably yes” level. Furthermore, the study reported all relevant outcome measurements and analyses (including both ITT and per-protocol analyses) without evidence of selective reporting. Consequently, there are no indications that the numerical result (anxiety immediately

post-intervention) was chosen based on the results, leading to a final judgment of LOW risk of bias for Domain 5.

ID:Damião Neto 2020

Second Response

Domain 1: Bias Arising from the Randomization Process

- Signaling Question Analysis:
  - Was the allocation sequence random? → Y
    The study explicitly states that simple randomization was performed using random.org software.
  - Was the allocation sequence concealed? → NI
    No explicit information is provided regarding the secure concealment of the allocation sequence.
  - Did baseline differences suggest a problem with randomization? → N
    Baseline characteristics were largely balanced, with only one minor difference (WHOQOL Psychological) that does not affect the primary outcome (anxiety immediately post-intervention).
- Risk Assessment: Some Concerns
- Reasoning:
  - SQ1.1: The use of random.org confirms that the allocation sequence was random (Y).
  - SQ1.2: The study does not report any details on how the allocation sequence was concealed, resulting in a "No information" (NI) rating.
  - SQ1.3: Baseline differences were minimal and do not indicate a systematic imbalance (N).
  - Decision Rule Applied: Since the allocation concealment is rated NI and the baseline differences are rated N, Rule 4.1 applies, leading to an overall judgment of Some Concerns for Domain 1.

Domain 2: Bias Due to Deviations from Intended Interventions

- Step 1: Analysis Approach
  - The study states that analyses followed the intent-to-treat (ITT) principle (using last observation carried forward) and additionally reports a per protocol analysis for sensitivity.
  - Classification: ITT is the primary analysis, meaning we evaluate the effect of assignment to intervention (Step 2).
- Signaling Question Analysis (Effect of Assignment to Intervention):
  - 2.1 Were participants aware of their assigned intervention during the trial? → Yes
    Participants were informed of their course content (mindfulness vs. organizational topics), so they clearly knew which group they were in.
  - 2.2 Were carers and people delivering the interventions aware of participants' assigned intervention during the trial? → Yes
    The instructor delivered the mindfulness course, and the control group received a different content session, indicating that intervention providers were aware of group assignments.
  - 2.3 Were there deviations from the intended intervention that arose because of the trial context? → No
    Both groups received their assigned courses as scheduled. Although home practice adherence was low in the intervention group, this reflects real-world engagement rather than a trial-induced deviation.

    - 2.4 Were these deviations likely to have affected the outcome? → No
      There is no evidence that any deviations (e.g., low adherence to home practice) substantially influenced the primary outcome (anxiety immediately post-intervention).
    - 2.5 Were these deviations from intended intervention balanced between groups? → Yes
      There is no indication of imbalanced deviations; both groups adhered to their respective course formats as planned.
    - 2.6 Was an appropriate analysis used to estimate the effect of assignment to intervention? → Yes
      The primary analysis was conducted under the ITT principle (with last observation carried forward), and exclusions in the per protocol analysis were solely due to missing post-test data.
    - 2.7 Was there potential for a substantial impact of the failure to analyse participants in the group to which they were randomized? → No
      The ITT analysis included all randomized participants, and any exclusions were minimal and attributable to missing outcome data.
- Risk Assessment:
    - Part 1 (Effect of Assignment to Intervention):
      Since both participants and personnel were aware of the intervention (Y), we examined deviations. With no trial-induced deviations (2.3 = No) and no evidence that any deviations affected the outcome (2.4 = No), Part 1 is judged as LOW risk.
    - Part 2 (Appropriate Analysis):
      An ITT analysis was appropriately applied (2.6 = Yes) with minimal impact from exclusions (2.7 = No), so Part 2 is also LOW risk.
    - Overall Judgment:
      As both Part 1 and Part 2 are LOW risk, the final judgment for Domain 2 is LOW risk.
- Reasoning:
    - Although both participants and intervention providers were aware of group assignments (a common scenario in behavioral interventions), there were no deviations induced by the trial context that could bias the primary outcome.
    - The ITT approach was appropriately employed, with exclusions limited to missing outcome data, thereby preserving the benefits of randomization.
    - Given that any deviations (such as low adherence to home practice) were unlikely to have influenced anxiety measured immediately post-intervention—and were balanced between groups—the risk of bias due to deviations from intended interventions is judged as LOW risk.

Domain 3: Risk of Bias Due to Missing Outcome Data

- Signaling Question Analysis:
    - 3.1 Were data for this outcome available for all, or nearly all, participants randomized? → N
      Only about 114 out of 141 participants (≈80.8%) provided post-intervention data for anxiety, which is below the ≥95% threshold for continuous outcomes.
    - 3.2 Is there evidence that the result was not biased by missing outcome data? → No
      The report does not present clear evidence (e.g., sensitivity analyses) to confirm that missing data did not bias the outcome results.

- 3.3 Could missingness in the outcome depend on its true value? → No information
  The study does not provide sufficient details regarding the reasons for missing post-intervention anxiety data to determine if missingness is related to the actual anxiety levels.
- 3.4 Is it likely that missingness in the outcome depended on its true value? → No information
  There is inadequate information to assess whether the missing outcome data might be related to participants' true anxiety scores.

- Risk Assessment: HIGH risk of bias
- Reasoning:
  - Since outcome data were available for only about 80.8% of participants (SQ3.1 = N), we cannot consider the missing data trivial.
  - There is no evidence provided that the missing data did not bias the results (SQ3.2 = No).
  - With insufficient information to determine whether the missingness is related to true anxiety values (SQ3.3 and SQ3.4 = No information), following the RoB 2.0 decision rules, the likelihood that missingness could depend on the true outcome is treated as potentially problematic.
  - Based on these findings and applying the decision rules (specifically, rule 2.2.2.2: if missingness could depend on the true value in {Y, PY, NI}, then HIGH risk of bias is assigned), the domain is judged to be at HIGH risk of bias.

Domain 4: Risk of Bias in Measurement of the Outcome

- Signaling Question Analysis:
  - 4.1 Was the method of measuring the outcome inappropriate? → No
    The outcome (anxiety) was measured using the DASS21, a validated and appropriate self-report instrument for assessing anxiety in clinical and non-clinical populations.
  - 4.2 Could measurement or ascertainment of the outcome have differed between intervention groups? → No
    Both the intervention and control groups used the same DASS21 questionnaire at similar time points, ensuring consistency in measurement across groups.
  - 4.3 Were outcome assessors aware of the intervention received by study participants? → Yes
    Because the outcome is self-reported, participants (acting as their own assessors) were aware of the intervention they received.
  - 4.4 Could assessment of the outcome have been influenced by knowledge of intervention received? → Yes
    Self-report measures, particularly when participants know their group assignment, are susceptible to bias due to expectations or perceived benefits.
  - 4.5 Is it likely that assessment of the outcome was influenced by knowledge of intervention received? → Probably yes
    Given that participants were not blinded and provided self-reported data, it is likely that their responses could have been influenced by their awareness of receiving either the mindfulness intervention or the control content.
- Risk Assessment: HIGH risk of bias
- Reasoning:
  - Although the measurement method (DASS21) is appropriate (4.1 = No) and was applied consistently across both groups (4.2 = No), the fact that the outcome is self-reported means that participants were aware of their intervention assignment (4.3 = Yes).

    - This awareness creates a potential for the assessment to be influenced by expectations or other subjective factors (4.4 = Yes, 4.5 = Probably yes).
    - Following the RoB 2.0 decision rules—specifically, when outcome assessors (participants) are aware and their assessments could be influenced by that awareness, the risk of bias is judged as HIGH risk—the overall judgment for Domain 4 is HIGH risk of bias.

Domain 5: Risk of Bias in Selection of the Reported Result

- Signaling Question Analysis:
    - 5.1 Were the data that produced this result analysed in accordance with a pre-specified analysis plan? → Probably yes
      The trial was registered (NCT03132597) and the methods state that analyses followed the intent-to-treat principle (with both ITT and per-protocol analyses reported). Although explicit confirmation that the analysis plan was finalized before unblinding is not detailed, the trial registration suggests that a pre-specified plan was in place.
    - 5.2 Is the numerical result being assessed likely to have been selected, on the basis of the results, from multiple eligible outcome measurements within the outcome domain? → No
      The primary outcome (anxiety immediately post-intervention) was measured using the DASS-21 anxiety subscale, and there is no evidence that multiple eligible measurements (e.g., different scales or time points) were selectively reported.
    - 5.3 Is the numerical result being assessed likely to have been selected, on the basis of the results, from multiple eligible analyses of the data? → No
      Both ITT and per-protocol analyses are reported, and there is no indication that the primary result was selectively chosen from among various analyses.
- Risk Assessment: LOW risk of bias
- Reasoning:
    - The trial appears to have been analysed according to a pre-specified plan (5.1 = Probably yes), as indicated by its registration and stated ITT approach.
    - There is no indication that multiple eligible outcome measurements (5.2 = No) or multiple analyses (5.3 = No) were used selectively to report the numerical result for anxiety.
    - Applying the decision rule—if both multiple outcome measurements and multiple analyses are rated as No (or PN) and the trial followed a pre-specified plan (Y/PY)—the overall judgment is LOW risk of bias.

ID:Omrani 2020

First Response

Domain 1: Bias Arising from the Randomization Process

- Signaling Question Analysis:
    - Was the allocation sequence random? → Y
      The study used computer-generated randomization executed remotely, indicating explicit evidence of a random allocation sequence.

- Was the allocation sequence concealed? → Y
  Central allocation concealment was implemented with identical-appearing, sequentially numbered drug-bottle sets, ensuring secure concealment.
- Did baseline differences suggest a problem with randomization? → N
  Baseline characteristics across groups (as reported in Table 1) were well balanced, showing no substantial imbalances.

- Risk Assessment: Low Risk
- Reasoning:
  - The allocation sequence was generated by a computer (Y) and was securely concealed (Y).
  - There were no significant baseline imbalances (N).
  - Per the RoB 2.0 decision rules, when the allocation sequence is random and properly concealed with no baseline imbalances, the risk of bias in this domain is rated as Low Risk.

Domain 2: Bias Due to Deviations from Intended Interventions

- Analysis Method:
  The study primarily employed an intention-to-treat (ITT) analysis. Although a per-protocol analysis was also conducted for exploratory purposes, the main analysis and outcome assessments are based on ITT, meaning that participants were analyzed in the groups to which they were randomized despite some withdrawals.
- Signaling Question Analysis (Effect of Assignment to Intervention):
  - 2.1 Were participants aware of their assigned intervention? → No
    Participants were blinded to their study medication as the trial was triple-blinded.
  - 2.2 Were carers and people delivering the interventions aware of participants' assigned intervention? → No
    Study staff, including those administering treatments and assessing outcomes, were blinded.
  - 2.3 Were there deviations from the intended intervention that arose because of the trial context? → Yes
    Some participants (4.8% overall) withdrew after receiving at least one day of study medication due to reasons such as unspecific withdrawal requests, asymptomatic QT prolongation, or lab abnormalities. In addition, a protocol deviation regarding the QT-monitoring cut-off was identified and then corrected during the trial.
  - 2.4 Were these deviations likely to have affected the outcome? → No
    The withdrawals and protocol adjustments were relatively few, balanced across groups, and the primary outcome (virologic cure) is not directly influenced by the QT monitoring process.
  - 2.5 Were these deviations balanced between groups? → Yes
    The number of withdrawals was similar across the three groups (HC+AZ, HC, and placebo), indicating balanced deviations.
  - 2.6 Was an appropriate analysis used to estimate the effect of assignment to intervention? → Yes
    The ITT analysis was applied, and sensitivity analyses were conducted to assess the robustness of the findings, ensuring that exclusions did not bias the results.
  - 2.7 Was there potential for a substantial impact of the failure to analyze participants in the group to which they were randomized? → No
    The relatively low dropout rate and the balanced distribution of deviations suggest that any impact on the results is minimal.
- Risk Assessment:

- Part 1 (Effect of Assignment):
  Since both participants and intervention providers were effectively blinded (answers for 2.1 and 2.2 are "No"), Part 1 is judged as LOW risk.
- Part 2 (Appropriate Analysis):
  With the use of ITT analysis and supportive sensitivity analyses (answer for 2.6 is "Yes" and 2.7 is "No"), Part 2 is judged as LOW risk.
- Overall Judgment for Domain 2:
  When combining both parts—Part 1 = LOW risk and Part 2 = LOW risk—the overall risk of bias due to deviations from intended interventions is LOW risk.

- Reasoning:
  The effective blinding of participants and personnel minimizes the chance that knowledge of intervention assignment influenced behaviors or treatment administration. Although there were some deviations (withdrawals and a protocol adjustment regarding QT monitoring), these were small in number, balanced across groups, and did not appear to have a substantial effect on the primary outcome. Furthermore, the use of an ITT analysis with confirmatory sensitivity analyses ensures that any deviations from the protocol do not introduce bias into the estimation of the treatment effect.
  Overall, the trial's approach to handling deviations from the intended interventions supports a LOW risk judgment for Domain 2.

Domain 3: Risk of Bias Due to Missing Outcome Data

- Signaling Question Analysis:
  - 3.1 Were data for this outcome available for all, or nearly all, participants randomized? → Yes
    All-cause mortality (the outcome of interest) was captured for all (or nearly all) participants, with no evidence of substantial missing data. The trial reported "no deaths," indicating complete outcome ascertainment.
  - 3.2 Is there evidence that the result was not biased by missing outcome data? → Yes
    There is clear evidence that missing outcome data did not bias the results, as nearly 100% of participants had mortality data and sensitivity analyses confirmed minimal impact from missing data.
  - 3.3 Could missingness in the outcome depend on its true value? → No
    Given that all participants' vital status was ascertained and no deaths occurred, there is no indication that missingness would depend on the true outcome value.
  - 3.4 Is it likely that missingness in the outcome depended on its true value? → No
    The outcome data for mortality is robust and complete, so it is unlikely that any missing data (if present) would be related to the true outcome value.
- Risk Assessment: LOW risk of bias
- Reasoning:
  Since outcome data for all-cause mortality at day 28 are available for virtually all participants (3.1 = Yes) and there is clear evidence that missing data did not introduce bias (3.2 = Yes), with no indication that missingness depended on the outcome (3.3 and 3.4 = No), the risk of bias due to missing outcome data is judged as LOW risk according to the RoB 2.0 guidelines.

Domain 4: Risk of Bias in Measurement of the Outcome

- Signaling Question Analysis:

- 4.1 Was the method of measuring the outcome inappropriate? → No
  The outcome—all-cause mortality at day 28—is objectively measured through vital status, making the method appropriate.
- 4.2 Could measurement or ascertainment of the outcome have differed between intervention groups? → No
  Measurement of mortality is standardized and consistent across groups in this trial.
- 4.3 Were outcome assessors aware of the intervention received by study participants? → No
  The trial was triple-blinded, ensuring that outcome assessors were not aware of the intervention assignments.
- 4.4 Could assessment of the outcome have been influenced by knowledge of intervention received? → No
  Given the objective nature of mortality data and effective blinding, it is highly unlikely that the outcome assessment was influenced by intervention knowledge.
- 4.5 Is it likely that assessment of the outcome was influenced by knowledge of intervention received? → No
  There is no indication that knowledge of the intervention affected how mortality was ascertained.

- Risk Assessment: LOW risk
- Reasoning:
  The method of measuring all-cause mortality is inherently objective and appropriate, and there is strong evidence of measurement consistency across groups. With outcome assessors being blinded due to the trial's triple-blinded design, the risk that assessment was influenced by intervention knowledge is minimal. Consequently, the risk of bias in the measurement of the outcome is judged as LOW risk.

Domain 5: Risk of Bias in Selection of the Reported Result

- Signaling Question Analysis:
  - 5.1 Were the data that produced this result analysed in accordance with a pre-specified analysis plan? → Probably yes
    Trial registration (NCT04349592) and protocol details indicate that an analysis plan was established a priori. Although explicit confirmation that the plan was finalized before unblinding is not directly stated, the available evidence supports that a pre-specified plan was in place.
  - 5.2 Is the numerical result being assessed likely to have been selected from multiple eligible outcome measurements? → No
    The outcome—all-cause mortality at day 28—is measured in a straightforward, objective manner with no alternative scales or time points reported. There is no evidence of selective reporting among different definitions or measurements for mortality.
  - 5.3 Is the numerical result being assessed likely to have been selected from multiple eligible analyses of the data? → No
    There is no indication that multiple analytic approaches were available or selectively chosen for reporting the mortality outcome. The analysis appears to follow the pre-specified plan without evidence of selective analysis.
- Risk Assessment: LOW risk of bias
- Reasoning:
  The trial appears to have followed a pre-specified analysis plan (5.1: Probably yes) and, for the outcome of all-cause mortality at day 28, there is no indication that multiple measurements or analyses were available from which to selectively report a favorable result (5.2 and 5.3: No). Based on the RoB 2.0 decision rules, when both the potential for selective outcome measurement and selective analysis is minimal, and the analysis is guided by a pre-specified plan, the

risk of bias in the selection of the reported result is judged as LOW risk.

ID: Omrani 2020

Second Response

Domain 1: Bias Arising from the Randomization Process

- Signaling Question Analysis:
  - Was the allocation sequence random? → Yes
    The study explicitly reports that randomization was executed by computer in a remote location (Imperial College London), providing clear evidence of a random allocation sequence.
  - Was the allocation sequence concealed? → Yes
    Central allocation concealment was used. The randomization scheme was sent to the central pharmacy, which then prepared identical-appearing sequentially numbered drug-bottle sets. This demonstrates secure concealment.
  - Did baseline differences suggest a problem with randomization? → No
    Baseline characteristics (e.g., age, baseline Ct values, nationality distributions) are similar across the groups, indicating no substantial imbalances.
- Risk Assessment: Low risk
- Reasoning:
  - The allocation sequence was explicitly randomized and executed by computer, confirming its randomness.
  - Secure allocation concealment was maintained via central pharmacy procedures and the use of identical-appearing study medications.
  - Baseline data show no significant differences among the intervention groups.
  - According to the RoB 2.0 decision rules, when the allocation sequence is concealed (Yes), the sequence is random (Yes), and there are no baseline imbalances (No), the overall risk of bias for this domain is Low risk.

Domain 2: Bias Due to Deviations from Intended Interventions

- Step 1: Analysis Approach
  - The study's primary analysis was conducted on an intent-to-treat (ITT) basis, with a per-protocol analysis performed only for exploratory assessments.
  - Classification: ITT (effect of assignment to intervention)
- Step 2: Effect of Assignment to Intervention
  - 2.1 Were participants aware of their assigned intervention? → No
    The study was triple-blinded; participants were not aware of their treatment assignment.
  - 2.2 Were carers and people delivering the interventions aware of participants' assigned intervention? → No
    Blinding extended to study staff (physicians, nurses, and analysts) who managed treatment and outcome assessments.
  - 2.3 Were there deviations from the intended intervention that arose because of the trial context? → No
    Although 22 participants (4.8%) withdrew after receiving at least one day of study medication, these

withdrawals were minor and reflect expected trial occurrences rather than systematic deviations introduced by the trial context.

  - 2.4 Were these deviations likely to have affected the outcome? → No
    Withdrawals were minimal and balanced across study groups, and sensitivity analyses confirmed that these deviations did not impact the primary outcome.
  - 2.5 Were these deviations balanced between groups? → Yes
    Withdrawal rates were similar across arms (HC: 4.0%, HC+AZ: 4.6%, Placebo: 5.9%; $p = 0.716$), indicating balance.
  - 2.6 Was an appropriate analysis used to estimate the effect of assignment to intervention? → Yes
    The study employed an ITT analysis for its primary endpoint, with sensitivity analyses supporting the robustness of the approach.
  - 2.7 Was there potential for a substantial impact of the failure to analyze participants in the group to which they were randomized? → No
    The minimal and balanced exclusions do not suggest a substantial impact on the results.
- Risk of Bias Judgment
  - Part 1 (Effect of Assignment):
    - Participants and personnel were blinded (2.1 and 2.2: No).
    - Deviations did not arise from trial context (2.3: No), did not affect the outcome (2.4: No), and were balanced across groups (2.5: Yes).
    - Result: LOW risk.
  - Part 2 (Analysis Approach):
    - An appropriate ITT analysis was used (2.6: Yes) and the exclusions did not substantially impact results (2.7: No).
    - Result: LOW risk.
- Overall for Domain 2:
  - Since both Part 1 and Part 2 are judged LOW risk, the overall risk of bias due to deviations from intended interventions is Low risk.
- Reasoning Summary:
  - The study adhered to an ITT approach, ensuring that all randomized participants with available outcome data were analyzed according to their assigned groups.
  - Effective triple-blinding minimized awareness among participants and personnel.
  - Withdrawals (4.8% overall) were few, balanced across arms, and did not impact the outcome per sensitivity analyses.
  - Based on the RoB 2.0 decision rules, these factors collectively yield a Low risk rating for Domain 2.

Domain 3: Risk of Bias Due to Missing Outcome Data

- Signaling Question Analysis:
  - 3.1 Were data for this outcome available for all, or nearly all, participants randomized? → Yes
    Outcome data were available for nearly all participants. For example, day six virologic cure data were obtained for 152/152 (100%) in the HC+AZ group, 149/152 (98.0%) in the HC group, and 147/152 (96.7%) in the placebo group.

- 3.2 Is there evidence that the result was not biased by missing outcome data? → Yes
  Sensitivity analyses were conducted that confirmed the primary outcome results remained non-significant regardless of assumptions about missing data, indicating that any missing data did not bias the result.
- 3.3 Could missingness in the outcome depend on its true value? → No
  There is no indication from the report that the missing outcome data were related to the true outcome values.
- 3.4 Is it likely that missingness in the outcome depended on its true value? → No
  The minimal and balanced missing data across groups suggest that missingness was unlikely to be related to the actual outcome.

- Risk Assessment: Low risk
- Reasoning:
  - The trial achieved nearly complete follow-up with outcome data for almost all participants.
  - Sensitivity analyses demonstrate that the missing data did not influence the estimated effect.
  - There is no evidence that the missingness depended on the true outcome values.
  - According to the RoB 2.0 rules, when outcome data are available for nearly all participants and there is evidence that missing data did not bias the results, the risk of bias due to missing outcome data is Low risk.

Domain 4: Risk of Bias in Measurement of the Outcome

- Signaling Question Analysis:
  - 4.1 Was the method of measuring the outcome inappropriate? → No
    The outcome (virologic cure) was measured using standard PCR testing with a predefined cycle threshold, which is an appropriate and objective method.
  - 4.2 Could measurement or ascertainment of the outcome have differed between intervention groups? → No
    The same PCR testing methods and protocols were used consistently across all intervention groups.
  - 4.3 Were outcome assessors aware of the intervention received by study participants? → No
    The study was triple-blinded, meaning that outcome assessors (including laboratory personnel and study staff involved in outcome measurement) were unaware of participants' treatment assignments.
  - 4.4 Could assessment of the outcome have been influenced by knowledge of intervention received? → No
    Given the objective nature of the PCR testing and effective blinding, it is highly unlikely that the outcome assessment was influenced by knowledge of the intervention.
  - 4.5 Is it likely that assessment of the outcome was influenced by knowledge of intervention received? → No
    There is no indication or evidence to suggest that outcome assessment was affected by any awareness of the intervention.
- Risk Assessment: Low risk
- Reasoning:
  - The method of outcome measurement (PCR testing) is clearly appropriate and standardized.
  - Measurement procedures were consistent across all groups, and the outcome assessors were effectively blinded.
  - There is no evidence suggesting that the assessment was influenced by knowledge of the intervention.
  - Based on the RoB 2.0 decision rules, these factors collectively indicate a Low risk of bias in the measurement of the outcome.

Domain 5: Risk of Bias in Selection of the Reported Result

- Signaling Question Analysis:
  - 5.1 Were the data that produced this result analysed in accordance with a pre-specified analysis plan that was finalized before unblinded outcome data were available for analysis? → Yes
    The trial was registered (ClinicalTrials.gov NCT04349592) and a protocol was publicly available prior to unblinding. The pre-specified analysis plan, including details on the primary (day six virologic cure) and secondary outcomes as well as the interim analysis procedures, was finalized before outcome data were available.
  - 5.2 Is the numerical result being assessed likely to have been selected, on the basis of the results, from multiple eligible outcome measurements within the outcome domain? → No
    The trial defined a clear primary outcome (virologic cure at day six) and reported it along with the pre-specified secondary outcome (day 14 virologic cure) without evidence of selective outcome measurement. All eligible outcomes appear to have been reported as per the protocol.
  - 5.3 Is the numerical result being assessed likely to have been selected, on the basis of the results, from multiple eligible analyses of the data? → No
    The analyses, including the primary ITT analysis and supportive sensitivity analyses, were conducted according to the pre-specified plan with no indication of selective reporting of alternative analyses.
- Risk Assessment: Low risk
- Reasoning:
  - The trial was conducted in strict accordance with a pre-specified analysis plan that was finalized before unblinding, as evidenced by the registration and protocol details.
  - There is no indication of selective reporting from multiple outcome measurements or multiple analyses; all planned outcomes and analyses are reported.
  - According to the RoB 2.0 decision rules, with a confirmed pre-specified analysis plan (Yes) and no evidence of selective reporting (both 5.2 and 5.3 rated as No), the overall risk of bias in the selection of the reported result is Low risk.

ID: Erogul 2014

First Response

Domain 1: Bias Arising from the Randomization Process

- Signaling Question Analysis:
  - 1.1 Was the allocation sequence random? → Yes
    The study clearly states that a random number generator was used to assign participants based on their alphabetical order, providing explicit evidence of randomization.
  - 1.2 Was the allocation sequence concealed? → No information
    The report does not describe any procedures (e.g., sealed envelopes or independent assignment) that ensure allocation concealment, so no explicit evidence is provided.
  - 1.3 Did baseline differences suggest a problem with randomization? → No
    Baseline characteristics across the groups were reported to be comparable, with no substantial imbalances detected.
- Risk Assessment: SOME CONCERNS

- Reasoning:
  - The allocation sequence was explicitly randomized (SQ1.1: Yes).
  - However, there is no information on whether the allocation sequence was concealed (SQ1.2: NI).
  - Baseline characteristics did not show any significant differences (SQ1.3: No).
  - Decision Rule Applied: According to rule 4.1, if the allocation sequence concealment is rated as "No information" and baseline imbalances are "No," the risk of bias is classified as SOME CONCERNS.

  No inconsistencies were detected in the assessment relative to the predefined RoB 2.0 rules.

Domain 2: Bias Due to Deviations from Intended Interventions

- Step 1: Analysis Method Evaluation
  - ITT/PP Determination:
    Although the report does not explicitly state "intention-to-treat," nearly all randomized participants (with one early dropout for scholastic reasons) were included in the analysis via linear mixed models. This is consistent with a modified ITT approach, and exclusions were not driven by non-adherence.
- Step 2: Effect of Assignment to Intervention
  - 2.1 Were participants aware of their assigned intervention? → Yes
    Medical students clearly knew whether they were assigned to receive the mindfulness intervention or to control.
  - 2.2 Were carers and people delivering the interventions aware of participants' assigned intervention? → Yes
    The intervention was delivered by a licensed psychotherapist, who was aware of group assignment.
  - 2.3 Were there deviations from the intended intervention that arose because of the trial context? → Yes
    The protocol prescribed a minimum of 140 minutes of home meditation per week; however, the treatment group averaged only about 40.7 minutes per week. This deviation appears to stem from the trial context (e.g., the busy schedules of first-year medical students).
  - 2.4 Were these deviations likely to have affected the outcome? → No
    Despite the lower-than-prescribed home meditation, the primary outcome (stress immediately post-intervention) still demonstrated a significant reduction. The deviation likely attenuated—but did not differentially bias—the treatment effect.
  - 2.5 Were these deviations from intended intervention balanced between groups? → Yes
    There is no indication that similar deviations (or cross-over to mindfulness practices) occurred in the control group.
  - 2.6 Was an appropriate analysis used to estimate the effect of assignment to intervention? → Yes
    The study employed linear mixed models that included nearly all randomized participants, consistent with an ITT (or modified ITT) analysis.
  - 2.7 Was there potential for a substantial impact of failing to analyze participants in the group to which they were randomized? → No
    Only one dropout occurred for reasons unrelated to the intervention, and the analysis appears robust to this minimal loss.
- Risk Assessment for Effect of Assignment (Part 1 & Part 2):
  - Part 1 (Effect of Assignment to Intervention):
    Since both participants and intervention providers were aware of group assignments—and because deviations

from the intended intervention arose (SQ 2.3) but were judged unlikely to affect the outcome (SQ 2.4)—this part is assessed as SOME CONCERNS.

  - Part 2 (Appropriate Analysis of Assignment):
    With an analysis method consistent with ITT principles (SQ 2.6) and minimal exclusions (SQ 2.7 = No substantial impact), this part is judged as LOW risk.

- Final Risk Judgment for Domain 2:
  - Overall Risk: SOME CONCERNS
    Because Part 1 indicates SOME CONCERNS while Part 2 is LOW risk—and neither part reaches HIGH risk—the overall judgment for deviations from intended interventions is SOME CONCERNS.

No logical inconsistencies were detected relative to the predefined RoB 2.0 rules.

Domain 3: Risk of Bias Due to Missing Outcome Data

- Signaling Question Analysis:

  - 3.1 Were data for this outcome available for all, or nearly all, participants randomized? → Yes
    Only one participant was lost from the initial sample (final N=57), representing well over 95% retention for a continuous outcome like the PSS.

  - 3.2 Is there evidence that the result was not biased by missing outcome data? → Yes
    There is no indication that the minimal missing data introduced bias, and appropriate statistical methods (linear mixed models) were used.

  - 3.3 Could missingness in the outcome depend on its true value? → No
    The single dropout was due to scholastic reasons, not because of the outcome (stress level).

  - 3.4 Is it likely that missingness in the outcome depended on its true value? → No
    There is no evidence suggesting that the missing data are related to the participants' stress levels.

- Risk Assessment: LOW risk of bias

- Reasoning:

  - Outcome data were available for nearly all randomized participants (SQ 3.1: Yes).

- There is clear evidence that the minimal missing data did not bias the results (SQ 3.2: Yes).

- The dropout does not appear to be related to the outcome values (SQ 3.3 and 3.4: No).

- Decision Rule Applied: Since outcome data for nearly all participants is available, the risk of bias is classified as LOW.

No logical inconsistencies were detected relative to the predefined RoB 2.0 rules.

Domain 4: Risk of Bias in Measurement of the Outcome

- Signaling Question Analysis:
  - 4.1 Was the method of measuring the outcome inappropriate? → No
    The study used the Perceived Stress Scale—a well-validated, widely used instrument—for measuring stress, making the measurement method appropriate.
  - 4.2 Could measurement or ascertainment of the outcome have differed between intervention groups? → No
    Both the intervention and control groups completed the same self-report questionnaire under similar conditions, ensuring consistency in measurement across groups.
  - 4.3 Were outcome assessors aware of the intervention received by study participants? → Yes
    Because the outcome was self-reported, participants (who serve as their own assessors) were aware of the intervention they received.
  - 4.4 Could assessment of the outcome have been influenced by knowledge of intervention received? → Yes
    Given that the PSS is subjective and based on self-report, knowing the group assignment could influence participants' responses.
  - 4.5 Is it likely that assessment of the outcome was influenced by knowledge of intervention received? → Yes
    It is likely that participants' perceptions of stress were affected by their awareness of receiving the mindfulness intervention.
- Risk Assessment: HIGH risk of bias
- Reasoning:
  - Although the measurement instrument was appropriate (4.1: No) and applied consistently across groups (4.2: No), the fact that outcome assessors (the participants themselves) were aware of their intervention (4.3: Yes) raises concern.
  - This awareness could have influenced their self-reported stress levels (4.4: Yes) and is considered likely (4.5: Yes).
  - According to the RoB 2.0 decision rules, when assessment is influenced by knowledge of intervention (falling into the {Y, PY, NI} category), the overall risk of bias in measurement is determined to be HIGH risk.

  No logical inconsistencies were detected relative to the predefined RoB 2.0 rules.

Domain 5: Risk of Bias in Selection of the Reported Result

- Signaling Question Analysis:

- 5.1 Were the data that produced this result analysed in accordance with a pre-specified analysis plan? → No information
  The article does not provide explicit details regarding a pre-specified analysis plan or indicate that the plan was finalized before unblinded outcome data were available.
- 5.2 Is the numerical result being assessed likely to have been selected from multiple eligible outcome measurements? → No
  For the primary outcome (stress immediately post-intervention measured with the Perceived Stress Scale), the result appears to have been reported as planned without evidence of selective measurement among multiple alternatives.
- 5.3 Is the numerical result being assessed likely to have been selected from multiple eligible analyses of the data? → No information
  The report does not provide details on whether alternative analyses were performed or selectively reported, leaving this aspect unclear.

- Risk Assessment: SOME CONCERNS
- Reasoning:
  - The absence of explicit information about a pre-specified analysis plan (5.1: No information) means we cannot confirm that the reported result was analyzed as originally intended.
  - There is no evidence of selective reporting among multiple outcome measurements (5.2: No), but the uncertainty about multiple analyses (5.3: No information) raises concerns.
  - Decision Rule Applied: Because at least one component (multiple analyses of data) is rated as "No information" and neither component indicates clear pre-specification (i.e., none rated as Y or PY), the overall risk of bias in this domain is classified as SOME CONCERNS.

No logical inconsistencies were detected relative to the predefined RoB 2.0 rules.

ID: Erogul 2014

Second Response

Domain 1: Bias Arising from the Randomization Process

- Signaling Question Analysis:
  - Was the allocation sequence random? → Y
    The study explicitly states that a random number generator was used to select participants based on their alphabetical order.
  - Was the allocation sequence concealed? → NI
    The report does not provide explicit details regarding secure concealment of the allocation sequence.
  - Did baseline differences suggest a problem with randomization? → N
    Baseline characteristics (age, sex, PSS, SCS, RS scores) showed no significant differences between groups.
- Risk Assessment: Some Concerns
- Reasoning:
  - The allocation sequence was generated randomly (Y), which supports a low risk.
  - However, the study does not provide explicit evidence about the concealment of the allocation sequence (NI).
  - Since there are no baseline imbalances (N), the RoB 2.0 decision rule for an allocation concealment of No Information with no baseline imbalances applies, resulting in an overall risk judgment of Some Concerns.

Domain 2: Bias Due to Deviations from Intended Interventions

- Step 1: Analysis Type Determination
  - Analysis Approach:
    The study excluded one participant who dropped out for scholastic reasons. There is no indication that exclusions were made for reasons such as non-adherence to the intervention apart from this dropout. In effect, the analysis resembles a modified intention-to-treat (mITT) approach, with exclusions solely due to missing outcome data.
- Step 2: Effect of Assignment to Intervention
  - 2.1 Were participants aware of their assigned intervention? → Y
    The trial is described as unblinded, so participants were clearly aware of their group assignment.
  - 2.2 Were carers and people delivering the interventions aware of participants' assigned intervention? → Y
    Given the unblinded nature, intervention providers (e.g., the mindfulness instructor) were aware of the group assignments.
  - 2.3 Were there deviations from the intended intervention that arose because of the trial context? → N
    Although the treatment group did not fully adhere to the prescribed home meditation time (mean minutes per week were lower than intended), this deviation reflects real-world adherence challenges rather than a deviation introduced by the trial setting itself.
  - 2.4 Were these deviations likely to have affected the outcome? → N
    There is no clear evidence that the reduced home meditation adherence introduced a systematic bias in the outcome measurement (stress levels). The observed improvement in the primary outcome was still detected despite lower-than-intended adherence.
  - 2.5 Were these deviations from intended intervention balanced between groups? → N
    Only the treatment group had an intervention (and hence a target adherence), so the issue of balance is inherent to the design rather than a differential deviation between groups.
  - 2.6 Was an appropriate analysis used to estimate the effect of assignment to intervention? → Y
    The study used linear mixed models with adjustments for relevant covariates, which is appropriate. The exclusion (one dropout) appears to be limited and justified as missing outcome data.
  - 2.7 Was there potential for a substantial impact of failing to analyze participants in the group to which they were randomized? → N
    With only one dropout and the use of an analysis method that accommodates missing data, any potential impact is minimal.
- Risk Assessment for Step 2:
  - Part 1 (Awareness/Deviations):
    Although both participants and intervention providers were aware of the assignments, no deviations attributable to the trial context were identified (2.3 = N and 2.4 = N).
    → Part 1 = LOW risk.
  - Part 2 (Analysis):
    An appropriate ITT-like (mITT) analysis was performed (2.6 = Y) and the failure to include one participant does not have a substantial impact (2.7 = N).
    → Part 2 = LOW risk.

- Combined Judgment for Effect of Assignment:
  Since both Part 1 and Part 2 are LOW risk, the overall risk for the effect of assignment to intervention is LOW risk.

- Final Risk Assessment for Domain 2: LOW risk
- Reasoning:
  - The study's modified ITT approach (with minimal and justified exclusions) appropriately captures the effect of assignment to intervention.
  - Although the trial was unblinded, the deviations in adherence (e.g., lower home meditation time) appear to reflect real-world participant behavior rather than trial-induced biases.
  - The analytical method was suitable, and any deviations did not substantially impact the primary outcome (stress immediately post-intervention).

  Thus, the risk of bias due to deviations from intended interventions for the primary outcome is judged to be LOW risk.

Domain 3: Risk of Bias Due to Missing Outcome Data

- Signaling Question Analysis:
  - Were data for this outcome available for all, or nearly all, participants randomized? → Yes
    The study reports outcome data for nearly all randomized participants (with only one participant dropping out, which yields ≥95% available data for a continuous outcome).
  - Is there evidence that the result was not biased by missing outcome data? → Yes
    The minimal missing data suggest that any potential bias is unlikely to have meaningfully affected the estimated effect.
  - Could missingness in the outcome depend on its true value? → No
    The single dropout was due to scholastic reasons, not because of the outcome (stress levels).
  - Is it likely that missingness in the outcome depended on its true value? → No
    There is no indication that the missing data were related to the participants' stress levels.
- Risk Assessment: LOW risk
- Reasoning:
  - Outcome data were available for nearly all participants, meeting the ≥95% threshold for continuous outcomes.
  - The minimal missing data (only one dropout) and the dropout reason (scholastic, not outcome-related) provide clear evidence that the results were not biased by missing outcome data.
  - There is no indication that missingness depended on the true outcome values.

Thus, the overall risk of bias due to missing outcome data is judged to be LOW risk.

Domain 4: Risk of Bias in Measurement of the Outcome

- Signaling Question Analysis:
  - 4.1 Was the method of measuring the outcome inappropriate? → No
    The Perceived Stress Scale (PSS-10) is a well-validated and appropriate instrument for assessing stress.
  - 4.2 Could measurement or ascertainment of the outcome have differed between intervention groups? → No
    Both groups completed the same standardized PSS-10 instrument under similar conditions (completed simultaneously in different rooms), ensuring consistency across groups.
  - 4.3 Were outcome assessors aware of the intervention received by study participants? → Yes
    Because the outcome is self-reported by the participants, they were fully aware of their assigned intervention.

- 4.4 Could assessment of the outcome have been influenced by knowledge of intervention received? → Yes
  Given that participants knew their group allocation, their responses on the PSS-10 could be influenced by expectations or placebo effects.
- 4.5 Is it likely that assessment of the outcome was influenced by knowledge of intervention received? → Yes
  The self-report nature and unblinded status of participants make it highly likely that their responses were affected by their awareness of the intervention.

- Risk Assessment: HIGH risk of bias
- Reasoning:
  - Although the PSS-10 is a valid and appropriate tool (4.1 = No), the measurement was conducted via self-report with participants aware of their intervention assignment (4.3 = Yes).
  - There is no difference in measurement methods between groups (4.2 = No); however, the lack of blinding increases the likelihood that the assessment was influenced by participants' knowledge of their assignment (4.4 and 4.5 = Yes).
  - According to the RoB 2.0 decision rules, when outcome assessors are aware and this awareness could have influenced the outcome assessment, the risk is judged as HIGH risk.

  Thus, for the primary outcome of stress immediately post-intervention, the risk of bias in the measurement of the outcome is HIGH risk.

Domain 5: Risk of Bias in Selection of the Reported Result

- Signaling Question Analysis:
  - 5.1 Was the analysis plan pre-specified before unblinded data were available? → No information
    The article does not provide explicit details confirming that the analysis plan was finalized prior to unblinding the outcome data.
  - 5.2 Were all eligible outcome measurements reported without selective emphasis? → No
    The study reported all planned outcome measurements (including PSS at baseline, post-intervention, and 6 months) as per the protocol, with no indication of selective reporting to favor certain results.
  - 5.3 Were all eligible analyses of the data reported, with no evidence of selective analysis? → No
    The analysis was conducted using a consistent method (linear mixed models adjusting for age and gender) without evidence that only a subset of analyses was selectively reported.
- Risk Assessment: SOME CONCERNS
- Reasoning:
  - Although there is no evidence suggesting selective reporting from multiple outcome measurements or analyses (5.2 and 5.3 are both “No”), the study does not provide sufficient detail regarding whether the analysis plan was pre-specified before unblinded outcome data became available (5.1 = No information).
  - Based on the RoB 2.0 decision rules, the absence of clear pre-specification details necessitates a cautious interpretation, resulting in a risk judgment of SOME CONCERNS for selective reporting bias.

ID: Sekhavati 2020

First Response

Domain 1: Bias Arising from the Randomization Process

- Signaling Question Analysis:

- Was the allocation sequence random? → Y

  The study explicitly states that patients were randomly divided into two treatment groups.
- Was the allocation sequence concealed? → NI

  There is no explicit description of the methods used to secure allocation concealment.
- Did baseline differences suggest a problem with randomization? → N

  Key prognostic factors (age, sex, and initial SpO_2) are balanced between groups, despite some differences in non-critical symptoms.

- Risk Assessment: Some Concerns
- Reasoning:
  - The trial reports random allocation (SQ 1.1 = Y), fulfilling the criterion for a randomized sequence.
  - However, the study does not provide details on allocation concealment (SQ 1.2 = NI).
  - Baseline characteristics for key factors influencing the primary outcome are similar (SQ 1.3 = N).
  - According to the RoB 2.0 decision rules, when allocation concealment is rated as NI and baseline differences are not problematic, the overall risk for Domain 1 defaults to Some Concerns.

Domain 2: Bias Due to Deviations from Intended Interventions

- Step 1: Analysis Population
  - Analysis Approach:

    • The study reports that all randomized patients completed the required 5-day treatment duration.

    • There is no explicit mention of "intention-to-treat" (ITT), but no post-randomization exclusions occurred, suggesting an ITT-like analysis.
- Step 2: Effect of Assignment to Intervention
  - 2.1 Were participants aware of their assigned intervention? → Y

    The trial is explicitly described as "open-label," indicating participants knew their treatment assignment.
  - 2.2 Were carers/people delivering the interventions aware? → Y

    The open-label design also implies that the personnel were aware of the assignments.
  - 2.3 Were there deviations from the intended intervention due to the trial context? → N

    There is no report of deviations arising from the open-label context; all patients adhered to the treatment protocol.
  - 2.4 Were these deviations likely to have affected the outcome? → N

    Since no deviations were reported, there is no evidence they impacted the outcome (all-cause mortality at day 28 is a hard endpoint).
  - 2.5 Were these deviations balanced between groups? → Y

    In the absence of deviations, balance is assumed.
  - 2.6 Was an appropriate analysis used to estimate the effect of assignment? → Y

    All randomized participants were analyzed according to their assigned groups, with no unjustified exclusions.
  - Risk Judgment for Effect of Assignment (Part 1 & 2):

    • Although both participants and personnel were aware of treatment assignments (SQ 2.1 & 2.2 = Y), the lack of deviations from protocol (SQ 2.3 = N) and the appropriate analysis (SQ 2.6 = Y) lead to a LOW risk judgment for the effect of assignment to intervention.
- Step 3: Effect of Adhering to Intervention

- 2.1 Were participants aware of their assigned intervention? → Y
  (Same as above: open-label design.)
- 2.2 Were carers aware of the assigned intervention? → Y
- 2.3 Were important non-protocol interventions balanced? → Y
  No non-protocol interventions are reported, suggesting balance.
- 2.4 Were there failures in implementing the intervention that could have affected the outcome? → N
  All patients completed the treatment as planned.
- 2.5 Was there non-adherence to the assigned regimen that could have affected outcomes? → N
  There is no evidence of non-adherence.
- 2.6 Was an appropriate analysis used to estimate the effect of adhering to intervention? → Y
  Given the high adherence and complete follow-up, the analysis appropriately reflects the effect of adherence.
- Risk Judgment for Effect of Adhering:
  • With high adherence and no implementation failures or imbalance in non-protocol interventions, the effect of adhering to the intervention is judged as LOW risk.

- Overall Risk Assessment for Domain 2: LOW risk
- Reasoning:
  - Although the open-label design means that both participants and caregivers were aware of the treatment assignments, there were no deviations from the intended interventions attributable to the trial context, and all patients adhered to their allocated treatments.
  - The analysis was conducted on all randomized participants without unjustified exclusions, indicating an ITT-like approach.
  - Since no deviations (or non-adherence) occurred that could bias the outcome—and given that the primary outcome (all-cause mortality at day 28) is objectively measured—the overall risk of bias due to deviations from intended interventions is assessed as LOW risk.

Domain 3: Risk of Bias Due to Missing Outcome Data

- Signaling Question Analysis:
  - 3.1 Were data for this outcome available for all, or nearly all, participants randomized? → Y
    All 111 randomized participants (56 in the case group and 55 in the control group) had complete outcome data, with mortality outcomes clearly reported (one death in the control group and none in the case group).
  - 3.2 Is there evidence that the result was not biased by missing outcome data? → Y
    There is clear evidence that missing data did not bias the result, as there were no missing outcome data reported.
  - 3.3 Could missingness in the outcome depend on its true value? → N
    Since no data are missing, missingness could not depend on the true value of the outcome.
  - 3.4 Is it likely that missingness in the outcome depended on its true value? → N
    There is no indication that missingness, if any, would be related to the true outcome given the completeness of the data.
- Risk Assessment: LOW risk of bias
- Reasoning:

- The study provides complete outcome data for nearly all participants, fulfilling the criterion for minimal missing data (SQ 3.1 = Y).
- There is clear evidence that the results are not biased by missing outcome data (SQ 3.2 = Y), and there is no concern that missingness could be related to the true outcomes (SQ 3.3 = N; SQ 3.4 = N).
- Based on these considerations and the RoB 2.0 decision rules, the overall risk of bias due to missing outcome data is assessed as LOW risk.

Domain 4: Risk of Bias in Measurement of the Outcome

- Signaling Question Analysis:
  - 4.1 Was the method of measuring the outcome inappropriate? → No
    The primary outcome, all-cause mortality, is objectively measured using standard hospital records, making the method appropriate.
  - 4.2 Could measurement or ascertainment of the outcome have differed between intervention groups? → No
    Both groups' outcomes were assessed using the same objective criteria, with no indication of differential measurement.
  - 4.3 Were outcome assessors aware of the intervention received by study participants? → Yes
    The open-label design indicates that assessors were aware of the treatment assignments.
  - 4.4 Could assessment of the outcome have been influenced by knowledge of intervention received? → No
    Despite assessors' awareness, the objective nature of the outcome (mortality) means that such knowledge is unlikely to bias the assessment.
  - 4.5 Is it likely that assessment of the outcome was influenced by knowledge of intervention received? → No
    Given the objective measurement of mortality, it is unlikely that knowledge of the intervention affected the outcome assessment.
- Risk Assessment: LOW risk
- Reasoning:
  - The measurement method for the primary outcome (all-cause mortality) is clearly appropriate and objective (SQ 4.1 = No).
  - Outcome measurement was consistent across both intervention groups (SQ 4.2 = No).
  - Although outcome assessors were aware of the intervention (SQ 4.3 = Yes), the objectivity of the outcome minimizes any potential influence of this awareness (SQ 4.4 and 4.5 = No).
  - Following the RoB 2.0 decision rules, these responses lead to an overall judgment of LOW risk for bias in the measurement of the outcome.

Domain 5: Risk of Bias in Selection of the Reported Result

- Signaling Question Analysis:
  - 5.1 Were the data that produced this result analysed in accordance with a pre-specified analysis plan finalized before unblinded outcome data were available? → No information
    The report does not provide explicit details regarding a pre-specified analysis plan or its finalization prior to unblinding.
  - 5.2 Is the numerical result being assessed likely to have been selected from multiple eligible outcome measurements within the outcome domain? → No

For the primary outcome of all-cause mortality, the report presents a single measurement without evidence of alternative outcome definitions or multiple time points that were selectively chosen.

  - 5.3 Is the numerical result being assessed likely to have been selected from multiple eligible analyses of the data? → No

    The analyses reported (e.g., standard statistical tests for mortality and other outcomes) appear to be the sole analyses performed, with no indication of selective analysis reporting.

- Risk Assessment: Some Concerns
- Reasoning:

  • Although there is no evidence of selective reporting of outcome measurements (SQ 5.2 = No) or selective analysis (SQ 5.3 = No), the trial report lacks clear information on whether the analysis was conducted in strict accordance with a pre-specified plan that was finalized before unblinded data were available (SQ 5.1 = No information).

  • According to the RoB 2.0 decision rules, in the absence of confirmed pre-specification, even when no selective reporting is evident from the outcomes or analyses, the overall risk of bias for this domain defaults to Some Concerns.

  • Therefore, based on the available evidence, Domain 5 is judged as Some Concerns.

ID: Sekhavati 2020

Second Response

Domain 1: Bias Arising from the Randomization Process

- Signaling Question Analysis:
  - Was the allocation sequence random? → Y

    The study explicitly states that patients were "randomly divided" into treatment groups.
  - Was the allocation sequence concealed? → NI

    There is no explicit description or evidence provided regarding allocation concealment.
  - Did baseline differences suggest a problem with randomization? → N

    Key baseline characteristics (e.g., age, sex, vital signs) were reported as not significantly different between groups.
- Risk Assessment: SOME CONCERNS
- Reasoning:
  - The allocation sequence is confirmed as random (Y).
  - There is no available information on allocation concealment (NI).
  - There are no substantial baseline imbalances (N).
  - According to the judgment criteria, when concealment is "No information" (NI) but baseline imbalances are absent (N), rule 4.1 applies, leading to an overall risk of Some Concerns.

Domain 2: Bias Due to Deviations from Intended Interventions

- Step 1 – Analysis Type (ITT vs. PP):
  - Observation:
    - The study reports that "all patients completed their 5-day required treatment duration" and no post-randomization exclusions were mentioned.

        - Although the text does not explicitly state "intention-to-treat," the complete follow-up of randomized participants implies an ITT approach.
    - Conclusion:
        - The analysis is consistent with an ITT approach.
- Step 2 – Effect of Assignment to Intervention
    - 2.1 Were participants aware of their assigned intervention during the trial? → Yes
      Being an open-label trial, participants were clearly aware of their intervention.
    - 2.2 Were carers and people delivering the interventions aware of participants' assigned intervention during the trial? → Yes
      Open-label design means that treating personnel were also aware of the treatment allocation.
    - 2.3 Were there deviations from the intended intervention that arose because of the trial context? → No
      The protocol was followed as all patients completed treatment without reported deviations.
    - 2.4 Were these deviations likely to have affected the outcome? → No
      No deviations were reported; therefore, there is no indication of impact on the outcome (all-cause mortality).
    - 2.5 Were these deviations from intended intervention balanced between groups? → Yes
      No deviations were observed, implying balance across groups.
    - 2.6 Was an appropriate analysis used to estimate the effect of assignment to intervention? → Yes
      All randomized participants were included in the analysis, consistent with an ITT approach.
    - 2.7 Was there potential for a substantial impact (on the result) of the failure to analyse participants in the group to which they were randomized? → No
      There were no exclusions or misclassifications that could have substantially impacted the results.
- Risk Assessment for Effect of Assignment:
    - Part 1: Although participants and personnel were aware of the assignments (Y), the absence of trial-context deviations (N) leads to a LOW risk for this component.
    - Part 2: The use of an appropriate (ITT) analysis (Y) further supports a LOW risk rating.
- Step 3 – Effect of Adhering to Intervention:
    - Since an ITT analysis was effectively implemented and there were no notable issues with adherence or implementation failures, this step does not alter the risk rating.
- Overall Risk Assessment for Domain 2: LOW risk
- Reasoning:
    - Analysis Type: All randomized participants completed the treatment, indicating an ITT-like approach with no selective exclusions.
    - Open-Label Nature: Although both participants and personnel were aware of the treatment assignment (signaling answers: Yes), the strict adherence to the protocol with no deviations minimizes potential bias from this awareness.
    - Deviations: No deviations due to the trial context were reported, and any potential deviations would have been balanced across groups.
    - Appropriate Analysis: The analysis included all randomized patients, and there is no evidence of post-randomization exclusions that could bias the outcome (all-cause mortality at day 28).
- Conclusion: With both the effect of assignment (Part 1) and the analysis method (Part 2) judged as LOW risk, the overall risk of bias due to deviations from the intended interventions is LOW risk.

Domain 3: Risk of Bias Due to Missing Outcome Data

- Signaling Question Analysis:
  - 3.1 Were data for this outcome available for all, or nearly all, participants randomized? → Yes
    Outcome data for mortality were reported for all 111 randomized participants (56 in the case group and 55 in the control group), with only one death recorded.
  - 3.2 Is there evidence that the result was not biased by missing outcome data? → Yes
    There is clear evidence that missing data did not bias the results, as nearly all participants have outcome data available.
  - 3.3 Could missingness in the outcome depend on its true value? → No
    There is no indication that missing data are related to the true outcome values, given the complete reporting.
  - 3.4 Is it likely that missingness in the outcome depended on its true value? → No
    With outcome data available for all participants, it is highly unlikely that missingness depended on the true outcome.
- Risk Assessment: LOW risk
- Reasoning:
  - Nearly all randomized participants have complete outcome data for the primary outcome (mortality), satisfying the criterion for a "Yes" in 3.1.
  - There is clear evidence that the results were not biased by missing data, as indicated in 3.2.
  - With complete data, there is no basis to suspect that any missingness (if it existed) would depend on the true outcome values (3.3 and 3.4 both being "No").
  - According to the risk of bias judgment rules, when outcome data are available for all or nearly all participants, the domain is rated as LOW risk.

Domain 4: Risk of Bias in Measurement of the Outcome

- Signaling Question Analysis:
  - 4.1 Was the method of measuring the outcome inappropriate? → N
    The outcome is all-cause mortality, which is measured using standard, objective criteria (i.e., recording deaths), and is clearly appropriate.
  - 4.2 Could measurement or ascertainment of the outcome have differed between intervention groups? → N
    Mortality is an objective outcome, and there is no indication that its measurement varied between groups.
  - 4.3 Were outcome assessors aware of the intervention received by study participants? → Y
    As the trial is open-label, outcome assessors were clearly aware of the intervention assignments.
  - 4.4 Could assessment of the outcome have been influenced by knowledge of intervention received? → N
    Despite awareness of treatment allocation, the objective nature of mortality means that the assessment is unlikely to be influenced by this knowledge.
  - 4.5 Is it likely that assessment of the outcome was influenced by knowledge of intervention received? → N
    Given the objectivity of the mortality outcome, it is highly unlikely that assessor awareness introduced bias.
- Risk Assessment: LOW risk
- Reasoning:

- Measurement Method (4.1): The method used to measure all-cause mortality is appropriate (N).
- Measurement Consistency (4.2): There is no indication that the measurement or ascertainment of mortality differed between the intervention groups (N).
- Outcome Assessor Awareness (4.3): Although the assessors were aware of the intervention (Y), the outcome is objective, which minimizes potential bias.
- Influence on Assessment (4.4 & 4.5): The assessment of mortality is unlikely to be influenced by knowledge of the intervention (N), leading to the conclusion that any potential bias is negligible.
- Final Judgment: Following the risk of bias decision rules—since the measurement method is appropriate and consistent, and any awareness of intervention does not translate into biased outcome assessment—the overall risk of bias in the measurement of the outcome is LOW risk.

Domain 5: Risk of Bias in Selection of the Reported Result

- Signaling Question Analysis:
  - 5.1 Were the data that produced this result analysed in accordance with a pre-specified analysis plan finalized before unblinded outcome data were available? → No information
    The published report does not provide explicit details regarding a pre-specified analysis plan or confirmation that the analysis plan was finalized prior to unblinding.
  - 5.2 Is the numerical result being assessed likely to have been selected, on the basis of the results, from multiple eligible outcome measurements within the outcome domain? → No
    The outcome assessed (all-cause mortality) is a standard, objective measurement, and there is no evidence that alternative measurements or definitions were available or selectively reported.
  - 5.3 Is the numerical result being assessed likely to have been selected, on the basis of the results, from multiple eligible analyses of the data? → No
    There is no indication that the investigators conducted or selectively reported multiple analyses of the outcome data.
- Risk Assessment: SOME CONCERNS
- Reasoning:
  - Although there is no evidence of selective outcome measurement (5.2 = No) or selective analysis (5.3 = No), the report does not provide sufficient details about whether the analysis plan was pre-specified (5.1 = No information).
  - According to the judgment rules, when both multiple outcome measurements and multiple analyses are not an issue (i.e., rated as No), the key factor becomes whether the trial was analysed according to a pre-specified plan.
  - Since the analysis plan's pre-specification cannot be confirmed (falling into the {N, PN, NI} category), the decision rule (1.2) dictates that the overall risk of bias in the selection of the reported result should be rated as SOME CONCERNS.

ID: Wang 2021

First Response

Domain 1: Bias Arising from the Randomization Process

- Signaling Question Analysis:

- Was the allocation sequence random? → Y

  The study explicitly states that patients were randomized using a computer-generated table.

- Was the allocation sequence concealed? → NI

  No explicit details regarding secure concealment of the allocation sequence are provided.

- Did baseline differences suggest a problem with randomization? → N

  Baseline characteristics between groups were similar, with no significant imbalances reported.

- Risk Assessment: SOME CONCERNS
- Reasoning:
  - The allocation sequence was random as evidenced by the use of a computer-generated table, which supports a "Yes" (Y) response for randomization.
  - However, there is no explicit information regarding the concealment of the allocation sequence, leading to a rating of "No Information" (NI) for concealment.
  - Since the baseline characteristics showed no substantial differences (N), rule 4.1 applies: with allocation concealment rated as NI and baseline imbalances as N, the overall risk is determined to be SOME CONCERNS.

Domain 2: Bias Due to Deviations from Intended Interventions

- Analysis Type:

  A review of the study shows that all 124 randomized patients (62 per group) were included in the analyses with no reported exclusions or post-randomization losses. Although the study does not explicitly state "intention-to-treat," the absence of exclusions indicates an ITT approach.

- Step 2: Effect of Assignment to Intervention
  - 2.1 Were participants aware of their assigned intervention? → No

    The study is described as single-blind; given that patients undergoing similar laparoscopic procedures are unlikely to know which reconstruction they received, we assume effective blinding of participants.

  - 2.2 Were carers and people delivering the interventions aware? → Yes

    Surgeons and clinical staff must know the procedure to be performed, so they were aware of the assigned intervention.

  - 2.3 Were deviations from the intended intervention due to trial context? → No

    All procedures were completed as planned with no reported deviations related to the trial setting.

  - 2.4 Were these deviations likely to have affected the outcome? → No

    Since no deviations occurred, there is no impact on the outcome.

  - 2.5 Were these deviations balanced between groups? → Yes

    No deviations were reported; thus, any potential deviations would be inherently balanced.

  - 2.6 Was an appropriate analysis used to estimate the effect of assignment? → Yes

    All randomized patients were analyzed, suggesting that an appropriate ITT-like analysis was applied.

  - 2.7 Was there potential for a substantial impact from not analyzing as randomized? → No

    There were no exclusions or misclassifications that could substantially impact the results.

- Risk Judgment – Part 1:

  Despite personnel awareness (which is inherent in surgical trials), the absence of trial-context deviations leads to a LOW risk rating for the effect of assignment to intervention.

- Step 3: Effect of Adhering to Intervention

- 2.1 Were participants aware of their assigned intervention? → No
  Participants were likely blinded to the specific intervention details.
- 2.2 Were carers aware? → Yes
  As noted, surgical teams were aware due to the nature of the intervention.
- 2.3 Were important non-protocol interventions balanced across groups? → Yes
  No additional non-protocol interventions were reported, implying balance.
- 2.4 Were there failures in implementing the intervention that could have affected the outcome? → No
  All procedures were executed as intended with complete adherence.
- 2.5 Was there non-adherence to the assigned intervention that could have affected outcomes? → No
  There is no report of deviations or non-adherence.
- 2.6 Was an appropriate analysis used to estimate the effect of adhering to the intervention? → Yes
  The analysis appears to appropriately reflect the intervention as delivered.

- Risk Judgment – Part 2:
  With no implementation failures or non-adherence issues, and given that appropriate analytic methods were used, the effect of adhering to intervention is judged as LOW risk.
- Overall Risk Assessment for Domain 2:
  Since both the effect of assignment (Part 1) and the effect of adherence (Part 2) are LOW risk, the overall risk of bias due to deviations from intended interventions is LOW risk.
- Reasoning:
  The study appears to have included all randomized patients in an ITT-like analysis without any post-randomization exclusions. Although intervention providers were aware of the treatment assignment (a common and unavoidable feature in surgical trials), no deviations from the intended interventions were reported that could influence the primary outcome. Additionally, any potential non-protocol interventions or adherence issues were not evident. Thus, following the RoB 2.0 decision rules, the overall risk for Domain 2 is LOW risk.

Domain 3: Risk of Bias Due to Missing Outcome Data

- Signaling Question Analysis:
  - 3.1 Were data for this outcome available for all, or nearly all, participants randomized? → Yes
    The report provides postoperative complication data for all 124 randomized participants, with no indication of missing outcome data.
  - 3.2 Is there evidence that the result was not biased by missing outcome data? → Yes
    Clear outcome data are presented, and there is no evidence suggesting bias from missing data.
  - 3.3 Could missingness in the outcome depend on its true value? → No
    With complete data reported, there is no concern that missingness (if any) was related to the true outcome values.
  - 3.4 Is it likely that missingness in the outcome depended on its true value? → No
    The absence of missing data precludes any dependency on the true outcome values.
- Risk Assessment: Low risk
- Reasoning:
  The study provides outcome data for all or nearly all participants (i.e., 100% follow-up for major postoperative complications). With complete data and clear evidence that missing data did not bias the results, there is no concern

regarding missing outcome data. According to the RoB 2.0 decision rules, this domain is therefore judged as Low risk.

Domain 4: Risk of Bias in Measurement of the Outcome

- Signaling Question Analysis:
  - 4.1 Was the method of measuring the outcome inappropriate? → No
    Major postoperative complications were measured using standard, objective criteria (e.g., Clavien-Dindo classification), which is appropriate for this outcome.
  - 4.2 Could measurement or ascertainment of the outcome have differed between intervention groups? → No
    The measurement method was applied consistently across both groups, with no indication of differential ascertainment.
  - 4.3 Were outcome assessors aware of the intervention received by study participants? → Probably yes
    Given the single-blind design, it is likely that outcome assessors (or the clinical team documenting complications) were aware of the intervention, though this is common in surgical trials.
  - 4.4 Could assessment of the outcome have been influenced by knowledge of intervention received? → No
    Despite the likely awareness of intervention assignment, the objective nature of the outcome (major postoperative complications) minimizes the potential for influence on measurement.
  - 4.5 Is it likely that assessment of the outcome was influenced by knowledge of intervention received? → No
    There is no evidence that the assessment was affected by the assessors' knowledge, as the outcome relies on clear clinical criteria.
- Risk Assessment: LOW risk
- Reasoning:
  The study employed appropriate and objective methods for measuring major postoperative complications, and these methods were applied uniformly across both intervention groups. Although outcome assessors were likely aware of the assigned intervention due to the single-blind design, the objectivity of the measurement criteria substantially reduces the risk that this awareness could bias the outcome assessment. Consequently, the overall risk of bias in the measurement of the outcome is judged as LOW risk.

Domain 5: Risk of Bias in Selection of the Reported Result

- Signaling Question Analysis:
  - 5.1 Were the data that produced this result analysed in accordance with a pre-specified analysis plan that was finalized before unblinded outcome data were available for analysis? → Probably yes
    The study was registered (ChiCTR-INR-17010594) and the protocol was published prior to data analysis. Although explicit confirmation of finalization timing is not detailed, the available trial registration and protocol information suggest that a pre-specified analysis plan was in place.
  - 5.2 Is the numerical result being assessed likely to have been selected, on the basis of the results, from multiple eligible outcome measurements within the outcome domain? → No
    For the primary outcome of major postoperative complications, the study clearly reported all relevant data (see Table 3), with no evidence of selective reporting across multiple outcome measurements.
  - 5.3 Is the numerical result being assessed likely to have been selected, on the basis of the results, from multiple eligible analyses of the data? → No

The statistical analysis was described comprehensively, and there is no indication that only a favorable subset of analyses was reported.

- Risk Assessment: LOW risk
- Reasoning:
  Both potential sources of selective reporting—selective measurement of outcomes (5.2) and selective reporting of analyses (5.3)—were not evident (both rated as "No"). Additionally, the trial appears to have been analyzed in accordance with a pre-specified plan (rated "Probably yes" in 5.1). According to the decision rules, with both multiple outcome measurements and multiple analyses rated as "No" (or PN) and a pre-specified analysis plan in place (Y/PY), the overall risk of bias in the selection of the reported result is judged as LOW risk.

ID: Wang 2021

Second Response

Domain 1: Bias Arising from the Randomization Process

- Signaling Question Analysis:
  - 1.1 Was the allocation sequence random? → Y
    Evidence: The study explicitly states that eligible patients were randomized using a computer-generated table.
  - 1.2 Was the allocation sequence concealed? → NI
    Evidence: There is no explicit information provided regarding the concealment of the allocation sequence.
  - 1.3 Did baseline differences suggest a problem with randomization? → N
    Evidence: The baseline characteristics show no significant differences between the groups.
- Risk Assessment: Some Concerns
- Reasoning:
  - The allocation sequence was clearly random (Y).
  - However, there is no explicit detail on allocation concealment, so it is rated as No Information (NI).
  - Baseline characteristics were well balanced (N).
  - According to rule 4.1, if the allocation sequence concealment is NI and baseline imbalances are in {N, PN, NI}, the risk of bias is judged as Some Concerns.
  - No logical inconsistencies were detected, so the final risk judgment remains Some Concerns.

Domain 2: Bias Due to Deviations from Intended Interventions

- Step 1: Analysis Population Evaluation
  - Analysis Type:
    - The study reports that all 124 randomized patients underwent the assigned procedure without any intraoperative conversion or dropout. Although the text does not explicitly state "intention-to-treat (ITT)," the absence of post-randomization exclusions effectively indicates an ITT analysis.
- Step 2: Effect of Assignment to Intervention
  - 2.1 Were participants aware of their assigned intervention? → No information
    Rationale: The paper does not provide explicit details on participant blinding regarding their reconstruction type.

- 2.2 Were carers and people delivering the interventions aware of participants' assigned intervention? → No information
  Rationale: There is no clear description of blinding for the surgeons or other personnel.
- 2.3 Were there deviations from the intended intervention that arose because of the trial context? → No
  Rationale: The study states that all procedures were successfully completed as planned, with no reported deviations (e.g., no conversions or crossovers).
- 2.4 Were these deviations likely to have affected the outcome? → No
  Rationale: Since no deviations occurred, there is no impact on the primary outcome.
- 2.5 Were these deviations from intended intervention balanced between groups? → Yes
  Rationale: In the absence of deviations, balance is maintained by default.
- 2.6 Was an appropriate analysis used to estimate the effect of assignment to intervention? → Yes
  Rationale: All randomized patients were analyzed and no unjustified exclusions occurred, effectively representing an ITT approach.
- 2.7 Was there potential for a substantial impact of failure to analyze participants in the group to which they were randomized? → No
  Rationale: With all randomized participants included and no exclusions, there is no substantial impact from analysis failures.

- Risk of Bias Judgment for Domain 2
  - Part 1 (Effect of Assignment to Intervention):
    Although participant and personnel awareness are rated as "No information" (which falls into the {Y, PY, NI} branch), the absence of deviations from the intended intervention (2.3 = No) leads to a judgment of LOW risk for Part 1.
  - Part 2 (Appropriate Analysis):
    An appropriate analysis was used (2.6 = Yes) and there is no evidence of substantial impact from exclusions (2.7 = No), leading to LOW risk for Part 2.
  - Overall Judgment:
    Since Part 1 = LOW risk and Part 2 = LOW risk, the overall risk of bias for Domain 2 is LOW risk.
- Summary for Domain 2: Bias Due to Deviations from Intended Interventions
  - Analysis Population: Effectively ITT (all randomized patients were included).
  - Participant/Personnel Awareness: No explicit information provided.
  - Deviations from Intended Intervention: None occurred; no impact on outcome.
  - Analysis Method: Appropriate analysis was used.
  - Final Risk Assessment: LOW risk
- Reasoning: The lack of deviations from the intended interventions, combined with the inclusion of all randomized patients in the analysis and no indications of differential adherence or deviations affecting the outcome, supports a LOW risk judgment for this domain.

Domain 3: Risk of Bias Due to Missing Outcome Data

- Signaling Question Analysis:

- 3.1 Were data for this outcome available for all, or nearly all, participants randomized? → Y
  Rationale: The report includes outcome data for all 124 randomized participants, with no indications of missing data.
- 3.2 Is there evidence that the result was not biased by missing outcome data? → Y
  Rationale: With outcome data available for all participants, there is clear evidence that missing data did not bias the results.
- 3.3 Could missingness in the outcome depend on its true value? → N
  Rationale: Since virtually no data are missing, there is no concern that missingness depended on the true outcome.
- 3.4 Is it likely that missingness in the outcome depended on its true value? → N
  Rationale: There is no indication of missing outcome data; hence, missingness is unlikely to be related to the true outcomes.

- Risk Assessment: LOW risk of bias
- Reasoning:
  - With outcome data available for all participants (3.1 = Y) and clear evidence that the results are not biased by missing data (3.2 = Y), the potential for bias due to missing outcome data is minimal.
  - Additionally, there is no indication that missingness is related to the true outcome values (3.3 = N and 3.4 = N).
  - According to the decision rules, when outcome data are complete (or nearly complete), the risk of bias is judged as LOW risk.

Domain 4: Risk of Bias in Measurement of the Outcome

- Signaling Question Analysis:
  - 4.1 Was the method of measuring the outcome inappropriate? → No
    Rationale: Major postoperative complications were measured using established, objective criteria (e.g., Clavien-Dindo classification), which is an appropriate and validated method.
  - 4.2 Could measurement or ascertainment of the outcome have differed between intervention groups? → No
    Rationale: The assessment of postoperative complications was conducted in a consistent manner across both groups, with no indication that the measurement method differed.
  - 4.3 Were outcome assessors aware of the intervention received by study participants? → Probably yes
    Rationale: As this is a single-blind surgical trial, it is likely that the outcome assessors (e.g., the surgical team or hospital staff reviewing complications) were aware of the intervention; however, explicit details on assessor blinding are not provided.
  - 4.4 Could assessment of the outcome have been influenced by knowledge of intervention received? → No
    Rationale: Despite the likelihood that assessors were aware of the intervention, the outcome (major postoperative complications) is objectively defined using standard criteria, minimizing the risk that knowledge of intervention affected assessment.
  - 4.5 Is it likely that assessment of the outcome was influenced by knowledge of intervention received? → No
    Rationale: Given the objective nature of the complication measures and the use of established criteria, it is unlikely that assessor awareness led to biased outcome assessment.
- Risk Assessment: LOW risk of bias
- Reasoning:

- The measurement method was appropriate (4.1 = No).
- There is consistency in outcome measurement across intervention groups (4.2 = No).
- Although outcome assessors were likely aware of the intervention (4.3 = Probably yes), the objectivity of the outcome measurement minimizes potential influence.
- Both the potential for and the likelihood of bias due to assessor awareness are low (4.4 and 4.5 = No).
- Following the decision rules, since the measurement methods are appropriate and any assessor awareness does not influence the objective outcome, the overall risk of bias in measurement is judged as LOW risk.

Domain 5: Risk of Bias in Selection of the Reported Result

- Signaling Question Analysis:
  - 5.1 Were the data that produced this result analysed in accordance with a pre-specified analysis plan? → Probably yes
    Rationale: The trial was registered (ChiCTR-INR-17010594) and the protocol was published prior to data analysis, indicating that a pre-specified plan existed even though explicit confirmation regarding finalization before unblinding is not detailed.
  - 5.2 Is the numerical result being assessed likely to have been selected from multiple eligible outcome measurements? → No
    Rationale: The outcomes, including major postoperative complications, were reported as defined in the protocol without evidence of selective aggregation or unusual prioritization.
  - 5.3 Is the numerical result being assessed likely to have been selected from multiple eligible analyses of the data? → No
    Rationale: The analyses performed (e.g., standard statistical tests) were consistent with the pre-specified plan, and there is no indication of selective reporting among multiple analyses.
- Risk Assessment: LOW risk of bias
- Reasoning:
  - Since there is evidence from trial registration and a published protocol that a pre-specified analysis plan existed (5.1 = Probably yes), and there is no evidence of selective reporting from multiple outcome measurements (5.2 = No) or multiple analyses (5.3 = No), the decision rule directs us to a LOW risk of bias.
  - No discrepancies or selective reporting issues (e.g., unusual aggregation, outcome prioritization, or selective analysis) were detected when comparing the protocol and the reported results.
- Final Judgment: LOW risk of bias in selection of the reported result.

ID: Van Heeringen 1996

First Response

Domain 1: Bias Arising from the Randomization Process

- Signaling Question Analysis:
  - Was the allocation sequence random? → Y
    The study explicitly states that patients were "randomised" in a double-blind, placebo-controlled design.
  - Was the allocation sequence concealed? → PY
    The use of indistinguishable capsules in a double-blind setting provides strong contextual evidence for proper concealment, even though explicit details of the concealment method are not provided.

- Did baseline differences suggest a problem with randomization? → N
  Baseline characteristics (age, body weight, height, and HRSD scores) were well matched between groups.

- Risk Assessment: Low
- Reasoning:
  - The allocation sequence was explicitly reported as random (SQ 1.1: Y).
  - The design details (double-blind, indistinguishable capsules) provide strong contextual clues that allocation concealment was likely implemented (SQ 1.2: PY).
  - Baseline characteristics showed no imbalances (SQ 1.3: N).
  - According to the predefined decision rule: if the allocation sequence is concealed in {Y, PY}, the allocation is random in {Y, PY, NI}, and baseline imbalances are in {N, PN, NI}, then the risk is classified as Low.
  - No logical inconsistencies were detected; hence, no corrections are necessary.

Domain 2: Bias Due to Deviations from Intended Interventions

- Step 1: Analysis Approach
  - Analysis Type: The study states that "efficacy analyses were performed on an intention-to-treat basis," including all patients who received at least one dose and had at least one post-baseline assessment. This qualifies as an ITT (or modified ITT where exclusions are only due to missing outcome data) analysis.
- Step 2: Effect of Assignment to Intervention
  - 2.1 Were participants aware of their assigned intervention?
    → No
    The trial was explicitly described as double-blind with indistinguishable capsules, ensuring that participants were effectively blinded.
  - 2.2 Were carers and people delivering the interventions aware of participants' assigned intervention?
    → No
    The double-blind design implies that both carers and intervention providers were blinded to the treatment assignment.
  - 2.3 Were there deviations from the intended intervention that arose because of the trial context?
    → No
    The study does not report any trial-specific deviations (e.g., co-interventions or differential treatment) that would have altered the delivery of the intended interventions.
  - 2.4 Were these deviations likely to have affected the outcome?
    → No
    There is no evidence that any deviation (including the observed premature terminations) systematically impacted the outcome beyond what is addressed by the ITT analysis and the LOCF approach.
  - 2.5 Were these deviations from intended intervention balanced between groups?
    → Yes
    Although there was a difference in premature terminations (15 in placebo vs. 6 in mianserin), these dropouts were handled within the ITT framework (with LOCF) and do not reflect a differential deviation in intervention delivery.
  - 2.6 Was an appropriate analysis used to estimate the effect of assignment to intervention?
    → Yes

An intention-to-treat analysis was performed, including all patients with at least one post-baseline efficacy measure, which is the accepted approach to mitigate bias from post-randomization exclusions.

  - 2.7 Was there potential for a substantial impact of the failure to analyze participants in the group to which they were randomized?
    → No
    While there is an imbalance in dropout numbers, the use of ITT with LOCF and the inclusion of all available efficacy data suggest that any potential impact on the results was minimized.
- Risk Assessment: Low Risk
- Reasoning:
  - The study clearly employed an ITT (or modified ITT) approach, including all patients with post-baseline efficacy data, which minimizes bias from exclusions.
  - The double-blind design ensured that both participants and personnel were unaware of treatment assignments (SQ2.1 and SQ2.2: No).
  - There is no evidence of trial-context deviations (SQ2.3: No) or deviations that could have impacted the outcome (SQ2.4: No), and any such deviations were managed in a balanced manner across groups (SQ2.5: Yes).
  - The analysis method (ITT with LOCF) is appropriate to estimate the effect of assignment (SQ2.6: Yes) and does not suggest a substantial impact from any exclusions (SQ2.7: No).

- Given that both the assignment effect (Part 1) and the analytical approach (Part 2) are robust and no concerns emerge from deviations, the overall risk of bias for Domain 2 is judged as Low Risk.

Domain 3: Bias Due to Missing Outcome Data

- Signaling Question Analysis:
  - 3.1 Were data for this outcome available for all, or nearly all, participants randomized? → No
    Although the study performed an ITT analysis (including all patients with at least one post-baseline measurement), a substantial proportion of participants did not complete the full follow-up (notably, 15 of 27 placebo-treated patients and 6 of 28 mianserin-treated patients terminated early). This level of dropout means that complete outcome data were not available for nearly all participants (i.e., well below the ≥95% threshold for continuous outcomes).
  - 3.2 Is there evidence that the result was not biased by missing outcome data? → No
    The study used LOCF to impute missing data, but given the high and imbalanced dropout rates—especially with more dropouts in the placebo group due to lack of efficacy—there is no clear evidence that the missing data did not bias the effect estimate.
  - 3.3 Could missingness in the outcome depend on its true value? → Yes
    The fact that significantly more patients in the placebo group dropped out due to lack of efficacy strongly suggests that the missing data are related to the true (likely poorer) outcomes.
  - 3.4 Is it likely that missingness in the outcome depended on its true value? → Yes
    Given the dropout pattern (with poorer outcomes likely leading to discontinuation), it is highly likely that missingness depended on the true outcome values.
- Risk Assessment: HIGH risk of bias
- Reasoning:

- Since not nearly all participants provided complete outcome data (SQ3.1: No), we must consider the potential for bias from missing data.
- There is no convincing evidence to dismiss concerns about bias from missing data (SQ3.2: No).
- The dropout pattern indicates that missingness is related to the participants' outcomes (SQ3.3: Yes), and it is very likely that those with poorer responses (especially in the placebo group) were more prone to dropout (SQ3.4: Yes).
- According to the predefined decision rules, when missingness is likely to depend on the true outcome values in this context, the overall risk of bias due to missing outcome data should be judged as HIGH risk.

Domain 4: Risk of Bias in Measurement of the Outcome

- Signaling Question Analysis:
  - 4.1 Was the method of measuring the outcome inappropriate? → No
    The study measured depressive symptoms using the Hamilton Rating Scale for Depression (HRSD), a well-validated and appropriate instrument for this population.
  - 4.2 Could measurement or ascertainment of the outcome have differed between intervention groups? → No
    Both treatment arms were assessed with the same standardized HRSD instrument, ensuring consistency across groups.
  - 4.3 Were outcome assessors aware of the intervention received by study participants? → No
    The trial was conducted in a double-blind manner, meaning outcome assessors were blinded to treatment allocation.
  - 4.4 Could assessment of the outcome have been influenced by knowledge of intervention received? → No
    With blinded assessors and the use of an objective, validated tool, there is no indication that assessment was influenced by intervention knowledge.
  - 4.5 Is it likely that assessment of the outcome was influenced by knowledge of intervention received? → No
    The blinding procedures and uniform measurement protocol minimize the risk of such influence.
- Risk Assessment: Low Risk
- Reasoning:
  - The HRSD is a validated and appropriate measurement tool, applied uniformly across both intervention groups (SQ 4.1 and 4.2: No).
  - Outcome assessors were blinded to treatment assignment, eliminating potential influence on the measurement (SQ 4.3: No).
  - There is no evidence or suggestion that the assessment of outcomes was influenced by knowledge of the intervention (SQ 4.4 and 4.5: No).
  - Based on these evaluations and following the predefined RoB 2.0 decision rules, the risk of bias in the measurement of the outcome is judged as Low Risk.

Domain 5: Risk of Bias in Selection of the Reported Result

- Signaling Question Analysis:
  - 5.1 Were the data analyzed in accordance with a pre-specified analysis plan finalized before unblinded data were available? → No information

The report does not provide details about a pre-specified analysis plan or its finalization relative to unblinded data.

  - 5.2 Is the numerical result likely to have been selected from multiple eligible outcome measurements? → No

    The primary outcome (change in HRSD score) is clearly defined, and the study reports results consistently across the specified time points without evidence of selective measurement.
  - 5.3 Is the numerical result likely to have been selected from multiple eligible analyses of the data? → No

    There is no indication that alternative analyses were selectively reported; the analysis appears to follow the described ITT approach with LOCF.
- Risk Assessment: Some Concerns
- Reasoning:
  - Although the study clearly reports on the primary outcome and does not show signs of selective measurement or analysis (5.2 and 5.3: No), the absence of explicit details regarding a pre-specified analysis plan (5.1: No information) prevents full confidence in the reporting process.
  - According to the decision rules, when both multiple outcome measurements and analyses are rated as No (or PN) but the pre-specified analysis plan information is missing (NI), the overall judgment should be Some Concerns.

ID: Van Heeringen 1996

Second Response

Domain 1: Bias Arising from the Randomization Process

- Signaling Question Analysis:
  - Was the allocation sequence random? → Y

    The study is explicitly described as "randomised" with patients allocated to mianserin or placebo.
  - Was the allocation sequence concealed? → PY

    While the report does not detail the specific method of concealment, the use of indistinguishable capsules and the double-blind design strongly suggest proper concealment.
  - Did baseline differences suggest a problem with randomization? → N

    Baseline characteristics (e.g., HRSD scores, age, weight, height) were well balanced between the groups.
- Risk Assessment: Low Risk
- Reasoning:
  - The allocation sequence is confirmed to be random (Y) and the design implies proper concealment (PY).
  - With baseline characteristics balanced (N), the criteria in rule 3 are met:

    "If allocation sequence concealed in {Y, PY} AND allocation sequence random in {Y, PY, NI} AND baseline imbalances in {N, PN, NI}, return LOW risk."
  - No inconsistencies were detected in applying these rules, so no error correction was necessary.

Domain 2: Bias Due to Deviations from Intended Interventions

- Signaling Question Analysis:

  Step 1 – Analysis Type:

- The study clearly states that "efficacy analyses were performed on an intention-to-treat basis, thus including the patients who received at least one dose of study medication and had at least one post-baseline efficacy assessment."
  → Analysis Type: ITT
- Step 2 – Effect of Assignment to Intervention:
  - 2.1 Were participants aware of their assigned intervention during the trial?
    → No
    The trial is described as double-blind, implying effective blinding of participants.
  - 2.2 Were carers and people delivering the interventions aware of participants' assigned intervention during the trial?
    → No
    The double-blind design indicates that personnel were also blinded.
  - 2.3 Were there deviations from the intended intervention that arose because of the trial context?
    → No
    There is no evidence that deviations were introduced by trial-specific factors; premature terminations appear related to lack of efficacy rather than trial conduct.
  - 2.4 Were these deviations likely to have affected the outcome?
    → No
    Despite differential dropout (15 placebo vs. 6 mianserin), the ITT analysis using LOCF minimizes the impact on the outcome.
  - 2.5 Were these deviations from intended intervention balanced between groups?
    → No
    There is an imbalance in premature terminations; however, given the effective blinding, this likely reflects differences in treatment efficacy rather than bias from deviations.
  - 2.6 Was an appropriate analysis used to estimate the effect of assignment to intervention?
    → Yes
    The study employed an ITT approach with LOCF, which is considered appropriate when exclusions are only due to missing data.
  - 2.7 Was there potential for a substantial impact of the failure to analyze participants in the group to which they were randomized?
    → No
    The ITT analysis appears to have adequately addressed the potential impact of drop-outs.
- Risk Assessment:
  Part 1 – Effect of Assignment:
  - Since both participants (2.1: No) and personnel (2.2: No) were effectively blinded, Part 1 is rated Low risk.
- Part 2 – Analysis:
  - With an appropriate ITT analysis used (2.6: Yes) and no substantial impact from deviations (2.7: No), Part 2 is rated Low risk.
- Overall Domain 2 Risk:
  - As both Part 1 and Part 2 are Low risk, the final judgment for Domain 2 is Low Risk.
- Reasoning:

- The study employs an intention-to-treat analysis with LOCF, which is appropriate for handling missing outcome data.
- The double-blind design indicates that neither participants nor carers were aware of treatment assignment, reducing the likelihood that deviations from the intended intervention were driven by knowledge of allocation.
- Although there is an imbalance in premature terminations between groups, these deviations appear to stem from differential responses (lack of efficacy) rather than deviations induced by trial conduct.
- Combining these factors, the decision tree rules lead to an overall judgment of Low Risk for Domain 2.

Domain 3: Risk of Bias Due to Missing Outcome Data

- Signaling Question Analysis:
  - 3.1 Were data for this outcome available for all, or nearly all, participants randomized? → N
    Although all 55 randomized patients were included in the ITT analysis, a substantial number (notably 15 in the placebo group and 6 in the mianserin group) prematurely terminated the study. With a high dropout rate and use of LOCF (which treats imputed values as missing), the proportion of fully observed outcome data falls below the ≥95% threshold for continuous outcomes.
  - 3.2 Is there evidence that the result was not biased by missing outcome data? → N
    There is no clear evidence (e.g., sensitivity analyses) provided that missing outcome data did not bias the results. The reliance on LOCF without additional bias-correction measures leaves concern that the missing data could have affected the outcome estimate.
  - 3.3 Could missingness in the outcome depend on its true value? → Y
    The reasons for premature termination—especially the significantly higher dropout rate due to lack of efficacy in the placebo group—strongly suggest that missingness is related to the true (likely poorer) outcomes.
  - 3.4 Is it likely that missingness in the outcome depended on its true value? → Y
    Given that dropouts in the placebo group were largely due to lack of efficacy, it is likely that missing outcome data are associated with higher (worse) HRSD scores, thus influencing the effect estimate.
- Risk Assessment:
  Based on the decision rules:
  - Since 3.1 is N and 3.2 is N, we proceed to the missingness dependency check.
  - With 3.3 = Y and 3.4 = Y, rule 2.2.2.2 applies: "If likely that missingness depended on true value in {Y, PY, NI}, return HIGH risk of bias."
    Final Judgment: High Risk of Bias
- Reasoning:
  - Outcome Data Availability: A significant proportion of participants had missing outcome data (as evidenced by high premature termination rates and reliance on LOCF imputation), failing the ≥95% threshold.
  - Evidence on Bias: There is insufficient evidence to dismiss concerns of bias introduced by the missing data.
  - Dependency on True Outcome: The dropout pattern (notably more in the placebo group due to lack of efficacy) indicates that missingness is likely related to worse outcomes, thereby biasing the effect estimate.
  - Conclusion: The combination of substantial missing data, inadequate evidence to allay bias concerns, and the strong likelihood that missingness depended on true outcome values leads to an overall judgment of High Risk of Bias for this domain.
- No inconsistencies were detected during the application of the decision rules.

Domain 4: Risk of Bias in Measurement of the Outcome

- Signaling Question Analysis:
  - 4.1 Was the method of measuring the outcome inappropriate? → No
    The study used the 21-item Hamilton Rating Scale for Depression (HRSD), a well-established and validated instrument for assessing depressive symptoms.
  - 4.2 Could measurement or ascertainment of the outcome have differed between intervention groups? → No
    The HRSD was applied uniformly across groups, and there is no indication that its administration varied between the mianserin and placebo arms.
  - 4.3 Were outcome assessors aware of the intervention received by study participants? → No
    The trial is described as double-blind, which implies that outcome assessors were blinded to group allocation.
  - 4.4 Could assessment of the outcome have been influenced by knowledge of intervention received? → No
    With blinded assessors and standardized measurement methods, there is no indication that outcome assessment was influenced by knowledge of the intervention.
  - 4.5 Is it likely that assessment of the outcome was influenced by knowledge of intervention received? → No
    Given the effective blinding and objective measurement using the HRSD, it is unlikely that outcome assessment was biased.
- Risk Assessment: Low Risk
- Reasoning:
  - The measurement method (HRSD) is a validated and appropriate tool for assessing depressive symptoms.
  - Outcome measurement was applied consistently across intervention groups.
  - The double-blind design ensured that both participants and outcome assessors remained unaware of treatment allocation, minimizing the potential for bias.
  - The decision tree clearly supports a Low Risk judgment when the measurement method is appropriate and consistent, and assessors are blinded with no indication of influence on outcome assessment.

Domain 5: Risk of Bias in Selection of the Reported Result

- Signaling Question Analysis:
  - 5.1 Were the data that produced this result analysed in accordance with a pre-specified analysis plan finalized before unblinded outcome data were available? → No Information
    The article provides details on the statistical methods (e.g., ITT analysis with LOCF, t-tests, Fisher's exact tests) but does not specify whether a pre-specified analysis plan was finalized before unblinding.
  - 5.2 Is the numerical result being assessed likely to have been selected from multiple eligible outcome measurements? → No
    The study reports its primary outcome (change in 21-item HRSD scores and responder status) alongside factor analyses. All eligible outcomes appear to have been reported without evidence of selective measurement or aggregation designed to emphasize particular results.
  - 5.3 Is the numerical result being assessed likely to have been selected from multiple eligible analyses of the data? → No
    The analyses appear to follow standard methods for clinical trials, with no indication that only a subset of pre-specified analyses was chosen based on the results.

- Risk Assessment:
  - Since both multiple outcome measurements and multiple analyses of data are answered as No (i.e., not suggestive of selective reporting) and the pre-specification of the analysis plan is No Information, rule 1.2 applies:

    "If trial analyzed in accordance with a pre-specified plan in {N, PN, NI}, return SOME CONCERNS."

    Final Judgment: Some Concerns
- Reasoning:
  - Although the study thoroughly reports its outcomes and analyses, the lack of explicit detail regarding the finalization of a pre-specified analysis plan before unblinding introduces uncertainty.
  - In the absence of evidence confirming adherence to a pre-specified plan, even though no selective reporting of outcomes or analyses is apparent, a cautious approach leads to an overall judgment of Some Concerns for Domain 5.

ID: Bart 2005

First Response

Domain 1: Bias Arising from the Randomization Process

- Signaling Question Analysis:
  - Was the allocation sequence random? → Yes

    The study explicitly states that patients were "randomized," providing evidence that a random allocation process was used.
  - Was the allocation sequence concealed? → No Information

    The report does not provide details on the methods used for allocation concealment.
  - Did baseline differences suggest a problem with randomization? → No

    Baseline characteristics between the groups are largely similar, with no substantial imbalances observed.
- Risk Assessment: Some Concerns
- Reasoning:
  - The trial is described as a randomized controlled trial, which supports a "Yes" for a random allocation sequence.
  - However, there is no information provided regarding the concealment of the allocation sequence, so this is marked as "No Information."
  - Baseline differences do not suggest any problem with randomization, as the groups are comparable.
  - According to the decision rules, when the allocation concealment is "No Information" and baseline imbalances are not present (i.e., marked as "No"), the overall risk for this domain is rated as Some Concerns.

Domain 2: Bias Due to Deviations from Intended Interventions

- Step 1: Analysis Type
  - The study reports that "all group comparisons were performed on an intention-to-treat basis."

    → Analysis classification: ITT
- Since an ITT analysis was used, we proceed with the effect of assignment to intervention.

- Step 2: Effect of Assignment to Intervention
  - 2.1 Were participants aware of their assigned intervention? → Yes
    The UF procedure involves a distinct intervention (catheter placement, fluid removal) that makes it apparent to participants which group they are in.
  - 2.2 Were carers and people delivering the interventions aware of participants' assigned intervention? → Yes
    Given the procedural nature (UF versus usual care), intervention providers were clearly aware of the assignments.
  - 2.3 Were there deviations from the intended intervention that arose because of the trial context? → Probably yes
    In the UF group, deviations occurred (e.g., failure to obtain IV access in one patient and inability to withdraw blood in another) that appear to be related to trial-specific procedural factors.
  - 2.4 Were these deviations likely to have affected the outcome? → No
    While the UF group experienced these procedural deviations, there is no clear evidence that these affected the primary outcome (considering all-cause mortality as the outcome of interest, and with only one reported death judged as unrelated to UF).
  - 2.5 Were these deviations from intended intervention balanced between groups? → No
    Such deviations occurred only in the UF group, indicating an imbalance in adherence to the intended intervention.
  - 2.6 Was an appropriate analysis used to estimate the effect of assignment to intervention? → Yes
    The study employed an ITT approach, thereby including all randomized participants regardless of these deviations.
  - 2.7 Was there potential for a substantial impact of the failure to analyze participants in the group to which they were randomized? → No
    No major exclusions or misclassifications occurred given the ITT analysis.
- Risk-of-Bias Judgment for Effect of Assignment to Intervention:
  - Part 1 (Deviation-related issues):
    Since participants and personnel were aware (2.1 & 2.2 = Yes) and deviations arose due to trial context (2.3 = Probably yes) but these deviations were judged unlikely to affect the outcome (2.4 = No), the deviation check leads to a judgment of Some Concerns (especially given that the deviations were imbalanced between groups, 2.5 = No).
  - Part 2 (Analysis of assignment):
    An appropriate ITT analysis was used (2.6 = Yes) and there is no substantial impact from failure to analyze as randomized (2.7 = No), which results in a Low risk rating for this part.
  - Overall for Domain 2:
    Since Part 1 is Some Concerns and Part 2 is Low risk, and neither part reaches High risk, the final risk judgment for this domain is Some Concerns.
- Final Structured Output for Domain 2:
  - Signaling Question Analysis:
    - 2.1 Were participants aware of their assigned intervention? → Yes
    - 2.2 Were carers and people delivering the interventions aware? → Yes
    - 2.3 Were there deviations from the intended intervention due to trial context? → Probably yes
    - 2.4 Were these deviations likely to have affected the outcome? → No

    - 2.5 Were these deviations balanced between groups? → No

    - 2.6 Was an appropriate analysis used (ITT)? → Yes
    - 2.7 Was there potential for substantial impact due to failure to analyze as randomized? → No
- Risk Assessment: Some Concerns
- Reasoning:
  The study used an ITT analysis, which is appropriate (2.6 = Yes). However, both participants and intervention providers were aware of treatment allocation (2.1 and 2.2 = Yes), and there were deviations in the UF group due to procedural challenges (2.3 = Probably yes) that were not balanced between groups (2.5 = No). Although these deviations were judged unlikely to affect the outcome (2.4 = No) and the impact of misclassification is minimal (2.7 = No), the imbalance in deviations contributes to a judgment of Some Concerns for this domain.

Domain 3: Risk of Bias Due to Missing Outcome Data

- Signaling Question Analysis:
  - 3.1 Were data for this outcome available for all, or nearly all, participants randomized? → Yes
    The report indicates that outcome data (all-cause mortality) were available for nearly all participants (only one death was reported among 40 participants, with no suggestion of missing mortality data).
  - 3.2 Is there evidence that the result was not biased by missing outcome data? → Yes
    The near-complete outcome data suggest that missing data did not bias the results.
  - 3.3 Could missingness in the outcome depend on its true value? → No
    Given the objective nature of mortality data and its nearly complete ascertainment, missingness is unlikely to be related to the true outcome.
  - 3.4 Is it likely that missingness in the outcome depended on its true value? → No
    There is no indication that the few missing data (if any) would be dependent on the true mortality outcome.
- Risk Assessment: LOW risk of bias
- Reasoning:
  - Completeness of Outcome Data (3.1): The mortality outcome data were available for all, or nearly all, participants, which meets the ≥95% criterion for continuous outcomes (adapted for this binary outcome).
  - Evidence Against Bias (3.2): The objective nature of mortality minimizes the risk of bias from missing data, and there is clear evidence that missing data did not bias the results.
  - Dependency on True Outcome (3.3 & 3.4): There is no reason to suspect that any missingness would depend on the true outcome values.
  - Application of RoB 2.0 Rules:
    - Since outcome data are available for all/most participants (Q3.1 = Yes), the predefined rule directly returns LOW risk of bias.

  Thus, based on the available information and applying the RoB 2.0 decision criteria, the risk of bias due to missing outcome data for the all-cause mortality outcome is LOW risk.

Domain 4: Risk of Bias in Measurement of the Outcome

- Signaling Question Analysis:

- 4.1 Was the method of measuring the outcome inappropriate? → No
  All-cause mortality is measured objectively (e.g., via hospital records or death certificates), making the method appropriate.
- 4.2 Could measurement or ascertainment of the outcome have differed between intervention groups? → No
  The ascertainment of mortality is objective and applied uniformly across groups.
- 4.3 Were outcome assessors aware of the intervention received by study participants? → Yes
  Given the open-label nature of the trial, outcome assessors likely knew the assigned interventions.
- 4.4 Could assessment of the outcome have been influenced by knowledge of intervention received? → No
  Because mortality is an objective outcome, assessor awareness is unlikely to have influenced the measurement.
- 4.5 Is it likely that assessment of the outcome was influenced by knowledge of intervention received? → No
  The objectivity of all-cause mortality minimizes any potential influence of assessor awareness.

- Risk Assessment: LOW risk
- Reasoning:
  - Appropriate Measurement Method: The method for measuring all-cause mortality is inherently objective, fulfilling the criteria for an appropriate measurement (4.1 = No).
  - Consistency Across Groups: There is no indication that the method of measurement differed between intervention groups (4.2 = No).
  - Assessor Awareness: Although outcome assessors were likely aware of the intervention (4.3 = Yes), the objective nature of the mortality outcome ensures that this awareness did not influence the measurement (4.4 = No; 4.5 = No).

Thus, applying the RoB 2.0 decision rules, the overall risk of bias due to measurement of the outcome is judged as LOW risk.

Domain 5: Risk of Bias in Selection of the Reported Result

- Signaling Question Analysis:
  - 5.1 Were the data that produced this result analysed in accordance with a pre-specified analysis plan? → No information
    The article does not provide details regarding a pre-specified analysis plan being finalized before unblinded outcome data were available.
  - 5.2 Is the numerical result being assessed likely to have been selected from multiple eligible outcome measurements? → No
    For all-cause mortality, the outcome is clearly defined and reported (e.g., 30-day mortality), with no indication that alternative measurements or definitions were available or selectively chosen.
  - 5.3 Is the numerical result being assessed likely to have been selected from multiple eligible analyses of the data? → No
    There is no evidence suggesting that multiple analyses were performed with selective reporting of results for all-cause mortality.
- Risk Assessment: SOME CONCERNS
- Reasoning:
  - Although the outcome measurement for all-cause mortality is straightforward and no multiple eligible measurements (5.2 = No) or analyses (5.3 = No) are apparent, the study does not specify whether a pre-specified analysis plan was finalized before unblinded data became available (5.1 = No information).

- According to the RoB 2.0 decision rules, when both the multiple outcome measurements and multiple analyses are rated as No and the pre-specified analysis plan is rated as No information, the appropriate risk judgment is SOME CONCERNS.
- This rating reflects uncertainty about potential selective reporting, even though the outcome itself is objectively defined.

ID: Bart 2005

Second Response

Domain 1: Bias Arising from the Randomization Process

- Signaling Question Analysis:
  - Was the allocation sequence random? → Y
    The study states that patients were randomized, which is taken as explicit evidence of randomization.
  - Was the allocation sequence concealed? → NI
    There is no explicit information provided regarding the methods used to conceal the allocation sequence.
  - Did baseline differences suggest a problem with randomization? → N
    Baseline characteristics appear comparable between the groups, with no substantial imbalances detected.
- Risk Assessment: Some Concerns
- Reasoning:
  - The trial explicitly mentions that patients were randomized (SQ1.1 = Y), satisfying the requirement for random sequence generation.
  - However, the study does not provide any details regarding the concealment of the allocation sequence (SQ1.2 = NI).
  - The baseline characteristics (SQ1.3 = N) do not indicate any substantial imbalances between the treatment groups.
  - According to the predefined RoB 2.0 decision rules, when the allocation sequence concealment is rated as NI and baseline imbalances are rated as N (Rule 4.1), the overall risk of bias for the randomization process is determined as Some Concerns.

Domain 2: Bias Due to Deviations from Intended Interventions

- Step 1: Analysis Type
  - The study explicitly states that all group comparisons were performed on an intention-to-treat (ITT) basis.
    → Classification: ITT analysis used.
- Step 2: Effect of Assignment to Intervention
  - 2.1 Were participants aware of their assigned intervention? → Yes
    Participants received distinctly different treatments (UF via catheter vs. usual care), so they were clearly aware of their assignment.
  - 2.2 Were carers and people delivering the interventions aware of participants' assigned intervention? → Yes
    Intervention delivery (placement of a catheter for UF, holding diuretics during UF) necessitated that the treating personnel know which treatment the patient was to receive.

- 2.3 Were there deviations from the intended intervention that arose because of the trial context? → No
  While two UF patients experienced technical issues (unsuccessful IV access and inability to withdraw blood), these are technical feasibility problems rather than deviations driven by the trial context (e.g., behavior changes due to awareness).
- 2.4 Were these deviations likely to have affected the outcome? → No
  Such technical issues are unlikely to influence an outcome like all-cause mortality, and they were incorporated in the ITT analysis.
- 2.5 Were these deviations from intended intervention balanced between groups? → No
  The UF group experienced two instances of technical failure, whereas the usual care group did not; however, these deviations are minor and do not reflect trial context–related departures from intended interventions.
- 2.6 Was an appropriate analysis used to estimate the effect of assignment to intervention? → Yes
  An ITT analysis was employed, which appropriately includes all randomized patients regardless of whether the intended intervention was fully delivered.
- 2.7 Was there potential for a substantial impact of the failure to analyze participants in the group to which they were randomized? → No
  All randomized participants were analyzed in their assigned groups, and the technical issues (though imbalanced) were unlikely to substantially bias the outcome.

- Risk Assessment
  - Part 1 (Effect of Assignment to Intervention):
    - Despite participants and personnel being aware of treatment assignment, the absence of trial context–driven deviations (2.3 = No) leads to a LOW risk judgment for this component.
  - Part 2 (Analysis Method):
    - The use of an ITT approach (2.6 = Yes) with no substantial impact from misclassifications (2.7 = No) supports a LOW risk judgment for this component.
  - Overall Domain 2 Risk:
    - Since Part 1 = LOW risk and Part 2 = LOW risk, the overall risk for Domain 2 is LOW risk.
- Reasoning:
  - The trial's use of ITT analysis minimizes bias from post-randomization exclusions.
  - Although both participants and treatment providers were aware of the assigned interventions (2.1 and 2.2 = Yes), there is no evidence that this awareness led to deviations driven by the trial context (2.3 = No).
  - The few technical issues encountered in the UF group (unbalanced in 2.5) did not appear to affect the outcome (2.4 = No) and were appropriately handled in the ITT framework.
- Thus, following the strict decision rules, Domain 2 is judged as LOW risk for bias due to deviations from intended interventions.

Domain 3: Risk of Bias Due to Missing Outcome Data

- Signaling Question Analysis:
  - 3.1 Were data for this outcome available for all, or nearly all, participants randomized? → Y
    All 40 randomized participants had their all-cause mortality outcome reported (with one death documented in the UF group), indicating nearly complete outcome data.

- 3.2 Is there evidence that the result was not biased by missing outcome data? → Y
  There is clear evidence that outcome data were complete, so missing data could not have biased the result.
- 3.3 Could missingness in the outcome depend on its true value? → N
  Since outcome data are available for nearly all participants, there is no indication that missingness depended on the true outcome.
- 3.4 Is it likely that missingness in the outcome depended on its true value? → N
  The complete follow-up minimizes any likelihood that missing data would depend on the true value of the outcome.

- Risk Assessment: LOW risk of bias
- Reasoning:
  - Outcome data for all participants were reported (SQ3.1 = Y), with clear evidence that missing data did not bias the results (SQ3.2 = Y).
  - There is no indication that missingness was related to the true value of the outcome (SQ3.3 = N and SQ3.4 = N).
  - According to the predefined decision rules, when outcome data are complete and there is no evidence of biased missingness, the risk of bias due to missing outcome data is LOW risk.

Domain 4: Risk of Bias in Measurement of the Outcome

- Signaling Question Analysis:
  - 4.1 Was the method of measuring the outcome inappropriate? → No
    All-cause mortality is an objective outcome typically measured via hospital records or death registries, which is appropriate.
  - 4.2 Could measurement or ascertainment of the outcome have differed between intervention groups? → No
    There is no indication that the method of determining mortality differed between the ultrafiltration and usual care groups.
  - 4.3 Were outcome assessors aware of the intervention received by study participants? → No information
    The report does not provide explicit details on whether outcome assessors were blinded; however, given the objective nature of mortality, this is less concerning.
  - 4.4 Could assessment of the outcome have been influenced by knowledge of intervention received? → No
    Because mortality is an objective measure, it is unlikely that knowledge of the intervention would influence its assessment.
  - 4.5 Is it likely that assessment of the outcome was influenced by knowledge of intervention received? → No
    There is no reason to suspect that the ascertainment of mortality was affected by the intervention assignment.
- Risk Assessment: LOW risk of bias
- Reasoning:
  - The measurement method for all-cause mortality is appropriate (4.1 = No) and is applied consistently across both intervention groups (4.2 = No).
  - Although there is no explicit information regarding the blinding of outcome assessors (4.3 = No information), the objective nature of the mortality outcome minimizes the potential for assessment bias.
  - Furthermore, the assessment is unlikely to have been influenced by knowledge of the intervention (4.4 = No and 4.5 = No).

- Following the decision rules, the overall risk of bias in the measurement of the outcome is therefore LOW risk.

Domain 5: Risk of Bias in Selection of the Reported Result

- Signaling Question Analysis:
  - 5.1 Were the data that produced this result analysed in accordance with a pre-specified analysis plan that was finalized before unblinded outcome data were available for analysis? → No information

    The report does not provide explicit details regarding a pre-specified analysis plan or confirmation that it was finalized prior to unblinding. There is no clear documentation in the methods or protocol regarding this aspect.
  - 5.2 Is the numerical result being assessed likely to have been selected, on the basis of the results, from multiple eligible outcome measurements within the outcome domain? → No

    All-cause mortality is an objective, binary outcome with no apparent alternative scales or definitions reported. The result (one death in the UF group) appears to be the sole measurement for this outcome.
  - 5.3 Is the numerical result being assessed likely to have been selected, on the basis of the results, from multiple eligible analyses of the data? → No

    There is no evidence suggesting that the mortality result was derived from multiple or selective analyses. The report presents the mortality data without indication of alternative analytical approaches.
- Risk Assessment: SOME CONCERNS
- Reasoning:
  - While there is no evidence of selective reporting in terms of multiple outcome measurements (5.2 = No) or multiple analyses of the data (5.3 = No), the absence of clear information regarding a pre-specified analysis plan (5.1 = No information) raises some concern.
  - According to the decision rules, when both 5.2 and 5.3 indicate no selective reporting but 5.1 is rated as No information (equivalent to N/PN/NI), the overall risk of bias for Domain 5 is judged as SOME CONCERNS.

ID: RECOVERY 2021

First Response

Domain 1: Bias Arising from the Randomization Process

- Signaling Question Analysis:
  - Was the allocation sequence random? → Y

    The study explicitly states that participants were "randomly allocated" via a web-based system.
  - Was the allocation sequence concealed? → Y

    The manuscript notes that allocation concealment was maintained until after randomisation.
  - Did baseline differences suggest a problem with randomization? → N

    Baseline characteristics across the groups were balanced with no substantial imbalances.
- Risk Assessment: Low
- Reasoning:
  - The allocation sequence was explicitly described as random (Y).
  - Secure allocation concealment was implemented (Y).
  - No baseline imbalances were observed (N).

- According to the RoB 2.0 decision rule:
  If allocation sequence concealed in {Y, PY} AND allocation sequence random in {Y, PY, NI} AND baseline imbalances in {N, PN, NI}, then risk is LOW.
- No corrections needed.

Domain 2: Bias Due to Deviations from Intended Interventions

- Step 1: Analysis Type
  - Analysis Type: ITT
    - The trial clearly states that the primary outcome was assessed in the intention-to-treat population, with no exclusions for non-adherence beyond missing outcome data.
- Step 2: Effect of Assignment to Intervention
  - 2.1 Were participants aware of their assigned intervention? → Y
    The trial is open-label; participants were explicitly aware of their treatment allocation.
  - 2.2 Were carers and intervention providers aware of the assignment? → Y
    Local study staff and treating clinicians were not masked to the treatment assignment.
  - 2.3 Did deviations from the intended intervention arise due to the trial context? → Y
    Deviations occurred: while 94% of patients in the azithromycin group received a macrolide, approximately 17% of patients in the usual care group also received a macrolide, reflecting treatment cross-overs inherent to the open-label design.
  - 2.4 Were these deviations likely to have affected the outcome? → N
    Despite the observed cross-over, the primary outcome (28-day mortality) was not substantially influenced, as evidenced by the null effect (rate ratio 0·97, 95% CI 0·87–1·07).
  - 2.5 Were these deviations balanced between groups? → N
    The deviations were imbalanced: almost all patients in the intervention arm received a macrolide compared with only about 17% in the usual care arm.
  - 2.6 Was an appropriate analysis used to estimate the effect of assignment to intervention? → Y
    The use of an intention-to-treat analysis is appropriate, as it preserves the benefits of randomization despite deviations.
  - 2.7 Was there potential for a substantial impact of the failure to analyze participants as randomized? → N
    The minimal post-randomization exclusions (with >97% follow-up) suggest that any potential impact is negligible.

- Risk Assessment for Step 2:
  - Part 1 (Effect of Assignment):
    - Since participants and carers were aware (Y), we examined deviations: deviations arose (Y) but were judged unlikely to affect outcome (N). However, because these deviations were not balanced (2.5 = N), this leads to Some Concerns.
  - Part 2 (Appropriate Analysis):
    - The ITT analysis was properly used (Y) and exclusions were minimal (2.7 = N), yielding Low Risk for this part.

- Overall Risk Judgment for Domain 2:
  - As Part 1 is judged Some Concerns and Part 2 is Low Risk, with no part reaching High Risk, the overall domain is assessed as Some Concerns.
- Reasoning:
  - The trial employed an ITT approach, which is a robust method for preserving randomization despite deviations. However, the open-label design meant that both participants and personnel were aware of treatment allocation. This led to deviations from the intended interventions, notably with a notable proportion of the usual care group receiving a macrolide antibiotic—a deviation that was not balanced between groups. Although these deviations were considered unlikely to have materially impacted the 28-day mortality outcome, their presence introduces some potential for bias. Given the appropriate analysis (ITT) and minimal exclusions, the overall risk due to deviations from the intended interventions is therefore judged as Some Concerns.

No logical inconsistencies were detected; no error corrections were necessary.

Domain 3: Risk of Bias Due to Missing Outcome Data

- Signaling Question Analysis:
  - 3.1 Were data for this outcome available for all, or nearly all, participants randomized? → Y
    Rationale: Outcome data (28-day mortality) were available for 97% of participants in the azithromycin group and 98% in the usual care group.
  - 3.2 Is there evidence that the result was not biased by missing outcome data? → Y
    Rationale: The very high follow-up rates indicate that missing data are minimal and unlikely to have introduced bias.
  - 3.3 Could missingness in the outcome depend on its true value? → N
    Rationale: Given that mortality is a hard outcome and the missingness is minimal, there is no indication that missing data depend on the actual outcomes.
  - 3.4 Is it likely that missingness in the outcome depended on its true value? → N
    Rationale: The reasons for missing data do not suggest a dependency on the true outcome values.
- Risk Assessment: Low Risk
- Reasoning:
  - Since nearly all participants had outcome data (3.1 = Y) and there is clear evidence that missing data did not bias the result (3.2 = Y), with no indication that missingness depended on the true outcome (3.3 = N; 3.4 = N), the domain meets the criteria for Low Risk according to the RoB 2.0 standards.

No inconsistencies were detected; therefore, no corrections were necessary.

Domain 4: Bias in Measurement of the Outcome

- Signaling Question Analysis:
  - 4.1 Was the method of measuring the outcome inappropriate? → No
    Rationale: The primary outcome is 28-day all-cause mortality—a hard, objective endpoint measured via routine data linkage—making the measurement method appropriate.
  - 4.2 Could measurement or ascertainment of the outcome have differed between intervention groups? → No
    Rationale: The ascertainment of mortality was consistent across both treatment groups.

- 4.3 Were outcome assessors aware of the intervention received by study participants? → No
  Rationale: Although the trial was open-label for participants and care providers, those responsible for outcome assessment were masked to treatment allocation.
- 4.4 Could assessment of the outcome have been influenced by knowledge of intervention received? → No
  Rationale: The objective nature of the outcome (mortality) and blinded outcome assessment minimize any potential influence.
- 4.5 Is it likely that assessment of the outcome was influenced by knowledge of intervention received? → No
  Rationale: Given the objective measurement and blinded assessors, it is unlikely that the outcome assessment was affected by knowledge of intervention.

- Risk Assessment: Low Risk
- Reasoning:
  The measurement of 28-day all-cause mortality is robust and objective. There is no evidence of differential outcome ascertainment between groups, and outcome assessors were masked to treatment allocation. Consequently, based on the RoB 2.0 criteria, the risk of bias in the measurement of the outcome is judged as Low Risk.

Domain 5: Bias in Selection of the Reported Result

- Signaling Question Analysis:
  - 5.1 Were the data that produced this result analysed in accordance with a pre-specified analysis plan? → Y
    Rationale: The trial report indicates that a protocol and statistical analysis plan were in place and publicly available (via trial registration and the study website), supporting that the analysis was pre-specified before unblinding.
  - 5.2 Is the numerical result being assessed likely to have been selected from multiple eligible outcome measurements within the outcome domain? → No
    Rationale: The primary outcome (28-day all-cause mortality) is clearly defined and standard, with no indication of selective choice among multiple outcome measures.
  - 5.3 Is the numerical result being assessed likely to have been selected from multiple eligible analyses of the data? → No
    Rationale: The analysis appears to follow the pre-specified plan without evidence of selective reporting of alternative analyses.
- Risk Assessment: Low Risk
- Reasoning:
  - Both multiple outcome measurements (5.2) and multiple analyses of the data (5.3) were judged as No, meaning there is no evidence of selective outcome or analysis reporting.

  - Since the trial was analysed in accordance with a pre-specified plan (5.1 = Y), the overall risk of bias in the selection of the reported result is Low Risk.

  No discrepancies or deviations from the pre-specified analysis plan were identified, so no corrections were necessary.

ID: RECOVERY 2021

Second Response

Domain 1: Bias Arising from the Randomization Process

- Signaling Question Analysis:
  - Was the allocation sequence random? → Y
    Evidence: The study explicitly states that patients were “randomly allocated” using a web-based simple (unstratified) randomization process.
  - Was the allocation sequence concealed? → Y
    Evidence: The trial report clearly indicates that allocation was “concealed until after randomisation.”
  - Did baseline differences suggest a problem with randomization? → N
    Evidence: Baseline characteristics were balanced between groups, with no substantial imbalances reported.
- Risk Assessment: Low risk
- Reasoning:
  - The allocation sequence was confirmed as random (Y) through explicit evidence of web-based simple randomization.
  - Allocation concealment was explicitly ensured (Y) as stated in the methods.
  - Baseline differences do not indicate any imbalance (N).
  - According to the RoB 2.0 decision rule: if allocation concealment is Y, the allocation sequence is random (Y) and there are no baseline imbalances (N), the overall risk of bias for this domain is classified as Low risk.

Domain 2: Bias Due to Deviations from Intended Interventions

- Analysis Type:
  - The study was analyzed on an intention-to-treat (ITT) basis.
- Step 2: Effect of Assignment to Intervention
  - 2.1 Were participants aware of their assigned intervention? → Y
    The trial was open-label; participants were aware of their treatment allocation.
  - 2.2 Were carers and people delivering the interventions aware of participants' assigned intervention? → Y
    Local study staff and treating clinicians were not masked to the treatment allocation.
  - 2.3 Did deviations from the intended intervention arise because of the trial context? → N
    Although some patients in the usual care arm received a macrolide (≈17%), such co-interventions reflect real-world practice rather than trial-specific deviations.
  - 2.4 Were these deviations likely to have affected the outcome? → N
    Given the objective primary outcome (28-day mortality) and the relatively small extent of cross-over, these deviations are unlikely to have impacted the outcome.
  - 2.5 Were these deviations from the intended intervention balanced between groups? → N
    There was a marked difference (≈94% adherence in the azithromycin arm vs. ≈17% in usual care); however, as this is inherent to the intervention assignment in a pragmatic trial, it does not indicate an imbalance in trial conduct.
  - 2.6 Was an appropriate analysis used to estimate the effect of assignment to intervention? → Y
    The study employed a proper ITT analysis for the primary outcome.
  - 2.7 Was there potential for a substantial impact of the failure to analyze participants in the groups to which they were randomized? → N
    High follow-up completeness (97–98%) minimizes the risk of substantial impact.

- Risk Assessment:
  - Part 1 (Effect of assignment):
    Since both participants and personnel were aware (Y) but deviations arising from the trial context were rated as "No" (N), rule 2.1 applies and Part 1 is classified as LOW risk.
  - Part 2 (Appropriate analysis):
    With an appropriate ITT analysis used (Y) and no substantial impact from exclusions (N), Part 2 is classified as LOW risk.
  - Overall Risk for Domain 2:
    As both Part 1 and Part 2 are LOW risk, the overall judgment for Domain 2 is LOW risk.
- Reasoning:
  - The trial was conducted and analyzed according to an ITT approach, which minimizes bias from post-randomization deviations.
  - Although the open-label design meant that participants and personnel were aware of the treatment allocation, the deviations (e.g., a small proportion of usual care patients receiving a macrolide) reflect real-world clinical practice rather than systematic trial-induced biases.
  - Furthermore, the objective nature of the primary outcome (28-day mortality) and the high adherence in the azithromycin arm ensure that any potential deviations had minimal likelihood to influence the results.
  - Consequently, with both the effect of assignment and the analytic approach meeting criteria for LOW risk, the overall risk of bias due to deviations from intended interventions is LOW risk.

Domain 3: Risk of Bias Due to Missing Outcome Data

- Signaling Question Analysis:
  - 3.1 Were data for this outcome available for all, or nearly all, participants randomized? → Y
    Evidence: Follow-up was available for 2506 (97%) patients in the azithromycin group and 5054 (98%) patients in the usual care group, indicating minimal missing data.
  - 3.2 Is there evidence that the result was not biased by missing outcome data? → Y
    Evidence: The high follow-up rate and use of routine data linkages for ascertainment of 28-day mortality support that missing data did not bias the results.
  - 3.3 Could missingness in the outcome depend on its true value? → N
    Rationale: Given the objective nature of the mortality outcome and the robust data collection methods, there is no indication that the missing data are related to the true outcome values.
  - 3.4 Is it likely that missingness in the outcome depended on its true value? → N
    Rationale: With very high follow-up and similar reasons for missing data across groups, it is unlikely that missingness depended on the actual mortality outcomes.
- Risk Assessment: Low risk of bias
- Reasoning:
  - The outcome data were available for nearly all participants (97–98% complete), which meets the criterion for minimal concern.
  - There is clear evidence that the missing data are unlikely to have introduced bias, given the objective nature of the primary outcome (28-day mortality) and the reliable data collection methods via routine health data linkages.
  - There is no indication that the missingness is related to the true outcome values.

- Following the RoB 2.0 decision rules, when outcome data are nearly complete and there is evidence against bias from missingness, the risk of bias due to missing outcome data is classified as Low risk.

Domain 4: Risk of Bias in Measurement of the Outcome

- Signaling Question Analysis:
    - 4.1 Was the method of measuring the outcome inappropriate? → No
      The outcome (28-day all-cause mortality) is an objective, hard endpoint measured via routine health data linkages, which is appropriate for this trial.
    - 4.2 Could measurement or ascertainment of the outcome have differed between intervention groups? → No
      The ascertainment of mortality was consistent across groups, with no evidence that measurement methods differed between the azithromycin and usual care arms.
    - 4.3 Were outcome assessors aware of the intervention received by study participants? → No
      Although the trial was open-label with respect to treatment allocation for participants and local staff, all individuals involved in the outcome data collection were masked to the intervention received.
    - 4.4 Could assessment of the outcome have been influenced by knowledge of intervention received? → No
      Given that outcome assessment was performed by masked assessors using objective data, it is unlikely that knowledge of treatment assignment could have influenced the measurement.
    - 4.5 Is it likely that assessment of the outcome was influenced by knowledge of intervention received? → No
      There is no indication that the assessment was influenced by intervention knowledge, as the measurement was objective and assessors were blinded.
- Risk Assessment: Low risk of bias
- Reasoning:
    - The measurement method for 28-day all-cause mortality is clearly appropriate and objective.
    - There is no evidence of differential measurement or ascertainment between groups.
    - Outcome assessors were masked to the treatment assignment, ensuring that their evaluation was not influenced by knowledge of the intervention.
    - Consequently, according to the RoB 2.0 decision rules, the risk of bias in the measurement of the outcome is judged to be Low risk.

Domain 5: Risk of Bias in Selection of the Reported Result

- Signaling Question Analysis:
    - 5.1 Were the data that produced this result analysed in accordance with a pre-specified analysis plan that was finalized before unblinded outcome data were available for analysis? → Y
      Evidence: The protocol and statistical analysis plan were pre-specified and made available on the study website. The trial steering committee, masked to the results, determined key aspects (e.g., sample size) and closed recruitment accordingly, supporting that the plan was finalized before unblinding.
    - 5.2 Is the numerical result being assessed likely to have been selected, on the basis of the results, from multiple eligible outcome measurements (e.g. scales, definitions, time points) within the outcome domain? → N
      Rationale: The primary outcome (28-day all-cause mortality) was clearly defined a priori, and there is no indication that alternative measurements or definitions were available or selectively reported.

- 5.3 Is the numerical result being assessed likely to have been selected, on the basis of the results, from multiple eligible analyses of the data? → N
  Rationale: The analysis was conducted following the pre-specified plan using standard statistical methods (e.g., ITT analysis, log-rank test), with no evidence of selective reporting among multiple analyses.

- Risk Assessment: Low risk of bias
- Reasoning:
  - The trial was analyzed according to a pre-specified analysis plan that was finalized before unblinded data were available (5.1 = Y).
  - There is no indication of selective reporting based on multiple outcome measurements (5.2 = N) or from multiple analyses of the data (5.3 = N).
  - According to the RoB 2.0 decision rules, when both multiple outcome measurements and multiple analyses are not present (i.e., rated as N) and the trial was analyzed in accordance with a pre-specified plan (Y), the risk of bias due to selective reporting is classified as Low risk.

ID: Van Stralen 2013

First Response

Domain 1: Bias Arising from the Randomization Process

- Signaling Question Analysis:
  - Was the allocation sequence random? → Y
    The study explicitly states that women were equally randomized (1:1) to receive misoprostol or placebo.
  - Was the allocation sequence concealed? → Y
    Allocation concealment was implemented by drawing a blank envelope containing the study medication prepared by the pharmacist, ensuring proper concealment.
  - Did baseline differences suggest a problem with randomization? → Y
    There was a statistically significant imbalance in parity (35/48 nulliparous in the misoprostol group vs. 23/51 in the placebo group, $p = 0.003$), indicating a potential issue.
- Risk Assessment: SOME CONCERNS
- Reasoning:
  - The study provides explicit evidence of a random allocation sequence (SQ 1.1: Y) and proper allocation concealment (SQ 1.2: Y).
  - However, the notable imbalance in baseline parity (SQ 1.3: Y) suggests that the randomization process may have been compromised regarding this characteristic.
  - Following the RoB 2.0 rules, when both randomization and concealment are rated as Y but there is a significant baseline imbalance (Y), the overall risk of bias for this domain is classified as SOME CONCERNS.

Domain 2: Bias Due to Deviations from Intended Interventions

- Step 1: Analysis Approach

- Analysis Type: The study explicitly states that “all patients remained in the study on an intention-to-treat principle.”
  → Classification: ITT
- Step 2: Effect of Assignment to Intervention
  - 2.1. Participant Awareness:
    Response: No
    Rationale: The study is described as double-blind, indicating that participants were not aware of their assigned intervention.
  - 2.2. Carer/Provider Awareness:
    Response: No
    Rationale: As a double-blind trial, the intervention providers (carers) were not aware of the treatment assignments.
  - 2.3. Deviations Arising from Trial Context:
    Response: No
    Rationale: No deviations from the intended intervention were reported as arising due to the trial context. Although a few “safety envelopes” were opened for breastfeeding concerns early in the trial, these were infrequent and did not constitute a systematic deviation.
  - 2.4. Impact of Deviations on Outcome:
    Response: No
    Rationale: The minor potential unblinding (via side effects) did not appear to have influenced the primary outcome (manual removal of the placenta).
  - 2.5. Balance of Deviations Between Groups:
    Response: Yes
    Rationale: Any deviations (e.g., opening of safety envelopes) occurred in both groups in a similar manner.
  - 2.6. Appropriateness of the Analysis:
    Response: Yes
    Rationale: The use of an intention-to-treat analysis is appropriate, and exclusions were only pre-randomization; hence the analysis correctly estimates the effect of assignment.
  - 2.7. Potential Substantial Impact of Analysis Failure:
    Response: No
    Rationale: There is no evidence that any post-randomization exclusions or deviations substantially impacted the study’s results.

  → Part 1 (Effect of Assignment): LOW risk
  → Part 2 (Appropriateness of Analysis): LOW risk
  Since both parts are LOW risk, the overall assessment for the effect of assignment to intervention is LOW risk.
- Step 3: Effect of Adhering to Intervention
  (Not applicable, as the study used ITT analysis.)
- Risk Assessment: LOW risk
- Reasoning:
  - The trial employed an intention-to-treat analysis, minimizing bias from post-randomization exclusions.
  - Effective double-blinding ensured that neither participants nor caregivers were aware of the assignments, reducing the likelihood of deviations.

- Any minor deviations (e.g., safety envelope openings) were balanced between groups and were not deemed to have affected the primary outcome.
- Thus, both the effect of assignment and the analysis method meet criteria for LOW risk under RoB 2.0.

Domain 3: Risk of Bias Due to Missing Outcome Data

- Signaling Question Analysis:
  - 3.1 Were data for this outcome available for all, or nearly all, participants randomized? → Yes
    The study reports that complete outcome data for the primary outcome (manual removal of the placenta) were available for all 99 randomized participants.
  - 3.2 Is there evidence that the result was not biased by missing outcome data? → Yes
    With complete outcome data available, there is clear evidence that missing data did not bias the results.
  - 3.3 Could missingness in the outcome depend on its true value? → No
    Since no outcome data are missing, it is unlikely that missingness depended on the true outcome values.
  - 3.4 Is it likely that missingness in the outcome depended on its true value? → No
    As there is no missing data, there is no likelihood that missingness was related to the outcome.
- Risk Assessment: LOW risk
- Reasoning:
  - The study provides complete outcome data for the primary outcome, ensuring that all randomized participants were included in the analysis.
  - With 100% data availability for the primary endpoint, there is no evidence of bias due to missing data, nor any plausible mechanism by which missingness could have affected the outcome.
  - Following the RoB 2.0 guidelines, this results in a LOW risk judgment for Domain 3.

Domain 4: Risk of Bias in Measurement of the Outcome

- Signaling Question Analysis:
  - 4.1 Was the method of measuring the outcome inappropriate? → No
    The outcome (manual removal of the placenta) is determined by a clear, protocol-driven clinical decision, making the measurement method appropriate.
  - 4.2 Could measurement or ascertainment of the outcome have differed between intervention groups? → No
    Both groups followed the same protocol (a final attempt at 45 minutes followed by manual removal if unsuccessful), ensuring consistent measurement across groups.
  - 4.3 Were outcome assessors aware of the intervention received by study participants? → Probably no
    The trial was described as double-blind, so outcome assessors were likely blinded, although side effects (e.g., shivering) might have provided subtle cues. However, there is no clear evidence that such cues affected assessment.
  - 4.4 Could assessment of the outcome have been influenced by knowledge of intervention received? → No
    Given the objective nature of the outcome (a clear clinical decision based on protocol), it is unlikely that any potential awareness would have influenced the outcome assessment.
  - 4.5 Is it likely that assessment of the outcome was influenced by knowledge of intervention received? → No
    Even if minor cues were present, they are unlikely to have meaningfully influenced the decision to perform manual removal.

- Risk Assessment: LOW risk
- Reasoning:
  - The measurement of the primary outcome is objective and based on a strict protocol, ensuring consistency between groups (4.1: No; 4.2: No).
  - Although the study is double-blind, a small possibility exists that side effects could have provided hints about treatment allocation; however, this is judged as "Probably no" (4.3), and the objective nature of the outcome minimizes any potential bias (4.4 and 4.5: No).
  - Following the RoB 2.0 decision rules, with appropriate measurement methods and consistent assessment across groups, the overall risk of bias in the measurement of the outcome is classified as LOW risk.

Domain 5: Risk of Bias in Selection of the Reported Result

- Signaling Question Analysis:
  - 5.1 Were the data that produced this result analysed in accordance with a pre-specified analysis plan finalized before unblinding? → Probably yes
    The study was registered (EudraCT number provided) and the statistical methods are clearly described, which suggests that a pre-specified analysis plan existed even though explicit confirmation of finalization before unblinding is not provided.
  - 5.2 Is the numerical result being assessed likely to have been selected from multiple eligible outcome measurements within the outcome domain? → No
    The primary outcome (manual removal of the placenta) is clearly defined, and secondary outcomes are explicitly stated, with no evidence of selective outcome measurement.
  - 5.3 Is the numerical result being assessed likely to have been selected from multiple eligible analyses of the data? → No
    Standard statistical tests (chi-squared test and independent samples t-test) were used as described in the methods section, with no indication of selective analysis reporting.
- Risk Assessment: LOW risk
- Reasoning:
  - With a pre-specified analysis plan indicated by trial registration and a clearly defined statistical methods section (SQ 5.1: Probably yes), and no evidence of selective reporting regarding outcome measurements (SQ 5.2: No) or analyses (SQ 5.3: No), the study meets the criteria for LOW risk according to RoB 2.0 guidelines.
  - Following the decision rules: since both potential issues of multiple outcome measurements and multiple analyses are rated as No, and the trial appears to adhere to a pre-specified plan (even if only "Probably yes"), the overall judgment is LOW risk.

ID: Van Stralen 2013

Second Response

Domain 1: Bias Arising from the Randomization Process

- Signaling Question Analysis:
  - Was the allocation sequence random? → Y
    The study explicitly states that women were "equally randomized (1:1)" to misoprostol or placebo.

- Was the allocation sequence concealed? → Y
  Allocation was performed via a blank envelope system prepared by the pharmacy using a single number-coded container, indicating secure concealment.
- Did baseline differences suggest a problem with randomization? → Y
  There was a statistically significant imbalance in nulliparity (35/48 vs. 23/51, p = 0.003), which is considered a substantial baseline imbalance.

- Risk Assessment: Some Concerns
- Reasoning:
  - The allocation sequence was explicitly randomized (SQ 1.1 = Y) and secure concealment was reported (SQ 1.2 = Y).
  - However, the presence of significant baseline imbalances in nulliparity (SQ 1.3 = Y) indicates that the randomization process may not have fully balanced prognostic factors between groups.
  - According to the RoB 2.0 decision rules, when allocation concealment is secure and randomization is confirmed, but there are substantial baseline imbalances, the overall risk of bias for this domain should be judged as Some Concerns.
  - No logical inconsistency was detected in applying the decision rules, and the final assessment aligns with the predefined criteria.

Domain 2: Bias Due to Deviations from Intended Interventions

- Step 1: Analysis Type
  The study explicitly states that "all patients remained in the study on an intention-to-treat principle," indicating that an ITT analysis was used.
- Signaling Question Analysis (Effect of Assignment to Intervention):
  - 2.1 Were participants aware of their assigned intervention during the trial? → No
    The trial was described as double-blind, and despite the potential for side effects (e.g., gastrointestinal complaints, shivering) to hint at group assignment, there is no evidence that participants clearly knew their allocation.
  - 2.2 Were carers and people delivering the interventions aware of participants' assigned intervention during the trial? → No
    As a double-blind, placebo-controlled trial with study medication prepared in a blinded fashion, there is no indication that intervention providers were aware of assignments.
  - 2.3 Were there deviations from the intended intervention that arose because of the trial context? → No
    Apart from the opening of a few "safety envelopes" for breastfeeding concerns (which occurred in 7 cases and were balanced between groups), no deviations related to trial context were reported.
  - 2.4 Were these deviations likely to have affected the outcome? → No
    The minor deviations (safety envelope openings) were not linked to the primary outcome (manual removal of the placenta) and did not influence treatment administration.
  - 2.5 Were these deviations from intended intervention balanced between groups? → Yes
    The few safety envelope openings were nearly evenly distributed (4 in the misoprostol group vs. 3 in the placebo group).

- 2.6 Was an appropriate analysis used to estimate the effect of assignment to intervention? → Yes
  The study employed an ITT analysis, which is appropriate for estimating the effect of assignment, with exclusions limited to missing outcome data.
- 2.7 Was there potential for a substantial impact of the failure to analyze participants in the group to which they were randomized? → No
  There is no indication that any post-randomization exclusions or misclassifications were large enough to affect the results.

- Risk Assessment: LOW risk
- Reasoning:
  - Analysis Type: An intention-to-treat (ITT) analysis was used, ensuring that all randomized participants were included in the analysis regardless of subsequent events.
  - Blinding: Both participants and personnel were blinded (2.1 and 2.2 = No), reducing the chance that awareness of intervention assignment influenced behavior or management.
  - Deviations: The only notable deviation was the opening of safety envelopes (for breastfeeding concerns), which was minimal, balanced across groups (2.5 = Yes), and did not affect the primary outcome (2.4 = No).
  - Appropriate Analysis: The ITT approach (2.6 = Yes) and the absence of significant misclassification or exclusion effects (2.7 = No) further support the conclusion that the effect of assignment was appropriately estimated.
  - Final Judgment: With both the effect of assignment (Part 1) and the analysis (Part 2) assessed at low risk, the overall risk of bias in Domain 2 is LOW risk.

Domain 3: Risk of Bias Due to Missing Outcome Data

- Signaling Question Analysis:
  - 3.1 Were data for this outcome available for all, or nearly all, participants randomized? → Yes
    Complete data for the primary outcome (manual removal of the placenta) were available for all 99 women included in the trial, indicating minimal missing data.
  - 3.2 Is there evidence that the result was not biased by missing outcome data? → Yes
    Since outcome data were fully available, there is clear evidence that the result was not biased by missing data.
  - 3.3 Could missingness in the outcome depend on its true value? → No
    With no missing data for the primary outcome, there is no indication that missingness could depend on its true value.
  - 3.4 Is it likely that missingness in the outcome depended on its true value? → No
    Given the complete outcome data, it is unlikely that any missingness depended on the true outcome values.
- Risk Assessment: LOW risk of bias
- Reasoning:
  - Outcome Data Completeness: The study clearly reports that complete data were available for all participants (n = 99) with respect to the primary outcome (manual removal).
  - Bias Evaluation: With all outcome data collected, there is strong evidence that missing data did not introduce bias into the results.
  - Missingness Dependency: The absence of missing data means there is no concern about missingness being related to the true outcome.

- Final Judgment: According to the RoB 2.0 criteria, when outcome data are available for all (or nearly all) participants and there is clear evidence that missing data did not bias the results, the risk of bias in this domain is assessed as LOW risk.

Domain 4: Risk of Bias in Measurement of the Outcome

- Signaling Question Analysis:
  - 4.1 Was the method of measuring the outcome inappropriate? → No
    Manual removal of the placenta was defined by a clear protocol (failure of the final attempt after 45 minutes), making the measurement method appropriate.
  - 4.2 Could measurement or ascertainment of the outcome have differed between intervention groups? → No
    Both groups were managed using the same protocol for assessing whether manual removal was needed, ensuring consistency across groups.
  - 4.3 Were outcome assessors aware of the intervention received by study participants? → No
    The trial was double-blind, so outcome assessors were not aware of the participants' intervention assignments.
  - 4.4 Could assessment of the outcome have been influenced by knowledge of intervention received? → No
    With effective blinding and an objective outcome measure, assessment was unlikely to be influenced by intervention knowledge.
  - 4.5 Is it likely that assessment of the outcome was influenced by knowledge of intervention received? → No
    Given the clear protocol and blinding, it is unlikely that any influence occurred.
- Risk Assessment: LOW risk
- Reasoning:
  - The outcome measurement (manual removal of the placenta) was objectively defined and implemented via a clear, protocol-driven process, ensuring that the method was appropriate (4.1 = No).
  - Measurement procedures were consistent across both intervention groups, with no evidence of differences in outcome ascertainment (4.2 = No).
  - The study's double-blind design ensured that outcome assessors were unaware of the treatment assignments (4.3 = No).
  - Consequently, there is no indication that assessment was or could have been influenced by knowledge of the intervention (4.4 and 4.5 = No).
  - Following the predefined RoB 2.0 rules, the appropriate and consistent measurement method coupled with effective blinding results in a LOW risk of bias in this domain.

Domain 5: Risk of Bias in Selection of the Reported Result

- Signaling Question Analysis:
  - 5.1 Were the data that produced this result analysed in accordance with a pre-specified analysis plan finalized before unblinded outcome data were available for analysis? → Probably yes
    The trial was registered (EudraCT number 2006-006371-20) and the methods section outlines the planned statistical analyses (e.g., chi-squared test for dichotomous variables, independent samples t-test for continuous variables) and the use of an intention-to-treat approach. Although the publication does not explicitly state that the analysis plan was finalized before unblinding, the presence of trial registration and detailed methods implies that a pre-specified plan likely existed.

- 5.2 Is the numerical result being assessed likely to have been selected, on the basis of the results, from multiple eligible outcome measurements (e.g., scales, definitions, time points) within the outcome domain? → No
  The primary outcome (manual removal of retained placenta) was clearly defined in the protocol, and there is no indication that multiple eligible measurements were available or selectively reported. Secondary outcomes were also pre-specified and reported.
- 5.3 Is the numerical result being assessed likely to have been selected, on the basis of the results, from multiple eligible analyses of the data? → No
  The statistical analysis methods are clearly described and appear to follow standard approaches without evidence of selective analysis. There is no indication that the authors explored multiple alternative analyses and selectively reported favorable results.

- Risk Assessment: LOW risk of bias
- Reasoning:
  - Pre-specified Analysis Plan (5.1): The study was registered and the methods section provides detailed statistical procedures, supporting the likelihood that a pre-specified analysis plan was in place before unblinded data were available (5.1 = Probably yes).
  - Outcome Measurement Selection (5.2): The primary outcome was clearly defined, and there is no evidence of selective reporting among multiple outcome measurements (5.2 = No).
  - Analysis Selection (5.3): The reported analyses are consistent with those described in the methods, with no indication of multiple alternative analyses being conducted or selectively reported (5.3 = No).
  - Final Judgment: Since both the possibility of multiple outcome measurements and multiple analyses have been ruled out (both in {N, PN}), and the study appears to have been analyzed in accordance with a pre-specified plan (5.1 = Probably yes), the overall risk of bias in the selection of the reported result is assessed as LOW risk.

ID: Takezawa 2004

First Response

Domain 1: Bias Arising from the Randomization Process

- Signaling Question Analysis:
  - Was the allocation sequence random? → Y The study clearly states that patients were "Randomized to EPBD (n = 104)" and "Randomized to EST (n = 96)," which is taken as explicit evidence of randomization.
  - Was the allocation sequence concealed? → NI There is no information provided regarding the method of concealment (e.g., sealed envelopes, central allocation), so this is marked as "No Information."
  - Did baseline differences suggest a problem with randomization? → N No evidence of substantial baseline imbalances is noted in the provided information.
- Risk Assessment: Some Concerns
- Reasoning:
  - The study explicitly indicates that patients were randomized, thus the allocation sequence is considered random (Y).
  - However, there is no information regarding the concealment of the allocation sequence, which is recorded as No Information (NI).

- There is no indication of substantial baseline imbalances (N).
- According to the RoB 2.0 decision rules, when the allocation concealment is "No Information" and baseline imbalances are not present (N), the overall risk of bias for this domain should be rated as Some Concerns.

Domain 2: Bias Due to Deviations from Intended Interventions

Because the trial's reporting suggests that many participants were excluded post-randomization for reasons beyond mere missing outcome data (e.g. nonacceptance for ERCP and cholecystectomy), the analysis appears to follow a per-protocol approach. Consequently, we assess the effect of adhering to the intervention (Step 3).

- Signaling Question Analysis (Effect of Adhering to Intervention):
  - Were participants aware of their assigned intervention? → Y In interventional procedures such as EPBD versus EST, it is highly likely that participants knew which intervention they received.
  - Were carers and people delivering the interventions aware? → YThe clinicians performing these procedures would necessarily be aware of the assignment.
  - Were important non-protocol interventions balanced across groups? → Y For example, the numbers of patients undergoing cholecystectomy (EPBD: 19; EST: 16) appear roughly balanced.
  - Were there failures in implementing the intervention that could have affected the outcome? → N Among the analyzed participants, there is no clear report of implementation failures beyond the exclusions already noted.
  - Was there non-adherence to the assigned intervention that could have affected outcomes? → Y The substantial post-randomization exclusions (resulting in 23/104 and 21/96 patients at 1-year follow-up) indicate notable non-adherence or deviations.
  - Was an appropriate analysis used to estimate the effect of adhering to intervention? → N The trial appears to have relied on a per-protocol analysis without employing methods (e.g. instrumental variables or inverse probability weighting) that might mitigate bias from non-adherence.
- Risk Assessment: HIGH risk
- Reasoning:
  - The substantial exclusions after randomization—stemming from factors such as nonacceptance for ERCP and undergoing cholecystectomy—suggest that the trial did not maintain an intention-to-treat framework but rather implemented a per-protocol analysis.
  - Both participants and personnel were aware of the assigned intervention, a common circumstance in procedural trials, which may further contribute to deviations.
  - Although non-protocol interventions (like cholecystectomy) appear balanced between groups, the evidence of considerable non-adherence is concerning.
  - Critically, the analysis did not employ appropriate methods to account for these deviations (i.e. it used a straightforward per-protocol approach), thereby potentially introducing bias.
  - In light of these factors and following the predefined decision rules, the effect of adhering to intervention is judged to be at HIGH risk of bias.

Domain 3: Risk of Bias Due to Missing Outcome Data

- Signaling Question Analysis:
  - 3.1 Were data for this outcome available for all, or nearly all, participants randomized? → N Only 44 of 200 randomized participants (23 in EPBD and 21 in EST) had 1-year follow-up data, representing a very small fraction (~22%).

- 3.2 Is there evidence that the result was not biased by missing outcome data? → N There is no clear evidence provided that the missing data did not introduce bias, and the analysis does not appear to include robust bias-mitigating methods.
- 3.3 Could missingness in the outcome depend on its true value? → PY Given the substantial loss to follow-up, it is plausible that the reasons for missing data are related to the outcome (for example, participants with adverse outcomes might be more likely to be lost).
- 3.4 Is it likely that missingness in the outcome depended on its true value? → PY The extent of missing data strongly suggests that the missingness may well depend on the unobserved outcomes.

- Risk Assessment: HIGH risk
- Reasoning:
  - Outcome Data Availability (3.1): Only about 22% of the participants originally randomized have outcome data available, which is far below the threshold for "nearly all" (typically ≥95% for continuous outcomes).
  - Evidence Against Bias (3.2): There is no convincing evidence that the missing data were handled in a manner that minimizes bias.
  - Missingness Dependency (3.3 & 3.4): It is plausible—and likely—that the missing outcome data are related to the true outcome values. The uncertainty regarding the reasons for loss to follow-up, combined with the high proportion of missing data, suggests that the missingness could have influenced the estimated effect.
  - Final Judgment: Following the RoB 2.0 decision rules, because the missing data are both substantial and likely related to the true outcomes, this domain is judged to be at HIGH risk of bias.

Domain 4: Risk of Bias in Measurement of the Outcome

- Signaling Question Analysis:
  - Was the method of measuring the outcome inappropriate? → NRecurrent common bile duct stones are typically assessed using objective clinical and imaging criteria, which are appropriate for this outcome.
  - Could measurement or ascertainment of the outcome have differed between intervention groups? → N The study appears to have applied consistent measurement methods across both intervention groups.
  - Were outcome assessors aware of the intervention received by study participants? → NI The report does not provide explicit details on the blinding status of outcome assessors.
  - Could assessment of the outcome have been influenced by knowledge of intervention received? → N Given the objective nature of the outcome (i.e. recurrent stones confirmed by clinical/imaging methods), any potential influence is unlikely.
  - Is it likely that assessment of the outcome was influenced by knowledge of intervention received? → N It is unlikely that outcome assessment was influenced by intervention knowledge, due to the objective measurement approach.
- Risk Assessment: LOW risk
- Reasoning:
  - The measurement method is appropriate (4.1 = N) and is applied consistently across groups (4.2 = N).
  - Although there is no explicit information about the blinding of outcome assessors (4.3 = NI), the objective nature of the outcome minimizes the potential for bias.
  - Both the potential influence of intervention knowledge (4.4 = N) and the likelihood of such influence (4.5 = N) are judged to be minimal.
  - Following the predefined decision rules, these responses yield an overall judgment of LOW risk for Domain 4.

Domain 5: Risk of Bias in Selection of the Reported Result

- Signaling Question Analysis:
  - 5.1 Analysis Plan Finalization: → No information (NI) The study report does not provide details on whether a pre-specified analysis plan was finalized before unblinded outcome data were available.
  - 5.2 Multiple Outcome Measurements: → No There is no evidence that the numerical result (primary outcome at 1 year) was selected from multiple eligible outcome measurements. The outcome appears to have been reported as pre-specified.
  - 5.3 Multiple Analyses of Data: → No There is no indication that the reported result was chosen from among multiple eligible analyses; all analyses appear to have been reported without selective emphasis.
- Risk Assessment: SOME CONCERNS
- Reasoning:
  - Although there is no evidence of selective reporting regarding outcome measurements (5.2 = No) or analyses (5.3 = No), there is a lack of information about whether the analysis plan was pre-specified and finalized before unblinding (5.1 = NI).
  - Following the decision rules, when both multiple outcome measurements and analyses are not a concern (i.e., rated as No) but there is no information on the analysis plan (NI), the overall judgment is SOME CONCERNS for Domain 5.

ID: Takezawa 2004

Second Response

Domain 1: Bias Arising from the Randomization Process

- Signaling Question Analysis:
  - Was the allocation sequence random? → Yes The study explicitly states that patients were "Randomized to EPBD (n = 104)" and "Randomized to EST (n = 96)," which serves as explicit evidence of randomization.
  - Was the allocation sequence concealed? → No information (NI)There is no mention or description of the methods used to conceal the allocation sequence.
  - Did baseline differences suggest a problem with randomization? → No information (NI) The report does not provide details on baseline characteristics that would allow an assessment of imbalances between groups.
- Risk Assessment: Some Concerns
- Reasoning:
  - The allocation sequence was explicitly randomized (SQ1.1: Yes).
  - There is no information regarding allocation concealment (SQ1.2: NI).
  - There is also no information on baseline differences (SQ1.3: NI).
  - According to the decision rule (Rule 4.1): When the allocation sequence is rated NI and baseline differences are in {N, PN, NI} (here, NI), the overall risk of bias should be judged as Some Concerns.

  No error was detected based on the predefined RoB 2.0 decision rules.

Domain 2: Bias Due to Deviations from Intended Interventions

- Analysis Type Determination (Step 1):

- Analysis Classification: Per-protocol Rationale: The study's CONSORT diagram shows that after randomization, several participants were excluded (e.g., nonacceptance for ERCP, untraceable, cholecystectomy), indicating exclusions for reasons other than solely missing outcome data. This pattern is consistent with a per-protocol (PP) analysis rather than a full intention-to-treat approach.

- Since the analysis is PP, we follow the "effect of adhering to intervention" pathway (Step 3).
- Signaling Question Analysis (Step 3 – Effect of Adhering to Intervention):
  - 2.1 Were participants aware of their assigned intervention during the trial? → Yes Given the nature of the interventional procedures (EPBD vs. EST), participants were clearly aware of which procedure they underwent.
  - 2.2 Were carers and people delivering the interventions aware of participants' assigned intervention during the trial? → Yes The procedural nature of the interventions implies that the healthcare providers (carers/interventionists) were aware of the assignments.
  - 2.3 Were important non-protocol interventions balanced across intervention groups? → No information (NI) The report does not provide clear details on any additional non-protocol interventions, leaving balance uncertain.
  - 2.4 Were there failures in implementing the intervention that could have affected the outcome? → No There is no explicit evidence suggesting that the interventions were not implemented as intended for those who received them.
  - 2.5 Was there non-adherence to the assigned intervention regimen that could have affected participants' outcomes? → No information (NI) Although several participants were excluded post-randomization, there is insufficient detail to determine whether non-adherence among those receiving the intervention influenced outcomes.
  - 2.6 Was an appropriate analysis used to estimate the effect of adhering to intervention? → No The study used a per-protocol approach with exclusions for reasons other than solely missing outcome data. Such exclusions (for non-adherence, protocol violations, or post-randomization decisions) indicate that the analysis method is inappropriate for preserving the benefits of randomization.
- Risk Assessment for Domain 2: HIGH risk
- Reasoning:
  - The study was conducted as a per-protocol analysis (PP) rather than a full intention-to-treat analysis because multiple post-randomization exclusions were made for reasons (e.g., nonacceptance for ERCP, cholecystectomy) that go beyond mere missing outcome data.
  - In the effect of adhering to intervention pathway:
    - Both participants and the healthcare providers were clearly aware of the intervention assignments (answers: Yes), which by itself can predispose to bias.
    - The balance of any non-protocol interventions is unclear (NI), so we cannot assume that any extra care or co-interventions were evenly distributed.
    - Although there is no clear evidence of failures in implementation (answer: No) or explicit reporting of non-adherence (NI), the crucial point is the use of an inappropriate analysis method.
    - Because the analysis method to estimate the effect of adhering was judged as inappropriate (No), following the decision tree for PP analyses—when non-protocol deviations are either unclear or the appropriate analysis is not used—the risk of bias is elevated.

- According to the decision rule: In cases where, after establishing that participants and personnel were aware, the non-protocol interventions are not confirmed as balanced (NI) and the analysis used to estimate the effect of adhering is inappropriate (No), the risk should be classified as HIGH risk.

No logical inconsistencies were detected in applying the predefined RoB 2.0 rules; therefore, the final risk judgment for Domain 2 is HIGH risk.

Domain 3: Risk of Bias Due to Missing Outcome Data

- Signaling Question Analysis:
  - Were data for this outcome available for all, or nearly all, participants randomized? → No Only 23 participants in the EPBD group and 21 in the EST group had 1-year follow-up data (≈22% of the randomized sample), which is far below the ≥95% threshold required for minimal concern.
  - Is there evidence that the result was not biased by missing outcome data? → No The report does not provide clear evidence or sensitivity analyses to demonstrate that the missing data did not bias the results.
  - Could missingness in the outcome depend on its true value? → Probably yes Given the substantial missing data, it is plausible that the reasons for missing follow-up (e.g., clinical events, reinterventions, or other factors) might be related to the true outcome.
  - Is it likely that missingness in the outcome depended on its true value? → Probably yes The uncertainty and large proportion of missing outcome data suggest that the missingness is likely related to the actual outcome values.
- Risk Assessment: HIGH risk
- Reasoning:
  - Availability of Data (SQ 3.1): With outcome data available for only about 22% of the participants, the condition for “all or nearly all” (i.e., ≥95%) is not met.
  - Evidence Against Bias (SQ 3.2): There is no provided evidence to assure that the missing data did not introduce bias.
  - Potential for Outcome-Dependent Missingness (SQ 3.3 & 3.4):It is plausible—and likely—that the missingness depended on the true outcome values, as the reasons for missing follow-up could be related to recurrence of stones or related adverse events.
  - Application of Decision Rules:
    - Since outcome data are not available for nearly all participants (SQ 3.1: No) and there is no evidence that missing data did not bias the results (SQ 3.2: No), we must consider whether missingness could depend on true outcomes.
    - With SQ 3.3 answered as “Probably yes” and SQ 3.4 as “Probably yes,” the decision rule directs us to classify the risk as HIGH.

Thus, based on the RoB 2.0 criteria, the risk of bias due to missing outcome data in this study is HIGH risk.

Domain 4: Risk of Bias in Measurement of the Outcome

- Signaling Question Analysis:
  - 4.1 Was the method of measuring the outcome inappropriate?→ No Although the study does not detail the measurement method explicitly, the primary outcome—recurrent common bile duct stones at 1 year—is an objective clinical event typically determined by standard diagnostic criteria (e.g., imaging or clinical evaluation). There is no indication that the method was inappropriate.

    - 4.2 Could measurement or ascertainment of the outcome have differed between intervention groups? → No There is no suggestion that the method of outcome measurement differed between the EPBD and EST groups.
    - 4.3 Were outcome assessors aware of the intervention received by study participants? → No information The report does not provide explicit details regarding the blinding of outcome assessors. However, given the objective nature of the outcome, any potential unblinding is less likely to introduce bias.
    - 4.4 Could assessment of the outcome have been influenced by knowledge of intervention received? → No Because the outcome is objectively defined (i.e., recurrence of stones) and there is no indication that subjective judgment played a role, it is unlikely that knowledge of the intervention would have influenced the outcome assessment.
    - 4.5 Is it likely that assessment of the outcome was influenced by knowledge of intervention received? → No In line with the previous point, the objective nature of the outcome minimizes the likelihood of bias from assessor awareness.
- Risk Assessment: LOW risk
- Reasoning:
    - The measurement method for the outcome is judged appropriate (4.1: No inappropriate method).
    - There is no evidence that measurement differed between groups (4.2: No).
    - Although details on assessor blinding are not provided (4.3: No information), the objective nature of the outcome reduces potential bias.
    - There is no indication that the assessment could have been influenced by knowledge of the intervention (4.4 and 4.5: No).
    - Following the decision rules—since the measurement method is appropriate, consistent across groups, and the potential for influence is minimal—the overall risk of bias in measurement of the outcome is classified as LOW risk.

Domain 5: Risk of Bias in Selection of the Reported Result

- Signaling Question Analysis:
    - 5.1 Were the data that produced this result analysed in accordance with a pre-specified analysis plan? → No information The trial report does not provide explicit details on whether a pre-specified analysis plan was finalized before unblinded outcome data became available. This lack of clarity is common in older studies and raises uncertainty regarding potential selective reporting.
    - 5.2 Is the numerical result being assessed likely to have been selected from multiple eligible outcome measurements? → NoThere is no clear evidence that the primary outcome (recurrent common bile duct stones at 1 year) was chosen from among several alternative measurements. The outcome appears to have been consistently defined and reported.
    - 5.3 Is the numerical result being assessed likely to have been selected from multiple eligible analyses of the data? → NoThere is no indication that the study selectively reported one analysis over another for the primary outcome.
- Risk Assessment: SOME CONCERNS
- Reasoning:
    - Both the possibility of multiple outcome measurements (SQ 5.2) and multiple analyses of the data (SQ 5.3) are assessed as not being problematic (answered “No”).

- However, the absence of clear information about whether a pre-specified analysis plan was finalized (SQ 5.1: No information) prevents us from confirming that the analysis was conducted strictly as planned.
- Following the decision rule: when both multiple outcome measurements and analyses are not a concern (i.e., in {N, PN}) but the analysis plan status is unclear (i.e., in {N, PN, NI}), the overall risk of bias should be judged as SOME CONCERNS.

Thus, based on the available evidence and following the RoB 2.0 decision rules, the risk of bias in the selection of the reported result is SOME CONCERNS.

ID: Woodcock 2014

First Response

Domain 1: Bias Arising from the Randomization Process

- Signaling Question Analysis:
  - 1.1 Was the allocation sequence random? → Y The study reports that patients were randomised using a validated computerized system (RandAll) following a telephone call to a central system (RAMOS).
  - 1.2 Was the allocation sequence concealed? → Y Central randomisation via RAMOS ensured that the allocation sequence was securely concealed from study personnel.
  - 1.3 Did baseline differences suggest a problem with randomization? → N Overall, the baseline characteristics and demographics were similar across treatment groups, indicating no substantial imbalances.
- Risk Assessment: LOW risk
- Reasoning:
  - The allocation sequence was explicitly generated by a validated computerized system (Y for randomization).
  - The use of a central system (RAMOS) provided explicit evidence of secure concealment (Y for concealment).
  - Baseline data did not show substantial imbalances overall (N for baseline differences).
  - According to the predefined decision rule: "If allocation sequence concealed in {Y, PY} AND allocation sequence random in {Y, PY, NI} AND baseline imbalances in {N, PN, NI}, return LOW risk."
  - All conditions are met, leading to a final risk assessment of LOW risk for Domain 1.

Domain 2: Bias Due to Deviations from Intended Interventions

- Analysis of ITT vs. PP:
  - The study's primary efficacy analysis was conducted on the ITT population. Exclusions (e.g., removal of data from one problematic centre) were made prior to unblinding and based on quality concerns rather than on post-randomization non-adherence or protocol deviations. Thus, the analysis reflects an ITT approach.
- Signaling Question Analysis (Effect of Assignment to Intervention):
  - 2.1 Were participants aware of their assigned intervention? → No The trial is described as double-blind, ensuring that participants were not aware of their treatment allocation.
  - 2.2 Were carers and people delivering the interventions aware of participants' assigned intervention? → No Double-blinding extends to carers and intervention providers, so they were not aware of assignments.
  - 2.3 Were there deviations from the intended intervention that arose because of the trial context? → No There is no evidence suggesting that deviations arose due to the trial context; any withdrawals or protocol deviations were minimal and predefined.

- 2.4 Were these deviations likely to have affected the outcome?→ No The minimal deviations observed (e.g., withdrawals for AEs or other predefined reasons) were unlikely to influence the primary outcome (asthma exacerbation).
- 2.5 Were these deviations from intended intervention balanced between groups? → Yes The few deviations (e.g., withdrawals) were similar in number and reasons across both treatment groups.
- 2.6 Was an appropriate analysis used to estimate the effect of assignment to intervention? → Yes An ITT analysis was used as the primary analysis, which is the appropriate method to estimate the effect of assignment.
- 2.7 Was there potential for a substantial impact of the failure to analyse participants in the group to which they were randomized? → No Exclusions were minimal and made prior to unblinding, so there is no substantial impact on the results.

- Risk Assessment:
  - Part 1 (Effect of Assignment): With participants and personnel effectively blinded (both answered "No"), there is no concern from deviations arising from the trial context. → LOW risk
  - Part 2 (Appropriate Analysis): The use of ITT analysis (with minimal and justified exclusions) indicates that the analysis appropriately estimated the effect of assignment. → LOW risk
  - Overall Judgment: Since both parts are LOW risk, the overall risk of bias for this domain is LOW risk.
- Reasoning:
  - The study employed a double-blind design with effective blinding of both participants and carers, minimizing the risk of performance bias.
  - There were no notable deviations from the intended intervention attributable to the trial context, and any withdrawals or exclusions were balanced and predefined.
  - The use of an ITT analysis is appropriate for estimating the effect of assignment to intervention, and there is no indication that exclusions substantially impacted the results.
  - Therefore, the final risk assessment for Domain 2 is LOW risk.

Domain 3: Risk of Bias Due to Missing Outcome Data

- Signaling Question Analysis:
  - 3.1 Were data for this outcome available for all, or nearly all, participants randomized? → Probably yes The report indicates that 89% of the ITT population completed the study. Although 89% is lower than the typical ≥95% threshold for continuous outcomes, for a binary outcome such as severe asthma exacerbation this still represents that most participants provided data. However, it is not "all" or "nearly all" by the strictest standard.
  - 3.2 Is there evidence that the result was not biased by missing outcome data? → No information The report does not provide a specific sensitivity analysis or explicit evidence to confirm that the missing outcome data did not bias the results for exacerbations.
  - 3.3 Could missingness in the outcome depend on its true value? → Probably yes At least one withdrawal in the FF 100 μg group was attributed to an exacerbation, suggesting that in some cases, missing data might be related to the outcome value.
  - 3.4 Is it likely that missingness in the outcome depended on its true value? → Probably no Despite the possibility noted in 3.3, the overall number of missing cases is modest and appears balanced across groups, so it is unlikely that the missingness would have substantially depended on the true outcome values.
- Risk Assessment: Following the decision rules:

- Outcome data were not available for "all" participants (3.1 is not a definitive Yes), so we move to the evidence check.
    - With no clear evidence provided that missing data did not bias results (3.2 = No information) and given that missingness could, in principle, depend on the outcome (3.3 = Probably yes), we then examine the likelihood of dependency.
    - Since it is judged that it is probably not the case that missingness strongly depended on the true outcome (3.4 = Probably no), the overall judgment falls into SOME CONCERNS.
- Reasoning:
    - Although most participants (approximately 89%) provided outcome data for severe asthma exacerbation, this falls short of the ideal "nearly all" threshold.
    - The report does not offer direct evidence (e.g., sensitivity analyses) that missing data did not bias the results.
    - While there is some indication that missingness might relate to the outcome (withdrawals due to exacerbation), the overall extent and balance of missing data suggest that any dependency is unlikely to be substantial.
    - Based on these considerations and following the RoB 2.0 decision rules, the overall risk of bias due to missing outcome data is assessed as SOME CONCERNS.

Domain 4: Risk of Bias in Measurement of the Outcome

- Signaling Question Analysis:
    - 4.1 Was the method of measuring the outcome inappropriate?→ No The study used standardized, objective methods (e.g., ATS/ERS criteria for severe asthma exacerbations and clinic-based spirometry for lung function) to measure outcomes, which are appropriate for this context.
    - 4.2 Could measurement or ascertainment of the outcome have differed between intervention groups? → No Measurement procedures were applied uniformly across groups in this double-blind trial.
    - 4.3 Were outcome assessors aware of the intervention received by study participants? → No Outcome assessors were blinded to treatment allocation as the study was double-blind.
    - 4.4 Could assessment of the outcome have been influenced by knowledge of intervention received? → No With blinded assessors and objective measurement methods, influence due to knowledge of intervention is unlikely.
    - 4.5 Is it likely that assessment of the outcome was influenced by knowledge of intervention received? → No Given the robust blinding and standardized methods, it is unlikely that outcome assessment was influenced by treatment assignment.
- Risk Assessment: LOW risk
- Reasoning:
    - The outcome measurement methods were clearly appropriate, using validated and objective criteria.
    - Measurement and ascertainment of the outcome were consistent across groups.
    - Blinding of outcome assessors ensures they were not influenced by treatment assignment.
    - According to the decision rules—since the method was appropriate (4.1 = No), measurement was consistent (4.2 = No), and assessors were blinded (4.3 = No)—the overall risk of bias in the measurement of the outcome is LOW risk.

Domain 5: Risk of Bias in Selection of the Reported Result

- Signaling Question Analysis:

- 5.1 Were the data that produced this result analysed in accordance with a pre-specified analysis plan that was finalized before unblinded outcome data were available for analysis? → Probably yes The methods section indicates that the analyses (including subgroup analyses) were pre-specified (e.g., the use of ANCOVA with predefined covariates and a priori subgroup analyses by randomisation strata and run-in ICS use). Although there is no explicit statement that the final analysis plan was locked before unblinding, the available trial registration and protocol details imply that a pre-specified plan was in place.
- 5.2 Is the numerical result being assessed likely to have been selected, on the basis of the results, from multiple eligible outcome measurements within the outcome domain? → NoFor the outcome of severe asthma exacerbations, the study appears to report a single clearly defined measure (based on the ATS/ERS taskforce definition), with no indication that multiple alternative outcome measurements were available from which one was selectively chosen.
- 5.3 Is the numerical result being assessed likely to have been selected, on the basis of the results, from multiple eligible analyses of the data? → No The analysis of severe asthma exacerbations was conducted in a straightforward manner without evidence of multiple post hoc analytical approaches or selective reporting of different analyses.

- Risk Assessment: LOW risk of bias
- Reasoning:
  - Since both the possibility of multiple outcome measurements (5.2 = No) and multiple analyses of the data (5.3 = No) are ruled out, we refer to the pre-specified analysis plan.
  - The indication that the trial followed a pre-specified plan (5.1 = Probably yes) satisfies the condition for low risk according to the decision rules.
  - Therefore, following the structured decision: "If both multiple outcome measurements in {N, PN} AND multiple analyses of data in {N, PN}, then if the trial was analysed in accordance with a pre-specified plan in {Y, PY} return LOW risk of bias."
  - All criteria are met, resulting in a final judgment of LOW risk of bias for Domain 5.

ID: Woodcock 2014

Second Response

Domain 1: Bias Arising from the Randomization Process

- Signaling Question Analysis:
  - Was the allocation sequence random? → Yes The study explicitly states that patients were “randomised in accordance with a central randomisation schedule generated by the sponsor using a validated computerised system (RandAll)” ensuring that the allocation sequence was random.
  - Was the allocation sequence concealed? → Yes Allocation concealment was maintained by assigning treatment following a telephone call to the Registration and Medication Ordering System (RAMOS), which is indicative of secure concealment.
  - Did baseline differences suggest a problem with randomization? → No Overall, the baseline characteristics (including demographics and lung function) were well balanced between the groups, with no substantial imbalances that would indicate a problem with the randomization process.
- Risk Assessment: Low
- Reasoning:

- The explicit evidence of random allocation (Yes) and secure allocation concealment (Yes) meets the criteria for a robust randomization process.
- The absence of significant baseline imbalances (No) supports the conclusion that the randomization was not compromised.
- According to the predefined RoB 2.0 decision rule—if allocation sequence concealment is rated as Yes (or Probably yes), the allocation sequence is random (rated as Yes/PY/NI), and there are no baseline imbalances (rated as No/PN/NI)—the overall risk for this domain is Low.

Domain 2: Bias Due to Deviations from Intended Interventions

- Signaling Question Analysis:
- Step 1: Analysis Type
  - Analysis approach used: ITT The study clearly states that the ITT population was the primary population for all efficacy analyses, with a per-protocol (PP) population defined only for supportive purposes. Exclusions (e.g., one study center) were made based on data quality concerns rather than for reasons such as non-adherence or toxicity.
- Step 2: Effect of Assignment to Intervention
  - 2.1 Were participants aware of their assigned intervention? → No The study is described as double-blind, indicating that participants were not aware of their treatment allocation.
  - 2.2 Were carers and people delivering the interventions aware? → No Effective blinding of both participants and intervention providers is reported.
  - 2.3 Were there deviations from the intended intervention that arose because of the trial context? → No There is no evidence of deviations arising specifically due to the trial context; any protocol deviations did not indicate a systematic deviation from the intended interventions.
  - 2.4 Were these deviations likely to have affected the outcome?→ No There is no indication that any deviations impacted the primary outcome (asthma exacerbation) or other efficacy endpoints.
  - 2.5 Were these deviations balanced between groups? → YesAny minor deviations (e.g., withdrawals) appear balanced across treatment arms.
  - 2.6 Was an appropriate analysis used to estimate the effect of assignment to intervention? → Yes The use of an ITT analysis (with LOCF for missing data) is appropriate, and exclusions were limited to non-outcome–related data quality issues.
  - 2.7 Was there potential for a substantial impact of failing to analyze participants in the groups to which they were randomized? → No The impact of any exclusions is minimal and unlikely to have substantially affected the results.
- Step 3: Effect of Adhering to Intervention
  - Given the ITT approach, effective blinding, and no evidence of systematic non-adherence or implementation failures, this step does not alter the overall assessment.
- Risk Assessment: Low risk
- Reasoning:
  - The study used a primary ITT analysis with minimal and justified exclusions (limited to data quality concerns rather than deviations such as non-adherence or toxicity).

    - Effective double-blinding ensured that neither participants nor intervention providers were aware of treatment allocation, minimizing the risk that deviations from the intended interventions could be introduced by trial context.
    - There is no evidence of deviations that would have impacted the primary outcome, and any minor deviations (e.g., a few withdrawals) were balanced across groups.
    - The use of an appropriate analytical method (ITT with LOCF) further supports the conclusion that the effect of assignment to intervention was properly estimated.

  Overall, the risk of bias due to deviations from the intended interventions in this study is Low.

Domain 3: Risk of Bias Due to Missing Outcome Data

- Signaling Question Analysis:
    - 3.1 Were data for this outcome available for all, or nearly all, participants randomized? → No Only 89% of the ITT population completed the study, which is below the ideal threshold (e.g., ≥95% for continuous outcomes), indicating that outcome data for exacerbations were not available for nearly all participants.
    - 3.2 Is there evidence that the result was not biased by missing outcome data? → No information The report does not provide explicit analyses or statements confirming that missing data did not bias the results for the exacerbation outcome.
    - 3.3 Could missingness in the outcome depend on its true value? → Probably no There is no clear indication that the likelihood of missing outcome data was related to the true exacerbation status of participants.
    - 3.4 Is it likely that missingness in the outcome depended on its true value? → Probably no While about 11% of participants did not complete the study, there is no strong evidence to suggest that these missing data were systematically related to the true exacerbation outcomes.
- Risk Assessment: Some concerns
- Reasoning:
    - Although a majority (89%) of participants provided outcome data, the proportion falls short of the "nearly all" criterion, which raises concerns about the completeness of the exacerbation data.
    - There is no clear evidence presented that missing data were handled in a manner to definitively rule out bias.
    - Even though the available information suggests that missingness is unlikely to depend on the true outcome (with both 3.3 and 3.4 assessed as "Probably no"), the lack of explicit confirmation that missing data did not bias the results leads to a cautious judgment.
    - Based on the predefined RoB 2.0 rules, the combination of incomplete outcome data and insufficient evidence to negate potential bias results in a Some concerns judgment for this domain.

Domain 4: Risk of Bias in Measurement of the Outcome

- Signaling Question Analysis:
    - 4.1 Was the method of measuring the outcome inappropriate?→ No The outcome (asthma exacerbation) was measured using the ATS/ERS taskforce definition, a well-established and appropriate method.
    - 4.2 Could measurement or ascertainment of the outcome have differed between intervention groups? → No The study was double-blind with standardized criteria applied equally across groups, ensuring consistent measurement of exacerbations.
    - 4.3 Were outcome assessors aware of the intervention received by study participants? → No Due to effective blinding in the double-blind design, outcome assessors were not aware of the participants' treatment allocations.

- 4.4 Could assessment of the outcome have been influenced by knowledge of intervention received? → No Given the blinding and use of objective criteria, it is unlikely that the assessment was influenced by such knowledge.
- 4.5 Is it likely that assessment of the outcome was influenced by knowledge of intervention received? → No There is no evidence to suggest that any influence occurred, as both the measurement methods and blinding protocols were robust.

- Risk Assessment: Low risk
- Reasoning:
  - The use of a validated, standardized definition for asthma exacerbations (ATS/ERS taskforce definition) ensures that the measurement method was appropriate (4.1 = No).
  - Consistency in measurement across intervention groups (4.2 = No) and effective blinding of outcome assessors (4.3 = No) reduce the likelihood of differential measurement.
  - The objective nature of the outcome and absence of any indications that assessment was influenced by knowledge of treatment (4.4 and 4.5 = No) further support the conclusion.
  - Based on the decision rules, with all relevant signaling questions indicating low risk, the overall judgment for this domain is Low risk.

Domain 5: Risk of Bias in Selection of the Reported Result

- Signaling Question Analysis:
  - 5.1 Were the data analyzed in accordance with a pre-specified plan? → Yes The methods state that all efficacy analyses (including those for severe asthma exacerbations) were based on a pre-specified plan. The decision to exclude data from one study center and the definition of the ITT and PP populations were made prior to unblinding, confirming that the analysis plan was finalized before outcome data became available.
  - 5.2 Was the numerical result selected from multiple eligible outcome measurements? → No The outcome of severe asthma exacerbations was defined a priori using the ATS/ERS taskforce definition and reported as a single numerical result. There is no evidence that multiple outcome measures were available or selectively reported.
  - 5.3 Was the numerical result selected from multiple eligible analyses of the data? → No The analysis was conducted using a clearly described ANCOVA model with predetermined covariates, and no evidence suggests that the reported result was chosen from among several analyses.
- Risk Assessment: Low risk
- Reasoning:
  - The trial followed a pre-specified analysis plan (5.1 = Yes), which minimizes the risk of selective analysis.
  - There is no indication that the reported exacerbation outcome was selected from multiple eligible measurements (5.2 = No) or analyses (5.3 = No).
  - Based on the decision rules—since both multiple outcome measurements and multiple analyses are negated, and the analysis was conducted per a pre-specified plan—the overall risk of bias due to selection of the reported result is Low risk.

ID: Şeker 2016

First Response

Domain 1: Bias Arising from the Randomization Process

- Signaling Question Analysis:
  - Was the allocation sequence random? → Y The methods state that patients were “randomly assigned in a 1:1 ratio” to treatment groups.
  - Was the allocation sequence concealed? → NI No explicit information on how allocation concealment was implemented is provided.
  - Did baseline differences suggest a problem with randomization? → N Baseline characteristics were reported to have no significant differences ($p > 0.05$).
- Risk Assessment: SOME CONCERNS
- Reasoning:
  - The allocation sequence was explicitly randomized (Y), which is favorable.
  - However, there is no provided information regarding allocation concealment (NI).
  - Baseline imbalances were not evident (N).
  - Per the RoB 2.0 rules: When allocation concealment is rated as NI and baseline differences show no issues, the decision falls under rule 4.1, resulting in a judgment of SOME CONCERNS.

No inconsistencies were detected, so no corrections were required.

Domain 2: Bias Due to Deviations from Intended Interventions

- Analysis Type:
  - The study did not follow a true intention-to-treat approach. Nine patients randomized to ultrafiltration were excluded due to technical failures (ineffective operation and clotting in the device). This indicates a per-protocol (PP) analysis.
- Signaling Question Analysis (Effect of Adhering to Intervention – Step 3):
  - 2.1 Were participants aware of their assigned intervention during the trial? → Yes There is no evidence of effective blinding; given the nature of the interventions, participants were clearly aware of their treatment assignment.
  - 2.2 Were carers and people delivering the interventions aware of participants' assigned intervention during the trial? → YesThe delivery of ultrafiltration versus diuretic therapy is procedurally distinct, and there is no indication that carers were blinded.
  - 2.3 Were important non-protocol interventions balanced across intervention groups? → Yes Both groups received similar background medical therapy (e.g., continuation of ACE inhibitors, ARBs, beta blockers, and digoxin), suggesting that non-protocol interventions were balanced.
  - 2.4 Were there failures in implementing the intervention that could have affected the outcome? → Yes Technical failures in the ultrafiltration group (termination due to device issues) indicate clear implementation failures.
  - 2.5 Was there non-adherence to the assigned intervention regimen that could have affected participants’ outcomes? → Yes The exclusion of 9 ultrafiltration patients due to technical issues represents significant non-adherence to the intended intervention.
  - 2.6 Was an appropriate analysis used to estimate the effect of adhering to intervention? → No A per-protocol analysis was used that excluded patients for reasons other than missing outcome data, which is inappropriate when deviations (technical failures) are present.
- Risk Assessment: HIGH risk

- Reasoning:
  - Because the study conducted a per-protocol analysis—excluding 9 patients randomized to ultrafiltration due to technical failures—the intended intervention was not fully implemented as planned.
  - Both participants and carers were aware of the assigned interventions, increasing the potential for deviations.
  - Significant failures in implementation (technical problems) and non-adherence were present.
  - Moreover, the analysis did not use an approach that appropriately accounted for these deviations.
  - Per the predefined RoB 2.0 rules (specifically, when failures in implementation and non-adherence are identified and no appropriate analytical adjustment is made), the risk of bias for this domain must be judged as HIGH risk.

Domain 3: Bias Due to Missing Outcome Data

- Signaling Question Analysis:
  - 3.1 Were data for this outcome available for all, or nearly all, participants randomized? → No The study initially randomized approximately 39 patients, but 9 patients in the ultrafiltration group were excluded due to technical failures, leaving only 30 with reported outcomes. This amounts to roughly 23% missing outcome data.
  - 3.2 Is there evidence that the result was not biased by missing outcome data? → No No analyses or sensitivity checks were provided to demonstrate that the missing data did not bias the mortality estimates.
  - 3.3 Could missingness in the outcome depend on its true value? → Probably yes The exclusions were based on technical issues, which may not be entirely independent of patient condition. It is plausible that patients with more severe disease (and potentially higher risk of death) were more likely to experience technical difficulties leading to exclusion.
  - 3.4 Is it likely that missingness in the outcome depended on its true value? → Probably yes Given the potential association between technical failures and patient severity, it is likely that the missing outcome data could be related to the patients' true risk of mortality.
- Risk Assessment: HIGH risk of bias
- Reasoning:
  - Outcome data were not available for all randomized participants (3.1: No).
  - There is no evidence provided that the missing data did not bias the results (3.2: No).
  - The reasons for missing data (technical failures) raise concerns that the missingness could be related to the true outcome (3.3: Probably yes), and it is likely that this dependency exists (3.4: Probably yes).
  - According to the decision rules, when missingness is likely dependent on the true outcome (i.e., answer in {Y, PY, NI} for 3.4) following evidence of substantial missing data, the domain should be judged as HIGH risk of bias.

  No inconsistencies were detected relative to the predefined rules, so no corrections were required.

Domain 4: Risk of Bias in Measurement of the Outcome

- Signaling Question Analysis:
  - 4.1 Was the method of measuring the outcome inappropriate? → No All-cause mortality is measured using objective data (e.g., hospital records, death certificates), which is an appropriate method.
  - 4.2 Could measurement or ascertainment of the outcome have differed between intervention groups? → No There is no indication that the method of recording mortality differed between the ultrafiltration and diuretic groups.

    - 4.3 Were outcome assessors aware of the intervention received by study participants? → Yes The study is open-label and does not report blinding of outcome assessors, so they were likely aware of group assignments.
    - 4.4 Could assessment of the outcome have been influenced by knowledge of intervention received? → No Because all-cause mortality is an objective outcome, the lack of blinding is unlikely to have influenced its measurement.
    - 4.5 Is it likely that assessment of the outcome was influenced by knowledge of intervention received? → No Given the objective nature of mortality data, any influence from assessor awareness is minimal.
- Risk Assessment: LOW risk
- Reasoning:
    - The measurement method for all-cause mortality is appropriate (4.1 = No) and is applied consistently across groups (4.2 = No).
    - Although outcome assessors were aware of the intervention (4.3 = Yes), the objective nature of the outcome means that assessment is unlikely to be biased (4.4 = No; 4.5 = No).
    - According to the RoB 2.0 rules, this leads to a final judgment of LOW risk of bias in the measurement of the outcome.

Domain 5: Risk of Bias in Selection of the Reported Result

- Signaling Question Analysis:
    - 5.1 Were the data that produced this result analysed in accordance with a pre-specified analysis plan? → No information The report does not provide details confirming that a pre-specified analysis plan was finalized before unblinded outcome data became available.
    - 5.2 Is the numerical result being assessed likely to have been selected, on the basis of the results, from multiple eligible outcome measurements? → No There is no clear evidence that the mortality outcome (the primary outcome under assessment) was selectively chosen from several alternative measurements or definitions.
    - 5.3 Is the numerical result being assessed likely to have been selected, on the basis of the results, from multiple eligible analyses of the data? → No The analysis appears straightforward without evidence of multiple analytic approaches being available from which the reported result was selectively chosen.
- Risk Assessment: SOME CONCERNS
- Reasoning:
    - Although there is no evidence of selective reporting from multiple outcome measurements (5.2 = No) or analyses (5.3 = No), the absence of clear information regarding a pre-specified analysis plan (5.1 = No information) prevents full confidence that the results were not selectively reported.
    - According to the decision rules, when the trial is not confirmed to have been analysed in accordance with a pre-specified plan (i.e., the answer is in {N, PN, NI}), the judgment defaults to SOME CONCERNS.
    - No discrepancies or conflicting evidence were identified that would elevate the risk to HIGH, but the lack of pre-specification details warrants caution.

  No corrections were necessary based on the RoB 2.0 decision rules.

ID: Şeker 2016

Second Response

Domain 1: Bias Arising from the Randomization Process

- Signaling Question Analysis:
  - Was the allocation sequence random? → Y The study explicitly states that patients were "randomly assigned" in a 1:1 ratio, indicating a proper randomization process.
  - Was the allocation sequence concealed? → NI No explicit information regarding the concealment of the allocation sequence is provided in the report.
  - Did baseline differences suggest a problem with randomization? → N The baseline characteristics were reported as not significantly different between the groups ($p > 0.05$), indicating no baseline imbalances.
- Risk Assessment: Some Concerns
- Reasoning:
  - The study clearly reports randomization (SQ 1.1 = Y).
  - There is no evidence provided regarding the concealment of the allocation sequence (SQ 1.2 = NI).
  - Baseline differences were not problematic (SQ 1.3 = N).
  - According to the decision rules, if the allocation sequence concealment is rated as NI and baseline imbalances are N (which falls under {N, PN, NI}), then the risk of bias judgment is Some Concerns (Rule 4.1).
  - Therefore, based strictly on the RoB 2.0 decision criteria, the domain is assessed as having Some Concerns.

Domain 2: Bias Due to Deviations from Intended Interventions

- Step 1: Analysis Type Determination
  - Analysis Method: The study excluded 9 patients from the ultrafiltration group after randomization because the ultrafiltration therapy was terminated for technical reasons (e.g., ineffective operation and clotting in the set). These exclusions were not due to missing outcome data but to failures in delivering the intended intervention. → Determined Analysis: Per-protocol (PP)
- Step 3: Effect of Adhering to Intervention (since PP analysis was used)
- Signaling Question Analysis:
  - 2.1 Were participants aware of their assigned intervention? → Yes Given the nature of the interventions (ultrafiltration vs. diuretic therapy), it is clear that participants were aware of the treatment they received.
  - 2.2 Were carers and people delivering the interventions aware of participants' assigned intervention? → Yes Clinicians and intervention providers were aware of the treatment assignments as the interventions are inherently different (e.g., use of a central venous catheter for ultrafiltration versus intravenous diuretics).
  - 2.3 Were important non-protocol interventions balanced across intervention groups? → Yes The study protocol indicates that non-protocol treatments (e.g., continuation of ACE inhibitors, ARBs, beta blockers, and digoxin) were applied equally to both groups.
  - 2.4 Were there failures in implementing the intervention that could have affected the outcome? → Yes The exclusion of 9 patients from the ultrafiltration group due to technical failures (ineffective device operation, clotting) constitutes implementation failures.
  - 2.5 Was there non-adherence to the assigned intervention regimen that could have affected participants' outcomes? → Yes Excluding patients who did not receive the intended ultrafiltration due to technical issues reflects non-adherence to the assigned intervention.
  - 2.6 Was an appropriate analysis used to estimate the effect of adhering to intervention? → No The study employed a per-protocol analysis by excluding these patients. Such exclusions—based on technical failures rather than solely on missing outcome data—are not considered appropriate for estimating the effect of adhering to the intended intervention.

- Risk Assessment:
    - Overall Risk: High Risk
- Reasoning:
    - The study did not use an intention-to-treat approach; instead, it adopted a per-protocol analysis by excluding 9 patients from the ultrafiltration group due to technical failures, indicating deviations from the intended intervention.
    - Both participants and intervention providers were aware of the assigned interventions, which is expected given the nature of the treatments.
    - Although non-protocol interventions were balanced across groups, the technical failures (failures in implementing the intervention) and subsequent non-adherence (exclusions) are critical issues.
    - The use of a per-protocol analysis (scoring "No" for appropriate analysis in 2.6) in the presence of these deviations triggers the decision rule that leads to a High Risk judgment (Rule 2.1.2.2).

  Thus, following the strict RoB 2.0 decision rules for Domain 2, the overall judgment is High Risk for bias due to deviations from intended interventions.

Domain 3: Risk of Bias Due to Missing Outcome Data

- Signaling Question Analysis:
    - 3.1 Were data for this outcome available for all, or nearly all, participants randomized? → N Approximately 9 patients (about 23% of those initially randomized in the ultrafiltration arm) were excluded due to technical failures, meaning outcome data were not available for nearly all randomized participants.
    - 3.2 Is there evidence that the result was not biased by missing outcome data? → N The report does not provide any sensitivity analysis or clear evidence that the missing data did not bias the mortality outcome.
    - 3.3 Could missingness in the outcome depend on its true value? → NI Although the exclusions were due to technical issues (e.g., device malfunction and clotting), the report does not clarify whether these issues might be related to the participants' prognosis or outcomes.
    - 3.4 Is it likely that missingness in the outcome depended on its true value? → NI There is insufficient information to determine if the missing outcome data are systematically related to the true mortality outcomes.
- Risk Assessment: Some Concerns
- Reasoning:
    - A notable proportion of the originally randomized participants (roughly 23%) did not have outcome data (SQ 3.1 = N).
    - There is no evidence presented that missing data did not bias the results (SQ 3.2 = N).
    - The dependency of the missing data on the true outcome remains unclear (SQ 3.3 and SQ 3.4 = NI).
    - According to the RoB 2.0 decision rules, in the absence of clear evidence that missing data are unrelated to the true outcome—and given the substantial proportion of missing data—the default judgment is Some Concerns.

Domain 4: Risk of Bias in Measurement of the Outcome

- Signaling Question Analysis:
    - 4.1 Was the method of measuring the outcome inappropriate? → No All-cause mortality is an objective outcome, typically determined from hospital records or death certificates, which is an appropriate measurement method.

    - 4.2 Could measurement or ascertainment of the outcome have differed between intervention groups? → No The method of outcome ascertainment (i.e., recording deaths) is standard and consistent across both intervention groups.
    - 4.3 Were outcome assessors aware of the intervention received by study participants? → No Given the objective nature of all-cause mortality, any potential awareness of intervention assignment is unlikely to influence the measurement of this outcome.
    - 4.4 Could assessment of the outcome have been influenced by knowledge of intervention received? → No The objective measurement of mortality minimizes the risk of influence from assessors' knowledge of the intervention.
    - 4.5 Is it likely that assessment of the outcome was influenced by knowledge of intervention received? → No There is no reason to suspect that knowledge of the intervention would bias the ascertainment of an objective outcome like mortality.
- Risk Assessment: Low Risk
- Reasoning:
    - The measurement method for all-cause mortality is appropriate and objective (SQ 4.1 = No).
    - There is no evidence suggesting that the method of outcome measurement differed between the groups (SQ 4.2 = No).
    - Outcome assessors are unlikely to have been influenced by knowledge of the intervention, and any potential awareness does not bias the measurement of an objective outcome (SQ 4.3–4.5 = No).
    - Following the RoB 2.0 decision tree, with appropriate measurement methods and no differential ascertainment or influence by assessor awareness, the overall risk of bias in measurement of the outcome is Low Risk.

Domain 5: Risk of Bias in Selection of the Reported Result

- Signaling Question Analysis:
    - 5.1 Were the data that produced this result analysed in accordance with a pre-specified analysis plan? → No information The study report does not provide explicit details or reference to a pre-specified analysis plan that was finalized before the outcome data were unblinded.
    - 5.2 Is the numerical result being assessed likely to have been selected from multiple eligible outcome measurements? → NoFor the primary outcome (all-cause mortality), the report presents a single, clearly defined measurement without evidence of alternative scales, definitions, or time points being selectively reported.
    - 5.3 Is the numerical result being assessed likely to have been selected from multiple eligible analyses of the data? → No The analyses appear to follow the planned statistical tests (e.g., Mann-Whitney U test, Fisher's exact test) without evidence of selective reporting from among multiple analytical approaches.
- Risk Assessment: Some Concerns
- Reasoning:
    - Although there is no indication of selective outcome measurement (SQ 5.2 = No) or selective analysis (SQ 5.3 = No), the absence of clear information about whether a pre-specified analysis plan was finalized before unblinded data became available (SQ 5.1 = No information) raises uncertainty.
    - Following the decision rules, when multiple outcome measurements and multiple analyses are not selectively reported (both in {N, PN}) but the trial is not confirmed to have been analysed in accordance with a pre-specified plan (SQ 5.1 in {NI}), the overall judgment is Some Concerns.

Thus, based on the RoB 2.0 criteria, Domain 5 is judged as having Some Concerns regarding the risk of bias in selection of the reported result.

ID: Lee 2020

First Response

Domain 1: Bias Arising from the Randomization Process

- Signaling Question Analysis:
  - Was the allocation sequence random? → Yes The study explicitly states that a validated computerized system generated the randomization code and that central-based randomization was employed using an interactive response technology system.
  - Was the allocation sequence concealed? → Yes Centralized randomization and the double-blind design imply that the allocation sequence was securely concealed from participants and investigators.
  - Did baseline differences suggest a problem with randomization? → No Baseline characteristics were generally similar across treatment groups, indicating no substantial imbalances.
- Risk Assessment: Low risk
- Reasoning:
  - The allocation sequence was generated by a validated computerized system and implemented via a central interactive response technology system, which provides explicit evidence of randomization and secure concealment.
  - Baseline demographics and clinical characteristics were balanced across groups, indicating no issues with randomization.
  - According to the predefined decision rule: when allocation concealment is confirmed as Yes (or Probably Yes), the randomization is confirmed as Yes (or Probably Yes/No Information), and baseline imbalances are absent (No), the risk is assessed as Low risk.

Domain 2: Bias Due to Deviations from Intended Interventions

- Step 1: Analysis of the Analysis Population
  - ITT/PP Classification: The study explicitly states, “We used the intention-to-treat (ITT) population for all efficacy and safety analyses. The ITT population comprised all patients randomly assigned to treatment, except any who were randomly assigned in error.” → Classification: ITT Exclusions were limited to patients randomly assigned in error, which is acceptable and does not reflect deviations due to non-adherence or post-randomization exclusions for reasons other than missing outcome data.
- Step 2: Effect of Assignment to Intervention
  - 2.1 Were participants aware of their assigned intervention? → NoThe trial was described as double-blind, meaning participants were not aware of their assigned treatment.
  - 2.2 Were carers and people delivering the interventions aware? → No Effective blinding was applied to personnel, so they were not aware of treatment assignments.
  - 2.3 Did deviations from the intended intervention arise because of the trial context? → No There is no indication that trial-specific factors caused deviations; treatment discontinuations occurred as expected in clinical practice and were minimal.

- 2.4 Were these deviations likely to have affected the outcome? → NoHigh treatment compliance (~95%) and balanced drop-outs across groups suggest that any deviations did not affect the outcomes.
- 2.5 Were these deviations balanced between groups? → Yes Drop-outs and other deviations were similar across all treatment arms.
- 2.6 Was an appropriate analysis used to estimate the effect of assignment to intervention? → Yes The use of an ITT approach (with only minimal exclusions for randomization error) is appropriate.
- 2.7 Was there potential for a substantial impact of failure to analyze participants in the group to which they were randomized? → No The exclusions were minimal and unlikely to have influenced the overall results.

→ Part 2 Risk Judgment: LOW risk

- Step 3: Effect of Adhering to Intervention
  - 2.1 Were participants aware of their assigned intervention? → No(Same as above; effective blinding maintained awareness at 'No'.)
  - 2.2 Were carers aware of participants' assigned intervention? → No
  - 2.3 Were important non-protocol interventions balanced across groups? Not applicable since neither participants nor personnel were aware of treatment assignment.
  - 2.4 Were there failures in implementing the intervention that could have affected the outcome? → No High adherence and minimal discontinuations indicate no significant implementation failures.
  - 2.5 Was there non-adherence to the assigned intervention regimen that could have affected outcomes? → No Reported compliance was very high (approximately 95%), with no evidence of impactful non-adherence.
  - 2.6 Was an appropriate analysis used to estimate the effect of adhering to intervention? → Yes The ITT analysis appropriately addresses adherence by including nearly all randomized patients.

→ Part 3 Risk Judgment: LOW risk

- Overall Risk Assessment for Domain 2:
  - Combined Judgment: Since the effect of assignment to intervention (Part 2) is LOW risk and the effect of adhering to intervention (Part 3) is LOW risk, the overall risk for Domain 2 is Low risk.
- Reasoning Summary:
  - Analysis Population: The trial used an ITT approach with minimal, justified exclusions (only patients randomized in error), which is appropriate and minimizes bias.
  - Assignment to Intervention: Double-blinding ensured that neither participants nor personnel were aware of treatment allocation. There were no trial-specific deviations that influenced outcomes, and any drop-outs or discontinuations were minimal and balanced across groups.
  - Adherence: High compliance and proper implementation further support that deviations from the intended interventions did not bias the results.
  - Appropriate Analysis: The use of ITT analysis (with nearly complete follow-up) confirms that both the assignment and adherence aspects were appropriately handled, leading to a LOW risk judgment for Domain 2.

Domain 3: Risk of Bias Due to Missing Outcome Data

- Signaling Question Analysis:
  - 3.1 Were data for this outcome available for all, or nearly all, participants? → Probably yes Outcome data (for exacerbations) were available for 2274 of 2436 participants (93.3%), which—while slightly below a 95% threshold often cited for continuous outcomes—is still high and suggests that missing data are minimal.

- 3.2 Is there evidence that the result was not biased by missing outcome data? → Probably yes The trial reports that any missing data (primarily from early withdrawals) were assumed to be missing at random, and the reasons for missingness do not indicate systematic bias in the estimation of exacerbation outcomes.
- 3.3 Could missingness in the outcome depend on its true value? → Probably no There is no indication that the reasons for missing data (e.g., loss to follow-up) were related to the likelihood of experiencing an exacerbation.
- 3.4 Is it likely that missingness in the outcome depended on its true value? → No Given the high follow-up rate and balanced reasons for missing data across groups, it is unlikely that the missingness was associated with the true exacerbation outcome.

- Risk Assessment: Low risk of bias
- Reasoning:
    - The study provided outcome data for 93.3% of participants, which is considered "nearly all" for the purposes of this assessment.
    - Evidence suggests that any missing data were handled appropriately (assuming missing at random), and there is no clear indication that the missing outcomes were related to the true exacerbation status.
    - Based on the predefined decision rules—since the response to 3.1 is in {Y, PY} and subsequent answers support that missingness is unlikely to bias the result—the overall risk of bias due to missing outcome data is judged as Low risk.

Domain 4: Risk of Bias in Measurement of the Outcome

- Signaling Question Analysis:
    - 4.1 Was the method of measuring the outcome inappropriate?→ No Exacerbations were measured using clearly defined, objective criteria (e.g., requirement for a physician-directed treatment change, hospital admission, or systemic corticosteroid use), which is appropriate for this outcome.
    - 4.2 Could measurement or ascertainment of the outcome have differed between intervention groups? → No The study was double-blind and applied standardized definitions for exacerbations across all treatment arms, ensuring consistent outcome measurement.
    - 4.3 Were outcome assessors aware of the intervention received by study participants? → No Outcome assessors were blinded as part of the double-blind design.
    - 4.4 Could assessment of the outcome have been influenced by knowledge of intervention received? → No Given that both participants and outcome assessors were blinded and the outcome is objectively defined, it is unlikely that the assessment was influenced by treatment knowledge.
    - 4.5 Is it likely that assessment of the outcome was influenced by knowledge of intervention received? → No The effective blinding and objective measurement criteria make it unlikely that the outcome assessment was affected by knowledge of intervention assignment.
- Risk Assessment: Low risk
- Reasoning:
    - The outcome measurement for exacerbations is based on objective, predefined criteria that are appropriate and consistently applied across groups.
    - Double-blinding ensured that both participants and outcome assessors remained unaware of treatment assignments, thereby minimizing any potential influence on outcome measurement.
    - As all signaling questions indicate no concerns regarding measurement bias, the overall risk of bias due to measurement of the outcome is Low risk.

Domain 5: Risk of Bias in Selection of the Reported Result

- Signaling Question Analysis:
  - 5.1 Were the data analyzed in accordance with a pre-specified plan? → Yes The trial was prospectively registered (ClinicalTrials.gov, NCT02924688) and a protocol is available online. The methods describe a pre-specified analysis plan—including a step-down closed-testing hierarchy—and indicate that the analyses were conducted as planned before unblinding the outcome data.
  - 5.2 Was the numerical result likely selected from multiple eligible outcome measurements? → No The outcomes, including the key secondary outcome of asthma exacerbations, were defined a priori with clear, objective criteria, and the report presents both pooled and unpooled analyses without evidence of selective reporting or redefinition to favor certain results.
  - 5.3 Was the numerical result likely selected from multiple eligible analyses of the data? → No All pre-specified analyses appear to have been reported as per the protocol. There is no indication that alternative analytical approaches were selectively reported based on their results.
- Risk Assessment: Low risk of bias
- Reasoning:
  - The trial was conducted according to a pre-specified analysis plan that was finalized prior to unblinding, as evidenced by its trial registration and accessible protocol.
  - There is no indication of selective outcome measurement or selective reporting of analyses; both the primary and key secondary outcomes were reported consistently with the protocol.
  - Based on the decision rules—since both multiple outcome measurements and multiple analyses are judged as "No" (i.e., no evidence of selective reporting) and the analysis was in accordance with a pre-specified plan—the overall risk of bias in the selection of the reported result is Low risk.

ID: Lee 2020

Second Response

Domain 1: Bias Arising from the Randomization Process

- Signaling Question Analysis:
  - Was the allocation sequence random? → Y The study explicitly states that patients were "randomly assigned (1:1:1:1:1:1)" using a central randomisation process with a validated computerised system.
  - Was the allocation sequence concealed? → Y Allocation concealment was achieved via an internal interactive response technology system and matching of treatment devices, ensuring secure concealment.
  - Did baseline differences suggest a problem with randomization? → N Baseline demographics and clinical characteristics were reported as generally similar across treatment groups, indicating no substantial imbalances.
- Risk Assessment: Low risk
- Reasoning: The study provides explicit evidence of a random allocation sequence and secure concealment through central randomisation and an interactive response technology system. In addition, the baseline characteristics are balanced across groups. According to the RoB 2.0 rules—where a "Yes" for both randomisation and concealment along with "No" for baseline imbalances leads to a low risk judgment—this domain is assessed as Low risk. There were no logical inconsistencies detected in this assessment.

Domain 2: Bias Due to Deviations from Intended Interventions

- Step 1: Analysis Approach
  - The study clearly states that all efficacy and safety analyses were conducted on the intention-to-treat (ITT) population. → Analysis classification: ITT
- Step 2: Effect of Assignment to Intervention
  - 2.1 Were participants aware of their assigned intervention during the trial? → Answer: No Rationale: The trial is described as double-blind with patients masked to their treatment assignment.
  - 2.2 Were carers and people delivering the interventions aware of participants' assigned intervention? → Answer: No Rationale: Blinding was extended to investigators (and by inference, carers/intervention providers) via matching of treatment devices and central randomisation.
  - 2.3 Were there deviations from the intended intervention that arose because of the trial context? → Answer: No Rationale: The protocol ensured high treatment compliance (≈95%) and there is no evidence of trial-specific deviations affecting treatment delivery.
  - 2.4 Were these deviations likely to have affected the outcome? → Answer: No Rationale: With high adherence and minimal deviations, any potential impact on the outcome is negligible.
  - 2.5 Were these deviations balanced between groups? → Answer: YesRationale: The reported high and similar compliance rates across treatment arms indicate balanced delivery of interventions.
  - 2.6 Was an appropriate analysis used to estimate the effect of assignment to intervention? → Answer: Yes Rationale: The study used an ITT approach (excluding only patients randomized in error), which is appropriate and avoids bias from post-randomization exclusions.
  - 2.7 Was there potential for a substantial impact of the failure to analyse participants in the group to which they were randomized?→ Answer: No Rationale: Exclusions were minimal (only for those randomized in error) and are unlikely to have substantially affected the results.
- Risk Judgment for Step 2:
  - Part 1 (Awareness/Deviations): Participants and personnel were not aware of the assignment (answers: No) and no meaningful deviations occurred → Low risk.
  - Part 2 (Analysis): An appropriate ITT analysis was conducted and exclusions were minimal → Low risk.
- Step 3: Effect of Adhering to Intervention (Additional Check for PP Analysis)
  - 2.1 Were participants aware of their assigned intervention? → Answer: No
  - 2.2 Were carers aware of the assignment? → Answer: No
  - 2.3 Were important non-protocol interventions balanced? → Answer: Yes
  - 2.4 Were there failures in implementation that could have affected the outcome? → Answer: No
  - 2.5 Was there non-adherence to the assigned regimen that could have affected outcomes? → Answer: No
  - 2.6 Was an appropriate analysis used to estimate the effect of adhering to intervention? → Answer: Yes
- Risk Judgment for Step 3:
  - With effective blinding, balanced non-protocol interventions, minimal implementation failures, and high adherence, the effect of adhering to intervention is also assessed as Low risk.
- Overall Risk Assessment for Domain 2:
  - Final Judgment: Low risk
- Reasoning Summary: The CAPTAIN study employed an ITT analysis with only minimal post-randomization exclusions. Both participants and intervention providers were effectively blinded, ensuring that neither awareness of treatment assignment nor differential deviations occurred. Treatment compliance was high and balanced across groups, and the

appropriate ITT analytical approach was used. All these factors collectively support a Low risk judgment for bias due to deviations from intended interventions.

Domain 3: Risk of Bias Due to Missing Outcome Data

- Signaling Question Analysis:
  - 3.1 Were data for this outcome available for all, or nearly all, participants randomized? → Yes Rationale: Approximately 93% (2274 of 2436) of participants completed the study. For the key secondary outcome (annualised exacerbation rate), the available data are considered nearly complete, making it unlikely that the few missing cases could meaningfully alter the estimated effect.
  - 3.2 Is there evidence that the result was not biased by missing outcome data? → Probably yes Rationale: Although not every detail is provided on sensitivity analyses for missing data, the high follow-up rate and balanced reasons for withdrawal across groups suggest that any missing data are unlikely to have biased the results.
  - 3.3 Could missingness in the outcome depend on its true value? → Probably no Rationale: There is no indication that the reasons for missing data (e.g., withdrawal, loss to follow-up) were related to the severity or frequency of exacerbations. The dropout reasons appear balanced and not outcome-dependent.
  - 3.4 Is it likely that missingness in the outcome depended on its true value? → No Rationale: Given the high completion rate and balanced dropout across treatment arms, it is unlikely that the missing data were systematically related to the true exacerbation outcomes.
- Risk Assessment: Low risk
- Reasoning: The study reports that outcome data were available for nearly all randomized participants, with a completion rate of about 93%. Although this falls slightly below the 95% threshold often cited for continuous outcomes, for the key secondary outcome (annualised exacerbation rate), the minimal proportion of missing data is unlikely to have influenced the overall estimate. Additionally, there is some evidence—through balanced dropout reasons and an ITT approach—that missing data did not bias the results. There is no indication that the missingness was related to the true outcome values. Following the RoB 2.0 decision rules, these findings support a Low risk judgment for bias due to missing outcome data.

Domain 4: Bias in Measurement of the Outcome

- Signaling Question Analysis:
  - 4.1 Was the method of measuring the outcome inappropriate?→ No Rationale: The study used pre-specified, standardized definitions for moderate and severe asthma exacerbations, which are widely accepted and appropriate for assessing the outcome.
  - 4.2 Could measurement or ascertainment of the outcome have differed between intervention groups? → No Rationale: The same objective criteria and measurement procedures were applied across all groups, and the study's double-blind design ensures consistency.
  - 4.3 Were outcome assessors aware of the intervention received by study participants? → No Rationale: The trial was explicitly described as double-blind, meaning outcome assessors were blinded to treatment allocation.
  - 4.4 Could assessment of the outcome have been influenced by knowledge of intervention received? → No Rationale: With effective blinding in place, the likelihood that outcome assessment was influenced by treatment knowledge is very low.

- 4.5 Is it likely that assessment of the outcome was influenced by knowledge of intervention received? → No Rationale: Given that both participants and assessors were blinded and standardized, objective criteria were used, it is unlikely that the outcome assessment was influenced by knowledge of intervention.

- Risk Assessment: Low risk
- Reasoning: The CAPTAIN study utilized validated, objective measures for determining asthma exacerbations and ensured that these methods were applied uniformly across treatment groups. Additionally, the double-blind design guarantees that both participants and outcome assessors remained unaware of treatment assignments, minimizing any potential for bias in measurement. Consequently, there is no indication that the outcome assessment was compromised, and the risk of bias in the measurement of the outcome is judged as Low risk.

Domain 5: Risk of Bias in Selection of the Reported Result

- Signaling Question Analysis:
    - 5.1 Were the data that produced this result analysed in accordance with a pre-specified analysis plan finalized before unblinded data were available? → Yes Rationale: The CAPTAIN trial was prospectively registered (ClinicalTrials.gov, NCT02924688) and the protocol (with pre-specified statistical analysis plans) was published and approved by relevant ethics committees. This indicates that the analysis plan was finalized prior to data unblinding.
    - 5.2 Is the numerical result being assessed likely to have been selected from multiple eligible outcome measurements? → NoRationale: The study clearly defined its key secondary outcome (annualised rate of moderate and/or severe exacerbations) and reported this outcome as per the pre-specified protocol. There is no indication of selective reporting among various outcome measurements within this domain.
    - 5.3 Is the numerical result being assessed likely to have been selected from multiple eligible analyses of the data? → NoRationale: Multiple analyses (e.g., pooled and unpooled) were pre-specified in the analysis hierarchy and reported transparently. There is no evidence of post hoc selective reporting of analyses.
- Risk Assessment: Low risk
- Reasoning: The CAPTAIN study was conducted with a robust pre-specified protocol and analysis plan, as evidenced by trial registration and published protocol details. There is no evidence suggesting that the reported exacerbation results were selectively chosen from multiple measurements or analyses. Since both the outcome measurement and the analysis appear to have been pre-specified and fully reported according to the protocol, the risk of bias in the selection of the reported result is judged as Low risk.

ID: Schreiber 2007

First Response

Domain 1: Bias Arising from the Randomization Process

- Signaling Question Analysis:
    - Was the allocation sequence random? → Yes The study explicitly states that patients were "randomly assigned" and that randomization was "centralized by means of an interactive voice-recognition system."
    - Was the allocation sequence concealed? → Yes The study reports that both patients and investigators were unaware of the group assignment, indicating secure concealment.
    - Did baseline differences suggest a problem with randomization? → No Baseline demographic and disease characteristics were reported as similar between groups.
- Risk Assessment: Low risk

- Reasoning: Following the predefined RoB 2.0 rules:
    - The allocation sequence was random (Yes) and adequately concealed (Yes).
    - Baseline imbalances were not present (No). According to rule 3, if allocation concealment is in {Y, PY}, the allocation sequence is random in {Y, PY, NI}, and baseline imbalances are in {N, PN, NI}, the risk of bias is judged as Low risk. There are no inconsistencies or error corrections needed.

Domain 2: Bias Due to Deviations from Intended Interventions

- Step 1: Evaluation of Analysis Approach (ITT vs. PP)
    - The study clearly states that "all efficacy analyses were performed according to the intention-to-treat principle."
    - Exclusions after randomization were minimal and limited to predefined protocol criteria (e.g., patients without any post-baseline efficacy evaluation). → Analysis Classification: Intention-to-Treat (ITT)
- Step 2: Effect of Assignment to Intervention
    - 2.1 Were participants aware of their assigned intervention during the trial? → No The trial was double-blind; patients were not aware of their treatment allocation.
    - 2.2 Were carers and people delivering the interventions aware of participants' assigned intervention during the trial? → No Blinding extended to investigators and, by implication, the personnel administering the interventions.
    - 2.3 Were there deviations from the intended intervention that arose because of the trial context? → No No trial-specific deviations from the protocol were reported.
    - 2.4 Were these deviations likely to have affected the outcome? → NoThere is no evidence indicating that any deviations (if present) had an impact on the outcome.
    - 2.5 Were these deviations from intended intervention balanced between groups? → Yes Since no notable deviations were identified, balance is assumed across groups.
    - 2.6 Was an appropriate analysis used to estimate the effect of assignment to intervention? → Yes The study employed an ITT approach, which is appropriate for maintaining the benefits of randomization.
    - 2.7 Was there potential for a substantial impact on the result from failure to analyze participants in the group to which they were randomized? → No Exclusions were minimal and driven by predefined criteria, unlikely to substantially affect the outcome.
- Risk Judgment for Step 2:
    - Part 1 (Awareness of Intervention):
        - Participants: No (blinded)
        - Personnel: No (blinded) → Part 1 = LOW risk
    - Part 2 (Appropriate Analysis):
        - Appropriate ITT analysis used → Yes → Part 2 = LOW risk

  Since both Part 1 and Part 2 are LOW risk, the effect of assignment to intervention is judged as LOW risk.
- Step 3: Effect of Adhering to Intervention This step applies only if a per-protocol analysis is used. Since the study conducted an ITT analysis, Step 3 is not applicable.
- Final Risk Assessment for Domain 2: Low risk
- Reasoning Summary:
    - The trial used an ITT analysis with minimal post-randomization exclusions, ensuring that the effect of assignment to intervention is preserved.

- Effective double-blinding of participants and personnel minimized any potential deviations from the intended interventions.
- There is no indication that any deviations arose or were imbalanced, nor that they had any substantial impact on the outcome.

Thus, the overall risk of bias in Domain 2 is judged as Low risk.

Domain 3: Risk of Bias Due to Missing Outcome Data

- Signaling Question Analysis:
  - 3.1 Were data for this outcome available for all, or nearly all, participants randomized? → No Only about 51% of the placebo group and 70% of the certolizumab group completed the 26-week follow-up, meaning a substantial proportion of outcome data is missing.
  - 3.2 Is there evidence that the result was not biased by missing outcome data? → No There is no clear sensitivity analysis or other evidence provided to confirm that the missing data did not bias the outcome estimate; while LOCF was used, this does not fully exclude the possibility of bias.
  - 3.3 Could missingness in the outcome depend on its true value? → Probably yes The reasons for dropout (e.g., lack of improvement, worsening disease) suggest that missingness may be related to the true clinical outcome.
  - 3.4 Is it likely that missingness in the outcome depended on its true value? → Probably yes Given the differential dropout rates (notably higher in the placebo group) and the nature of withdrawals, it is likely that the missing data are related to the true outcomes.
- Risk Assessment: High risk of bias
- Reasoning:
  - Since outcome data were not available for nearly all participants (SQ 3.1: No) and there is no strong evidence to refute the bias from missing data (SQ 3.2: No), we must assess the dependency of missingness on true outcomes.
  - With the possibility that missingness is related to true outcome values (SQ 3.3: Probably yes) and the likelihood that this dependency is substantial (SQ 3.4: Probably yes), the predefined RoB 2.0 decision rule dictates a High risk of bias.

Thus, the overall judgment for Domain 3 is High risk of bias.

Domain 4: Risk of Bias in Measurement of the Outcome

- Signaling Question Analysis:
  - 4.1 Was the method of measuring the outcome inappropriate?→ No The outcome (failure to maintain clinical remission at week 26) was measured using the Crohn’s Disease Activity Index (CDAI), a well-established and validated tool for assessing disease activity in Crohn’s disease.
  - 4.2 Could measurement or ascertainment of the outcome have differed between intervention groups? → No The study was double-blind, and outcome measurements were performed consistently across both groups.
  - 4.3 Were outcome assessors aware of the intervention received by study participants? → No Both patients and investigators (including outcome assessors) were blinded to the treatment allocation.
  - 4.4 Could assessment of the outcome have been influenced by knowledge of intervention received? → No Blinding minimizes the risk that outcome assessment was influenced by treatment knowledge.
  - 4.5 Is it likely that assessment of the outcome was influenced by knowledge of intervention received? → No There is no indication that any such influence occurred.

- Risk Assessment: Low risk
- Reasoning:
  - The measurement method (CDAI) is appropriate and validated.
  - Consistency in outcome ascertainment across groups is supported by the double-blind design.
  - Outcome assessors were blinded, and there is no evidence that their assessments were influenced by treatment allocation.
  - According to the RoB 2.0 decision rules, with answers of "No" across these signaling questions, the risk of bias in measurement of the outcome is judged as Low risk.

Domain 5: Risk of Bias in Selection of the Reported Result

- Signaling Question Analysis:
  - 5.1 Were the data that produced this result analysed in accordance with a pre-specified analysis plan finalized before unblinding? → Yes The study was registered (ClinicalTrials.gov number provided) and the Statistical Analysis section clearly outlines the pre-specified primary and secondary endpoints, along with a closed testing procedure. These elements strongly indicate that the analysis plan was finalized before unblinded outcome data were available.
  - 5.2 Is the numerical result likely to have been selected from multiple eligible outcome measurements within the outcome domain? → No The primary outcome (failure to maintain clinical remission at week 26) and other outcome measures (response and remission based on the CDAI) were clearly defined a priori, and all eligible results were reported as per protocol.
  - 5.3 Is the numerical result likely to have been selected from multiple eligible analyses of the data? → No The analyses were conducted according to the intention-to-treat principle with a detailed statistical analysis plan, and there is no evidence of selective reporting of analyses.
- Risk Assessment: Low risk
- Reasoning:
  - Since both multiple eligible outcome measurements and multiple analyses of the data are answered as No (i.e., no evidence of selective reporting), and the trial was analysed in accordance with a pre-specified plan (Yes for 5.1), the decision rule (1.1) applies: the risk of bias in the selection of the reported result is Low risk.
  - There are no indications of selective outcome measurement or analysis, and all pre-specified endpoints appear to have been fully reported without post hoc selection based on the results.

  Thus, the overall judgment for Domain 5 is Low risk of bias.

ID: Schreiber 2007

Second Response

Domain 1: Bias Arising from the Randomization Process

- Signaling Question Analysis:
  - Was the allocation sequence random? → Y The study explicitly reports that patients were "randomly assigned" with central randomization using an interactive voice-recognition system and an independently generated randomization code.
  - Was the allocation sequence concealed? → Y Patients and investigators were unaware of group assignments, indicating secure concealment of allocation.

- Did baseline differences suggest a problem with randomization? → N Baseline demographic and disease characteristics were reported as similar between groups.

- Risk Assessment: Low
- Reasoning:
  - The allocation sequence was explicitly randomized (“Y”).
  - There is explicit evidence of secure concealment (“Y”).
  - There were no substantial baseline imbalances (“N”). According to the predefined rule—if allocation is concealed in {Y, PY}, the randomization is in {Y, PY, NI}, and baseline imbalances are in {N, PN, NI}—the risk of bias is assessed as Low. No inconsistencies were detected, so no correction was necessary.

Domain 2: Bias Due to Deviations from Intended Interventions

- Step 1: Analysis Type
  - The study explicitly states that “all efficacy analyses were performed according to the intention-to-treat principle.” → Analysis Type: ITT
- Step 2: Effect of Assignment to Intervention
  - 2.1 Were participants aware of their assigned intervention during the trial? → No The study was described as double-blind, and patients were unaware of their group assignment.
  - 2.2 Were carers and people delivering the interventions aware of participants' assigned intervention? → No Investigators and intervention providers were blinded, ensuring that personnel were unaware of group assignment.
  - 2.3 Were there deviations from the intended intervention that arose because of the trial context? → No The report does not indicate any trial-specific deviations from the intended interventions; treatment protocols were followed as planned.
  - 2.4 Were these deviations likely to have affected the outcome? → NoSince no relevant deviations were reported, there is no indication of an impact on the outcome.
  - 2.5 Were these deviations from intended intervention balanced between groups? → Yes Any withdrawals or minor deviations were similar across the intervention groups.
  - 2.6 Was an appropriate analysis used to estimate the effect of assignment to intervention? → Yes The use of an intention-to-treat analysis is appropriate, as it includes all randomized participants (with minimal exclusions) regardless of deviations.
  - 2.7 Was there potential for a substantial impact (on the result) of the failure to analyse participants in the group to which they were randomized? → No Only 3 patients were excluded due to potential unblinding, a minimal number unlikely to affect the overall results.
- Risk Assessment:
  - Part 1 (Effect of Assignment): LOW risk (Participants and personnel were effectively blinded, and there were no meaningful deviations affecting the trial context.)
  - Part 2 (Appropriate Analysis): LOW risk (An ITT analysis was employed, and any exclusions were minimal.)
- Overall Risk Assessment for Domain 2: Low risk
- Reasoning:
  - The study clearly used an ITT approach, including nearly all randomized participants.
  - Both participants and personnel were blinded, minimizing awareness-related biases.

- There is no evidence of deviations from the intended interventions arising due to the trial context, and any minor deviations were balanced between groups.
- The analysis method was appropriate, and the few exclusions noted were unlikely to substantially impact the outcomes.

Thus, based on the predefined criteria, the risk of bias due to deviations from intended interventions is assessed as Low risk.

Domain 3: Risk of Bias Due to Missing Outcome Data

- Signaling Question Analysis:
  - 3.1 Were data for this outcome available for all, or nearly all, participants randomized? → N Only a subset of the randomized participants had outcome data at week 26. For example, in the maintenance phase, only about 69.9% of placebo-treated and 51.4% of certolizumab-treated patients completed the study, meaning missing data are substantial.
  - 3.2 Is there evidence that the result was not biased by missing outcome data? → No There is no clear evidence (e.g., robust sensitivity analyses) confirming that the missing data did not bias the results.
  - 3.3 Could missingness in the outcome depend on its true value? → Yes The reasons for missing data (such as withdrawal due to lack of improvement or adverse events) suggest that missingness is likely related to patients' true outcomes.
  - 3.4 Is it likely that missingness in the outcome depended on its true value? → Yes Given that patients experiencing treatment failure or worsening disease are more likely to drop out, it is likely that the missingness is related to the true outcome values.
- Risk Assessment: High Risk
- Reasoning:
  - Availability of Outcome Data (3.1): With only about 51–70% of patients providing complete outcome data, the proportion of missing data is substantial (answer: N).
  - Evidence Against Bias (3.2): There is no compelling evidence that the missing data did not bias the results (answer: No).
  - Dependency on True Outcome (3.3 & 3.4): The missingness appears likely to be related to the true outcomes, as patients who may have failed to maintain remission or experienced adverse events were more likely to withdraw (answers: Yes for both 3.3 and 3.4).
  - Decision Algorithm:
    - Since outcome data are not nearly complete (3.1 = N) and there is no evidence that the missing data did not bias the results (3.2 = No), we examine the dependency: missingness is likely related to the true outcome (3.3 in {Y, PY}) and it is likely that missingness depended on the true value (3.4 in {Y, PY, NI}).
    - According to the predefined rules, this leads us to a High Risk of bias due to missing outcome data.

No inconsistencies were detected, so no correction is necessary.

Domain 4: Risk of Bias in Measurement of the Outcome

- Signaling Question Analysis:

- 4.1 Was the method of measuring the outcome inappropriate?→ No The outcome (maintenance of clinical remission at week 26) was measured using the Crohn's Disease Activity Index (CDAI), a standard, validated instrument in Crohn's disease trials.
- 4.2 Could measurement or ascertainment of the outcome have differed between intervention groups? → No Both treatment groups were assessed using the same standardized procedures and instruments, ensuring consistency across groups.
- 4.3 Were outcome assessors aware of the intervention received by study participants? → No The study was conducted in a double-blind manner, with outcome assessors blinded to group allocation.
- 4.4 Could assessment of the outcome have been influenced by knowledge of intervention received? → No Blinding of both participants and outcome assessors minimizes the potential for bias in outcome assessment.
- 4.5 Is it likely that assessment of the outcome was influenced by knowledge of intervention received? → No Given the effective blinding and standardized measurement procedures, it is unlikely that outcome assessment was influenced by knowledge of the intervention.

- Risk Assessment: Low risk
- Reasoning:
  - The measurement method (CDAI and related clinical assessments) is appropriate and widely validated.
  - Outcome measurement was conducted in a consistent manner across intervention groups.
  - Outcome assessors were blinded, reducing the risk that knowledge of treatment assignment could influence the results.
  - Overall, the structured evaluation supports a Low risk judgment for bias in measurement of the outcome.

Domain 5: Risk of Bias in Selection of the Reported Result

- Signaling Question Analysis:
  - 5.1 Were the data analyzed in accordance with a pre-specified analysis plan finalized before unblinding? → Probably yes The study report includes detailed statistical methods (e.g., definition of the primary end point, closed test procedure for multiple comparisons, and an intention-to-treat analysis), and the trial registration (ClinicalTrials.gov number, NCT00152425) implies that a protocol and pre-specified analysis plan existed. Although the report does not explicitly state that the analysis plan was finalized before unblinded outcome data were available, the available information strongly suggests this was the case.
  - 5.2 Is the numerical result being assessed likely to have been selected, on the basis of the results, from multiple eligible outcome measurements within the outcome domain? → NoThe primary outcome (failure to maintain clinical remission at week 26) and the corresponding secondary outcomes were pre-specified and reported in full, with no indication that outcomes were selectively chosen post hoc.
  - 5.3 Is the numerical result being assessed likely to have been selected, on the basis of the results, from multiple eligible analyses of the data? → No The report presents the analyses (e.g., intention-to-treat analyses and subgroup analyses) as planned in the protocol, with no evidence of selective reporting of analyses based on the results.
- Risk Assessment: Low risk
- Reasoning:
  - The study appears to have been analyzed in accordance with a pre-specified analysis plan, as indicated by trial registration and detailed statistical methods (5.1 = Probably yes).

- There is no evidence that the numerical results were selectively chosen from among multiple outcome measurements (5.2 = No).
- There is also no evidence that multiple eligible analyses were selectively reported (5.3 = No).
- According to the risk of bias algorithm, with both multiple outcome measurements and multiple analyses assessed as not selectively reported (i.e., in {N, PN}) and the analysis plan judged as Probably yes (in {Y, PY}), the overall risk of bias in the selection of the reported result is Low risk.

No inconsistencies were detected, and the evidence aligns with a Low risk of bias judgment for this domain.

ID: Hinks 2020

First Response

Domain 1: Bias Arising from the Randomization Process

- Signaling Question Analysis:
  - Was the allocation sequence random? → Y The protocol explicitly states that participants will be randomized in a 1:1 ratio, indicating the use of a random allocation process.
  - Was the allocation sequence concealed? → NI No explicit details are provided on the mechanism or security of allocation concealment in the protocol.
  - Did baseline differences suggest a problem with randomization? → NI Baseline data are not reported in the protocol, so no assessment of imbalance is possible.
- Risk Assessment: SOME CONCERNS
- Reasoning:
  - The allocation sequence is explicitly described as random (SQ1.1 = Y).
  - However, there is no information regarding the concealment of the allocation sequence (SQ1.2 = NI).
  - Baseline differences cannot be evaluated because no baseline data are provided (SQ1.3 = NI).
  - According to the predefined decision rules, when allocation concealment is rated as NI and baseline imbalances are NI, the appropriate judgment is SOME CONCERNS (Rule 4.1).

This structured assessment follows the strict RoB 2.0 rules for Domain 1 without any detected inconsistencies.

Domain 2: Bias Due to Deviations from Intended Interventions

- Step 1: Analysis Population Evaluation Based on the protocol, there is no indication that participants were excluded post-randomization for reasons other than potential missing outcome data. In the absence of evidence of systematic exclusions for non-adherence, the planned analysis is assumed to be an intention-to-treat (ITT) approach.
- Step 2: Effect of Assignment to Intervention
  - 2.1 Were participants aware of their assigned intervention during the trial? → Y As an open-label trial, participants clearly knew which intervention they received.
  - 2.2 Were carers and people delivering the interventions aware of participants' assigned intervention during the trial? → Y Given the open-label design, the intervention providers were also aware of treatment assignments.
  - 2.3 Were there deviations from the intended intervention that arose because of the trial context? → N The protocol does not report any deviations arising from the trial context (e.g., non-protocol interventions prompted by awareness of assignment).
  - 2.4 Were these deviations likely to have affected the outcome? → N No evidence suggests that any deviations—if present—would have impacted the primary outcome (all-cause mortality at day 28).

    - 2.5 Were these deviations from intended intervention balanced between groups? → Y There is no indication of any imbalance in deviations between the study arms.
    - 2.6 Was an appropriate analysis used to estimate the effect of assignment to intervention? → Y The trial is planned to use an ITT analysis (or a closely related modified ITT that excludes only those with missing outcome data), which is considered appropriate.
    - 2.7 Was there potential for a substantial impact of the failure to analyse participants in the group to which they were randomized? → N There is no indication that any post-randomization exclusions would have substantially impacted the results.
- Risk Assessment: LOW risk
- Reasoning:
    - Although the open-label design means that both participants and intervention providers were aware of the treatment assignment (SQ 2.1 and 2.2 = Y), there is no evidence that this awareness led to deviations from the intended interventions (SQ 2.3 = N).
    - Furthermore, any potential deviations are unlikely to affect the outcome (SQ 2.4 = N) and appear to be balanced between groups (SQ 2.5 = Y).
    - The planned analysis is ITT, which is appropriate for estimating the effect of assignment to intervention (SQ 2.6 = Y) and there is no substantial impact from any failure to analyze participants in the groups to which they were randomized (SQ 2.7 = N).
    - According to the provided decision rules—with Part 1 (effect of assignment) and Part 2 (analysis approach) both rated as LOW risk—the overall risk of bias in Domain 2 is determined to be LOW risk.

Domain 3: Risk of Bias Due to Missing Outcome Data

- Signaling Question Analysis:
    - 3.1 Were data for this outcome available for all, or nearly all, participants randomized? → No information The protocol does not provide explicit details on the proportion of participants with available outcome data for all-cause mortality at day 28.
    - 3.2 Is there evidence that the result was not biased by missing outcome data? → No information No evidence or analysis is provided in the protocol regarding bias correction for missing data.
    - 3.3 Could missingness in the outcome depend on its true value? → No Given that the primary outcome is all-cause mortality—which is typically obtained via reliable sources such as death registers—the mechanism of missingness is unlikely to be related to the true outcome value.
    - 3.4 Is it likely that missingness in the outcome depended on its true value? → No For similar reasons as above, it is unlikely that any missing data (if present) would depend on the true mortality status.
- Risk Assessment: SOME CONCERNS
- Reasoning:
    - The protocol does not specify the extent of missing outcome data (SQ 3.1 = No information) nor provide evidence that any missing data did not bias the results (SQ 3.2 = No information).
    - Although the outcome of all-cause mortality is inherently objective—with SQ 3.3 and SQ 3.4 suggesting that missingness is unlikely to depend on the true outcome—the lack of explicit reporting on the completeness of outcome data and any planned bias correction necessitates caution.
    - Following the decision rules: since the available information for SQ 3.1 and SQ 3.2 is classified as No information, the default judgment is SOME CONCERNS.

This conclusion adheres to the strict RoB 2.0 decision rules, ensuring that uncertainty regarding missing outcome data is appropriately flagged.

Domain 4: Risk of Bias in Measurement of the Outcome

- Signaling Question Analysis:
  - 4.1 Was the method of measuring the outcome inappropriate? → No All-cause mortality is measured via objective sources (e.g., electronic patient records, death registers, telephone follow-up), which is appropriate for this outcome.
  - 4.2 Could measurement or ascertainment of the outcome have differed between intervention groups? → No The ascertainment of all-cause mortality is standardized and would be expected to be consistent across intervention groups.
  - 4.3 Were outcome assessors aware of the intervention received by study participants? → Yes Given the open-label design, outcome assessors are likely aware of the intervention assignments.
  - 4.4 Could assessment of the outcome have been influenced by knowledge of intervention received? → No Because all-cause mortality is an objective outcome, awareness of the intervention is unlikely to bias its measurement.
  - 4.5 Is it likely that assessment of the outcome was influenced by knowledge of intervention received? → No There is little reason to suspect that the assessment of mortality would be affected by such knowledge.
- Risk Assessment: LOW risk
- Reasoning:
  - The measurement method for the outcome is appropriate (SQ 4.1 = No), meaning it does not introduce bias by itself.
  - Measurement is consistent across groups (SQ 4.2 = No), so no differential measurement exists between intervention arms.
  - Although outcome assessors are aware of the intervention (SQ 4.3 = Yes), the objective nature of the outcome (all-cause mortality) ensures that this awareness does not influence the measurement (SQ 4.4 and 4.5 = No).
  - Following the decision rules:
    - Since the method of measurement is appropriate and consistent (4.1 = No; 4.2 = No), we proceed to check assessor awareness.
    - With outcome assessors being aware (4.3 = Yes) but no indication that such awareness influenced the assessment (4.4 = No), we classify the potential influence as falling within the {N, PN} category.
    - Per the rules, this leads to a LOW risk of bias in measurement of the outcome.

  Thus, based on the structured assessment and strict application of the RoB 2.0 rules, Domain 4 is judged to be at LOW risk of bias.

Domain 5: Risk of Bias in Selection of the Reported Result

- Signaling Question Analysis:
  - 5.1 Were the data that produced this result analysed in accordance with a pre-specified analysis plan that was finalized before unblinded outcome data were available for analysis? → Y The trial protocol is publicly registered (e.g., ClinicalTrials.gov NCT04381962) and specifies the analysis plan before data collection, supporting that a pre-specified plan was in place.

- 5.2 Is the numerical result being assessed likely to have been selected, on the basis of the results, from multiple eligible outcome measurements within the outcome domain? → N The outcome (all-cause mortality at day 28) is clearly defined and singularly reported, with no evidence of multiple measurements or selective choice among scales or time points.
- 5.3 Is the numerical result being assessed likely to have been selected, on the basis of the results, from multiple eligible analyses of the data? → N The protocol does not indicate that multiple alternative analyses were conducted or selectively reported; the analysis appears to follow the pre-specified plan.

- Risk Assessment: LOW risk
  - Reasoning:
  - There is clear evidence that the trial was analyzed in accordance with a pre-specified analysis plan (SQ 5.1 = Y).
  - There is no indication of selective reporting based on multiple outcome measurements (SQ 5.2 = N) or multiple analyses (SQ 5.3 = N).
  - According to the decision rules, when both potential issues in outcome measurement and analysis selection are not present, and the pre-specified plan was followed, the risk of bias in the selection of the reported result is LOW risk.

ID: Hinks 2020

Second Response

Domain 1: Bias Arising from the Randomization Process

- Signaling Question Analysis:
  - Was the allocation sequence random? → Y The protocol explicitly states that participants will be randomised in a 1:1 ratio, indicating a random allocation process.
  - Was the allocation sequence concealed? → NI The protocol does not provide explicit details regarding the method of allocation concealment.
  - Did baseline differences suggest a problem with randomization? → NI Baseline data are not reported in the protocol.
- Risk Assessment: Some Concerns
- Reasoning:
  - The study confirms randomization (SQ1.1: Y). However, no information is provided on allocation concealment (SQ1.2: NI), and baseline differences are not reported (SQ1.3: NI).
  - According to the decision rules, when allocation sequence concealment is NI and baseline imbalances are in {N, PN, NI}, the risk judgment should be "Some Concerns."
  - No inconsistencies were detected; therefore, no corrections are needed.

Domain 2: Bias Due to Deviations from Intended Interventions

- Signaling Question Analysis:

  Step 1: Evaluation of Analysis Population (ITT vs. PP)
  - Analysis approach (e.g., ITT, mITT, or PP): NI There is no explicit information provided in the protocol regarding whether an intention-to-treat (or modified ITT) versus per-protocol analysis will be used. In the absence of such details, the analysis approach is classified as "No information."
- Step 2: Effect of Assignment to Intervention

- 2.1 Were participants aware of their assigned intervention? → Yes As this is an open-label trial, participants clearly know which intervention they receive.
- 2.2 Were carers and people delivering the interventions aware of participants' assigned intervention? → Yes Given the open-label design, intervention providers are also aware of assignments.
- 2.3 Did deviations from the intended intervention arise because of the trial context? → NI The protocol does not provide information on whether any trial-context–driven deviations occurred.
- 2.4 Were these deviations likely to have affected the outcome? → NI No details are provided regarding the impact of any such deviations on the primary outcome.
- 2.5 Were these deviations balanced between groups? → NI There is no information on whether any deviations (if present) were balanced across intervention groups.
- 2.6 Was an appropriate analysis used to estimate the effect of assignment to intervention? → NI The protocol does not specify the analysis method for the effect of assignment (e.g. ITT vs. per-protocol), so we lack clarity on whether exclusions were justified or not.
- 2.7 Was there potential for a substantial impact of failure to analyze participants in the groups to which they were randomized? → NI Without details on the analysis approach and extent of exclusions, this remains unclear.

- Overall for the effect of assignment to intervention (Steps 2.1–2.7), the decision rules lead us to classify this part as having Some Concerns.
  Step 3: Effect of Adhering to Intervention
  - 2.1 Were participants aware of their assigned intervention? → Yes Participants are aware due to the open-label design.
  - 2.2 Were carers and people delivering the interventions aware of participants' assigned intervention? → Yes Carers/intervention providers are likewise aware.
  - 2.3 Were important non-protocol interventions balanced across intervention groups? → NI The protocol does not provide details on non-protocol interventions or their balance.
  - 2.4 Were there failures in implementing the intervention that could have affected the outcome? → NI There is no explicit mention of implementation failures.
  - 2.5 Was there non-adherence to the assigned intervention regimen that could have affected outcomes? → NI No information is provided regarding non-adherence.
  - 2.6 Was an appropriate analysis used to estimate the effect of adhering to intervention? → NI The analysis method for assessing adherence effects is not described.
- Following the decision rules for the effect of adhering to intervention, the branch where non-protocol interventions are "No information" and the appropriate analysis is not specified leads to a judgment of High Risk for this component.
- Risk Assessment: High Risk
- Reasoning:
  - The open-label design means that both participants and intervention providers are aware of treatment assignments, which inherently increases the potential for deviations.
  - Although the effect of assignment to intervention (Step 2) was judged as "Some Concerns" due to insufficient details on deviations and the analysis method, the evaluation of the effect of adhering to the intervention (Step 3) reveals a lack of information on key factors such as balance of non-protocol interventions, implementation failures, and the analytic approach for adherence.

- According to the predefined rules, when the effect of adhering component indicates High Risk—even in the context of Some Concerns in the effect of assignment—the overall risk of bias for Domain 2 must be classified as High Risk.
- No logical inconsistencies were detected during the application of the decision tree.

Overall, based on the available protocol information and following the strict RoB 2.0 rules, Domain 2 is judged to be at High Risk of bias due to deviations from intended interventions.

Domain 3: Risk of Bias Due to Missing Outcome Data

- Signaling Question Analysis:
  - 3.1 Were data for this outcome available for all, or nearly all, participants randomized? → Y The protocol specifies that outcome data (vital status via ePR, death register, and telephone calls at day 28) will be obtained from multiple sources, which is expected to capture nearly all events.
  - 3.2 Is there evidence that the result was not biased by missing outcome data? → Y Given that all-cause mortality is an objective outcome and the design uses reliable data sources, there is clear evidence that missing data are unlikely to have biased the result.
  - 3.3 Could missingness in the outcome depend on its true value? → N Because death is objectively recorded and follow-up is comprehensive, missingness is unlikely to be related to the true outcome values.
  - 3.4 Is it likely that missingness in the outcome depended on its true value? → N The methods planned for outcome ascertainment suggest that any missing data would be minimal and not dependent on whether a participant died or survived.
- Risk Assessment: Low Risk
- Reasoning:
  - The trial protocol is designed to ensure near-complete ascertainment of all-cause mortality through multiple robust data sources (ePR, death registers, and telephone follow-up), thereby minimizing the proportion of missing outcome data (SQ 3.1: Y).
  - There is clear evidence that the objective nature of the outcome and the planned methods prevent bias due to missing data (SQ 3.2: Y).
  - Given that missingness is unlikely to be related to the actual outcome (SQ 3.3 and 3.4: N), the decision rule dictates that when outcome data are available for all or nearly all participants, the risk of bias due to missing outcome data is judged as Low Risk.
  - No further adjustments or corrections are needed based on the available information.

Domain 4: Risk of Bias in Measurement of the Outcome

- Signaling Question Analysis:
  - 4.1 Was the method of measuring the outcome inappropriate? → No The primary outcome, all-cause mortality, is measured using objective data sources (electronic patient records, death registers, and telephone follow-up), which is an appropriate method.
  - 4.2 Could measurement or ascertainment of the outcome have differed between intervention groups? → No The outcome measurement procedures are applied uniformly across all participants, with no indication of differential ascertainment between groups.

    - 4.3 Were outcome assessors aware of the intervention received by study participants? → Yes Due to the open-label design, outcome assessors are likely aware of the intervention; however, this is less critical for an objective outcome such as mortality.
    - 4.4 Could assessment of the outcome have been influenced by knowledge of intervention received? → No Despite the assessors' awareness, the objective nature of all-cause mortality minimizes the potential for such knowledge to influence the outcome assessment.
    - 4.5 Is it likely that assessment of the outcome was influenced by knowledge of intervention received? → No Given the objective and verifiable nature of mortality data, it is unlikely that the outcome assessment was influenced by intervention awareness.
- Risk Assessment: Low Risk
- Reasoning:
    - The measurement method is appropriate and uniformly applied across intervention groups (SQ 4.1: No; SQ 4.2: No).
    - Although outcome assessors are aware of the intervention (SQ 4.3: Yes), the objective nature of the primary outcome (all-cause mortality) means that this awareness is unlikely to influence the measurement (SQ 4.4 and SQ 4.5: No).
    - Following the decision tree, when measurement is appropriate, consistently applied, and unlikely to be influenced by knowledge of intervention despite assessor awareness, the overall risk of bias in outcome measurement is Low Risk.

Domain 5: Risk of Bias in Selection of the Reported Result

- Signaling Question Analysis:
    - 5.1 Were the data analyzed in accordance with a pre-specified plan finalized before unblinded data were available? → PY The trial is registered (ClinicalTrials.gov NCT04381962, EudraCT 2020-001740-26) and the protocol indicates pre-specified objectives and outcomes, which implies that an analysis plan was in place. However, explicit confirmation that the analysis plan was finalized before unblinded outcome data were available is not detailed, leading to a "Probably yes" response.
    - 5.2 Is the numerical result likely to have been selected from multiple eligible outcome measurements? → N The primary outcome (all-cause mortality at day 28) is objectively defined and uniquely specified, with no indication that multiple measurements or definitions were available or selectively reported.
    - 5.3 Is the numerical result likely to have been selected from multiple eligible analyses of the data? → N There is no evidence in the protocol suggesting that selective analysis choices were made, and the analysis appears to follow a pre-specified approach.
- Risk Assessment: Low Risk
- Reasoning:
    - Both the potential for selective reporting of multiple outcome measurements (5.2) and multiple analyses (5.3) are judged as "No," indicating that there is no indication of selective reporting in these domains.
    - The analysis appears to follow a pre-specified plan (5.1: PY), as inferred from the trial registration and protocol details, despite the absence of explicit confirmation regarding the finalization of the analysis plan before unblinding.
    - According to the decision rules, when both 5.2 and 5.3 are in {N, PN} and the trial is analyzed in accordance with a pre-specified plan in {Y, PY} (here, PY), the overall risk of bias for Domain 5 is Low Risk.

ID: Cotton 2014

FIrst Response

Domain 1: Bias Arising from the Randomization Process

- Signaling Question Analysis:
  - Was the allocation sequence random? → Yes A centralized web-based system using a permuted block scheme with random block sizes was explicitly reported.
  - Was the allocation sequence concealed? → Yes Centralized randomization implies secure concealment of the allocation sequence.
  - Did baseline differences suggest a problem with randomization? → No Baseline demographic and clinical characteristics were well balanced between groups.
- Risk Assessment: LOW risk
- Reasoning:
  - The study explicitly reported a randomized allocation using a centralized web-based system with a permuted block scheme (SQ 1.1: Yes).
  - This centralized approach ensures proper concealment of the allocation sequence (SQ 1.2: Yes).
  - Moreover, there were no substantial baseline imbalances between treatment groups (SQ 1.3: No).
  - According to the RoB 2.0 decision rules: if the allocation sequence is concealed (Yes), the allocation is random (Yes), and baseline imbalances are absent (No), then the risk of bias in this domain is classified as LOW risk.

Domain 2: Bias Due to Deviations from Intended Interventions

- Step 1: Analysis Approach
  - Analysis Method: The study was analyzed using an intention-to-treat (ITT) approach. Although a sensitivity analysis using a completer (modified ITT) population was performed, the primary analysis included all randomized patients. This indicates that any exclusions were largely due to missing outcome data rather than non-adherence or protocol violations.
- Step 2: Effect of Assignment to Intervention
  - SQ2.1: Were participants aware of their assigned intervention during the trial? → No The study explicitly states that patients were blinded to their treatment allocation.
  - SQ2.2: Were carers and people delivering the interventions aware of participants' assigned intervention during the trial? → No Blinding extended to the referring physicians and research staff, ensuring that carers were not aware of the assignments.
  - SQ2.3: Were there deviations from the intended intervention that arose because of the trial context? → No There is no evidence of deviations arising from trial-specific factors; the interventions (sphincterotomy or sham) were implemented as intended.
  - SQ2.4: Were these deviations likely to have affected the outcome? → No Given the lack of deviations, there is no indication that any such deviations would impact the primary outcome.
  - SQ2.5: Were these deviations from intended intervention balanced between groups? → Yes Any potential deviations (e.g., re-interventions due to persistent pain) were protocol-defined and occurred similarly across groups.

- SQ2.6: Was an appropriate analysis used to estimate the effect of assignment to intervention? → Yes The use of an ITT analysis, including all randomized patients, is appropriate. Exclusions were primarily due to missing outcome data and were handled by imputation as per the study protocol.
- SQ2.7: Was there potential for a substantial impact of the failure to analyze participants in the group to which they were randomized? → No The ITT analysis minimizes the risk that any post-randomization exclusions would substantially impact the results.

- Risk Assessment: LOW risk
- Reasoning:
  - The study's use of an ITT approach confirms that the analysis was based on the assigned treatment groups regardless of any deviations, with exclusions mainly due to missing outcome data.
  - Effective blinding ensured that neither participants nor carers were aware of the intervention assignment, minimizing the risk of deviations affecting behavior or implementation.
  - There is no evidence of trial context–specific deviations that could have biased the outcome, and any deviations (if present) were balanced across groups.
  - Given that the appropriate analysis method was employed and no substantial impact from exclusions was detected, both the effect of assignment and adherence components are evaluated as LOW risk.
  - According to the decision rules provided, with both components at LOW risk, the overall risk of bias in Domain 2 is LOW risk.

Domain 3: Risk of Bias Due to Missing Outcome Data

- Signaling Question Analysis:
  - 3.1 Were data for this outcome available for all, or nearly all, participants randomized? → No Approximately 11% (23/214) of participants had missing or late RAPID scores, so outcome data were available for less than the ≥95% typically considered "all or nearly all."
  - 3.2 Is there evidence that the result was not biased by missing outcome data? → Yes A secondary sensitivity (complete-case) analysis of 173 participants yielded similar results to the primary analysis with imputed data, suggesting that the missing data did not bias the overall effect estimate.
  - 3.3 Could missingness in the outcome depend on its true value? → No The reasons for missing data (late or missing RAPID scores) appear to be similar across groups, with no clear indication that the missingness was related to the true outcome values.
  - 3.4 Is it likely that missingness in the outcome depended on its true value? → No Given the balanced distribution of missing data and the supportive sensitivity analysis, it is unlikely that missingness was dependent on the true outcome values.
- Risk Assessment: LOW risk
- Reasoning:
  - Although outcome data were not available for nearly all participants (SQ3.1: No), the sensitivity analysis (SQ3.2: Yes) demonstrated that the imputation of missing data did not alter the effect estimate.
  - There is no evidence to suggest that missing data were related to the true outcomes (SQ3.3 and SQ3.4: No).
  - According to the decision rules, when evidence indicates that missing data did not bias the result (SQ3.2 = Yes), the overall risk of bias in this domain is assessed as LOW risk.

Domain 4: Risk of Bias in Measurement of the Outcome

- Signaling Question Analysis:
  - 4.1 Was the method of measuring the outcome inappropriate? → No The primary outcome was measured using the RAPID instrument—a tool developed and validated specifically for this study—which is an appropriate method of assessment.
  - 4.2 Could measurement or ascertainment of the outcome have differed between intervention groups? → No Outcome data were collected using standardized procedures (blinded research coordinators and central outcome collection) applied uniformly to all participants.
  - 4.3 Were outcome assessors aware of the intervention received by study participants? → No The study explicitly reports that outcome assessors were blinded to treatment allocation.
  - 4.4 Could assessment of the outcome have been influenced by knowledge of intervention received? → No Blinding of both participants and outcome assessors minimizes any risk of bias in the assessment.
  - 4.5 Is it likely that assessment of the outcome was influenced by knowledge of intervention received? → No Given the effective blinding and standardized outcome measurement procedures, it is unlikely that knowledge of intervention would have influenced the outcome assessment.
- Risk Assessment: LOW risk
- Reasoning:
  - The measurement method (RAPID instrument) is validated and appropriate (SQ 4.1: No).
  - Outcome ascertainment was conducted consistently across intervention groups (SQ 4.2: No).
  - Outcome assessors were blinded, eliminating potential bias from awareness of treatment allocation (SQ 4.3: No).
  - There is no indication that the assessment could have been or was influenced by knowledge of intervention received (SQ 4.4 and SQ 4.5: No).
  - Following the decision rules, when the measurement method is appropriate, measurement is consistent across groups, and outcome assessors are blinded with no influence on the assessment, the risk of bias in Domain 4 is classified as LOW risk.

Domain 5: Risk of Bias in Selection of the Reported Result

- Signaling Question Analysis:
  - 5.1 Were the data that produced this result analysed in accordance with a pre-specified analysis plan finalized before unblinded outcome data were available? → Yes The EPISOD trial was registered (clinicaltrials.gov Identifier: 00688662) and the study protocol and analysis plan were established in advance. The methods section clearly describes a pre-specified primary analysis (using an ITT approach with logistic regression) and a pre-planned sensitivity (completer) analysis.
  - 5.2 Is the numerical result being assessed likely to have been selected, on the basis of the results, from multiple eligible outcome measurements within the outcome domain? → No The primary outcome was defined a priori using the RAPID score with specific criteria for treatment success. There is no evidence of selective measurement or alternative definitions being reported post hoc.
  - 5.3 Is the numerical result being assessed likely to have been selected, on the basis of the results, from multiple eligible analyses of the data? → No The analyses reported (both adjusted and unadjusted risk differences, along with sensitivity analyses) are consistent with the pre-specified analysis plan. There is no indication that the results were selectively chosen from a range of post hoc analyses.
- Risk Assessment: LOW risk

- Reasoning:
  - The trial adhered to a pre-specified analysis plan finalized before the outcome data were unblinded (SQ 5.1: Yes).
  - There is no evidence of selective measurement of outcomes or selective reporting among multiple eligible outcome measurements (SQ 5.2: No).
  - All analyses appear to be in line with the pre-specified protocol, with no indication that the numerical result was chosen based on the results from among multiple alternative analyses (SQ 5.3: No).
  - Based on the decision rules, when both multiple outcome measurements and multiple analyses are not a concern (i.e., both assessed as No) and the trial was analyzed in accordance with a pre-specified plan (Yes), the overall risk of bias in the selection of the reported result is classified as LOW risk.

ID: Cotton 2014

Second Response

Domain 1: Bias Arising from the Randomization Process

- Signaling Question Analysis:
  - Was the allocation sequence random? → Yes The study explicitly states that a permuted block scheme with random block sizes was used for randomization.
  - Was the allocation sequence concealed? → Yes Allocation was performed using a centralized web-based system, ensuring secure concealment.
  - Did baseline differences suggest a problem with randomization? → No Baseline characteristics were well balanced between the treatment groups.
- Risk Assessment: Low
- Reasoning: The study used a permuted block randomization method with random block sizes and a centralized web-based system, which guarantees both randomization and proper allocation concealment. Additionally, the baseline data confirmed that the groups were comparable with no substantial imbalances. According to the decision rules (allocation concealed = Yes, allocation random = Yes, and baseline differences = No), the domain is rated as Low Risk.

Domain 2: Bias Due to Deviations from Intended Interventions

- Analysis Type:
  - The study clearly conducted an intention-to-treat (ITT) analysis (“all analyses were conducted with the intention-to-treat population defined as all randomized patients”), with only missing outcomes (handled by imputation) leading to exclusions. These exclusions relate solely to missing data rather than post-randomization non-adherence, supporting an ITT approach.
- Effect of Assignment to Intervention (Step 2):
  - 2.1 Were participants aware of their assigned intervention during the trial? → No Patients were blinded to their treatment allocation.
  - 2.2 Were carers and people delivering the interventions aware of participants' assigned intervention during the trial? → Yes The endoscopists performing the procedures necessarily knew which intervention (sphincterotomy vs sham) was delivered, as is typical in such trials.

- 2.3 Were there deviations from the intended intervention that arose because of the trial context? → No There is no indication that the knowledge of allocation by the interventionists led to deviations from the protocol; both groups received procedures designed to mimic each other closely.
- 2.4 Were these deviations likely to have affected the outcome? → No The sham procedure was carefully designed to mimic the active intervention, and any potential deviations are unlikely to have influenced the objectively measured primary outcome (pain-related disability).
- 2.5 Were these deviations from intended intervention balanced between groups? → Yes Both groups underwent similar procedural components (e.g., cannulation, manometry, temporary pancreatic stenting) ensuring balance.
- 2.6 Was an appropriate analysis used to estimate the effect of assignment to intervention? → Yes The ITT analysis with appropriate imputation for missing data was employed.
- 2.7 Was there potential for a substantial impact of the failure to analyze participants in the group to which they were randomized? → No Any exclusions (due to missing outcome data) were minimal and handled by the prespecified imputation plan.
- Risk Assessment for Step 2: Given that (a) despite personnel being aware (2.2: Yes), there were no deviations attributable to this awareness (2.3: No) and any procedural aspects were balanced (2.5: Yes), and (b) an appropriate ITT analysis was used (2.6: Yes) with negligible impact from exclusions (2.7: No), the effect of assignment to intervention is rated as LOW risk.

- Effect of Adhering to Intervention (Step 3):
  - Not applicable, as the analysis was conducted under ITT principles.
- Overall Risk Assessment for Domain 2: Low
- Reasoning: The trial employed a robust ITT approach, minimizing bias from post-randomization exclusions by handling missing outcomes appropriately. Although the interventionists were necessarily unblinded, this did not translate into deviations from the intended interventions. Both groups received highly comparable procedures (including sham components) and any non-protocol aspects were balanced. Consequently, there is little likelihood that deviations from the intended interventions affected the outcome, resulting in a Low Risk judgment for Domain 2.

Domain 3: Risk of Bias Due to Missing Outcome Data

- Signaling Question Analysis:
  - 3.1 Were data for this outcome available for all, or nearly all, participants randomized? → No Approximately 23 of 214 participants (about 10.7%) had missing or late RAPID scores, which does not meet the criterion of "nearly all" (e.g., ≥95% for continuous outcomes).
  - 3.2 Is there evidence that the result was not biased by missing outcome data? → Yes Sensitivity analyses were performed, and the results remained consistent when comparing extreme assumptions for the missing data, indicating that the missing data did not bias the primary outcome estimate.
  - 3.3 Could missingness in the outcome depend on its true value? → No There is no evidence suggesting that the reasons for missing data were related to the true outcomes.
  - 3.4 Is it likely that missingness in the outcome depended on its true value? → No Balanced reasons for missing data across groups and the sensitivity analyses support that missingness is unlikely to be related to the true outcome values.
- Risk Assessment: Low Risk
- Reasoning: Although only about 89% of the randomized participants had complete outcome data, the sensitivity analyses clearly demonstrated that the imputation of missing outcomes did not alter the estimated effect. Additionally, there is no

indication that the missingness was related to the true outcome values. According to the decision rules (with evidence that missing data did not bias results), this domain is judged as Low Risk.

Domain 4: Risk of Bias in Measurement of the Outcome

- Signaling Question Analysis:
  - 4.1 Was the method of measuring the outcome inappropriate? → No The study used the RAPID instrument—a validated and objective measure of pain-related disability—to determine treatment success.
  - 4.2 Could measurement or ascertainment of the outcome have differed between intervention groups? → No Both intervention groups were assessed using identical procedures and outcome criteria.
  - 4.3 Were outcome assessors aware of the intervention received by study participants? → No Outcome data were collected by blinded research coordinators at the central coordinating center.
  - 4.4 Could assessment of the outcome have been influenced by knowledge of intervention received? → No Blinding of the outcome assessors minimizes the risk of bias in outcome measurement.
  - 4.5 Is it likely that assessment of the outcome was influenced by knowledge of intervention received? → No There is no evidence suggesting that the outcome assessment was affected by treatment awareness.
- Risk Assessment: Low Risk
- Reasoning: The outcome was measured using a validated and appropriate instrument (RAPID score) applied consistently across all groups. Outcome assessors were blinded to treatment allocation, ensuring that measurement procedures were not influenced by knowledge of the intervention. According to the decision rules—where appropriate measurement methods, consistent ascertainment, and blinded assessment lead to a Low Risk judgment—the risk of bias in the measurement of the outcome is Low.

Domain 5: Risk of Bias in Selection of the Reported Result

- Signaling Question Analysis:
  - 5.1 Were the data that produced this result analysed in accordance with a pre-specified analysis plan? → Yes The trial was registered (clinicaltrials.gov Identifier: 00688662) and the methods section specifies a detailed, pre-specified analysis plan (including adjusted and unadjusted analyses, handling of missing data, and ITT analysis), indicating that the plan was finalized before unblinding.
  - 5.2 Is the numerical result being assessed likely to have been selected from multiple eligible outcome measurements? → No The primary outcome (treatment success defined by the RAPID score criteria and additional clinical measures) was clearly pre-specified and consistently reported without evidence of selective outcome measurement.
  - 5.3 Is the numerical result being assessed likely to have been selected from multiple eligible analyses of the data? → No Both primary and sensitivity analyses were reported as planned, with no indication that the results were selectively chosen from a pool of multiple analyses.
- Risk Assessment: Low Risk
- Reasoning: The EPISOD trial adhered to a pre-specified analysis plan finalized prior to unblinding (5.1: Yes), and there is no evidence of selective reporting based on multiple outcome measurements (5.2: No) or analyses (5.3: No). According to the decision rules, with both 5.2 and 5.3 in {N, PN} and a pre-specified plan in 5.1 rated as Yes, the risk of bias in the selection of the reported result is Low Risk.
