# Supplementary material for: Evaluating the Accuracy of Large Language Models in Risk-of-Bias Assessment Using Version 2 of the Cochrane Risk-of-Bias Tool for Randomized Trials: Exploratory Feasibility Study
Source: J Med Internet Res. 2026 Sep 25;28:e84915. doi: 10.2196/84915 (PMC13614218; doi:10.2196/84915)
Supplement: Supplementary file 3 [file jmir-v28-e84915-s003.pdf]

Table S1. The reference standard response for each randomized controlled trial and domain

No.	RCT	Primary Outcome	Randomization process	Deviations from intended interventions	Missing outcome data	Measurement of the outcome	Selection of the reported results	Overall
1	Marenzi 2014	All-cause mortality	Low	Low	Low	Low	Low	Low
2	Liu 2021	Efficacy as a continuous outcome	Low	Low	Low	Low	High	High
3	Katial 2011	Asthma exacerbation	Low	Low	High	Low	Low	High
4	Munteanu 2021	Mean overall pain	Low	Low	Low	Low	Low	Low
5	Lebares 2018	Depression immediately post-intervention	High	High	Low	Low	Low	High
6	Maher 2017	Manual removal of the placenta	Low	Low	Low	Low	Low	Low
7	Daher 2000	All-cause mortality	High	Low	Low	Low	Low	High
8	Toouli 2000	Proportion of participants without treatment success at the longest follow-up (1–4 years)	High	Low	High	Low	High	High
9	Hanna 2012	All-cause mortality	Low	Low	High	High	Low	High
10	Hu 2020	All-cause mortality	Low	Low	Low	Low	Low	Low
11	Tavakoli Ardakani 2019	Efficacy as a continuous outcome	Low	Low	Low	Low	High	High
12	Cavalcanti 2020	All-cause mortality at day 28	Low	Low	Low	Low	Low	Low
13	Peters 2016	Asthma exacerbation	Low	Low	Low	Low	Low	Low
14	Damião Neto 2020	Anxiety immediately post-intervention	High	High	Low	High	High	High
15	Omrani 2020	All-cause mortality at day 28	Low	Low	Low	Low	Low	Low
16	Erogul 2014	Stress immediately post-intervention	Low	High	Low	High	High	High
17	Sekhavati 2020	All-cause mortality at day 28	High	High	Low	Low	Low	High

18	Wang 2021	Major postoperative complications	Low	Low	Low	High	Low	High
19	Van Heeringen 1996	Efficacy as a continuous outcome	High	Low	High	Low	High	High
20	Bart 2005	All-cause mortality	Low	Low	Low	Low	Low	Low
21	RECOVERY 2021	All-cause mortality at day 28	Low	Low	Low	Low	Low	Low
22	Stralen 2013	Manual removal of the placenta	High	Low	Low	Low	High	High
23	Takezawa 2004	Proportion of participants with serious adverse events (recurrent common bile duct stones, at 1 year)	Low	High	High	Low	High	High
24	Woodcock 2014	Asthma exacerbation	Low	Low	Low	Low	Low	Low
25	Seker 2016	All-cause mortality	Low	High	Low	Low	Low	High
26	Lee 2020	Asthma exacerbation	Low	Low	Low	Low	Low	Low
27	Schreiber 2007	Failure to maintain clinical remission at week 26	Low	Low	Low	Low	Low	Low
28	Hinks 2020	All-cause mortality at day 28	Low	High	Low	Low	Low	High
29	Cotton 2014	Proportion of participants without treatment success at the longest follow-up (1–4 years)	Low	Low	Low	Low	Low	Low

Table S2. Thresholds to interpretation of Cohen's kappa [30]

Cohen's Kappa	Interpretation
0	No agreement
0.01-0.20	Slight agreement
0.21-0.40	Fair agreement
0.41-0.60	Moderate agreement
0.61-0.80	Substantial agreement
0.81-0.99	Near-perfect agreement
1	Perfect agreement

Table S3. The results of assessment for each domain and RCT

No.	RCT	Response	Randomization process	Deviations from intended interventions	Missing outcome data	Measurement of the outcome	Selection of the reported results	Overall
1	Marenzi 2014	Response 1	Low	Low	Low	Low	Low	Low
1	Marenzi 2014	Response 2	Low	Low	Low	Low	Low	Low
2	Liu 2021	Response 1	High	Low	Low	High	High	High
2	Liu 2021	Response 2	High	Low	Low	Low	High	High
3	Katial 2011	Response 1	High	Low	High	Low	High	High
3	Katial 2011	Response 2	Low	Low	Low	Low	Low	Low
4	Munteanu 2021	Response 1	Low	Low	Low	Low	Low	Low
4	Munteanu 2021	Response 2	Low	Low	Low	Low	Low	Low
5	Lebares 2018	Response 1	Low	Low	Low	High	Low	High
5	Lebares 2018	Response 2	Low	Low	Low	High	Low	High
6	Maher 2017	Response 1	Low	High	Low	Low	Low	High
6	Maher 2017	Response 2	Low	Low	Low	Low	Low	Low
7	Daher 2000	Response 1	High	Low	High	Low	High	High
7	Daher 2000	Response 2	High	Low	Low	Low	High	High
8	Toouli 2000	Response 1	High	High	Low	Low	High	High
8	Toouli 2000	Response 2	High	Low	Low	Low	High	High
9	Hanna 2012	Response 1	Low	Low	High	Low	Low	High
9	Hanna 2012	Response 2	Low	Low	Low	Low	High	High
10	Hu 2020	Response 1	High	Low	Low	Low	High	High
10	Hu 2020	Response 2	High	Low	Low	Low	High	High
11	Tavakoli Ardakani 2019	Response 1	Low	High	Low	Low	Low	High
11	Tavakoli Ardakani 2019	Response 2	High	High	Low	Low	Low	High
12	Cavalcanti 2020	Response 1	Low	Low	Low	Low	Low	Low
12	Cavalcanti 2020	Response 2	Low	Low	Low	Low	Low	Low

13	Peters 2016	Response 1	Low	Low	Low	Low	Low	Low
13	Peters 2016	Response 2	Low	Low	Low	Low	Low	Low
14	Damião Neto 2020	Response 1	High	Low	Low	High	Low	High
14	Damião Neto 2020	Response 2	High	Low	High	High	Low	High
15	Omrani 2020	Response 1	Low	Low	Low	Low	Low	Low
15	Omrani 2020	Response 2	Low	Low	Low	Low	Low	Low
16	Erogul 2014	Response 1	High	High	Low	High	High	High
16	Erogul 2014	Response 2	High	Low	Low	High	High	High
17	Sekhavati 2020	Response 1	High	Low	Low	Low	High	High
17	Sekhavati 2020	Response 2	High	Low	Low	Low	High	High
18	Wang 2021	Response 1	High	Low	Low	Low	Low	High
18	Wang 2021	Response 2	High	Low	Low	Low	Low	High
19	Van Heeringen 1996	Response 1	Low	Low	High	Low	High	High
19	Van Heeringen 1996	Response 2	Low	Low	High	Low	High	High
20	Bart 2005	Response 1	High	High	Low	Low	High	High
20	Bart 2005	Response 2	High	Low	Low	Low	High	High
21	RECOVERY 2021	Response 1	Low	High	Low	Low	Low	High
21	RECOVERY 2021	Response 2	Low	Low	Low	Low	Low	Low
22	Stralen 2013	Response 1	High	Low	Low	Low	Low	High
22	Stralen 2013	Response 2	High	Low	Low	Low	Low	High
23	Takezawa 2004	Response 1	High	High	High	Low	High	High
23	Takezawa 2004	Response 2	High	High	High	Low	High	High
24	Woodcock 2014	Response 1	Low	Low	High	Low	Low	High

24	Woodcock 2014	Response 2	Low	Low	High	Low	Low	High
25	Seker 2016	Response 1	High	High	High	Low	High	High
25	Seker 2016	Response 2	High	High	High	Low	High	High
26	Lee 2020	Response 1	Low	Low	Low	Low	Low	Low
26	Lee 2020	Response 2	Low	Low	Low	Low	Low	Low
27	Schreiber 2007	Response 1	Low	Low	High	Low	Low	High
27	Schreiber 2007	Response 2	Low	Low	High	Low	Low	Low
28	Hinks 2020	Response 1	High	Low	High	Low	Low	High
28	Hinks 2020	Response 2	High	High	Low	Low	Low	High
29	Cotton 2014	Response 1	Low	Low	Low	Low	Low	Low
29	Cotton 2014	Response 2	Low	Low	Low	Low	Low	Low

Table S4. The study-specific accuracy of assessments

No.	RCT	Type	Response	TP	TN	FP	FN	Accuracy	Sensitivity	Specificity	F-score
1	Marenzi 2014	Heart	Response 1	0	5	0	0	100.0%	Not Available	1.00	Not Available
1	Marenzi 2014	Heart	Response 2	0	5	0	0	100.0%	Not Available	1.00	Not Available
2	Liu 2021	Psychology	Response 1	1	2	2	0	60.0%	1.00	0.50	0.50
2	Liu 2021	Psychology	Response 2	1	3	1	0	80.0%	1.00	0.75	0.67
3	Katial 2011	Lung	Response 1	1	2	2	0	60.0%	1.00	0.50	0.50
3	Katial 2011	Lung	Response 2	0	4	0	1	80.0%	0.00	1.00	Not Available
4	Munteanu 2021	Bone	Response 1	0	5	0	0	100.0%	Not Available	1.00	Not Available
4	Munteanu 2021	Bone	Response 2	0	5	0	0	100.0%	Not Available	1.00	Not Available
5	Lebares 2018	Psychology	Response 1	0	2	1	2	40.0%	0.00	0.67	Not Available
5	Lebares 2018	Psychology	Response 2	0	2	1	2	40.0%	0.00	0.67	Not Available
6	Maher 2017	OBGYN	Response 1	0	4	1	0	80.0%	Not Available	0.80	Not Available
6	Maher 2017	OBGYN	Response 2	0	5	0	0	100.0%	Not Available	1.00	Not Available
7	Daher 2000	Infection	Response 1	1	2	2	0	60.0%	1.00	0.50	0.50
7	Daher 2000	Infection	Response 2	1	3	1	0	80.0%	1.00	0.75	0.67
8	Toouli 2000	GI	Response 1	2	1	1	1	60.0%	0.67	0.50	0.67
8	Toouli 2000	GI	Response 2	2	2	0	1	80.0%	0.67	1.00	0.80
9	Hanna 2012	Heart	Response 1	1	3	0	1	80.0%	0.50	1.00	0.67
9	Hanna 2012	Heart	Response 2	0	2	1	2	40.0%	0.00	0.67	Not Available
10	Hu 2020	Heart	Response 1	0	3	2	0	60.0%	Not Available	0.60	Not Available
10	Hu 2020	Heart	Response 2	0	3	2	0	60.0%	Not Available	0.60	Not Available
11	Tavakoli Ardakani 2019	Psychology	Response 1	0	3	1	1	60.0%	0.00	0.75	Not Available

11	Tavakoli Ardakani 2019	Psychology	Response 2	0	2	2	1	40.0%	0.00	0.50	Not Available
12	Cavalcanti 2020	Infection	Response 1	0	5	0	0	100.0%	Not Available	1.00	Not Available
12	Cavalcanti 2020	Infection	Response 2	0	5	0	0	100.0%	Not Available	1.00	Not Available
13	Peters 2016	Lung	Response 1	0	5	0	0	100.0%	Not Available	1.00	Not Available
13	Peters 2016	Lung	Response 2	0	5	0	0	100.0%	Not Available	1.00	Not Available
14	Damião Neto 2020	Psychology	Response 1	2	1	0	2	60.0%	0.50	1.00	0.67
14	Damião Neto 2020	Psychology	Response 2	2	0	1	2	40.0%	0.50	0.00	0.57
15	Omrani 2020	Infection	Response 1	0	5	0	0	100.0%	Not Available	1.00	Not Available
15	Omrani 2020	Infection	Response 2	0	5	0	0	100.0%	Not Available	1.00	Not Available
16	Erogul 2014	Psychology	Response 1	3	1	1	0	80.0%	1.00	0.50	0.86
16	Erogul 2014	Psychology	Response 2	2	1	1	1	60.0%	0.67	0.50	0.67
17	Sekhavati 2020	Infection	Response 1	1	2	1	1	60.0%	0.50	0.67	0.50
17	Sekhavati 2020	Infection	Response 2	1	2	1	1	60.0%	0.50	0.67	0.50
18	Wang 2021	GI	Response 1	0	3	1	1	60.0%	0.00	0.75	Not Available
18	Wang 2021	GI	Response 2	0	3	1	1	60.0%	0.00	0.75	Not Available
19	Van Heeringen 1996	Psychology	Response 1	2	2	0	1	80.0%	0.67	1.00	0.80
19	Van Heeringen 1996	Psychology	Response 2	2	2	0	1	80.0%	0.67	1.00	0.80
20	Bart 2005	Heart	Response 1	0	2	3	0	40.0%	Not Available	0.40	Not Available

20	Bart 2005	Heart	Response 2	0	3	2	0	60.0%	Not Available	0.60	Not Available
21	RECOVERY 2021	Infection	Response 1	0	4	1	0	80.0%	Not Available	0.80	Not Available
21	RECOVERY 2021	Infection	Response 2	0	5	0	0	100.0%	Not Available	1.00	Not Available
22	Stralen 2013	OBGYN	Response 1	1	3	0	1	80.0%	0.50	1.00	0.67
22	Stralen 2013	OBGYN	Response 2	1	3	0	1	80.0%	0.50	1.00	0.67
23	Takezawa 2004	GI	Response 1	3	1	1	0	80.0%	1.00	0.50	0.86
23	Takezawa 2004	GI	Response 2	3	1	1	0	80.0%	1.00	0.50	0.86
24	Woodcock 2014	Lung	Response 1	0	4	1	0	80.0%	Not Available	0.80	Not Available
24	Woodcock 2014	Lung	Response 2	0	4	1	0	80.0%	Not Available	0.80	Not Available
25	Seker 2016	Heart	Response 1	1	1	3	0	40.0%	1.00	0.25	0.40
25	Seker 2016	Heart	Response 2	1	1	3	0	40.0%	1.00	0.25	0.40
26	Lee 2020	Lung	Response 1	0	5	0	0	100.0%	Not Available	1.00	Not Available
26	Lee 2020	Lung	Response 2	0	5	0	0	100.0%	Not Available	1.00	Not Available
27	Schreiber 2007	GI	Response 1	0	4	1	0	80.0%	Not Available	0.80	Not Available
27	Schreiber 2007	GI	Response 2	0	4	1	0	80.0%	Not Available	0.80	Not Available
28	Hinks 2020	Infection	Response 1	0	2	2	1	40.0%	0.00	0.50	Not Available
28	Hinks 2020	Infection	Response 2	1	3	1	0	80.0%	1.00	0.75	0.67
29	Cotton 2014	GI	Response 1	0	5	0	0	100.0%	Not Available	1.00	Not Available
29	Cotton 2014	GI	Response 2	0	5	0	0	100.0%	Not Available	1.00	Not Available

Table S5. The domain-specific consistency between two assessments

Domain	Agreement across two assessments	Proportion of observed agreement	Cohen Kappa	PABAκ
Domain 1	27	93.1%	0.86	0.86
Domain 2	23	79.3%	0.39	0.59
Domain 3	24	82.8%	0.56	0.66
Domain 4	28	96.6%	0.84	0.93
Domain 5	27	93.1%	0.85	0.86

Abbreviation: PABAκ = Prevalence- and bias-adjusted kappa

Table S6. The study-specific consistency between two assessments

No.	RCT	Agreement across two assessments	Proportion of observed agreement	Cohen Kappa	PABAκ
1	Marenzi 2014	5	100.0%	Not available	1
2	Liu 2021	4	80.0%	0.62	0.6
3	Katial 2011	2	40.0%	0.00	-0.2
4	Munteanu 2021	5	100.0%	Not available	1
5	Lebares 2018	5	100.0%	1.00	1
6	Maher 2017	4	80.0%	0.00	0.6
7	Daher 2000	4	80.0%	0.62	0.6
8	Toouli 2000	4	80.0%	0.62	0.6
9	Hanna 2012	3	60.0%	-0.25	0.2
10	Hu 2020	5	100.0%	1.00	1
11	Tavakoli Ardakani 2019	4	80.0%	0.55	0.6
12	Cavalcanti 2020	5	100.0%	Not available	1
13	Peters 2016	5	100.0%	Not available	1
14	Damião Neto 2020	4	80.0%	0.62	0.6
15	Omrani 2020	5	100.0%	Not available	1
16	Erogul 2014	4	80.0%	0.55	0.6
17	Sekhavati 2020	5	100.0%	1.00	1
18	Wang 2021	5	100.0%	1.00	1
19	Van Heeringen 1996	5	100.0%	1.00	1
20	Bart 2005	4	80.0%	0.62	0.6
21	RECOVERY 2021	4	80.0%	0.00	0.6
22	Stralen 2013	5	100.0%	1.00	1
23	Takezawa 2004	5	100.0%	1.00	1
24	Woodcock 2014	5	100.0%	1.00	1
25	Seker 2016	5	100.0%	1.00	1
26	Lee 2020	5	100.0%	Not available	1
27	Schreiber 2007	5	100.0%	1.00	1
28	Hinks 2020	3	60.0%	0.17	0.2
29	Cotton 2014	5	100.0%	Not available	1

Abbreviation: PABAκ = Prevalence-adjusted bias-adjusted κ

References:

1. Marenzi G, Muratori M, Cosentino ER, Rinaldi ER, Donghi V, Milazzo V, et al. Continuous ultrafiltration for congestive heart failure: the CUORE trial. J Card Fail. 2014;20(1):9-17. PMID:24269855 doi:10.1016/j.cardfail.2013.11.004
2. Liu P, Li P, Li Q, Yan H, Shi X, Liu C, et al. Effect of pretreatment of S-ketamine on postoperative depression for breast cancer patients. J Invest Surg. 2021;34(8):883-888. PMID:31948296 doi:10.1080/08941939.2019.1710626
3. Katial RK, Bernstein D, Prazma CM, Lincourt WR, Stempel DA. Long-term treatment with fluticasone propionate/salmeterol via Diskus improves asthma control versus fluticasone propionate alone. Allergy Asthma Proc. 2011;32(2):127-136. PMID:21189151 doi:10.2500/aap.2011.32.3426
4. Munteanu SE, Landorf KB, McClelland JA, Roddy E, Cicuttini FM, Shiell A, et al. Shoe-stiffening inserts for first metatarsophalangeal joint osteoarthritis: a randomised trial. Osteoarthritis Cartilage. 2021;29(4):480-490. PMID:33588086 doi:10.1016/j.joca.2021.02.002
5. Lebares CC, Hershberger AO, Guvva EV, Desai A, Mitchell J, Shen W, et al. Feasibility of formal mindfulness-based stress-resilience training among surgery interns: a randomized clinical trial. JAMA Surg. 2018;153(10):e182734. PMID:30167655 doi:10.1001/jamasurg.2018.2734
6. Maher MA, Sayyed TM, Elkhouly NI. Different routes and forms of uterotonics for treatment of retained placenta: a randomized clinical trial [Retracted]. J Matern Fetal Neonatal Med. 2017;30(18):2179-2184. PMID:27677547 doi:10.1080/14767058.2016.1242124
7. Daher EF, Nogueira CB. Evaluation of penicillin therapy in patients with leptospirosis and acute renal failure. Rev Inst Med Trop Sao Paulo. 2000;42(6):327-332. PMID:11136519 doi:10.1590/s0036-46652000000600005
8. Toouli J, Roberts-Thomson IC, Kellow J, Dowsett J, Saccone GT, Evans P, et al. Manometry based randomised trial of endoscopic sphincterotomy for sphincter of Oddi dysfunction. Gut. 2000;46(1):98-102. PMID:10601063 doi:10.1136/gut.46.1.98
9. Hanna MA, Tang WH, Teo BW, O'Neill JO, Weinstein DM, Lau SM, et al. Extracorporeal ultrafiltration vs conventional diuretic therapy in advanced decompensated heart failure. Congest Heart Fail. 2012;18(1):54-63. PMID:22277179 doi:10.1111/j.1751-7133.2011.00231.x
10. Hu J, Wan Q, Zhang Y, Zhou J, Li M, Jiang L, et al. Efficacy and safety of early ultrafiltration in patients with acute decompensated heart failure with volume overload: a prospective, randomized, controlled clinical trial. BMC Cardiovasc Disord. 2020;20(1):447. PMID:33054727 doi:10.1186/s12872-020-01733-5
11. Tavakoli Ardakani M, Mehrpooya M, Mehdizadeh M, Beiraghi N, Hajifathali A, Kazemi MH, et al. Sertraline treatment decreased the serum levels of interleukin-6 and high-sensitivity C-reactive protein in hematopoietic stem cell transplantation patients with depression: a randomized double-blind, placebo-controlled clinical trial. Bone Marrow Transplant. 2020;55(4):830-832. PMID:31383995 doi:10.1038/s41409-019-0623-0
12. Cavalcanti AB, Zampieri FG, Rosa RG, Azevedo LCP, Veiga VC, Avezum A, et al; Coalition Covid-19 Brazil I Investigators. Hydroxychloroquine with or without azithromycin in mild-to-moderate Covid-19. N Engl J Med. 2020;383(21):2041-2052. PMID:32706953 doi:10.1056/NEJMoa2019014
13. Peters SP, Bleecker ER, Canonica GW, Park YB, Ramirez R, Hollis S, et al. Serious asthma events with budesonide plus formoterol vs budesonide alone. N Engl J Med. 2016;375(9):850-860. PMID:27579635 doi:10.1056/NEJMoa1511190
14. Damião Neto A, Lucchetti ALG, da Silva Ezequiel O, Lucchetti G. Effects of a required large-group mindfulness meditation course on first-year medical students' mental health and quality of life: a randomized controlled trial. J Gen Intern Med. 2020;35(3):672-678. PMID:31452038 doi:10.1007/s11606-019-05284-0

15. Omrani AS, Pathan SA, Thomas SA, Harris TRE, Coyle PV, Thomas CE, et al. Randomized double-blinded placebo-controlled trial of hydroxychloroquine with or without azithromycin for virologic cure of non-severe Covid-19. EClinicalMedicine. 2020;29:100645. PMID:33251500 doi:10.1016/j.eclinm.2020.100645
16. Erogul M, Singer G, McIntyre T, Stefanov DG. Abridged mindfulness intervention to support wellness in first-year medical students. Teach Learn Med. 2014;26(4):350-356. PMID:25318029 doi:10.1080/10401334.2014.945025
17. Sekhavati E, Jafari F, SeyedAlinaghi S, Jamalimoghadamsiahkali S, Sadr S, Tabarestani M, et al. Safety and effectiveness of azithromycin in patients with COVID-19: an open-label randomised trial. Int J Antimicrob Agents. 2020;56(4):106143. PMID:32853672 doi:10.1016/j.ijantimicag.2020.106143
18. Wang J, Wang Q, Dong J, Yang K, Ji S, Fan Y, et al. Total laparoscopic uncut Roux-en-Y for radical distal gastrectomy: an interim analysis of a randomized, controlled, clinical trial. Ann Surg Oncol. 2021;28(1):90-96. PMID:32556870 doi:10.1245/s10434-020-08710-4
19. van Heeringen K, Zivkov M. Pharmacological treatment of depression in cancer patients: a placebo-controlled study of mianserin. Br J Psychiatry. 1996;169(4):440-443. PMID:8894194 doi:10.1192/bjp.169.4.440
20. Bart BA, Boyle A, Bank AJ, Anand I, Olivari MT, Kraemer M, et al. Ultrafiltration versus usual care for hospitalized patients with heart failure: the Relief for Acutely Fluid-Overloaded Patients With Decompensated Congestive Heart Failure (RAPID-CHF) trial. J Am Coll Cardiol. 2005;46(11):2043-2046. PMID:16325039 doi:10.1016/j.jacc.2005.05.098
21. RECOVERY Collaborative Group. Azithromycin in patients admitted to hospital with COVID-19 (RECOVERY): a randomised, controlled, open-label, platform trial. Lancet. 2021;397(10274):605-612. PMID:33545096 doi:10.1016/S0140-6736(21)00149-5
22. van Stralen G, Veenhof M, Holleboom C, van Roosmalen J. No reduction of manual removal after misoprostol for retained placenta: a double-blind, randomized trial. Acta Obstet Gynecol Scand. 2013;92(4):398-403. PMID:23231499 doi:10.1111/aogs.12065
23. Takezawa M, Kida Y, Kida M, Saigenji K. Influence of endoscopic papillary balloon dilation and endoscopic sphincterotomy on sphincter of Oddi function: a randomized controlled trial. Endoscopy. 2004;36(7):631-637. PMID:15243887 doi:10.1055/s-2004-814538
24. Woodcock A, Lötvall J, Busse WW, Bateman ED, Stone S, Ellsworth A, et al. Efficacy and safety of fluticasone furoate 100 µg and 200 µg once daily in the treatment of moderate-severe asthma in adults and adolescents: a 24-week randomised study. BMC Pulm Med. 2014;14:113. PMID:25007865 doi:10.1186/1471-2466-14-113
25. Şeker A, Kayataş M, Hüzmeli C, Candan F, Yılmaz MB. Comparison of ultrafiltration and intravenous diuretic therapies in patients hospitalized for acute decompensated biventricular heart failure. Turk J Nephrol. Jan 2019;25(1):79-87. URL: https://www.turkjnephrol.org/index.php/pub/article/view/1036 [Accessed 2026-08-28]
26. Lee LA, Bailes Z, Barnes N, Boulet LP, Edwards D, Fowler A, et al. Efficacy and safety of once-daily single-inhaler triple therapy (FF/UMEC/VI) versus FF/VI in patients with inadequately controlled asthma (CAPTAIN): a double-blind, randomised, phase 3A trial. Lancet Respir Med. 2021;9(1):69-84. PMID:32918892 doi:10.1016/S2213-2600(20)30389-1
27. Schreiber S, Khaliq-Kareemi M, Lawrance IC, Thomsen OØ, Hanauer SB, McColm J, et al; PRECISE 2 Study Investigators. Maintenance therapy with certolizumab pegol for Crohn's disease. N Engl J Med. 2007;357(3):239-250. PMID:17634459 doi:10.1056/NEJMoa062897
28. Hinks TSC, Barber VS, Black J, Dutton SJ, Jabeen M, Melhorn J, et al. A multi-centre open-label two-arm randomised superiority clinical trial of azithromycin versus usual care in ambulatory COVID-19: study protocol

for the ATOMIC2 trial. Trials. 2020;21(1):718. PMID:32807209 doi:10.1186/s13063-020-04593-8
29. Cotton PB, Durkalski V, Romagnuolo J, Pauls Q, Fogel E, Tarnasky P, et al. Effect of endoscopic sphincterotomy for suspected sphincter of Oddi dysfunction on pain-related disability following cholecystectomy: the EPISOD randomized clinical trial. JAMA. 2014;311(20):2101-2109. PMID:24867013 doi:10.1001/jama.2014.5220
30. Zach. Cohen's Kappa Statistic: Definition & Example. Statology. Published February 22, 2021. https://www.statology.org/cohens-kappa-statistic/. [accessed 7 May, 2025].
